# Families’ and clinicians’ experiences with telehealth assessments for autism: A mixed-methods systematic review

**DOI:** 10.1371/journal.pdig.0000931

**Published:** 2025-07-29

**Authors:** Panos Katakis, Paige Frankson, Georgia Lockwood Estrin, Jeanne Wolstencroft, Venus Mirzaei, Shermina Sayani, David Skuse, Michelle Heys

**Affiliations:** 1 East London NHS Foundation Trust, London, United Kingdom; 2 Great Ormond Street Institute of Child Health, University College London, London, United Kingdom; 3 School of Health & Wellbeing, College of Medical, Veterinary and Life Sciences, University of Glasgow, Glasgow, United Kingdom; 4 NHS Highland, Inverness, United Kingdom; 5 School of Life and Medical Sciences, University of Hertfordshire, Hatfield, United Kingdom; 6 School of Psychology, University of East London, London, United Kingdom; The University of Hong Kong, HONG KONG

## Abstract

Recently, the utilization of telehealth for the evaluation of autism spectrum disorder (ASD) in children has increased considerably. Although past studies have explored the feasibility and validity of telehealth assessment procedures for ASD, the acceptability and perspectives of families and clinicians regarding telehealth for autism evaluations have not yet been systematically examined. This mixed-methods systematic review aimed to synthesize the available evidence to understand the experiences of families and clinicians with telehealth. We followed the Joanna Briggs Institute methodology guidelines for conducting mixed-method systematic reviews using the convergent integrated approach. We searched relevant databases (EMBASE, MEDLINE, PsycINFO, CINAHL, ASSIA) and other sources (e.g., grey literature) to identify eligible articles (PROSPERO: CRD42022332500). Data from eligible studies were pooled and subjected to thematic synthesis. In total, 27 studies were included in this review, involving 1013 caregivers and 521 clinicians who shared their perceptions and experiences with telehealth. Overall, participants were highly satisfied with telehealth procedures and noted several advantages, including increased convenience, flexibility, and efficiency (e.g., reduced costs and travel time), improved service provision and access to timely care, and enhanced clinical effectiveness. However, certain disadvantages, such as technical difficulties, difficulties observing certain behaviors, perceived lack of accuracy, concerns about the family’s role and safeguarding issues, among others, were also reported. Telehealth was believed to improve equity for some families (i.e., geographically remote families) while potentially disadvantaging others (i.e., socioeconomically disadvantaged families and those with limited English proficiency). Children who were older, less active, less medically and psychosocially complex and those with a clearer presentation of ASD were considered more suitable for a telehealth evaluation for ASD. In conclusion, this review provides new insights into the experiences of families and clinicians with telehealth, highlighting its potential uses for ASD evaluations and identifying areas for improvement and future research.

## Author summary

Although assessments for autism traditionally occur in person in clinical settings, in the last few years, many services have explored ways to conduct these evaluations remotely using telehealth. In this review, we searched scientific sources extensively for articles exploring clinicians’ and families’ experiences with telehealth assessments for autism in children. Both groups described several advantages associated with telehealth, including increased flexibility, convenience, efficiency, service improvement, and timely access for families. However, some disadvantages were also reported, such as difficulties with technology, a perceived lack of accuracy, and challenges navigating complex cases virtually. Participants also believed that, although telehealth can help services reach families residing in remote areas, this approach may disadvantage less affluent families and those less proficient in English. Older children, those presenting with more pronounced autistic traits, and children without additional medical or psychosocial difficulties and needs were considered more suitable for a telehealth evaluation. While telehealth has the potential to significantly improve services and access for autistic children and their families, further research is needed to optimize virtual diagnostic procedures and to broaden accessibility so that a wider range of the population may benefit from these advancements.

## Introduction

Autism Spectrum Disorder (ASD) is a complex neurodevelopmental condition associated with difficulties in social communication and interaction, restricted interests and repetitive behaviors, and differences in sensory processing [1]. Globally, approximately 0.7% to 1% of the general population meets the diagnostic criteria for ASD [2–4]. Early features of autism can be identified by 18 months [5], and ASD can be reliably diagnosed by the age of two [6]. A multi-stage assessment process is employed to diagnose ASD [7]. Children are often initially identified by relevant screening tools [8,9]. Children scoring above the cut-off during screening and those with developmental concerns are typically referred for a comprehensive diagnostic evaluation of autism, consisting of a developmental interview with families and a structured behavioral observation of the child [10]. Information from other sources (e.g., cognitive or adaptive assessments) also informs the diagnostic decision-making process.

### Limitations of traditional assessment procedures for autism

The current diagnostic procedures for autism present several limitations. Although the importance of early diagnosis has been emphasized [11], significant delays have been documented globally, with children receiving a diagnosis at an average age of approximately 60 months [12].

Multiple factors contribute to these diagnostic delays. Globally, neurodevelopmental services face extensive waiting lists [13,14]. This possibly reflects the inefficiencies of the conventional diagnostic pathways for autism in combination with insufficient capacity to meet increased demand. Regarding the former, the current assessment procedures are remarkably resource-intensive, involving lengthy clinical appointments [15] that incur substantial costs for services [16]. In addition, the administration of gold-standard diagnostic tools for autism, such as the Autism Diagnostic Observation Schedule-Second Edition (ADOS-2) [17], requires extensive and costly training. Factors related to the child’s presentation and symptom severity [18–20], the lack of sufficient pre-assessment information [21], and previous misdiagnoses can engender further delays [22–24].

Diagnostic delays are more pronounced in families from ethnic minority groups, those with limited English proficiency in English-speaking countries and socially and economically disadvantaged families [25]. For example, high-income families are more likely to have their children diagnosed earlier compared to low-resourced families [18,19]. Families in rural and remote areas are also disadvantaged, as they are required to travel long distances to reach neurodevelopmental services [26–28]. Families from ethnic minority backgrounds and those not fluent in English also face several barriers accessing and utilizing appropriate services [29–31]. Lastly, pandemic-related social restrictions can further exacerbate existing delays [32].

### Assessing autism via telehealth

Lately, telehealth has been widely used across many fields of medicine and is considered the “new normal” [33,34]. In the context of assessing autism in children, clinicians have employed both synchronous and asynchronous means [35]. Asynchronous, or store-and-forward, methods include the utilization of videos captured in the home environment or other settings (e.g., school) to code for autistic behaviors and traits. Evidence suggests that some autistic phenotypes can be identified through video observation [36–38], and novel tools, such as the Naturalistic Observation Diagnostic Assessment (NODA) [39], have been used to assess for autism via video material. Synchronous assessments include real-time consultations via video-conferencing technology, using either adapted gold-standard tools [40,41] or novel tools, such as the TELE-ASD-PEDS, developed specifically for telehealth use [42].

Several reviews have concluded that screening and diagnostic evaluations for autism are feasible and largely equivalent to face-to-face assessments in clinical settings [43–49]. A recent review also found that virtual assessment procedures demonstrated, in most cases, excellent psychometric properties, with a diagnostic agreement of 80-88.2% with the outcomes from in-person assessments [35]. However, most studies demonstrated low quality, included small samples, and were conducted in high-income, Western settings, limiting their usefulness for understanding the context across different populations.

### The current review

Although several reviews have explored the feasibility, validity and diagnostic accuracy of telehealth tools, stakeholders’ experiences with using telehealth for autism assessments in children have not yet been systematically examined. Therefore, this review aimed to a) explore the perceived advantages and disadvantages of utilizing telehealth for the assessment of autism among clinicians and families, b) understand issues related to equity and telehealth for the diagnostic assessment of autism, and c) determine which groups of children are most suitable for telehealth assessment. We reviewed both quantitative and qualitative data and synthesized the mixed-methods evidence with the aim of understanding stakeholders’ experiences and providing practical suggestions for further research and clinical practice.

## Methods

### Design

To develop this systematic review, the Preferred Reporting Items for Systematic reviews and Meta-Analyses (PRISMA) guidelines were followed [50], and a protocol was registered with PROSPERO (CRD42022332500). This systematic review was conducted as part of the Children’s Autism Technology-Assisted Assessments (CHATA) project, which aims to develop and evaluate a novel online diagnostic assessment pathway for autism for ethnically diverse preschool children in London, UK [51].

For this systematic review, the Joanna Briggs Institute (JBI) methodology guidelines for conducting mixed-method systematic reviews were followed [52]. Since this study explores topics (e.g., advantages and challenges) that can be studied using both quantitative and qualitative methods, the convergent integrated approach of the JBI was followed. This allows for the exploration and synthesis of both quantitative and qualitative data.

### Search strategy

To identify eligible studies, a rigorous search strategy was developed, using multiple sources. The following bibliographic databases were searched: EMBASE, MEDLINE, PsycINFO, Cumulative Index to Nursing and Allied Health Literature (CINAHL) and Applied Social Sciences Index and Abstracts (ASSIA). Relevant publishers (e.g., Taylor & Francis, Elsevier) and leading journals in the field were also manually searched. A grey literature search was conducted to identify unpublished studies and dissertations using OpenGrey, EThOS, and ProQuest Dissertations & Theses. Additionally, the articles included in this review and relevant systematic reviews were hand-searched to identify further articles.

The databases were searched using the following terms: (autism spectrum disorder* OR ASD OR autis* OR autis* disorder OR Asperger’s Syndrome OR pervasive developmental disorder OR PDD-NOS) AND (online OR digital OR telehealth OR virtual OR remote) AND (assessment OR diagnosis OR identification OR screening) AND (satisfaction OR feasibility OR focus group OR interview OR experienc* OR attitud* OR qualitative OR expectat* OR concern* OR advantag* OR disadvantag* OR benef* OR challeng* OR view* OR perspectiv* OR barrier* OR enabl* OR positive* OR negative* OR limitation*).

### Selection criteria

Articles from inception until 14 October 2023 were retrieved. No limitations were set regarding the language of publication or the setting in which the study was conducted. Given that our search strategy prioritized sensitivity over specificity, the PICOS (Population, Intervention, Comparison, Outcome, and Study Design) framework was utilized to develop the inclusion and exclusion criteria [53]. The eligibility criteria for each PICOS domain can be found in Table 1.

**Table 1:** PICOS criteria.

Domain	Inclusion Criteria	Exclusion Criteria
**Population**	Caregivers of children who underwent an assessment of autism via telehealth Clinicians who administered a telehealth evaluation for autism for children	Adults undergoing an assessment for autism Caregivers and clinicians who have not participated in a telehealth evaluation
**Intervention**	Diagnostic assessment of autism via telehealth Clinician-guided screening for autism via telehealth	Assessment of non-neurodevelopmental conditions Population-based screening for autism
**Comparator**	Diagnostic assessment in person or no comparator	No study was excluded based on the comparator group
**Outcome(s)**	Satisfaction Diagnostic agreement between telehealth and in-person assessment Experiences with telehealth Attitudes towards telehealth	Psychometric properties Cost-effectiveness
**Study design**	Any study design with relevant quantitative or qualitative data	Theoretical papers, conference abstracts commentaries, editorials, reviews

### Study selection and data extraction

All articles retrieved through database search were uploaded to the Rayyan software [54], a tool utilized throughout all the stages of the study selection process. Following deduplication, the records were screened by the first author (PK), while another author (PF) reviewed 20% of the articles to ensure the integrity of the screening process. The concordance between the two reviewers was examined by calculating the percentage agreement and Cohen’s Kappa. Any discrepancies were resolved through discussion between the reviewers, or the broader research team when consensus could not be reached.

Data from each study were extracted by the first author to a standardized extraction form using Microsoft Excel. This included bibliographic information (title, authors, year of publication), information about the setting of the study (city and country), study design, participant information (sample size, age, gender), the tools used, the mode of assessment (synchronous, asynchronous, or mixed), the analyses conducted, and the main results. A second reviewer independently checked all extracted data to ensure accuracy and reduce risk of bias. Any disagreements on extracted data were resolved through discussion.

### Quality assessment

The quality of the included studies was assessed using the Mixed Methods Appraisal Tool (MMAT) [55]. This tool has been widely validated and used in mixed-methods systematic reviews and can be used for the assessment of the quality of a) qualitative studies, b) quantitative randomized controlled trials, c) quantitative non-randomized trials, d) quantitative descriptive studies, and e) mixed-methods studies. Reflecting the guidelines of good practice for thematic synthesis [56], no study was excluded on the basis of quality assessment results. Consistent with JBI guidelines and in light of the lack of consensus in the literature, the level of certainty was not assessed for this review.

### Data synthesis

The data synthesis followed multiple stages. Initially, in line with the convergent integrated approach of the JBI, the data extracted from quantitative studies underwent data transformation; they were “qualitized”, namely transformed from their original form to their narrative interpretation or textual description. The “qualitized” data were subsequently aggregated and analyzed together with the qualitative data from the qualitative studies. Thematic synthesis of the pooled data was conducted by the first author (PK) following Thomas and Harden protocol [57], which involves three stages. Initially, an inductive “line-by-line” coding of the data was conducted. The codes were subsequently categorized as referring to either families or clinicians and grouped into descriptive themes. Generating descriptive themes was a highly reflective and iterative process. The preliminary descriptive themes were reviewed thoroughly by two additional researchers (PF, GLE) and refined. Finally, the descriptive themes were grouped under several overarching analytical themes, some predefined based on the concepts of interest in this review (e.g., advantages and disadvantages of telehealth) and others developed inductively based on the descriptive themes generated. The analysis was conducted using the NVivo 12 software.

## Results

**Fig 1:**
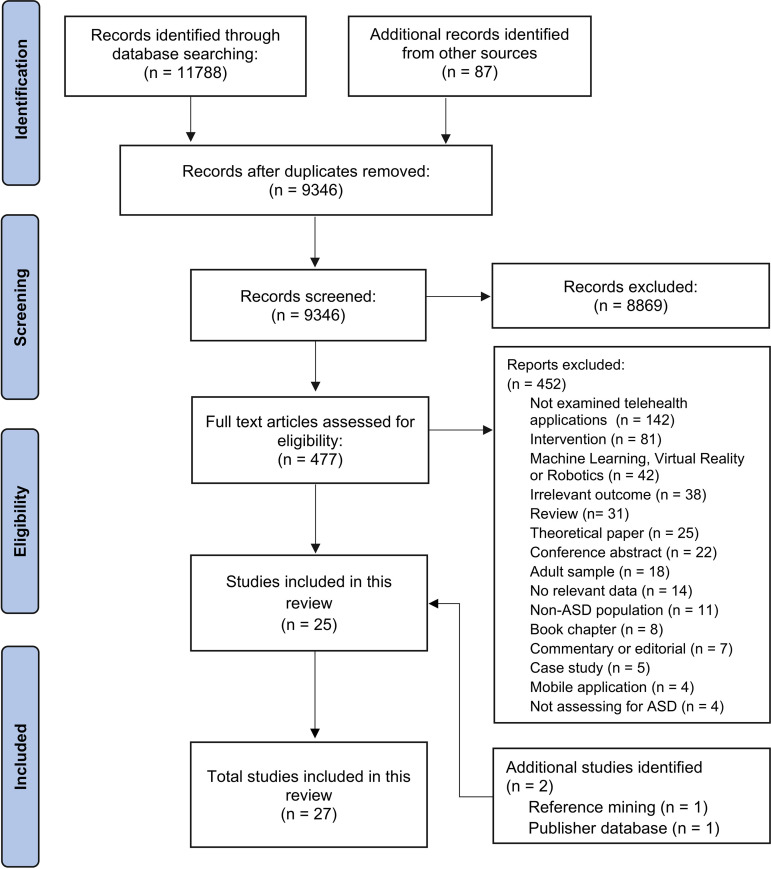
Flowchart of study selection.

A total of 11,788 records were obtained through the database search, and following deduplication, 9346 studies were retained and screened. After excluding irrelevant papers, 477 potentially eligible studies were subjected to full-text screening. The two reviewers exhibited an agreement rate of 93.47% (K = 0.42). From these studies, 452 articles were excluded, leaving 25 studies that met the eligibility criteria and were included in this review. An additional eligible article was identified via a publisher database, and one article cited one of the included studies. In total, 27 studies were included in this review [40,41,58–82]. The study selection procedure is illustrated in the PRISMA flowchart (Fig 1).

### Study characteristics

A detailed summary of the study characteristics is presented in Table 2. Most studies (K = 18) were conducted in the USA, three in Australia, one in the UK, one in Argentina and four studies recruited participants from more than one country. Thirteen studies employed a quantitative study design, two were qualitative, and the remaining 12 studies utilized a mixed-methods design. In most studies (K = 24) the telehealth evaluation was performed utilizing synchronous methods and three studies incorporated both synchronous and asynchronous methods. Regarding the tools used, nine studies reported using the TELE-ASD-PEDS, either as a standalone tool or in conjunction with other instruments, and other studies employed a combination of other tools to assess for autism. In total, 1013 caregivers and 521 clinicians provided data on their experiences participating in a telehealth assessment for autism.

**Table 2:** Study characteristics.

Study	Country	Study design	Setting	Sample	Clinician specialty	Mode of assessment	Telehealth tool(s)^*^	Outcomes
Bain et al. (2021)	USA, Canada	Quantitative acceptability study	Family’s Home	Clinicians: 100	Child Neurologists: 96 Nurse practitioners: 4	Synchronous	N/R	Satisfaction, Challenges, Appropriateness
Pedernera Bradichansky et al. (2021)	Buenos Aires, Argentina	Quantitative acceptability study	Family’s Home	Families: 72	N/A	Synchronous	N/R	Perceptions, Advantages
Corona et al. (2021)	Tennessee, USA	Mixed-methods study	Clinic room	Parents: 52	N/R	Synchronous	TAP, TELE-STAT	Satisfaction, Perceptions
Corona et al. (2024)	Tennessee, USA	Randomized Controlled Trial	Clinic room	Parents: 144 Clinicians: 15	Psychological providers	Synchronous	TAP, TELE-STAT	Satisfaction
Esther et al. (2022)	Sydney, Australia	Mixed-methods study	Family’s Home	Parents: 25	N/A	Synchronous	Diagnostic interview, VABS-3	Advantages, Disadvantages, Preference, Suggestions
Gibbs et al. (2021)	Sydney, Australia	Mixed-methods Study	Family’s Home	Caregivers: 56 Clinicians: 7	Registered psychologists	Synchronous	ADI-R ADOS-2-informed observation	Satisfaction Experiences, Perspectives
Hodge et al. (2024)	New South Wales, Australia	Mixed-methods Study	Family’s Home	Caregivers: 10	N/A	Synchronous	Clinical interview, TAP, VABS-3	Experiences
Jones et al. (2022)	Illinois, USA	Mixed-methods Study	Family’s Home	Parents: 12	N/A	Synchronous	Diagnostic Interview, TAP	Satisfaction, Advantages, Experiences
Juárez et al. (2018)	Tennessee, USA	Pilot study	Family’s Home	Families: 45 Clinicians: 5	Licensed clinical psychologists	Synchronous	Clinical interview STAT	Satisfaction
Kellom et al. (2023)	Multiple states in USA	Mixed-methods Study	Family’s Home	Families: 22 Clinicians: 13	Physicians Psychologists	Synchronous	N/A	Satisfaction, Benefits, Challenges, Equity
Kennelly et al. (2022)	Texas, USA	Quantitative acceptability study	Family’s home	Caregivers: 106	N/A	Synchronous	N/R	Satisfaction
Kryszak et al. (2022)	Multiple States of USA, Canada	Qualitative study	Family’s home	Clinicians: 35	clinical psychologists, developmental behavioral pediatricians, psychiatrists, occupational therapists, nurse practitioners, speech-language pathologists	Synchronous	Diagnostic Interview, ADOS-2	Experiences
Matthews et al. (2021)	Arizona, USA	Mixed-methods study	Family’s home	Parents: 48 Clinicians: 5	Psychologists	Mixed	Clinical Interview, NODA, behavioral observation based on ADOS-2, VABS-3, KBIT-2	Satisfaction Acceptability
McNally Keehn et al. (2022)	Indianapolis, USA	Quantitative acceptability study	Family’s home	Families: 119 Clinicians: 9	Licensed psychologists	Synchronous	Clinical interview Behavioral observation	Satisfaction, Diagnostic certainty, Experiences
McNally Keehn et al. (2023)	Indianapolis, USA	Quantitative acceptability study	Family’s home	Clincians: 11	Licensed psychologists: 7 Pediatricians: 4	Synchronous	Clinical interview TAP, VABS-3	Diagnostic certainty Barriers
Phelps et al. (2022)	Oregon, USA	Quantitative acceptability study	Family’s home	Clinicians: 2	Licensed clinical psychologist, Pre-doctoral Psychology intern	Synchronous	BASC-3 ABAS-3 CARS-2 Behavioral observation	Factors influencing diagnostic outcome
Reese et al. (2013)	Kansas, USA	Pilot study	Medical center	Parents: 10	N/A	Synchronous	ADI-R ADOS	Satisfaction
Reese et al. (2015)	Kansas, USA	Quantitative acceptability study	Medical center	Parents: 64	N/A	Synchronous	20-minute unstructured play Modified ADOS2 ADI-R algorithm items, Medical and family history	Satisfaction
Reisinger et al. (2022)	Indianapolis, USA	Quantitative acceptability study	Family’s home	Parents: 141 Clinicians: 11	Psychologists: 7 Pediatricians: 4	Synchronous	TAP	Satisfaction
Spain et al. (2022a)	USA, UK, Europe, Argentina, Australia	Mixed-methods study	Family’s home	Clinicians: 52	MDT professionals	Mixed	Various tools	Experiences
Spain et al. (2022b)	UK	Qualitative study	Family’s home	Clinicians: 45	MDT professionals	Mixed	Various tools	Experiences, perspectives
Stavropoulos et al. (2022)	California, USA	Mixed-methods study	Family’s home	Caregivers: 15	N/A	Synchronous	TAP TAK	Satisfaction
Talbott et al. (2020)	Various States, USA	Feasibility study	Family’s home	Parents: 10	N/A	Synchronous	TEDI protocol	Satisfaction
Talbott et al. (2022a)	Various States, USA	Pilot study	Family’s home	Families: 30	N/A	Synchronous	TEDI protocol	Usefulness, Ease of Use, Effectiveness, Reliability, Satisfaction,
Talbott et al. (2022b)	Various States, USA	Mixed-methods study	Family’s home	Caregivers: 32	N/A	Synchronous	TEDI protocol	Satisfaction Benefits, Challenges, Suggestions
Wagner et al. (2021)	Various States, USA	Mixed-methods study	Family’s home	Clinicians: 9	Licensed clinical psychologists	Synchronous	TAP	Satisfaction, Perceptions, Diagnostic outcomes
Wagner et al. (2022)	USA and other countries	Mixed-methods study	Family’s home	Clinicians: 202	Clinical psychologists: 103 Developmental behavioral pediatricians: 34 Other medical providers: 10 School psychologists: 9 Behavior analysts: 4 Speech-language pathologists:6 Graduate students:7 Other professionals: 29	Synchronous	TAP	Satisfaction, Perceptions, Diagnostic outcome, Diagnostic certainty, Autism severity

*number of participants for whom data are available for any of the outcomes of interest. TAP: TELE-ASD-PEDS, BOSA: Brief Observation of Symptoms of Autism, ADI-R: Autism Diagnostic Interview-Revised, ADOS-2: Autism Diagnostic Observation Schedule-Second Edition, NODA: Naturalistic Observation Diagnostic Assessment, STAT: Screening Tool for Autism in Toddlers & Young Children, TAK: TELE-ASD-KIDS, CARS-2: Childhood Autism Rating Scale-Second Edition, VABS-2: Vineland Adaptive Behavior Scales-Second Edition, KBIT-2: Kaufman Brief Intelligence Test—Second Edition, TEDI: Telehealth Evaluation of Development for Infants, ABAS-3: Adaptive Behavior Assessment System, Third Edition, BASC-3: Behavior Assessment System for Children, Third Edition.

### Quality appraisal

Overall, the quality of the papers ranged from low to very high. Three studies were rated as low quality, as they met one or two criteria. Seven studies were rated as moderate quality, meeting three criteria. Thirteen studies were deemed high quality scoring four out of five on the tool. Finally, four studies met all five criteria of the MMAT and were rated as very high quality. A common limitation identified in the majority of quantitative and mixed-methods studies involved the high probability of nonresponse bias. Detailed data for each study can be found in the S1 Table.

### Data synthesis

**Fig 2:**
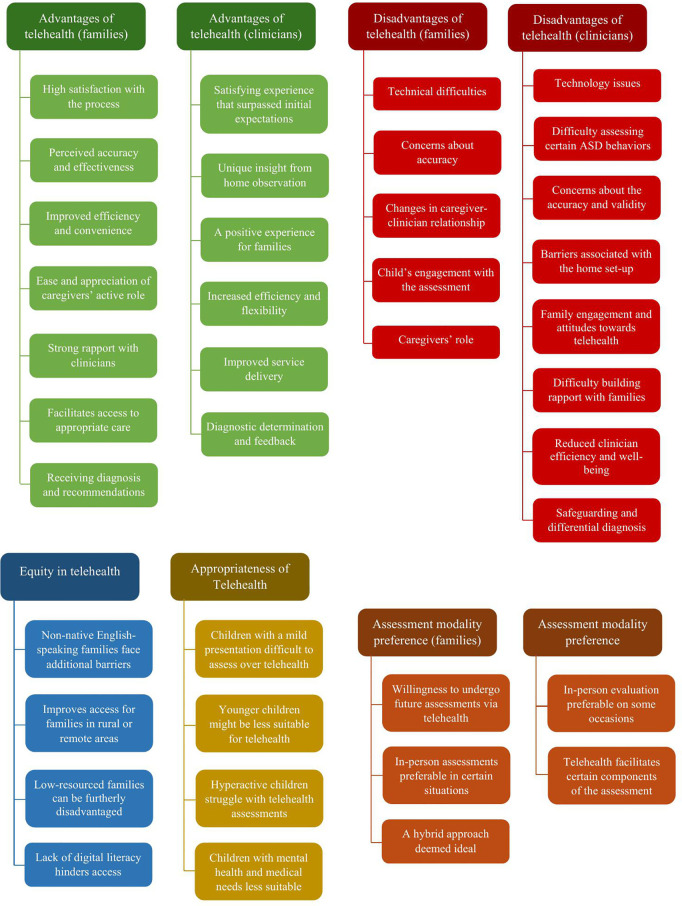
Analytical and descriptive themes.

The thematic synthesis generated the following five analytical themes: 1) advantages of telehealth, 2) disadvantages of telehealth, 3) assessment modality preference, 4) equity in telehealth, and 5) appropriateness of telehealth. An overview of all analytical and descriptive themes generated is presented in Fig 2.

#### Advantages of telehealth – Perceived advantages amongst families.

##### Theme 1. High satisfaction with the process

In multiple studies, caregivers reported high levels of satisfaction with the telehealth procedure [40,61,66–68,70,71,74,77,78]. The quality of the sound and the video was acceptable [63,64,78] and only a minority of families experienced substantial technical difficulties that impeded their participation [40,63,65,77]. For example, in one study 52% of participants reported no disadvantages, and 92% were satisfied with telehealth services [62]. Three studies found no difference in satisfaction scores between telehealth and in-person assessments among families [40,41,68].

Caregivers’ satisfaction with telehealth could depend on several factors. For example, higher satisfaction was reported by caregivers of children with more severe ASD and lower adaptive skills [74]. Families who received a diagnostic outcome via telehealth also demonstrated increased satisfaction [74]. Families’ satisfaction was also higher when clinicians appeared certain about the diagnostic outcome [74]. In two studies, caregivers of female children reported greater satisfaction with the diagnostic procedure compared to those of male children [70,74]. Additionally, caregivers preferring telehealth appointments were more satisfied with the telehealth procedure than those favoring an in-person visit [74]. Finally, the child’s age did not appear to be associated with the parent-rated acceptability [70].

##### Theme 2. Perceived accuracy and effectiveness

Caregivers appreciated having their child observed in a naturalistic environment, enabling clinicians to observe the child’s typical behaviors that might have been obscured in a clinical environment due to the child’s shyness or anxiety [59,65,67,70,78,80]. Considering the above, most caregivers perceived the telehealth assessment as effective and accurate [65,67,70,78–80] and exhibited high agreement with the diagnostic outcomes [70].

*I felt like the assessors really got to know my child and provided a great plan moving forward as well as accurate diagnoses* [65].

Families also praised clinicians’ expertise and competence in assessing autism [40,65,70] and their ability to gather relevant information via telehealth [66,71,74]. They also believed that clinicians could utilize TELE-ASD-PEDS effectively [60,64] and that this tool could successfully elicit behaviors of concern [60,61,64]. Overall, parents appeared satisfied with the clinician’s effort and ability to grasp the child’s presentation and found their questions relevant to understanding the child’s strengths and difficulties [41,60,65]. They were also pleased with their ability to communicate their concerns online [66,71,74] and felt that their concerns were adequately addressed [40]. Only a quarter of the participants believed that either themselves or the person they cared for was not able to convey or perform something online as they would in person [63].

In two studies, parents believed that the results from the telehealth evaluation would not differ from those from an in-person assessment [65,70] and perceived the virtual procedure as equally thorough as traditional assessment [70]. Most parents also reported that the equipment was not distracting and did not diminish the effectiveness of the assessment [66,71].

##### Theme 3. Improved efficiency and convenience

According to parents, telehealth significantly enhanced the efficiency of the diagnostic procedure in several respects [62,67]. Parents benefited from not needing to travel to an in-person health service [59,65,80], thereby reducing or even eliminating travel-related expenses [59,62,67] and saving substantial time [66,71,77]. Many families expressed satisfaction with the brief length of the assessment [60,61,64,78], which averaged between 23 and 39 minutes in TELE-ASD-PEDS studies [60,61].

*I think it was very efficient time wise. Both of us could be there to the hour, there is no travelling time and it was cheaper* [62].

Telehealth also offered flexible medical care [62,67], allowing families to schedule appointments more flexibly, accept appointments timely [67], and incorporate telehealth appointments within their work schedule and attend those from various locations, thus optimizing their routines [62]. Telehealth also enabled multiple stakeholders, including healthcare professionals and additional caregivers or members of the extended family to attend the consultation remotely [62,67].

Families also found telehealth to be comfortable and convenient [59,60,62,64,66,67,70,71,74,77,78]. Caregivers noted that both they and their child felt relaxed in a familiar environment, enhancing the child’s engagement with the assessment [62,63,67]. This was especially advantageous for families of children with sensory difficulties, who could face additional difficulties traveling to and attending the clinic [67,80]. Convenience was particularly valued during the COVID-19 pandemic, as families could attend their appointments from a safe and secure place [63].

##### Theme 4. Ease and appreciation of caregivers’ active role

Most caregivers appreciated the ease of accessing, participating and navigating the virtual assessment procedure [59,77–79]. In one study, all parents found the telehealth process easy, and the majority (80%) did not need to acquire technology-related knowledge prior to the assessment [77]. In studies utilizing the TELE-ASD-PEDS, caregivers demonstrated a good understanding of the telehealth assessment procedure and their role in that [60,61,64] and regarded the instructions as easy to understand and follow [60,61,64,65]. Finally, many parents were able to manage both their child and aspects of the technology during the consultation [65].

*The whole process was incredibly easy… I wish everything was telehealth. Even if it wasn’t the pandemic, with multiple children and going back and forth gets hard. It was the best experience ever* [77].

Caregivers also appreciated their role in the procedure. They felt comfortable playing with the child [60,61,64] and appreciated the parent-led nature of the process, which benefited the child, who felt more comfortable interacting with a familiar figure rather than an unfamiliar clinician [60].

##### Theme 5. Strong rapport with clinicians

Caregivers were satisfied with clinicians’ attitudes and support. Although some families were initially skeptical, many managed to establish a strong rapport with providers [65,67,80] and felt that both they and their children were comfortable interacting with clinicians via telehealth [40,63,64,70].

According to parents, telehealth did not impede clinicians’ abilities to convey compassion, active listening, and other positive attributes [40,65,67,68,80]. Parents valued being included in decisions regarding their child’s care [62,68] and felt their input was considered [41]. Most families believed clinicians used accessible language, which facilitated their engagement [62,68]. They also appreciated clinicians’ efforts to engage effectively with the procedure and the family, feeling that they were integral to the visit [66,71,74]. Overall, families felt well-supported and adequately guided through telehealth [59,78].

Regarding clinician-child interaction, most parents believed that their child was connected to the assessment staff through telehealth similarly to an in-person environment, with parents of female children being more likely to endorse this view [70]. Most parents also believed clinicians treated their children with respect [62], demonstrated concern for their privacy, and showed sensitivity towards their needs [68].

##### Theme 6. Facilitates access to appropriate care

Many families reported that telehealth allowed them to access timely care and have their needs met [65,67]. Telehealth was viewed as an optimal solution during COVID-19, enabling children to attend appointments despite physical restrictions [62,63] and reducing the risk of infection and safety-related concerns and anxiety [65,67].

*I think I would recommend telehealth assessments during a COVID-19 situation, definitely. It’s better to have the assessment by telehealth rather than not have it* [63].

A timely assessment through telehealth also facilitated parents’ access to early intervention services and appropriate support [62,65,66,74]. Furthermore, telehealth enabled parents to attend multiple medical appointments, allowing concerns to be addressed more efficiently, especially for those requiring regular services [67]. Most families preferred to undergo an assessment virtually rather than wait for an in-person assessment [70].

##### Theme 7. Receiving diagnosis and recommendations

Families valued receiving a diagnostic outcome and recommendations for their child via telehealth [62,67,77], with most feeling comfortable discussing the diagnosis with clinicians after the assessment [60,64]. They were satisfied with the clinician’s ability to explain the diagnosis and information provided and they found the procedure helpful in understanding the condition [41,65,68]. Most parents felt that their concerns were adequately addressed [65,70,77], and the appointment length allowed sufficient time for their questions [65]. Parents also found the recommendations and the recourses provided useful and insightful [41,62,64,65], helping them make decisions about the next steps [66,70,71]. By the end of the feedback session, most parents had a solid understanding of the next steps for their child [65,77].

*My questions and concerns where very well explained and answered during virtual video assessment and I’m sure it would have been the same as in person assessment* [65].

Given the nature of obtaining a lifelong diagnosis, parents valued having the time and space to process the diagnostic outcome [67], with reactions varying based on emotional readiness; those anticipating the diagnosis were more accepting, while others were more likely to question the telehealth process [67].

#### Advantages of telehealth – Perceived advantages amongst clinicians.

##### Theme 1. Satisfying experience that surpassed initial expectations

Most clinicians reported high levels of satisfaction with telehealth assessments [66,67,71,74] and tools such as TELE-ASD-PEDS [81]. Clinicians found the procedure enjoyable and were pleasantly surprised by the similarity between telehealth and traditional, in-person assessments [69]. Some clinicians also noted that the sudden shift to telehealth sparked research into the development of novel tools to address the limitations of traditional tools [76].

*But clinically, I’ve been really pleasantly surprised, and I found it rewarding and enjoyable. And it’s been a good challenge. It’s kept us all on our toes and had the opportunity now to train other people in virtual assessments, interns and people in supervised practice. And I’ve enjoyed that. I’ve enjoyed working with them* [69].

Provider satisfaction depended on their attitudes towards telehealth and adaptability. Those comfortable acquiring knowledge and skills about technology and who found novel challenges rewarding and enjoyable could adapt swiftly [69]. Access to appropriate technological infrastructure and support from colleagues and leadership was also helpful [69]. Finally, clinicians who were able to reach a diagnostic conclusion, particularly of ASD, and those with higher diagnostic certainty reported greater satisfaction [74].

##### Theme 2. Unique insight from home observation

A frequently cited benefit of telehealth was the opportunity to gain valuable insights from observing children in their home environment [69,81,82]. Assessing children in a naturalistic setting allowed clinicians to identify and evaluate typical behaviors that may not manifest in a clinical context [67,76], especially among overly anxious or behaviorally inhibited children [69], thereby enhancing the accuracy and validity of the procedure [69,76].

*And I actually think, with some of the little kids, we’re getting a better picture than we would have gotten when they come into clinic* […] [69].

Moreover, at home, children could play using their own toys and interact with familiar family members [69], providing unique insights into their special interests and preoccupations that could have been missed by structured tools such as ADOS [76]. Clinicians valued the opportunity to observe the family dynamics [69,76] and the space in which the child lives, which helped them understand the broader social determinants of health for the family [69]. This could enable clinicians to obtain a more spherical insight into the child’s presentation, foster empathy towards the parents, and provide more individualized treatment recommendations [69].

##### Theme 3. A positive experience for families

Clinicians highlighted the benefits telehealth provides families. Though initially skeptical, families appeared highly satisfied with the procedure [69]. They felt more at ease and less anxious joining their appointment from home [63,69,82], especially those who would typically experience distress associated with managing children’s behavioral difficulties while traveling to a medical service [69]. Telehealth was also seen as efficient for families, eliminating their need to travel [62,69] and associated costs [62,69]. Caregivers with limited availability could also join parts of the assessment flexibly, from various locations (e.g., their workplace) [62].

*It’s hard to transport these kids to appointments, take time off work, do different things. And so then being able to access us and these appointments easier has actually been a really good thing for families* [69].

The telehealth assessment also helped families understand the assessment procedure [67,81], see the child’s behaviors from another perspective and understand their difficulties, and ultimately accept the diagnosis [67]. Clinicians noted that parents’ emotional preparedness influenced their acceptance of the diagnosis [67]. The improved insight into the child’s difficulties and needs was likely to enhance families’ engagement with future interventions [67,69].

##### Theme 4. Increased efficiency and flexibility

Several clinicians believed that telehealth could improve clinical efficiency and flexibility [67,81]. Although at the beginning of the COVID-19 pandemic, the use of telehealth was seen as laborious and less efficient, partly due to clinicians’ unfamiliarity with the technology and lack of infrastructure, clinicians gradually grew to view telehealth more favorably and appreciate its efficiency over time [69].

Efficiency improvements included shorter interview lengths, reduced time for documentation and securing physical spaces, and optimized scheduling [69]. Fewer issues related to the clinical flow and more efficient triaging also contributed to improved service delivery [67]. Scheduling appointments was also easier and quicker for clinicians, offering more flexible and accessible appointments for parents [62,67,81]. Some clinicians even noted environmental benefits, such as a lower carbon footprint from reduced traveling [76].

Conducting assessments from home also increased clinicians’ convenience, flexibility, and personal efficiency [76,81,82]. Telehealth eliminated commutes, lowering travel expenses [76] and allowing clinicians to spend more time preparing for visits, creating flexible schedules, and balancing work with personal responsibilities, including childcare [69]. Clinicians could also continue work from home even when experiencing minor physical symptoms and were required to isolate [62].

[…] *In terms of work efficiency, I don’t have to spend that time commuting. I can work on patient paperwork. I can prepare. I have more time to prepare for my visit. So I really like that* [69].

##### Theme 5. Improved service delivery

Clinicians reported several improvements in service delivery. Telehealth enabled multiple stakeholders, including split families, extended family members, and other healthcare professionals to join the session from different locations and offer their insights on the child’s development and presentation [62,67,69,82]. The introduction of telehealth also ensured service continuity during COVID-19 [81,82], mitigating concerns about infection and safety [67].

*The other thing that I think that I think it works really well for are split families. I got a chance to meet so many dads that I’d never seen, grandmas, grandpas, teachers, parents showed up. I was like, “This is awesome.”* [69].

Adopting hybrid or fully virtual models of care also lowered the no-show rates [69,76] as clinicians could easily remind patients of their appointments and the period lapsed between booking the appointment and the appointment date was smaller [69]. Telehealth also facilitated families’ access, by allowing more flexible and accessible appointments [76]. Incorporating telehealth into usual care also enabled services to employ staff living outside of the area, thus surpassing distance barriers [76].

The successful utilization of telehealth depended on the perceived institutional and leadership support, the existence of clear guidelines and the degree to which clinicians’ clinical needs were met [69]. Technological infrastructure and administrative support were also deemed crucial by clinicians [69].

##### Theme 6. Diagnostic determination and feedback

Multiple clinicians believed that diagnosing children via telehealth is appropriate [58,82]. Most were satisfied with the information obtained [71,74], which they considered as useful as the information collected in in-person evaluations [70]. The information was also adequate to make an accurate diagnostic decision [69,70,76,81,82], and clinicians were comfortable addressing caregivers’ concerns and the referral questions [71] and providing a diagnosis virtually [67,81,82]. In two studies, clinicians could make a diagnostic decision in approximately 80–95% of the telehealth assessments [69,72].

Several factors could increase clinicians’ diagnostic confidence, including the availability of information from other sources (e.g., school reports) [69], prior experience assessing children [76], and the ability to observe the child on camera [67]. Furthermore, problem-solving as part of the team, observing other colleagues conducting virtual assessments and attending relevant training could boost clinicians’ confidence [69]. However, families’ skepticism towards receiving a diagnosis via telehealth could undermine clinician confidence [69]. The child’s demographics, adaptive functioning, internalising and externalising symptoms, the presence of an interpreter [73], and technology barriers were not associated with the clinician’s diagnostic certainty [71]. Clinicians noted that their assessment skills were improved as they became more familiar with delivering care over telehealth [62].

Furthermore, clinicians felt comfortable addressing parents’ concerns [70], discussing a diagnosis and providing recommendations to families through telehealth [81,82]. Many clinicians also mentioned that delivering a diagnosis via telehealth was no different than in person [75], with some families struggling to process the diagnosis emotionally, regardless of the mode of delivery [67]. Table 3 provides a summary of the descriptive themes reflecting the perceived advantages of telehealth among families and clinicians.

**Table 3:** Summary of themes related to the advantages of telehealth assessments for autism.

Stakeholder Group	Descriptive Theme	Summary of Findings
Families	High satisfaction with the process	Most caregivers were highly satisfied with telehealth assessments. Satisfaction was higher among caregivers who received a diagnosis, felt the clinician was confident, or preferred virtual modalities.
	Perceived accuracy and effectiveness	Many families believed the virtual assessment elicited the child’s typical behaviors and allowed for a comprehensive evaluation. They found the procedure comparable in quality to in-person assessments and valued clinicians’ expertise.
	Improved efficiency and convenience	Telehealth reduced travel time and costs, minimized disruption to routines, and enabled more flexible appointment scheduling. This option was also regarded as convenient, eliminating distress and discomfort associated with travel.
	Ease and appreciation of caregivers’ active role	Caregivers found the process easy to navigate without needing prior tech knowledge. Many appreciated and enjoyed their active role in supporting the assessment and facilitating child observation.
	Strong rapport with clinicians	Despite initial skepticism, many families reported building strong rapport with clinicians over telehealth. Clinicians’ empathy, compassion, and inclusion efforts contributed to families feeling supported and understood.
	Facilitates access to appropriate care	Telehealth enabled families to access timely diagnostic assessments and early interventions that would otherwise have been delayed due to long waiting lists.
	Receiving diagnosis and recommendations	Families were comfortable receiving diagnostic outcomes and feedback via telehealth. They found clinicians’ explanations clear and appreciated the practical guidance and resources provided.
Clinicians	Satisfying experience that surpassed initial expectations	Clinicians described the telehealth experience as unexpectedly positive and similar to in-person assessments. Their satisfaction was influenced by their adaptability, access to technological and institutional support, and diagnostic confidence.
	Unique insight from home observation	Telehealth allowed clinicians to observe children in their home environment, offering insight into their typical behaviors, family dynamics, and contextual factors not often evident in clinical settings. Some clinicians felt this enhanced assessment accuracy.
	A positive experience for families	Clinicians noted benefits of telehealth for caregivers, including convenience, better understanding of the assessment process, reduced costs and travel time, and greater flexibility.
	Increased efficiency and flexibility	Telehealth enhanced both clinical and personal efficiency for clinicians by optimizing scheduling, reducing documentation and the need to secure physical space, lowering travel costs, and improving work-life balance.
	Improved service delivery	Telehealth facilitated participation of multiple stakeholders (e.g., MDT members, teachers, extended family), reduced no-show rates, and expanded access to under-served populations.
	Diagnostic determination and feedback	Many clinicians considered the information obtained via telehealth adequate to make a diagnosis confidently and were comfortable delivering diagnostic feedback and recommendations.

#### Disadvantages of telehealth – Perceived disadvantages amongst families.

##### Theme 1. Technical difficulties

Families reported technical issues with the equipment, connectivity, or software used to access telehealth [59,64,65]. Some encountered difficulties seeing or hearing the assessor through their device [60,62,64], while others focused on issues around poor connectivity or drop-outs, worsening their experiences with telehealth [62,64]. Several families experienced device-related complications during assessments. For instance, some children became preoccupied with the screen or the camera, while others reported that the small screen hindered their engagement with the procedure [60]. Concerns were also raised about the camera’s inability to follow the children as they were moving [80]. Issues with the software posed further obstacles to the assessment process [59,62]. Importantly, technical difficulties were associated with reduced satisfaction with the telehealth visit [74].

*The video kept cutting in and out making it hard to hear and understand what was being said* [60].

##### Theme 2. Concerns about accuracy

Some parents expressed reservations about telehealth’s diagnostic accuracy for autism, suggesting it might yield less reliable results than in-person evaluations [65,67]. A primary concern involved the telehealth procedure’s perceived inability to elicit the behaviors of concern [60,64]. Some caregivers suggested that subtle behaviors were difficult for the clinician to detect virtually [63], with a significant number of families agreeing that there were important things they or their child could not do or say online [63,64]. Additionally, some parents viewed the virtual assessment as overly brief and less comprehensive compared to an in-person evaluation [60,64], while others questioned clinicians’ ability to diagnose their child without a physical assessment [62,67]. Several parents argued that clinical settings, unlike home environments, would naturally trigger relevant behaviors, thus providing more reliable diagnostic insights [77]. Notably, in one study, fewer than half of the participants (45%) believed that the telehealth assessment was as effective as in-person assessments [65].

*Especially with assessments, it is more effective in person because you can interact with the child and see the child in a different environment, which is needed with a child with different behaviors* [77].

##### Theme 3. Changes in caregiver-clinician relationship

Some caregivers mentioned that telehealth introduced changes in their relationship with practitioners [67]. The telehealth assessment process was often perceived as less personal compared to in-person evaluations [60]. Barriers to building rapport included technical issues [67] and the unavailability of the visual element (i.e., absence of video), which impeded families’ ability to engage emotionally during the assessment [67].

##### Theme 4. Child’s engagement with the assessment

Families voiced concerns about children’s engagement during telehealth sessions [62]. Some parents described their child’s difficulty remaining still near the screen, which rendered the observation challenging [77]. In a study involving infants, several caregivers believed that their child could not fully engage with the virtual session and that in-person assessments would better facilitate their engagement [80]. Some caregivers also mentioned that the child appeared less interactive with the clinician over telehealth [67].

*It was somewhat challenging trying to engage my child in the activities in the same fashion as a clinician would (given their unique skillset and my lack of training in this area)* […] [80].

##### Theme 5. Caregivers’ role

Although caregivers appreciated their active involvement in telehealth assessments, many were unclear about their role in the assessment and the degree to which they had to interact with the child [60,77]. Some families also found setting up the assessment and being responsible for directing the interactions challenging, with communication proving more difficult over telehealth than in person [80]. Certain parents noted the emotional aspect of witnessing their child’s challenges in real-time, as these became evident during assessment activities, which they found difficult to process [80].

*I enjoyed being a part of the process but worried my interaction was too much or too little* [60].

#### Disadvantages of telehealth – Perceived disadvantages amongst clinicians.

##### Theme 1. Technology issues

Clinicians described technology-related difficulties encountered by both themselves and the families. Bain et al [58] observed that nearly 90% of clinicians experienced difficulties accessing or navigating telehealth sessions. However, other studies indicated that only a minority of clinicians faced similar issues [62,71,72]. Technical difficulties encompassed poor internet connection, dropped calls [62,81], issues with audio and video quality [66,81], and platform glitches and usability problems [62,67]. Families also experienced technical issues [67], including inadequate internet connection [62,71]. In one study, clinicians had to assist caregivers with accessing the telehealth platform in almost 20% of evaluations [71]. Issues with camera positioning were also prevalent [76,81].

*Often families were in poor internet connection areas. Our internet connection was quite poor as well* [62].

Families encountering significant technical difficulties had to either reschedule their appointment or book a follow-up appointment [67]. Technology-related issues limited clinicians’ ability to conduct thorough clinical observations [81] and diminished their satisfaction with the assessment process [74]. Yet, several studies found that technological challenges disrupted clinicians’ ability to conduct assessments and gather useful information only in a minority of cases [66,71,72].

##### Theme 2. Difficulty assessing certain ASD behaviors

Clinicians found particular ASD features and behaviors challenging to assess via telehealth [63]. The assessment of eye contact was considered challenging [69,76,81], as children usually looked at the screen rather than the camera, and eye contact with family members was not easily identifiable [69]. Evaluating other non-verbal behaviors, such as facial expressions and gesturing, was also difficult [76], requiring clinicians to rely on caregivers’ reports, which the former found challenging [81].

*I think the hardest thing was the eye contact because I guess the client is not really engaging in eye contact at all. They’re looking at you on the screen, not in the camera. So that was challenging* [63].

Other ASD-related behaviors, such as sensory differences, which could not be easily elicited in the home environment [69,76], behaviors associated with social interaction and social reciprocity [69,70,76], restrictive and repetitive behaviors [70,76], and joint attention [63] were also deemed difficult to assess via telehealth by some clinicians. Other clinicians also had a hard time getting a sense of the child’s gait and posture via telehealth [76]. Finally, many clinicians struggled to evaluate behaviors that were subtle, yet crucial for establishing or ruling out a diagnosis [63,76].

##### Theme 3. Concerns about the accuracy and validity

Some clinicians questioned the validity of telehealth for diagnosing ASD, given the inability to conduct a physical examination, environmental distractions, difficulty observing certain behaviors and traits, and technical issues [67,76]. An additional concern was the lack of standardization of novel telehealth diagnostic tools (e.g., TELE-ASD-PEDS) and the difficulty adapting existing tools, such as the ADOS-2, for virtual administration [69,75,76]. The reliance on family reports over direct observation also reduced clinicians’ diagnostic confidence [63,75,76]. The diagnostic decision-making process was more difficult for complex cases and for less experienced health professionals [76]. Spain et al found that 77% of clinicians were unable to make a diagnostic decision in some cases via telehealth [75], while Bain et al reported that only 40% of practitioners believed ASD could be diagnosed in a first telehealth encounter [58].

*...[I] feel so strongly about it that it’s not valid...if the full assessment is done remotely, it’s not clinically valid I couldn’t in all conscience assign a diagnosis [about] something as profound as how you interact socially with another human being having never sat in a room with them* [76].

Some clinicians were likely to recommend in-person re-evaluations for children assessed remotely [69], while others refrained from providing official diagnoses to children they had not evaluated in person. Some services provided the opportunity for children to receive a future face-to-face assessment [76] or offered ASD-related recommendations or formulation-led feedback even without establishing an official diagnosis [75,76]. Nevertheless, some clinicians noted that complex cases remained challenging regardless of the assessment modality [67,69].

##### Theme 4. Barriers associated with the home set-up

Family and home-related barriers were also cited [71]. A prominent limitation involved environmental distractions, such as poor lighting, background noise, or other stimuli that distracted the child and hindered clinicians’ ability to carry out the assessment and obtain an accurate picture of the child’s presentation [62,76,82]. Privacy was sometimes lacking due to the presence of other children or family members, which introduced further complications [62,76,82]. Some families had difficulty securing a confidential space [69] and attended the assessment from unsuitable locations, such as workplaces [76]. The lack of necessary materials, such as specific play items for TELE-ASD-PEDS, could also impede the assessment’s feasibility [73].

*Parents were often at home stuck with kids not at school so there were a lot of other distractions going on at home* [62].

Importantly, one study found that home-related barriers disrupted the assessment in 19% of the evaluations but were not associated with clinicians’ ability to provide a diagnosis or with their diagnostic certainty [71]. Another study noted that while such barriers were present in 20% of cases, they disrupted clinicians’ ability to gather essential clinical information in 44% of these evaluations [72].

##### Theme 5. Family engagement and attitudes towards telehealth

Caregivers’ participation and attitudes towards telehealth influenced clinicians’ experiences. According to clinicians, caregivers displayed varied levels of understanding of the instructions provided [81], with some demonstrating limited adherence [63,81]. Clinicians viewed having parents elicit behaviors as inherently limiting, rendering the interpretation of the child’s behaviors difficult [67].

*I think one of the challenges was having to remind parents not to coach the child…. sometimes parents would do things like directing the child more than you would like in an observational assessment* [63].

Clinicians also commented on the families’ organization and preparation for the assessment. While several families were well-organized and proactive, others were unprepared, making the assessment more challenging [63]. Families’ stance towards the assessment introduced additional challenges, with a few parents “not taking the process seriously” [69] or exhibiting inappropriate behaviors [76].

Some families declined telehealth evaluations, even after receiving information about the procedure, preferring to wait for an in-person assessment. Although no clear pattern emerged among these families, most cited doubts about telehealth’s accuracy [69]. Clinicians observed that some parents viewed telehealth assessments as less effective than in-person evaluations [58], with others expressing apprehension or uncertainty about the process [81].

##### Theme 6. Difficulty building rapport with families

Overall, while some clinicians could connect with families virtually [67,69], others found establishing rapport via telehealth challenging [62,67,69,75,76], though the difficulties were often less pronounced than anticipated [69]. Clinicians considered getting to know the child more challenging via telehealth, requiring more time to build rapport and trust [62].

*I think we underestimate how much or how important that [rapport] is in building a relationship with our families and you know some of the gestures we use or the support we can offer them with a box of tissues or a warm word or stroke their arm or something. We can’t do any of that to support them or even asking them questions and then becoming distressed so I miss that physical contact* [62].

Many factors complicated rapport-building, including difficulties reading the child’s and caregivers’ body language and identifying nuanced behaviors [62]. Clinicians found exhibiting empathy and understanding interpersonal cues more challenging virtually rather than in person [67]. Audioconferencing also hindered emotional engagement compared to videoconferencing [67,68]. Finally, technical issues further hindered the development of rapport [67].

While rapport-building with caregivers was relatively straightforward, connecting with children varied [69]. Clinicians struggled to connect with children not familiar with interacting via a screen [75], and distractions at home further disrupted rapport-building [67]. Consequently, some clinicians were hesitant to deliver a “life-changing” diagnosis via telehealth [75,76].

##### Theme 7. Reduced clinician efficiency and well-being

Another disadvantage vocalised by clinicians concerned the reduced clinical efficiency associated with telehealth. For instance, administrative staff acquired increased workloads [62,69], and clinicians found workflows less efficient, requiring more time to prepare for and conduct assessments and to complete relevant documentation, with many also taking on additional administrative tasks [69]. Clinicians described working laboriously to meet clinical demands with little time to adapt to telehealth [69]. They also narrated how telehealth negatively affected their work-life balance, particularly for those managing multiple responsibilities (e.g., childcare) [69,76].

The transition to telehealth led to elevated work-related stress, especially early in the COVID-19 pandemic, stemming from the general uncertainty, unfamiliarity with telehealth, fatigue and disconnection from colleagues [69,76]. Many clinicians missed in-person interactions with colleagues and experienced increased isolation, which could negatively affect team cohesiveness and dynamics [69,76]. Some faced mental health difficulties from lone working, while others reported physical health issues, such as tiredness and eyestrain from prolonged screen exposure [76].

*I’ve never met my team. I’ve never met my supervisor. I’ve never met my patients in person...also it felt very isolated with the team and definitely didn’t help with some team dynamics...sometimes it’s nice to knock on somebody’s door and asking the question, or at least meet the people we work with* [76].

##### Theme 8. Safeguarding and differential diagnosis

Clinicians shared their experiences administering standardized tests online to assess domains such as cognitive abilities, developmental level and speech and language [69]. While some gathered adequate information and found certain tests (e.g., KBIT-2 or Vineland) accurate [70], many encountered challenges with online administration [69,70,76,82]. For instance, managing children’s behavior and maintaining their attention was often difficult [69]. Technical problems further complicated standardized testing, while subtle behaviors were also hard to observe [70]. Finally, several clinicians regarded the inability to perform a physical examination as a significant drawback [76,82].

Another major limitation of telehealth was the difficulty conducting thorough risk assessments and addressing safeguarding issues during virtual visits [69,75,76]. Clinicians faced challenges identifying potential abuse, neglect, or other safeguarding concerns [75,76]. Some noted the complexity of managing safeguarding issues remotely [69,76] without being in a clinical environment, where they could consult colleagues, and due to the presence of multiple members in the child’s environment during the assessment, limiting the information clinicians could gather [76]. Clinicians were also concerned about not always being aware of the client’s location during the assessment [76]. Given these challenges, clinicians highlighted the need for “safeguarding supervision” to help them navigate complex cases and ethical issues [76].

*We haven’t seen a huge increase in physical abuse, but we also aren’t seeing these kids in-person so we can’t be totally certain…So that’s been something we’ve talked about as a division that we’re at least somewhat worried about but don’t know what to do about it* [69].

A summary of the descriptive themes capturing the perceived disadvantages of telehealth among families and clinicians is presented in Table 4.

**Table 4:** Summary of themes related to the disadvantages of telehealth assessments for autism.

Stakeholder Group	Descriptive Theme	Summary of Findings
**Families**	Technical difficulties	Many families reported experiencing technical issues with the equipment, connectivity, and software, which had a negative impact on their satisfaction.
	Concerns about accuracy	Some parents expressed concerns about telehealth’s ability to elicit their child’s behaviors of concern and capture subtle difficulties or differences. As such, some perceived virtual assessments as less accurate than in-person evaluations.
	Changes in caregiver-clinician relationship	The patient-provider relationship was perceived by some parents as less personal, with technical difficulties hindering rapport building.
	Child’s engagement with the assessment	Some children struggled to remain still near the screen and engage with the appointment, a difficulty that might had been mitigated in a clinical context.
	Caregivers’ role	While many appreciated their involvement, some caregivers felt uncertain about their role in the assessment or found aspects of their facilitation emotionally or logistically challenging.
Clinicians	Technology issues	Some clinicians and families experienced technical challenges, such as poor internet connectivity, audio/video glitches, and platform usability issues.
	Difficulty assessing certain ASD behaviors	Certain behaviors and traits, such as eye contact, other non-verbal behaviors, and subtle traits were difficult to accurately assess remotely.
	Concerns about the accuracy and validity	Some clinicians questioned the accuracy of remote assessments, especially in complex cases. The lack of standardization of telehealth tools and reliance on caregiver reports further reduced diagnostic confidence.
	Barriers associated with the home set-up	Environmental distractions, lack of privacy, attendance from unsuitable locations, and absence of necessary materials challenged the feasibility and accuracy of the assessment.
	Family engagement and attitudes towards telehealth	Variability in caregiver preparation, understanding of instructions, and attitudes toward telehealth affected assessment quality. Some families showed low engagement or skepticism about the process.
	Difficulty building rapport with families	Establishing rapport, particularly with children, was more difficult via telehealth. Demonstrating empathy and understanding interpersonal cues remotely was also considered challenging.
	Reduced clinician efficiency and well-being	Telehealth introduced new administrative burdens, disrupted workflows, and, for some, worsened work-life balance. Clinicians also reported isolation, fatigue, and mental health strain associated with remote work.
	Safeguarding and differential diagnosis	Clinicians raised concerns about the challenges of conducting risk assessments and physical examinations via telehealth. Safeguarding issues were harder to detect, and ethical dilemmas were more difficult to manage.

#### Assessment modality preference – families.

##### Theme 1. Willingness to undergo future assessments via telehealth

In numerous studies, clinicians conveyed a strong willingness to participate in future diagnostic assessments for their child via telehealth [62–64,66,70,71,74] and would recommend the telehealth procedure to other families [61,62,66,71,74,78]. While in some studies, views were polarized [67,78], in others, families expressed a preference to attend appointments via telehealth [70], especially for follow-up appointments, appointments for a clinical interview or feedback, and appointments not necessitating the child’s presence [62]. Parents also favored telehealth for receiving “good news” (no diagnosis) about their child’s outcome [62]. Finally, most parents described being comfortable accessing virtually any appointments clinicians deem unnecessary to attend in person [62].

##### Theme 2. In-person assessments preferable in certain situations

Despite recognizing the benefits of telehealth, many families preferred for their child to undergo a comprehensive assessment in a clinical, in-person setting [62,64,65,78]. They felt in-person interactions would be more conducive to their child’s engagement [62,65] and provide a more accurate picture of their difficulties [65]. Some families also recognized the benefits of seeing their child’s interaction with a clinician in person [60].

*I like the telemedicine as it included me as a parent but I also like the full evaluation as it allows me to see what happens when someone else is working with my child* [60].

On certain occasions, in-person appointments were preferable. For example, only a minority of families would choose telehealth for an appointment with a new clinician [62]. Additionally, most parents would prefer receiving an ASD diagnosis in person, with only a small fraction (24%) comfortable with a physical assessment via telehealth, even if it could be completed over video [62].

##### Theme 3. A hybrid approach deemed ideal

While some families expressed a preference for either telehealth or in-person visits, many favored a hybrid model, wherein telehealth would complement in-person evaluation [60,62]. One study found that nearly half of families preferred a combination of virtual and in-person appointments, with the type of appointment depending on the context of the consultation, availability, and the stakeholders involved [62]. Some parents advocated using telehealth as an initial step in the diagnostic pathway, followed by an in-person consultation [60].

#### Assessment modality preference – clinicians.

##### Theme 1. In-person evaluation preferable on some occasions

Studies explored clinicians’ preferences regarding in-person versus telehealth assessments. In one study, clinicians indicated a preference to see the child in person in just over half of the evaluations [71], whereas Juárez et al found this preference in just 24% of the cases [66]. Clinicians favored in-person assessments for appointments involving testing, developmental assessment, or a physical examination [67]. In-person evaluations were also preferred for children with developmental delays, complex presentations and history, or when technical problems affected the virtual appointment [66]. Clinicians noted that in-person assessments were often necessitated for children with less clear presentations or in cases where a comprehensive observation was not feasible via telehealth [69].

##### Theme 2. Telehealth facilitates certain components of the assessment

Clinicians appreciated the integration of telehealth into their practices, and despite some variations across studies, most were keen to use this modality for future consultations [58,67,70] even when in-person assessments were to be readily accessible [70]. They favored telehealth over in-person evaluations for follow-up appointments, feedback sessions, and brief appointments not involving the child [67]. Furthermore, most clinicians considered obtaining developmental history easier via telehealth and preferred this modality for history-taking [67,76].

Many clinicians favored a hybrid assessment model, allowing them to provide flexible services tailored to families’ needs [75,76]. Some believed an initial telehealth consultation could improve the diagnostic assessment process by enabling clinicians to collect initial information and obtain insight into the child’s presentation in advance of the in-person evaluation [62]. However, some clinicians were concerned that telehealth might become a ”panacea”, offered to families who may benefit more from a comprehensive, in-person evaluation [75].

*[I am] happy to advocate a hybrid model, as long as the hybrid model is being hybrid to increase capacity without losing quality* [76].

Table 5 presents a summary of the descriptive themes related to families’ and clinicians’ preferences regarding autism assessment modality.

**Table 5:** Summary of themes related to assessment modality preference in telehealth autism assessments.

Stakeholder Group	Descriptive Theme	Summary of Findings
Families	Willingness to undergo future assessments via telehealth	Many families appeared willing to use telehealth again, particularly for follow-up appointments or sessions not necessitating the child’s attendance.
	In-person assessments preferable in certain situations	Certain families preferred in-person evaluations, especially for appointments with unfamiliar clinicians or when a diagnosis of ASD would be delivered.
	A hybrid approach deemed ideal	Numerous caregivers expressed support for a hybrid model, where remote and in-person assessment elements could complement each other.
Clinicians	In-person evaluation preferable on some occasions	Clinicians preferred in-person evaluations for complex cases, those needing extensive testing and examination, or when technical difficulties impeded the observation.
	Telehealth facilitates certain components of the assessment	Clinicians preferred telehealth for follow-up appointments, history-taking, feedback sessions, and appointments not involving the child, with some favoring a hybrid model.

#### Equity.

##### Theme 1. Non-native English-speaking families face additional barriers

In English-speaking countries, particularly the United States, Canada, and Australia, certain barriers to telehealth were amplified for families with limited English proficiency [62,67,69]. A prevalent challenge involved integrating interpreters in telehealth assessments, which could reduce the quality of the latter [67]. Clinicians observed a decrease in non-native English-speaking families accessing services during COVID-19, with many opting to wait for in-person appointments [69]. Therefore, providers were concerned that these families were disproportionately affected by the transition to telehealth [69]. Several ethnically and linguistically diverse families also favored in-person assessments, where they would feel more comfortable communicating their concerns [62].

*Overall it was positive but I have a little bit language barrier because I am not an English native speaker so I prefer face-to-face* [62]. (caregiver quote)

Non-Native English-speaking families faced challenges accessing and navigating telehealth platforms, which were primarily in English with no available translations [67]. Clinicians also noted that “the amount of bureaucracy” associated with online assessments (e.g., signing forms) could further deter these families from utilizing telehealth [67]. Some clinicians speculated that non-English-speaking families experience additional hardships that render their participation in virtual assessments challenging [69].

*The non-English-speaking families had a little bit more difficulty. And I think they were also the ones that were less comfortable doing virtual visits. Generally speaking, they’re the ones who have wanted to come in in person. They’ve also been the ones that have been more hard hit with COVID* [69]. (clinician quote)

Challenges also arose when guiding families through interactive play-based activities via an interpreter, especially when the latter joined without video [69]. Some clinicians were also concerned about potential cultural inappropriateness of certain questions and found it more difficult to develop a rapport and conduct an accurate observation with non-English speaking families, questioning their reliability for this population [67]. Corroborating the above, in one study, although 92.3% of clinicians felt comfortable diagnosing ASD in English-speaking children, only 61.6% felt comfortable diagnosing Spanish-speaking children virtually [67].

##### Theme 2. Improves access for families in rural or remote areas

There was a consensus amongst clinicians that telehealth assessments benefited rural or remote populations [62,63,67,69]. Clinicians reported that families residing far from metropolitan centers found telehealth convenient, as it eliminated the need to travel [63] and reduced travel-related expenses [67,69]. Additionally, families experienced less anxiety and stress associated with the child’s discomfort and dysregulation from traveling to services [63,67]. Clinicians concluded that telehealth could expand access for geographically distant families who might otherwise be prevented from attending in-person appointments, ultimately reducing healthcare disparities [67,69]. Families also appreciated the opportunity to attend appointments via telehealth, which might otherwise be inaccessible due to their remote location [62,80].

*I love that it’s Telehealth. We wouldn’t be able to participate in the study otherwise, since we don’t live nearby* [80]. (caregiver quote)

##### Theme 3. Low-resourced families can be further disadvantaged

Socioeconomically deprived families were particularly disadvantaged in accessing telehealth for their child’s assessment. In contrast, families with higher socioeconomic status possessed greater access to and understanding of telehealth and were able to navigate it more easily [69].

For low-resourced families, lack of access to adequate technology was a common barrier [69]. Although most families possessed technological devices (e.g., smartphones) and high-speed connection [62,69], some lacked sufficient technological hardware or strong internet connection, hindering their ability to use telehealth effectively [58,59,62,67,69]. The unavailability of play materials required in some assessment procedures (e.g., TELE-ASD-PEDS) posed an additional barrier [81]. Additional challenges included housing insecurity, lack of private space, and the presence of multiple children in the home [67,69,81].

*I think the disparities are there are patients and families that don’t have the resource to have the devices that they need and the Wi-Fi and lots of technology pieces that were happening* [69]. (clinician quote)

Clinicians raised ethical concerns about disparities telehealth might introduce, noting that families who were unable to use telehealth could face longer waiting times, thus widening the care gap [76]. Consequently, many clinicians concluded that telehealth was unlikely to facilitate access to care for the entire population [67]. Some clinicians, however, were uncertain about the extent of these disparities due to limited data on families who did not participate in telehealth assessments, while others felt that families struggling with in-person assessments might also find telehealth challenging [67].

##### Theme 4. Lack of digital literacy hinders access

Digital literacy among parents and clinicians could exacerbate health disparities. Clinicians mentioned that, although only a minority, some families were less comfortable using technology and telehealth platforms [69,76], requiring an in-person evaluation instead [67,69]. Some families found the process intimidating, even when clinicians made efforts to facilitate the technical side [67].

*But I would say that would be the other big barrier is that there were a small, but certainly significant group of people that either couldn’t use the technology or the technology wasn’t available* [69]. (clinician quote)

In studies exploring families’ experiences, some reported limited familiarity with technology and described themselves as less tech-savvy [62,67]. Importantly, those who encountered technological barriers believed that these challenges reduced the quality of their telehealth visits [67]. A summary of the themes pertaining to equity considerations in telehealth assessments for autism is provided in Table 6.

**Table 6:** Summary of themes related to equity in telehealth autism assessments.

Stakeholder Group	Descriptive Theme	Summary of Findings
Families and Clinicians	Non-native English-speaking families face additional barriers	Families with limited English proficiency faced additional challenges accessing telehealth, including difficulty navigating English-only platforms, while the integration of interpreters was also considered challenging by clinicians.
Families and Clinicians	Improves access for families in rural or remote areas	Telehealth improved access for families living in rural or remote areas by eliminating travel requirements, reducing associated costs and stress, and ultimately facilitating attendance and reducing healthcare disparities.
Families and Clinicians	Low-resourced families can be further disadvantaged	Families from socioeconomically disadvantaged backgrounds often faced barriers such as lack of digital devices, poor internet access, limited privacy, and unavailability of necessary materials, which could widen existing disparities in access to diagnostic services.
Families and Clinicians	Lack of digital literacy hinders access	Limited digital literacy among some families and clinicians hindered their ability to participate in or conduct telehealth assessments effectively.

#### Appropriateness of telehealth.

##### Theme 1. Children with a mild presentation difficult to assess over telehealth

Overall, clinicians found children with a mild presentation and subtle autistic traits harder to assess virtually compared to children exhibiting more pronounced difficulties or greater developmental impairment [63,67,69,70,75]. Clinicians noted that novel telehealth tools (e.g., NODA) were sometimes unable to capture subtle deficits in social communication and interaction or atypical behaviors, often necessitating an in-person assessment using ADOS [70]. Similarly, children with good verbal skills and flexible language were difficult to diagnose remotely [67,69,82].

*The one’s who I struggle with, we struggle with as a team, are the higher functioning children, like they’re five, and their verbal. Because then I want to see their face. I want to be right in front of them to catch the more subtle errors and omissions, and I’m not seeing it on video* [69].

Quantitative data corroborates these observations, indicating that children with mild presentation are less suitable for telehealth evaluations. For example, Corona et al [61] found that children initially missed in telehealth assessment but later diagnosed through in-person evaluation scored lower on TELE-ASD-PEDS and ADOS-2 and had higher adaptive skills. Another study found that children diagnosed via telehealth who did not require a follow-up in-person assessment had higher CARS-2 scores [73]. Additionally, McNally Keehn et al found that children with more pronounced autistic traits were more likely to receive a definite autism diagnosis than an “ASD unsure” outcome [72]. Finally, clinicians’ satisfaction with the assessment was positively correlated with the child’s symptom severity [74].

Reflecting on the difficulties assessing mild ASD-related difficulties, clinicians mentioned the role of “masking”, which can obscure relevant autistic features and behaviors. Some clinicians further noted that technical issues might induce “conversational asynchrony in the absence of social skills deficits”, potentially leading to erroneous diagnostic impressions [75]. Nevertheless, clinicians were confident that telehealth assessments are valuable for children with no autistic traits, where diagnostic decisions could be made with certainty [75].

##### Theme 2. Younger children might be less suitable for telehealth

The child’s age could influence clinicians’ ability to conduct an accurate observation and reach a diagnostic determination. In some studies, clinicians found that younger children, especially those with pronounced developmental impairments, were easier to assess via telehealth compared to older children with a milder presentation and good verbal skills [67,69,82]. Another study found that children receiving a diagnostic decision via telehealth tended to be younger on average than those referred for in-person evaluation [73].

Conversely, other studies identified younger children as more challenging to assess, as parental involvement and coaching were often required, limiting clinicians’ reliance on their own observations [63]. Additionally, younger children were less attentive compared to older children, which could hinder a satisfactory observation [70]. Furthermore, clinicians did not have to make significant modifications to their assessment procedures when assessing older children, emulating in-person assessments [63]. Similarly, families believed that it was more challenging for clinicians to accurately assess the abilities of younger or less verbal children online [67]. In their study, Corona et al [61] found that younger children were more likely to be misdiagnosed via telehealth, while in another study clinicians were more certain about the diagnostic outcome for older children compared to younger children [72].

Therefore, many clinicians concluded that telehealth assessments for younger children tend to be more complex, less reliable, and less appropriate [75,76]. In contrast, telehealth may suit older children and adolescents, especially those with good language skills and strong evidence from parents or schools [63,75].

##### Theme 3. Hyperactive children struggle with telehealth assessments

Clinicians’ capacity to conduct remote observations depended on the child’s activity level. Highly active and inattentive children were challenging to observe virtually [63,70,75], as they often moved out of camera view and struggled to stay still, while clinicians had little control over their behavior [63,69]. In such instances, clinicians were less confident in distinguishing ASD-related behaviors from other conditions, often necessitating an in-person evaluation [70]. Children with ADHD were also considered less suitable for a telehealth evaluation [63]. Families of children who struggled to engage with the screen also reported difficulties with the virtual assessments and exhibited a preference for in-person evaluations [63].

*I would say some of the kids that were quite hyperactive made it difficult because they wouldn’t stay in front of the camera or they’d go other places and you had to try and move around with them* [63].

##### Theme 4. Children with mental health and medical needs less suitable

Clinicians reported difficulties conducting virtual assessments for children with complex presentations, such as those with co-occurring mental health difficulties [75], heightened anxiety or behavioral inhibition [69]. Phelps et al found that children for whom a diagnostic decision was made via telehealth had fewer diagnoses of psychiatric disorders (i.e., mood or anxiety disorders) compared to those referred for an in-person evaluation [73]. Importantly, many clinicians felt unable to adequately support children with significant distress or related difficulties remotely [75].

Children with challenging behaviors who refused to engage also required an in-person assessment [63,69]. Furthermore, some clinicians questioned telehealth’s reliability for children with speech and language difficulties, intellectual disabilities, and learning difficulties [76].

Children with medically complex profiles were additionally viewed as more appropriate candidates for in-person evaluations [66]. Clinicians found assessing children with complex medical needs and other diagnoses, such as developmental delay or substance exposure, challenging [70,75,76,82], suggesting that a comprehensive evaluation using ADOS was necessary to establish their diagnosis [70].

*For others, and perhaps the majority [of children], this is not possible. This is especially the case when [the] presentation is complex, and there are other hypotheses about the root of the clients’ areas of difference (e.g., developmental trauma, acquired brain injury)* [75].

Table 7 summarizes the themes concerning the appropriateness of using telehealth for the assessment of autism.

**Table 7:** Summary of themes related to the appropriateness of telehealth autism assessments.

Stakeholder Group	Descriptive Theme	Summary of Findings
Clinicians	Children with a mild presentation difficult to assess over telehealth	Clinicians found it challenging to assess children with subtle or mild autistic traits via telehealth, often necessitating an in-person follow-up evaluation to reach a diagnostic conclusion.
Families and Clinicians	Younger children might be less suitable for telehealth	While views varied, younger children were generally considered more challenging to assess remotely, due to difficulties engaging with screen, their limited verbal skills, and reliance on parental coaching and reports.
Families and Clinicians	Hyperactive children struggle with telehealth assessments	Children with high levels of activity struggled to engage in telehealth assessments, hindering clinicians’ diagnostic confidence and rendering an in-person evaluation more suitable.
Clinicians	Children with mental health and medical needs less suitable	Children with co-occurring mental health difficulties or complex medical needs were less likely to receive diagnostic clarity via telehealth and were viewed as more suitable for an in-person assessment.

## Discussion

This is the first review to systematically examine families’ and clinicians’ experiences using telehealth for the assessment of children for autism. Caregivers and practitioners elaborated on a plethora of advantages that telehealth introduces to the assessment procedure, while they detailed several disadvantages associated with the virtual nature of the assessment. Both stakeholders also discussed the impact of telehealth on equity for certain populations and offered their insights on the groups of children who are better candidates for a telehealth evaluation.

Both stakeholders were satisfied with telehealth, noting it could expedite care by reducing wait times and enabling timely interventions for autistic children. Given that evidence-based interventions improve prognosis [83], developmental outcomes, and multiple domains of functioning [84–86], telehealth can have a long-lasting positive impact on autistic people’s lives. Telehealth also improves service access during restrictions (e.g., COVID-19 pandemic) and allows more flexible appointments. Several reviews highlight telehealth’s beneficial role in maintaining services during COVID-19 while ensuring the safety of patients, providers, and the general public [87]. Telehealth assessments for autism can also reduce costs for families, clinicians, and services, aligning with the broader literature suggesting significant cost reductions for medical services [88], including those offering interventions for autistic children [89,90]. The home environment could also help elicit children’s typical behaviors, enabling clinicians to make accurate judgments about the children [69,76]. This aligns with recent reviews suggesting that telehealth can be comparable or even superior to in-person services in offering clinical services to paediatric populations [91,92]. Families also appreciated receiving diagnoses and recommendations remotely, consistent with findings from a review suggesting that both providers and families are satisfied with using telehealth for the diagnosis of neurodevelopmental concerns [93].

A common disadvantage of telehealth involves technical difficulties. Although this is a common finding in the telehealth literature [94], in the included studies clinicians perceived these as disrupting their ability to complete the assessment and acquire relevant information only in a minority of cases [40,71]. Other barriers related to the family’s home setup and active role in the assessment. While families valued their involvement, both they and clinicians highlighted the importance of parents having a clear understanding of the assessment procedure and their role [67]. Therefore, caregivers should receive clear instructions about the assessment, including relevant materials they need (i.e., toys), their role and technical guidance in advance [63,67]. Organizational support is also essential for clinicians, including infrastructure, training in telehealth, and telehealth-focused supervision. Additionally, the establishment of new policies, clear legislation and clinical guidelines is deemed necessary [75].

Interestingly, while most families reported a good relationship with practitioners, the latter often reported difficulties in developing rapport with families. This discrepancy might stem from caregivers’ active role in the assessment, which, though valued by families, could be perceived as a barrier by many clinicians. Establishing patient-provider rapport via telehealth can be particularly complex, with patient-related, provider-related, technology-related, and other institution- or organization-related factors influencing the quality of the relationship [95]. For instance, technological difficulties (e.g., poor connection), lack of prior face-to-face interaction, telephone-only consultations, limited patient English proficiency, and socioeconomic status are a few factors that can introduce challenges to the provider-patient relationship [96]. Clinicians can improve rapport with families by learning how to navigate the virtual environment effectively, managing verbal and nonverbal communication, conveying empathy, fostering mutual respect and understanding, and demonstrating cultural humility [97].

Caregiver and clinician views on telehealth assessment’s accuracy were mixed. Although many clinicians were surprised by its accuracy, some believed that telehealth is less accurate than in-person assessments [67,76]. This contrasts with recent findings from a quantitative review showing that telehealth assessments for autism demonstrate high accuracy compared to traditional, face-to-face assessments [35]. Differences in perceptions could be explained by multiple factors, such as tools utilized, prior telehealth experience, inadequate infrastructure and poor organizational support, and preexisting attitudes towards telehealth. Clinicians also noted that observing and accurately assessing non-verbal and subtle ASD behaviors and traits remotely was challenging, limiting their diagnostic confidence [69,75]. Although establishing a diagnosis via telehealth might not always be possible or appropriate, highlighting its benefits, including the clinical effectiveness [92] and accuracy compared to in-person assessments [35], could improve clinician attitudes towards this modality.

In terms of equity, families in remote geographical areas, a population faced with barriers to accessing services [28], benefited from telehealth [62,67]. Nevertheless, families with limited English proficiency and those socially and economically disadvantaged faced additional barriers. While this finding primarily reflects experiences from English-speaking countries, limited command of the dominant language of a given country, irrespective of the specific language, may significantly affect access to telehealth services across different national contexts. This aligns with the broader literature proposing that although telehealth overcomes geographical barriers, it has yet to address social barriers [98]. A recent review exploring the use of telehealth during COVID-19 concluded that the greatest increase in telehealth utilization was by non-minority populations, such as younger, English-speaking, and White individuals [99]. Importantly, these barriers do not exist in isolation, and they are likely to intersect [100]. For example, non-native-English-speaking families are more likely to face socioeconomic difficulties and lack both access to and knowledge of how to use appropriate technological devices [67]. Therefore, efforts should focus on engaging with disadvantaged communities to understand the structural barriers they encounter, including the ways these impede their access to telemedicine, and offer tailored solutions aimed at promoting equitable implementation of telehealth [63,100,101]. Examples include enhancing language accessibility (e.g., by offering language-concordant providers and multilingual telehealth platforms), providing technological support and resources, and delivering relevant education [101]. Families who lack the capacity to participate in telehealth, even after offering accommodations, and those preferring an in-person assessment, should be given the option to undergo a face-to-face evaluation in a clinical facility [63,101]. Yet, many of the factors impeding access to telehealth similarly hinder access to face-to-face care, underscoring the need for comprehensive interventions and strategies that address the broader structural determinants limiting equitable access to neurodevelopmental services [25].

In terms of the children’s characteristics that may render them more suitable candidates for a remote evaluation, clinicians found children with complex medical or psychosocial presentations, as well as those with mild autistic features more difficult to assess virtually, necessitating an in-person clinical evaluation. In contrast, older children, those for whom information was already available, and those manifesting clear autistic traits could be diagnosed more comfortably. Therefore, services offering telehealth assessments should introduce streamlined neurodevelopmental assessment models with pre-assessment screening procedures to determine case suitability for virtual evaluation. Screening tools should also explore children’s activity levels, given that hyperactive children may struggle to participate in virtual assessments. Children with fewer medical and psychiatric comorbidities, those scoring highly in autism-specific or broader developmental screeners, and those for whom adequate pre-assessment information is available may be offered telehealth appointments [76].

While telehealth might not fully replace traditional assessments, it offers multiple benefits for families and providers. Telehealth can serve as an initial step for clinicians to gather information that informs in-person diagnostic assessments. It also allows clinicians to work flexibly and reach populations that face barriers to attending neurodevelopmental services. Ultimately, telehealth can reduce the gap of care by expanding access and improving efficiency through early screening and assessment of children with developmental concerns.

### Limitations and future directions

Some limitations of this review should be noted. Most studies were conducted in high-income Western countries (e.g., USA); therefore, results may be less applicable to low- and middle-income countries where additional barriers to telehealth access may exist. Furthermore, many lacked details on the assessment procedures followed. Given the large heterogeneity in the way telehealth consultations were performed (e.g., platform utilized, appointment length, and tools used), participant experiences may be tied to specific elements of the procedures rather than telehealth itself, complicating cross-study comparisons. Moreover, although a few studies attempted to explore participant characteristics that can influence satisfaction and experiences, most studies did not explore these associations. Non-response bias was also evident in most studies; potential participants who were unable or unwilling to attend virtual assessments did not share their views. It is important to note that most studies were conducted during or shortly after the COVID-19 pandemic, when clinicians and families had limited telehealth experience, guidance, and infrastructure. This could skew the results and partially explain some of the negative experiences both stakeholders reported.

Future studies should elucidate the optimal use cases of telehealth for the assessment of autism and identify which children benefit most from virtual assessments. Emphasis should be placed on ensuring that telehealth is offered equitably to serve diverse populations. Importantly, it is crucial that clinicians conducting neurodevelopmental assessments are trained in telehealth tools and protocols, and that efforts are made to improve clinicians’ attitudes towards this modality. Novel tools developed for telehealth use should be further researched and validated across various contexts. Finally, telehealth protocols should be co-produced with families and children to ensure they are acceptable and engaging.

## Conclusions

Although research is still in its infancy, the current evidence suggests that telehealth is an acceptable means of conducting diagnostic evaluations for autism. Telehealth has the potential to significantly improve service delivery and expedite families’ access to neurodevelopmental assessments, enhancing clinical effectiveness and efficiency. Given that many of the barriers reported in this paper are surmountable, the optimization of telehealth diagnostic protocols should be prioritized at both service (i.e., infrastructure and effective triaging) and clinician levels (i.e., attitudinal improvement and training). Equity factors should also be addressed to ensure that all families can benefit equitably from the introduction of technology to neurodevelopmental diagnostics. Ultimately, telehealth should not be treated as a “one-size-fits-all” solution and instead should be offered for certain groups of children who are deemed suitable and when families are agreeable and possess the appropriate resources.

## Supporting information

S1 Checklist PRISMA checklist. (DOCX)

S1 Data Family data file. (DOCX)

S2 Data Clinician data file. (DOCX)

S1 Table Critical appraisal of the studies included using the MMAT. (DOCX)

S2 Table List of studies subjected to full-text screening. (DOCX)
