# Supplementary material for: Families’ and clinicians’ experiences with telehealth assessments for autism: A mixed-methods systematic review
Source: PLOS Digit Health. 2025 Jul 29;4(7):e0000931. doi: 10.1371/journal.pdig.0000931 (PMC12306760; doi:10.1371/journal.pdig.0000931)
Supplement: S1 Data — (DOCX) [file pdig.0000931.s002.docx]

| Data on families’ experiences and attitudes towards telehealth | |
| --- | --- |
| Study | Qualitative and “qualitized” data |
| Pedernera Bradichansky et al. (2021) | The vast majority of families (87%) had not attended a virtual consultation prior to the COVID pandemic  83% of the families used their mobile phone to attend the virtual consultation  The vast majority of families (84%) felt comfortable attending a virtual neurodevelopmental evaluation  More than half of families (61%) reported that avoiding travel was a significant advantage  Just less than half of the families (44%) valued the importance of their child being in their natural environment when attending the evaluation  A quarter of the families (25%) reported that attending the evaluation virtually allowed them to save money  More than half of the families (54%) surveyed found the process of participating in a virtual diagnostic assessment easy and felt that they were guided adequately  The majority of families (70%) expressed their willingness to attend follow-up appointments remotely, although almost half of the families (48%) reported that they missed in-person consultations  A small percentage of parents (16%) experienced some difficulties associated with the virtual consultation, such as difficulty downloading the appropriate software or other technical difficulties |
| Corona et al. (2021) | Parents reported feeling highly comfortable playing with their children as part of a structured observational tele-screening using TELE-ASD-PEDS  Parents felt that the spoken instructions of the assessment using TELE-ASD-PEDS were easy to follow  Parents acquired a good understanding of the tele-screening procedure before it started  Parents believed that the clinician could effectively use the activities with the child and themselves  Almost all parents surveyed were satisfied with the overall duration of the tele-screening procedure, which lasted less than 30 minutes  Parents were generally comfortable being talked about a diagnosis of autism by a clinician after completing screening via telehealth using TELE-ASD-PEDS  The vast majority of parents (88%) believed that the tele-screening activity could largely elicit the child behaviors of concerns  Most parents (77%) indicated that they would prefer to both play with the child themselves and observe the clinicians play with the child in the context of a tele-assessment. A smaller percentage (19%) preferred to play with the child only by themselves, and a small minority would prefer to observe the child play with the clinician (4%)  When asked about changes they would make to the screening visit, 19 parents provided comments. Most comments (n=13) focused on technology, including concerns related to audio quality, the small size of the screen, and their children’s interest in accessing or touching the screen and camera. Parents also commented on the screening activities (n=4), including suggestions for adding more or different activities. Two parents suggested extending the amount of time for screening. As noted above, screening visits lasted 23 min, on average. One parent wrote that a screening visit of 30–45 min would be preferable  When asked what they liked more about the tele-screening, compared to their child’s full diagnostic evaluation, 32 parents provided a total of 35 comments. The majority of these comments (n=14) emphasized the parent-led nature of the screening process. Parents commented that they liked being involved and that their children were more comfortable or better able to pay attention when interacting with a parent rather than an unfamiliar clinician. Other comments focused on the shorter time of the screening as compared to a full diagnostic evaluation (n=6), the convenience of the screening process (n=4), and that the screening purpose and instructions were easy to understand (n=3)  When asked what they liked less about the tele-screening process, 15 parents provided a total of 16 comments. Three parents wrote that there was nothing they liked less. Four parents commented on technology issues. Four parents indicated that the full evaluation had gone into more detail, or that the behaviors they were most concerned about were not apparent during the tele-screening. Three parents noted that the tele-screening process was less personal than the full evaluation, and one parent voiced concern that they were not administering the activities correctly  Finally, when asked for other comparisons between the screening and full evaluation or other general comments, 36 parents provided a total of 46 comments. These comments addressed many of the topics described above, including technology, parent and child comfort, and parents’ role in the process. Some parents (n=8) commented that they thought that tele-screening may be useful as an initial step in the evaluation process, but also wanted face-to-face interaction with a provider or an opportunity for a provider to interact with their child as well (e.g., “I feel it was a comfortable tool for parent child but because it’s not in person, it might be easier to miss something, for that reason maybe should have more than one screening”)  “The video kept cutting in and out making it hard to hear and understand what was being said.” [technology theme]  “My child interacted with me rather than people he didn’t know” [Parent-led nature of screening theme]  “I enjoyed being a part of the process but worried my interaction was too much or too little” [Parent-led nature of screening theme]  “It feels like if the screening tool was just that- a screening, it would be good. But maybe a full evaluation is still needed? For whatever reason, [my child] often performs well at these type of things- like today, he didn’t get frustrated or have a meltdown. I wonder if he would have been overlooked if he had only received this screening instead of the full evaluation he got.”[Comparing diagnostic evaluations to tele-screening theme]  “I like the telemedicine as it included me as a parent, but I also like the full evaluation as it allows me to see what happens when someone else is working with my child.” [Comparing diagnostic evaluations to tele-screening theme]  “I feel like a screening should last a little longer. Maybe 30–45 min.” [time theme]  “It was fast and was able to hold his attention with all the different task.” [time theme]  “Really easy screening. Very comfortable for both baby and I.” [Comfort/convenience theme]  “I would change to have toys out of eyesight for child. Maybe in a box next to parent.” [screening space and activities theme]  “I appreciated the questions about eye contact and the doctor’s patience.” [Clinician and staff communication theme]  “It was about the same play wise” [other theme]  “Never had a visit like this for her.” [other theme] |
| Corona et al. (2024) | Parents on average had a good understanding of what they were expected to do as part of the assessment  Parents reported that the instructions regarding the activities of the tele-assessment tool (TELE-ASD-PEDS) and their role were easy to follow  o a large degree, parents believed that the tele-screening activity could elicit the child's behaviors of concern  Parents found playing with the child as part of the tele-screening very comfortable  Parents were satisfied with the duration (average 39 minutes) of the tele-screening assessment  Parents would recommend the telemedicine screening procedure for ASD to other families  On average, parents reported that they were satisfied with the remote screening procedure for their children |
| Esther et al. (2022) | An overall positive experience was reported by families, even though some technical issues were present during the telehealth appointment: ‘I didn't really have anything negative besides when there are interruptions that you cannot help it’ (Child B). Families also commented that the staff were attentive, polite, thorough and respectful in the appointments: ‘Over the phone they were always respectful towards her, they listened to what we were saying’ (Child E).  **Convenience and flexibility.** Families enjoyed the telehealth appointment for its convenience, as indicated by their report of being able to attend the appointment from anywhere: ‘we can go back and do the work because it is online… It was easy to fit around work. I don't need to take leave and my husband to be on that day’ (Child J). Families can save travel time to the hospital, be more flexible with their time and reduce financial cost: ‘We did not have to travel because (the appointment was) on a working day. I just had to take the time out… otherwise I have to plan for it and take another 2 or 3 h (off work and spend) 40 min there and 40 back and 1 h at the hospital. It did save commute time’ (Child C); ‘I think it was very efficient time wise. Both of us could be there to the hour, there is no travelling time and it was cheaper’ (Child I)  **Access to service during the pandemic.** Parents/carers valued the telehealth appointment because it continued to provide access to services amid physical distancing, lockdown and geographical distance: ‘Obviously due to COVID it was helpful. We didn't have to go out in the community and put ourselves at risk. Yes, so that was definitely helpful’ (Child E)  **Support to families.** Parents/carers indicated that they were being supported by clinicians during the telehealth appointments: ‘I wrote a lot of the stuff down like the questions we wanted to get answered and they answered everything we needed to know and put us in the direction that we needed to go’ (Child F). Parents/carers also expressed that the hybrid service delivery model during the pandemic allowed for the initiation of the assessment process and access to early intervention to commence before a face‐to‐face appointment: ‘It is a chance to clear the air or his progress and where he is up to and doctor explained… therapies needed to be done. It was clearly explained about my situation in both my child and everything went well with telehealth at home’ (Child H)  **Technical issues.** Poor internet connectivity, audio issues and glitches on telehealth platforms were reported to be the common challenges that parents/ carers encountered during the telehealth appointment: ‘The platform was difficult to get into’ (Child A); ‘There were a few hiccups with the freezing of the screen and things like that here and there but (the clinician) picked up where we left off when it came back to normal again during the time’ (Child B)  **Disengagement in telehealth appointment.** Children's attention was reported to vary, with some being more distractible when attending this style of appointment, while others were more focused: ‘I think (the clinician) can get a much better understanding of my child in particular from being in person rather than on a computer on a platform… he doesn't know the difference between the video call right at the end of the day but he video calls his father twice a day… and to him it's a game, it wasn't serious’ (Child A); ‘We are in a safe environment and they are comfortable and they are focussed on the screen’ (Child B)  **Limited direct observation of the child.** Parents/carers expressed concerns about completing the assessment with their child using only telehealth. They believed that the clinicians are not able to complete all assessments via telehealth or to fully observe and understand their child when observed through the computer: ‘I feel (the clinician) can't really assess, face to face is better so they can really assess my child’ (Child H)  **Limited experience of using telehealth.** Unfamiliarity with the telehealth platform and technology was also reported to be a challenge for this hybrid service delivery model. Some families had limited experience in using the technology: ‘I think there are people that are not very tech. It might be a bit difficult to get on line and log on and do’ (Child B). (Esther 2022, Parents/carers).  Overall, despite the convenience, flexibility and opportunity to access the service during a pandemic, face‐to‐face appointments were perceived by parents/carers as being preferable over telehealth for engaging their child to gain a full understanding of their complex presentation: ‘For my daughter, I don't speak for other family, she can't really talk much because she can say some words she doesn't know how to talk on the phone you need to see her in person’ (Child G). The personal interaction that occurs in the context of in‐person appointments was also seen as preferable: ‘I think all in all I would prefer the in person even though it saves me a drive down there, I like to look at somebody in the eye and be in the same room when I talk to them as much as possible’ (Child D)  Appointments for clinical interviews, assessment feedback and any follow‐up sessions that did not involve direct assessment of the child were perceived as being more suited to telehealth: ‘If you just want to know what is the feedback, what is the current state of things then I think telehealth is better’ (Child C); ‘It depends on what it is. If you are being tested obviously in person but if it is just catching up then telehealth would be fine with me’ (Child I); ‘I think it depends on what it is for. If it is a bit more hard to explain or you need to see the child in person. Sometimes it is a bit difficult if you on a screen or on a phone but I think it just varies depending on the appointment’ (Child B)  While still having a positive experience in the telehealth appointment, some families where English was a second language preferred face‐to‐ face appointment as it was easier for them to communicate in that context: ‘Overall it was positive but I have a little bit language barrier because I am not an English native speaker so I prefer face‐to‐face’ (Child K). Other families from a culturally and linguistically diverse background favoured a combination of face‐to‐face and telehealth appointments. There was no clear difference in the preference of appointment type between cultural and language backgrounds  There was also no particular trend in the preference of appointment type based on the age of the child, diagnostic outcomes or assessment completed. Nearly half of the families reported that a combination of telehealth and face‐to‐face appointments was preferable, with the type of appointment dependent on the context of the consultation, availability and participants involved in the appointment  While most families did not offer suggestions for improving the telehealth experience, a few parents/carers commented that improving the quality of the telehealth platform and providing clearer instructions for navigating the telehealth appointments would be beneficial: ‘…as long as people have the information of what to do and how to do it prior… if it was in advance then step by step, go to this, click on this, log onto this or a link. I just think that being prepared in advance would help a lot especially for those that are not sure and they can jump on and figure it out…’ (Child B) |
| Gibbs et al. (2021) | **Tele-assessments are a convenient option** All three stakeholder groups considered tele-assessments to be a convenient option. The convenience of being assessed in the comfort of the clients’ own homes was especially relevant during the COVID-19 pandemic. “I think I would recommend telehealth assessments during a COVID-19 situation, definitely. It’s better to have the assessment by telehealth rather than not have it. (Carer #8)  The opportunity to have an assessment within a familiar environment meant clients felt comfortable and relaxed at home. “It is a lot more relaxed when the children are in their own environment. I think that’s a plus.”(Carer #7)  Although clients, carers and clinicians shared mostly positive technology experiences, they also described some instances where technology negatively impacted on their assessment experience. All three stakeholder groups expressed concerns that some important aspects of client behaviour may not be as apparent to the clinician in a telehealth assessment. There was a perception from clients and carers that subtle behavioural characteristics that might be important for clinicians to observe may not have been apparent when their view was limited to what could be seen on the screen. “I guess it might be a little bit limiting, you know, as you can only see what’s on the screen  “Some of his mannerisms, when he gets under distress, he does tend to…stand up and, turn around and do a little circle. So it’s not really that noticeable. But he does do that occasionally and under that circumstance that wouldn’t have been noticed.” (Carer #3) “I think in a different environment, he might’ve engaged differently…I think there would be a lot more distractions.” (Carer #12)  Similarly, some carers thought that an online setting was not the best option for assessing their children. “[Son] has moderate to severe difficulties in both areas of development relating to autism so for telehealth, it presented a bit of a challenge because he wasn’t really engaging with the screen. We found the same with his therapy, while his occupational therapist and speech were conducting telehealth sessions, we had very limited engagement with the therapist over the screen. So it made it very difficult.” (Carer #10)  All families found the quality and clarity of the video acceptable  All families found the quality and clarity of the audio (sound) acceptable  80% of families reported that they felt comfortable interacting with the clinician via the telehealth platform  A small proportion of participants (27.2%) believed that either they or the person they cared for were unable to do or say things online that they could have done or said in person., while 41.8% of them were neutral and 30% disagreed  The majority of the families (67.8%) would be willing to undergo an assessment online in the future, if they needed to, and only 3.6% disagreed  Only a small minority of families (12.5%) reported that the difficulties impacted the assessment to some extent |
| Hodge et al. (2024) | Phone interviews were completed with 10 parents/ caregivers; eight were unable to be contacted for an interview. The majority of parents/ carers reported that this was their first time using telehealth. Some parents/caregivers (n = 4) experienced technical difficulties during the TELE-ASD-PEDS, such as audio issues and poor internet connectivity, but most parents/caregivers (n = 6) did not encounter any technical issues. All interviewees reported they were experienced in using technological devices, as well as videoconferencing technologies. Overall, parents/caregivers were comfortable interacting with the clinicians and their child online. All parents/caregivers reported that the verbal instructions from the clinicians were easy to follow and most of them understood the assessment activities before the TELE-ASD-PEDS. However, there were inconsistent opinions on whether the TELE-ASD-PEDS captured children’s behaviours that their parents/carers were mostly concerned about. More than half of the interviewees expressed that they would prefer face-to-face appointments for future assessments. Two interviewees felt that the duration of TELE-ASD-PEDS did not allow for comprehensive assessments, as well as to determine the level of autism. In response to open questions, parent suggestions for improving the assessment process included meeting the families face-to-face before the TELE-ASD-PEDS, providing more information on the purpose of the assessment items, longer consultation time, and multiple appointments across a number of settings. Parents/caregivers reported that they liked the convenience of the assessment, as it was completed at home, being able to meet the clinical team, as well as receiving recommendations after the assessment.  The majority of parents used either a mobile phone or a tablet to access the telehealth appointment  In the vast majority of cases (80%) it took less than 10 minutes to set up and start the appointment  A small proportion of parents reported some difficulties hearing the sound or seeing the video, while half of them reported no issues  The majority of parents (80%) agreed that they felt comfortable playing with their child as part of this assessment  All parents agreed that the instructions of the assessment were easy to follow  The vast majority of parents (90%) had a clear understanding of what they were expected to do before the assessment started  Most parents (60%) agreed that the clinician could use the activities of the assessment with their child and themselves  Most parents (70%) believed that the assessment lasted about the right amount of time  80% of parents reported feeling comfortable with someone talking to them about a diagnosis of autism after the telehealth assessment  40% of the parents believed that the activities could elicit the behaviours of concern, while Half of the participants disagreed that the activities could elicit those behaviours  Most parents (80%) felt comfortable interacting with the clinician online  The majority of parents (80%) agreed that there were important things that either themselves or the child was unable to do or say online that they or the child would have been able to do or say in person  The majority of parents (60%) would not be willing to do assessments online in the future, while many of them were neutral (30%)  Most parents (80%) reported that the quality and clarity of the video was acceptable  Most parents (80%) reported that the quality and clarity of the audio was acceptable  Half of the parents agreed that they had a good experience using the telehealth technology for their child’s diagnostic evaluation, while 30% of them disagreed with that statement  Half of the parents would not use telehealth in the future if they had the option to attend a face-to-face appointment, while 40% of them were neutral |
| Jones et al. (2022) | Overall, the following six themes characterized the qualitative benefits of telediagnostic assessments: accuracy of the telediagnostic assessment, logistical convenience of the telediagnostic assessment, ease of administration of the telediagnostic assessment, clinical rapport and expertise, thorough explanation of diagnosis, and access to intervention services. Some caregivers discussed the accuracy of the diagnosis and highlighted the advantage of observing the child in their natural environment.  Most caregivers noted the logistical convenience of the telediagnostic assessment, including the safety of telediagnostic assessment during the COVID-19 pandemic and the convenience of not having to allocate time to travel or arrange transportation to the clinic.  A few caregivers commented on the ease of administration of the TELE-ASD-PEDS  Most caregivers described their rapport with the clinicians and the clinicians’ expertise, including the compassion of the clinicians, their connection with the clinicians, and an overall positive experience interacting with the clinicians  Caregivers reported on the benefit of the thorough explanation of their child’s diagnosis and information regarding next steps  Some caregivers reported the primary benefit of the assessment was to provide access to intervention services  The following four themes characterized the qualitative areas of improvement for telediagnostic assessments: inaccuracy of the telediagnostic assessment, technical issues, preference for in-person assessments, and need for additional information.  Some caregivers noted concerns that not all behaviors were present during the teleassessment and may lead to an inaccurate diagnosis  Some caregivers reported that an in-person assessment visit and/or seeing the child on multiple occasions would obtain a more accurate view of the child  One caregiver reported technical issues during the teleassessment  Most caregivers reported their preference for in-person visits, including the need for a follow-up in-person visit  Many caregivers reported that they needed more information than was provided during the feedback visits about their child’s diagnosis and next steps (Jones 2022, Families).  Some caregivers suggested that providing the report prior to the feedback visit would have better prepared them to ask questions and would allow more time for the feedback visits  Notably, accuracy of the telediagnostic assessment and inaccuracy of the telediagnostic assessment as well as thorough explanation of the diagnosis and need for additional information were conflicting themes across the benefits and areas of improvement for telediagnostic assessments  Accuracy of the telediagnostic assessment “I felt like the assessors really got to know my child and provided a great plan moving forward as well as accurate diagnoses.”  Inaccuracy of the telediagnostic assessment “Not sure everything was accurate since they wasn’t able to truly really evaluate him” “I feel the assessors would see more from the child in a in-person assessment then in a virtual assessment”  Logistical convenience of the telediagnostic assessment “Virtual was the best we could do for everyone’s safety” “Saved me time. Not worry about transportation.”  Ease of administration of the telediagnostic assessment “The assessment was easy to follow and it was also flexible. The team helped us adapt it to meet our sons needs.”  Technical issues “We did have some complications sometimes that could of been normal technical issues.”  Clinical rapport and expertise “You guys were so caring and we could tell how passionate you both are with helping kids and parents as well. You made us feel very comfortable with the overall assessment.” “Wow you ladies certainly care and are passionate about your field of expertise. I couldn’t have asked for two better women to handle this sensitive issue. My deepest thanks.”  Preference for in-person assessments “While the virtual assessment went very well, an in-person assessment would have been less stressful and our son would have likely been more focused.” “I think being in person is ideal but this was second best and I feel it was a valuable process”  Need for additional information “I don’t think I got a chance to ask all my questions” “The process of going over the results and recommendations felt rushed.”  Thorough explanation of diagnosis “My questions and concerns where very well explained and answered during virtual video assessment and I’m sure it would have been the same as in person assessment.” “Everything was explained very well about XXX’s Autism diagnosis.”  Access to intervention services “The diagnosis from the team and all the information they provided helped us qualify for services through his school district.”  There was a consensus amongst parents that the virtual assessment would benefit the child and the family to a certain degree  The vast majority of parents (82%) strongly agreed that the autism assessment was easy for them to follow along with and administer with their child  The vast majority of parents (82%) strongly agreed that the team asked questions that were relevant in getting to know their child’s strengths and challenges  The vast majority of parents (82%) strongly agreed that the team made an effort to get a good picture of their child’s development  The vast majority of parents (82%) strongly agreed that the team listened to and heard their thoughts throughout the assessment visit regarding their child’s development  The vast majority of parents (82%) strongly agreed that the team delivered the diagnosis and answered questions in a way that was easy to understand  The vast majority of parents (82%) strongly agreed that the team listened to and heard them throughout the feedback visit regarding their child’s development  The vast majority of parents (82%) strongly agreed that they had the time to ask all of their questions during the feedback visit  The vast majority of parents (82%) strongly agreed that by the end of the feedback visit, they had a good idea of next steps for their child  The vast majority of parents (82%) strongly agreed that the team’s provided recommendations and resources will be beneficial for their child.  All parents found they video platform simple to use for the evaluation  A minority of families believed that the audio and visual quality of the sessions negatively impacted their ability to participate in the evaluation, while 64% of them strongly disagreed  The majority of parents agreed that was easy to manage both their child and the technology during the evaluation session  The majority of parents (73%) disagreed that the virtual nature of the sessions made it difficult to connect with the assessment team  Generally, almost half of the participants (45%) agreed that virtual sessions seemed as effective as in-person sessions would have been, while 36% of them neither agreed nor disagreed  When asked whether the virtual sessions were more stressful than in-person sessions would have been, the views were polarised; 27% agreed, 36% disagreed, and 36% neither agreed nor disagreed  The majority of parents (64%) would have preferred to do an in-person assessment to this virtual assessment |
| Kellom et al. (2023) | **Assessment.** Participants commented on the experience of having a child assessed through telehealth, expressing different opinions. Most parents and providers agreed that the child being in their natural environment improved the physician’s ability to observe their usual behaviors.  At the same time, participants (few providers and some caregivers) also questioned the accuracy of an assessment where the provider was not able to physically examine the patient  Preparing families for where and how a visit would be conducted was important. Some parents voiced concerns that their child was harder to engage on a screen and was less interactive with the physician. Overall, when comparing telehealth and in-person experiences, many participants suggested “there are pros and cons to each.”  Parents highlighted how children acted like themselves surrounded by familiar people and things, which made parents feel as though the physician could make an accurate assessment.  **Diagnosis.** Regarding confidence with diagnoses, participants noted that certain diagnoses, whether assessments are completed in-person or virtually, are challenging to make. There were discrepancies, however, in which patients respondents perceived to be more difficult to assess by telehealth  Families believed that it was harder for physicians to get a sense of the abilities of younger or less verbal children  Both stakeholder groups discussed additional elements that are likely to affect parents’ acceptance of diagnoses received over telehealth  Both groups acknowledged that caregivers’ acceptance of and reaction to the diagnosis depended partially on how emotionally prepared they were to hear that diagnosis. While some parents said things like, “I was expecting the diagnosis, so there wasn’t a real concern because I knew it was coming,” others who felt less ready were more likely to question the process. Providers described learning over time that they needed to assess parents’ expectations and appropriately prepare them for an unexpected diagnosis. Preparation included guidance about identifying a place to receive the feedback to have time and privacy to process their feelings. Caregivers appreciated having time and space for this in their own home  Overall, this concern [difficulty establishing rapport] was not reported by families, who described feeling heard. Parents noted that it was easier to focus on the conversation in their own home, where their child could play safely instead of needing to keep them occupied in a doctor’s office  Providers and parents alike noted that technical issues were the most significant barrier to developing rapport and engaging in the virtual visit. Members of both stakeholder groups who experienced visits in which a visual was not available (e.g., telephonic visits or video visits in which the camera did not work) expressed concerns about their ability to engage emotionally  **Access.** The overwhelming sentiment from participants was that remote visits improve access to care and decrease costs for families. Caregivers and providers recounting family experiences described the benefits of having less time away from work or school; not having to worry about transportation, parking, and their associated costs; not having to find child care for siblings; enabling multiple caregivers to join from different locations; and mitigation of infection-related concerns during the pandemic. Participants highlighted that travel-related benefits may have been most impactful for families who are geographically distant from DBP providers and likely experience more travel burdens and families who have anxiety related to travel to a medical office  Participants described ways in which scheduling appointments were improved with telehealth options. Families believed that telehealth offered them more scheduling flexibility and allowed them to accept last-minute appointment slots.  **Technology and Equity in Telehealth.** A limitation of our data is that we only spoke with people who successfully completed a telehealth visit, so caregiver experiences described represent those who agreed to and successfully engaged in an initial DBP visit using telehealth. Given this selection bias, it is not surprising all caregivers self-rated their ability to use digital technologies as good or very good, and most own a smartphone, tablet, or laptop. Despite reporting familiarity with and access to digital technology, these factors frequently came up during interviews as barriers.  Providers and caregivers named the usability of the telehealth platform as a frequent barrier to access. Some parents described difficulties with navigating platforms, even when instructions were provided, or they considered themselves tech-savvy. Families who experienced technological barriers believed strongly that these issues detracted from the quality of the visit.  Caregivers noted the importance of having devices and internet access that supported visit engagement, for themselves and for capturing what the child was doing. Caregivers felt differently about which device worked best; some expressed that cell phones were too small, and some used a tripod to position larger tablets. While all interviewed caregivers did have internet access, providers noted that families without reliable internet or with limited data plans face additional barriers to telehealth. (Kellom 2023, Families)  **Efficiency of Care** Overall, providers and caregivers perceived an increase or no change in the efficiency of DBP care by telehealth  Both stakeholder groups described how the addition of telehealth could be beneficial in meeting family needs.  Both stakeholder groups suggested that families needed to be adequately prepared for the technological aspects of joining and the emotional aspects of conversations about diagnoses  Balancing the value of home environment and not having a physical examination. People expressed views across the spectrum, with most suggesting that having the child in their usual setting improved the physician’s ability to observe usual behaviors and others questioning the accuracy of an assessment where the provider was not able to physically assess the patient and mediated by a device that for some children is distracting  “There were pros and cons. What was nice was she got to see him in the house, in the background doing what he would normally do. Whereas in an office setting, he’d be shy. What was difficult was that he didn’t necessarily talk to her. She couldn’t do the physical examination. So like, he has generalized hypotonia, but she couldn’t see that so much. He’s not so bad that he’s not functional, but that’s something she couldn’t really see without physically touching him.”  Families noted the importance of having a private space to speak candidly about their child without the child present, which some managed at home, and others preferred about the doctor’s office. “I think it would have been much more difficult for me to speak as candidly as I did with the doctor. We end up trying to sort of speak in code, or spell words, or say something without saying it. And that can make things really difficult, and lead to miscommunication. When I can speak openly and really say what’s going on at home or with my child, I think that gives the doctor better information to be able to appropriately treat her. So, I think that was really helpful to me to be able to speak openly, and not have to worry about crushing her feelings or damaging our relationship.”  *S*ome providers and parents felt like something was lost by not being in the office. “There’s that scepticism of oh come on. You haven’t actually seen him in-person. Or you’re making the assessment, despite the fact that 100% trusted her and I heard exactly what she was saying and could see it as well, there was still that stigma attached to it, but you haven’t actually seen him. But I got past it pretty quick.”  Diagnosis and buy-in. Some providers believed that telehealth enabled parents to be engaged in the assessment and understand better what the provider was observing. “I think because I was able to focus better through telemedicine, I was able to actually hear what the doctor was saying. It’s so tough, sometimes, in person because again, I’m trying to keep the kids from damaging anything. So, I was actually able to be more present in the conversation through a virtual appointment.”  Assessing parents’ expectations and appropriately preparing them for a telehealth visit to discuss diagnosis was highlighted. “I think one of the things that I really liked about her, too is that when she was giving the diagnosis, she stopped and then asked if we were okay and if we thought that—did we think we were gonna hear this. So it just made me feel even more better knowing that this isn’t just—that she really cares about the whole picture, not just giving a diagnosis of a child and jotting information down. She really cares how we felt about this, as well.”  Establishing rapport with families. Some believed that establishing rapport was more difficult over telehealth, but overall, this was not a concern for families who described feeling listened to and heard. Compared to any other doctor visits, I mean, it’s—the only thing that it takes away is that— being impersonal and that kinda one-on-one that you get with them and just that more intimacy. But like I said, I think Dr. [Name] did a really great job explaining stuff. And it kinda felt like with her that she was in the room with us. The way she would stop and she would explain, the way that she made sure that me and my husband were okay with the results of everything. So it really wasn’t any issue.”  Remote visits are easy and decrease the cost of care for families. “I don’t have to get everybody ready an hour before our appointment to leave, because I don’t have to worry about parking or getting lost or getting stuck in traffic or my kids having a total meltdown in the car.”  Certain family characteristics make telehealth particularly beneficial for access, such as their geographic distance from DBP care. “There are benefits to telemedicine, that we’re able to do something. Something is better than nothing. Logistically, it was much easier because we do travel a significant distance to go to [city]. And it’s challenging with small children to travel. It’s more time in terms of taking time off for work arranging schedules.”  Telehealth seemed to facilitate appropriate triage for how different types of concerns were managed, which likely resulted in more families’ concerns being addressed more efficiently“. Wait times are so much less with telemedicine. Like I can get an appointment, maybe in a couple of weeks versus a couple of months, which is really great for [Hospital]. Also, yeah, for a follow-up question or something where I’m really just talking to a doctor versus like, getting a scan or something. Then it’s really helpful to not have to go into [Hospital] all the time. Because I do go in like all the time”  The usability of the telehealth platform was cited by some as a potential barrier to access, particularly for families with PLOE [preferred language other than English] because directions and platforms tended to be available only in English “We’re not going to learn every single system in the world, right? It should be something that it’s intuitive to everybody, like, oh, here’s a Zoom link, here’s a Team’s link, here’s a whatever. Just log in and you do it. If everybody has their own proprietary platform, then we’ll all be learning like 100 different platforms and we won’t be able to master any of them.”  Familiarity with and access to technology frequently came up as a barrier to access that participants noted affected some families more than others. “I mean, if you sign up for telemedicine, you’re—you have to be prepared for the device. I mean, we had an iPad. I don’t know how it would go off of a smartphone or with that because I know not a lot of people have these devices and it wouldn’t be able to work for them. It just was able to work for me. But just the fact that if it froze or if we lost connection, that would be the only thing that I could see being frustrated and see where results wouldn’t be accurate. But it didn’t happen to me. But I could see that potentially down the line because you can’t control technology.”  Certain family characteristics may make telehealth less comfortable or feasible, including families experiencing housing insecurity or who do not have a private/quiet space. But I just think in certain circumstances, depending on the child and their needs, we should be able to come in, because technology isn’t— because my area where I live, it’s real frustrating to get reception. So it’s like, a lot of the times is going in and out."  Given some of the behavioral concerns of children presenting for DBP care, there are some unique benefits of telehealth for the patients and their families. “Getting her in the car, getting her to the appointments, getting her into the doctor. I mean, it’s just a challenge from start to finish. And then a lot of times we’ll end up having meltdowns after because it’s so chaotic in the doctor’s office. There’s just a parking garage, and construction, tons of noise and lights. And that’s very overwhelming for her, so it makes the rest of the day very difficult. So, from that aspect, it was a lot easier for her to interact with the doctor through telemedicine because she didn’t have all those other obstacles in her way.”  Families appreciated being directed to access notes and recommendations through electronic portals after the visit.  Overall the majority of families (73.7%) where satisfied with the telehealth visit  The majority of families (66.7%) would use telemedicine again for the assessment of their child, while around a quarter of the families (23.8%) were not willing to utilise telemedicine |
| Juárez et al. (2017) | Families saved an average of 3.92 hours of estimated travel time relative to visiting the hospital-based ASD clinic  Families reported being highly satisfied with the clinician’s ability to engage with them via telehealth  Families mentioned that they were very satisfied with their ability to communicate their concerns to the clinicians via telehealth  Families believed that the clinician was able to collect important information about their child via telehealth  Families reported high satisfaction with the information received through the telemedicine appointment and believed that those could help them make decisions for the next steps in the child’s support plan  The vast majority of the parents (90%) agreed that the equipment used to attend the telemedicine visit was not distracting and did not reduce the effectiveness of the visit  The vast majority of parents (97.5%) agreed that their telehealth visit was as private as an in-person visit  All families agreed that telemedicine made it easier and more convenient for them to attend the appointment for their child  All parents reported that they would be very likely to recommend telemedicine to others  Around 97.5% of parents would be interested in attending medical appointments via telehealth  All parents reported being highly satisfied with the telehealth assessment procedure |
| Esther et al. (2022) | The majority of parents/carers (92%) agreed that they were involved in decisions about their child's care  The vast majority of parents (96%) felt that their child was treated respectfully  All parents surveyed agreed that clinicians explained things in a way they could understand  The majority of parents (64%) of parents/carers believed that their children felt comfortable during the telehealth appointment, while 28% of them were unsure about how comfortable their children felt  Most parents/carers (92.0%) were happy with the service their children received via telehealth  Only a minority of parents (24%) would prefer to attend a telehealth session over an in-person appointment when it is a first-time appointment with a new clinician  The majority of parents (68%) would prefer to attend follow-up appointments via telehealth over an in-person appointment  Most parents (60%) would prefer to receive ‘good news’ about their child’s outcome via telehealth  Only a minority of patients (36%) would prefer to receive ‘bad news’ about their child’s outcome via telehealth  Only a small proportion of parents (24%) would not be comfortable attending a telehealth session for physical assessment, even if this could be completed over video  The majority of parents (72%) would be comfortable using telehealth for any appointments that clinicians say they don't need to be face to face  Only a very small percentage of parents (4%) would like all possible appointments to be face-to-face  A few parents (4%) mentioned that telehealth allows them to access specialist care that isn't available where they live  A few parents (8%) mentioned that telehealth could help them receive advice to help understand/manage the condition  An advantage reported by some parents is that telehealth could allow other members of the healthcare team to attend the consultation  The majority of parents (88%) mentioned that accessing medical consultation via telehealth was convenient  Some parents (36%) believed that an advantage of utilising telehealth is that it helped them save money  Most parents (76%) believed that an advantage of utilising telehealth is that it helped them save time (e.g., did not take excessive time off work or school)  Almost half of the parents surveyed perceived their ability to stay closer to home and family as a significant advantage of telehealth  A significant percentage of parents (76%) agreed that the telehealth assessment would allow for social distancing and isolation  A minority of parents (32%) believed that not being physically present meant there were limitations to the consultation  Only very few parents (8%) did not have easy access to internet‐enabled devices or Wi‐Fi/broadband  Issues with interpreter services were reported only by 4% of parents  Poor video quality was reported by 4% of parents  A small minority of parents found it difficult to concentrate in the telehealth session in the home setting  More than half of the parents surveyed (52%) mentioned that there were no disadvantages to the telehealth session  Some parents (28%) experienced difficulties hearing the assessor  Some parents (8%) experienced difficulties seeing the assessor  Only 4% of parents had their connection dropping out  Only 4% of parents had problems with their equipment (e.g., microphone or webcam)  A small minority of parents (8%) mentioned that they needed to change from one platform to another because the technology wasn't working  More than half of the parents mentioned that there were no issues with the telehealth consultation  Forty‐eight percent of parents/carers indicated that they were extremely likely to recommend telehealth to friends and family, with only 20% not so likely to recommend to others  64% of parents or carers reported that they would use telehealth again and 12% were unsure |
| Kennelly et al. (2022) | No significant differences in patient satisfaction for pediatric neurodevelopmental visits whether they were performed in-person or via telemedicine  A big percentage of families (89.08%) were highly satisfied with the concern that clinicians demonstrated towards their questions and worries  The vast majority of parents (92.2%) were very satisfied with the explanations the clinician gave about their child’s problem or condition  Most parents (83.70) were satisfied with the clinician’s efforts to include them in decisions about the child’s care  The majority (89.08) of parents were satisfied with the fact that clinicians used language they could easily comprehend  The vast majority of families (95%) were satisfied with the sensitivity displayed by clinicians towards the child’s needs  The vast majority of families (95%) were satisfied with the clinician’s concern about the child’s privacy  82.37% of parents were in general satisfied with the overall assessment procedure |
| Matthews et al. (2021) | On average, caregivers found the telehealth assessment largely acceptable (5.87 on a 7-point scale)  Only 16% of caregivers provided neutral or negative responses when asked how acceptable the assessment was. More than half of the participants (58%) found the telehealth model highly acceptable  The age of the child was not associated with the parent-rated acceptability  Parents of female clients rated the telehealth model as significantly more acceptable than parents of male clients  Parents of female children a) felt more comfortable using telehealth to complete the child’s diagnostic assessment, b) reported higher agreement with the statement that the clinician had expertise in autism diagnosis, c) were more likely to agree that the child seemed as connected to the staff through telehealth as s/he would have in person, d) were more likely to report that telehealth assessment was as thorough as an in-person appointment would have been, e) would be more willing to use telehealth to complete a diagnostic assessment even if in-person assessments were available, f) were more likely to believe that the results would have been different if the diagnostic assessment had been completed in person, g) indicated higher agreement with the statement that the psychologist satisfactorily addressed their questions and concerns during the telehealth feedback session, and h) were more likely to appreciate the opportunity to complete this evaluation trough telehealth, rather than wait for an in-person appointment  On average, parents agreed that they felt comfortable with the virtual diagnostic assessment  On average, parents strongly agreed that they felt comfortable talking with the psychologist via the telehealth portal  On average, parents agreed that the psychologist demonstrated expertise in diagnosing autism  On average, parents believed that their child seemed as connected to the staff through telehealth as s/he would have in person  The majority of parents believed that the telehealth assessment provided an opportunity for an accurate demonstration of the child’s behavior and interaction skills  On average, parents somewhat agreed that the telehealth assessment was as thorough as an in-person appointment would have been  Parent’s views were polarised when asked whether they would be willing to use telehealth to complete a diagnostic assessment even if in-person assessments were available, although the majority tended to somewhat agree  Parents on average strongly agreed with the results of this telehealth assessment  On average, parents disagreed that the results would have been different if the diagnostic assessment had been completed in person  The majority of parents valued the information the psychologist shared during the telehealth feedback session  The majority of parents strongly agreed that the psychologist satisfactorily addressed their questions and concerns during the telehealth feedback session  The majority of parents highly appreciated the opportunity to complete this evaluation through telehealth, rather than wait for an in-person appointment |
| McNally Keehn et al. (2022) | The vast majority of Families (99%) agreed that they were able to communicate their concerns to the telehealth provider during the visit  The vast majority of Families (93%) agreed that the telehealth provider was able to collect important information about their child  The vast majority of Families (95%) agreed that the information they received through the telehealth appointment will help them make decisions for the next steps in their child’s support plan  The majority of Families (92%) agreed that the equipment used during the telehealth visit was not distracting and did not take away from the effectiveness of the visit  The majority of Families (96%) agreed that the telehealth visit was just as private as an in-person evaluation  The majority of Families (91%) agreed that Telehealth made it easier and more convenient for them to visit with a provider  Families agreed (90%) that they were likely to recommend telehealth to others  The majority of Families (87%) agreed that they would be interested in participating in future telehealth visits at this clinic  The vast majority of families (95%) strongly agreed that they were satisfied with the telehealth experience  Nearly 30% of caregivers reported that the telehealth evaluation saved them 3 or more hours of travel time as compared with whether they had travelled to the clinic for traditional in-person evaluation  Almost half of the participants (45%) would prefer to have a future evaluation via telehealth |
| Reese et al. (2013) | Families in the telehealth condition and in-person condition reported similar levels of satisfaction  Families on average agreed that they felt comfortable talking to the clinicians online  Families on average agreed that the clinicians cared  Families on average agreed that the clinicians were competent  Families on average strongly agreed that they did not experience difficulties hearing  Families on average somewhat agreed that they did not experience trouble seeing  All families reported high levels of satisfaction with the telehealth assessment |
| Reese et al. (2015) | Overall, parents felt that their concerns were addressed adequately  Overall, parents Felt that clinicians recognised the child’s strengths and needs appropriately  Overall, parents reported that clinicians provided helpful and appropriate recommendations for their children Overall, parents Satisfied with the way the diagnosis was explained  Overall, parents Felt that clinicians considered their input  Overall, parents Communicated the resources for services that were available in their community  Overall, parents reported that the clinicians were respectful of their cultures and values  Overall, parents same levels of satisfaction between in-person assessment and telehealth assessment |
| Reisinger et al. (2022) | Approximately 91% of caregivers who completed the survey in the present study reported they were satisfied with the telehealth evaluation  Caregivers of children with lower adaptive skills were more satisfied with the telehealth evaluation  Caregivers of female children (M=4.7, SD=0.6) reported slightly higher satisfaction than caregivers of male children (M=4.5, SD=0.6)  Caregiver satisfaction was also significantly related to ASD diagnostic outcomes. Caregivers of children with greater ASD symptom severity were more satisfied with their telehealth evaluation  Caregivers of children diagnosed with ASD reported higher satisfaction ratings (M=4.7, SD=0.5) in comparison to those who did not receive an ASD diagnosis (M=4.0, SD=0.7) or the provider was unsure (e.g., could not rule in or out) of the diagnosis (M=4.3, SD=0.5; p=0.017)  Caregiver satisfaction was also significantly related to provider diagnostic certainty (p=0.02). Specifically, caregivers were more satisfied with providers who were certain about their ASD diagnosis (M=4.6, SD=0.6) in comparison to those who were uncertain (M=4.1, SD=0.8)  Caregiver satisfaction was also significantly related to their visit preference with caregivers preferring telehealth visits (M=4.8, SD=0.3) being more satisfied than those who preferred an in-person visit (M=4.3, SD=0.7)  All parents felt the telehealth provider was engaged and part of the visit  99% of parents were satisfied with their ability to communicate their concerns to the telehealth provider during the visit  94% of parents felt the telehealth provider was able to collect important information about their child  98% of the parents believed that the information they received through the telehealth appointment would help them make decisions for the next steps in their child’s support plan  87% of the parents were satisfied with the equipment used during the telehealth visit, which was not distracting and did not take away from the effectiveness of the visit  Around 96% of parents believed that their telehealth visit was just as private as an in-person evaluation  86% of parents agreed that telehealth made it easier and more convenient for them to visit a provider  86% of parents would be likely to recommend telehealth to others  81% of parents would be interested in participating in future telehealth visits at this clinic  Overall, around 91% of parents were satisfied to a certain degree with the telehealth evaluation, and 71% of them reported being very satisfied |
| Talbott et al. (2020) | Before completing the visit, Parents on average felt confident in their ability to use the telehealth system to participate in the assessment  Parents found the quality of the video, audio and connections highly acceptable  After completing the assessment, parents felt confident in their ability to use the telehealth system to participate in assessments in the future  On average, parents agreed that the assessment accurately reflected their child’s usual behavior  On average, parents agreed that the assessment session was an acceptable duration  On average, parents understood their role in the assessments  On average, parents felt satisfied with the materials and believed that it was helpful to receive the toy box  On average, parents felt well-supported by the assessment coaching/staff in spite of the distance  On average, parents reported being very willing to recommend this telehealth assessment procedure to other families  On average, parents strongly agreed that participating in the telehealth assessment was convenient  When asked whether they would choose a live assessment over a telehealth assessment, no matter the distance, on average participant’s stance was neutral, indicating that answers were mixed  When asked whether they would choose a telehealth assessment over a live assessment, no matter the distance, on average participant’s stance was neutral answers were mixed |
| Talbott et al. (2022a) | Most parents agreed that the telehealth assessment was useful  Most parents agreed that accessing the telehealth assessment was easy to use  Most parents agreed that the telehealth assessment was effective  Most parents agreed that the telehealth assessment was reliable  Most parents agreed that the telehealth assessment was satisfactory |
| Talbott et al. (2022b) | I liked getting the box of toys and cue cards. It made me feel prepared going in and had a good understanding of how the appointment would be.  The researchers were both very professional and insightful, while creating a supportive environment.  I love that it’s Telehealth. We wouldn’t be able to participate in the study otherwise, since we don’t live nearby  I really enjoy having two meetings with the researchers, to allow for things to come up that I may have forgotten in a single session or account for unusual behavior/fussiness, etc.  it was very helpful for my child to be home, in his own environment. This made his behavior and reactions genuine and provided an accurate assessment of his abilities.  It was my first go around, but getting the camera angled and moving from area to area with the laptop was a bit challenging  There is a little bit more leg work for families at the on-set, but nothing that’s not manageable.  I think because we were in a familiar setting, my child was less engaged in the activities than he might have been in a different environment.  Seeing my baby NOT react to some of the scenarios or asks of the clinicians. That was hard to see as a parent, but it is the reality.  One caregiver reported that an hour-long session would be enough time, “My son doesn’t like sitting in a high chair for long periods of time so there were a few occasions during the eval where he became very fussy.”  Thirty-one of Thirty-two caregivers reported at least one response falling in the “benefits” category. The 5 themes falling under the Benefits category included: Convenience, Provided Materials, Clinician Rapport, Session Structure, and Representative Infant Behavior. The most commonly reported theme identified by more than half of caregivers related to the study provided materials. Caregivers appreciated the convenience of having all needed materials sent for the session, and described how this facilitated communication with the examiners, who could easily describe the items needed for each activity, saying, I liked that the clinician could easily tell us what toys to use since the box was provided in advance. Many caregivers also reported positive rapport with the examiners, despite never having met face-to-face. Finally, many caregivers described the benefits of being able to complete the sessions in their home, without needing to travel, and where they and their infants were most comfortable. For example, one mother noted, Taking the travel out of the equation is ideal. Especially for parents with children who have sensory issues. This aspect alone is why I would choose telehealth over in-person visits.  Twenty-seven caregivers reported at least one comment falling into “Challenges.” The 5 themes related to Challenges included: Technology, Logistics, Engaging Child, Observing Child Challenges, and Length. As expected, these reported challenges primarily related to the technological aspects of conducting the session over telehealth. Comments in this theme focused primarily on the need to manage camera angles and on the distraction various devices generated for infants. Under the structural theme, some caregivers described challenges related to the logistics necessitated by the telehealth format. This included taking the lead role in setting up and directing the interactions, and feeling as though communication could be more difficult over video than in person. Whereas two parents described the familiar home environment had been a benefit in gathering a representative sample of infants’ behavior, a handful of parents reported feeling the session had not optimally engaged their child, with one parent noting, It was somewhat challenging trying to engage my child in the activities in the same fashion as a clinician would (given their unique skillset and my lack of training in this area). I appreciated the suggestions provided over the [device] to help. At times, I worried if I was drawing the same skills out of my child as a trained clinician would be. Three parents noted it was emotionally difficult to observe their child struggle with some of the tasks presented. One parent shared, Seeing my baby NOT react to some of the scenarios or asks of the clinician [was challenging]. That was hard to see as a parent, but it is the reality.  Thirteen of thirty-two caregivers reported at least one comment falling into “suggestions.” The 4 themes falling under the Suggestions category included: Instructions, Technology, Preparation, and Support. About a quarter of caregivers offered a suggestion falling under the ‘instructions’ theme. Specific suggestions across these themes varied. In terms of Instructions, some caregivers suggested they would have liked a pre-visit video tutorial or a practice session, and one parent thought more explicit instructions for the open-ended play activity would be helpful in eliciting target behaviors. Some families noted a pre-session could have been helpful in identifying locations for filming or the addition of phone stands or the use of Bluetooth audio devices to limit distractions for their infants. Suggestions falling under the Preparation theme included allowing infants to play with session materials ahead of time to reduce their novelty or conducting a “pre-interview.” Suggestions in the Support theme focused on parents’ requests to connect the evaluations with future clinical services (parent coaching or local interventions). |
