# Supplementary material for: Families’ and clinicians’ experiences with telehealth assessments for autism: A mixed-methods systematic review
Source: PLOS Digit Health. 2025 Jul 29;4(7):e0000931. doi: 10.1371/journal.pdig.0000931 (PMC12306760; doi:10.1371/journal.pdig.0000931)
Supplement: S2 Data — (DOCX) [file pdig.0000931.s003.docx]

| Data on clinicians’ experiences and attitudes towards telehealth | |
| --- | --- |
| Study | Qualitative and “qualitized” data |
| Bain et al. (2021) | Forty percent (40%) of practitioners believed that it is possible to diagnose ASD in a first telehealth encounter  The vast majority of neurologists believed that is appropriate to diagnose ASD (amongst other neurological disorders) over telehealth for children under 5 years old, and virtually all of them (99%) deemed a telehealth diagnosis to be appropriate for children between 6 and 17 years old  Clinicians reported difficulties accessing a telehealth session for 89% of consultations, over the COVID-19 pandemic  40% of clinicians mentioned that parents were facing “economic” issues, such as not having access to video-recording phones and high-speed internet, which limited their ability to attend a virtual appointment  The vast majority of clinicians (88%) experienced technical difficulties  29% of clinicians mentioned that parents attending a neurological assessment via telehealth believed that assessments provided via telehealth are not as effective as in-person evaluations  A small minority of clinicians (9%) believed that parents face barriers accessing telehealth because of religious or cultural factors  A small minority of clinicians (7%) reported that the parents expressed concerns about security and privacy associated with telehealth  A big proportion of neurologists (85%) surveyed intended to use telehealth to assess for ASD, amongst other neurological conditions, after the COVID pandemic  More than half of the neurologists (58%) reported that interpreters attended a virtual appointment |
| Corona et al. (2024) | Children diagnosed with ASD following in-person assessment who were not identified on tele-assessment (ASD-No ASD group) also had statistically significantly lower scores on both the TAP and TELE-STAT and ADOS-2  Children with high adaptive skills might be missed in a telehealth evaluation  Children in the No ASD-ASD group were significantly younger (m=1.97, SD=0.11) than children in all other groups. That is, the three children were inaccurately identified as having ASD by tele-assessment |
| Esther et al. (2022) | **Convenience.** Staff members reported that some families appeared more comfortable with the telehealth appointments as they could remain at home and the reduction in hospital visits was perceived as convenient  The hybrid model was perceived to enable easier service access for families in rural and remote areas  **Flexibility.** Clinicians indicated that utilizing telehealth promotes flexibility in clinical practice, work location and use of time. Clinicians felt that they could more easily make appropriate arrangements to meet the needs of families: ‘People have been able to work from home to do the interviews and some feedbacks. Parents that aren't able to spend the whole day can join in for just important parts or relevant parts of the assessment’.  Some staff also commented that the hybrid model allowed them to work from home if they had slight physical symptoms, or were required to isolate, therefore allowing appointments to continue  **Improved clinical service.** Clinicians perceived that the use of telehealth increased the engagement of different stakeholders such as caseworkers and other healthcare professionals involved in the care of the child, school staff and parents: ‘It allows the dad to be more involved within the feedback session or if split families to be able to attend it, mum and dad in the same space to give feedback to both parents at the same time without being physically together. We have also had good success with foster parents and the caseworker online with us to give feedback. That was fantastic’.  Clinicians felt that they had the opportunity to gather more information after the initial telehealth consultation and therefore were better prepared for the upcoming face‐to‐face appointment with the families: ‘some of the clinicians, particularly the doctors who take the history like that they might have two weeks between history and the appointment to contact schools’. Comprehensive assessment and better communication between the children's care providers can be achieved  **Improved assessment skills.** Staff have noted that over time, there has been improvement to service delivery using the hybrid model of assessment. This has included staff realizing that they have adapted communication styles and assessment techniques when relating to families online, resulting in what has been perceived to be an increase in parent and child engagement during telehealth  **Technical issues.** Technology challenges from the hospital and family's end were reported to be a disadvantage of the hybrid delivery service model. Staff experienced different technical glitches when conducting telehealth appointments with families: ‘Often families were in poor internet connection areas. Our internet connection was quite poor as well’. They also reported that some families had bandwidth issues, ‘Even sometimes trying to get them on a mobile phone or a telephone could be difficult’  or that families were not financially able to upgrade their mobile data plan for more seamless telehealth appointments: parents often ran out of money and then they didn't have access to the phone’  **Challenges to engage with families.** Clinicians perceived that it is more difficult to get to know the child via telehealth, and that additional time is required to build rapport and trust with families when compared to face‐to‐face appointments. Reading body language and subtle behaviours of the child and family members was also more difficult with telehealth, resulting in challenges when building personal connection and providing emotional support: ‘I think we underestimate how much or how important that [rapport] is in building a relationship with our families and you know some of the gestures we use or the support we can offer them with a box of tissues or a warm word or stroke their arm or something. We can't do any of that to support them or even asking them questions and then becoming distressed so I miss that physical contact’.  Distractions within the home could also affect rapport: ‘Parents were often at home stuck with kids not at school so there were a lot of other distractions going on at home’  **Team cohesiveness.** Staff members reflected that since the hybrid model divides the assessment service into multiple appointments and team members may be connecting to appointments from different physical locations, there is an increase in disconnection amongst the group: ‘we check in with each other and [need] to find out what happened which we didn't have to do before’. Staff members reported that communication among the team has become more challenging under the hybrid service delivery model ‘now there is a lot more communication required from different members of the team’ (Esther 2022, clinicians).  **Longer waitlist and increased staff workload.** The only appointments where clinicians felt an increase in workload and time were those involving interpreters. However, the administrative staff's workload did increase due to managing multiple bookings and cancellations. The current electronic booking systems did not enable them to alter appointments easily, resulting in more time and effort for rescheduling  A small minority (12%) of clinicians reported experiencing technical difficulties on their side |
| Gibbs et al. (2021) | The opportunity to have an assessment within a familiar environment meant clients felt comfortable and relaxed at home “If they’re more used to online, or they feel more comfortable online, because it’s less threatening for them, it’s less anxiety-provoking for them. And so, I think it fits them better” (Clinician #3)  In addition to enabling assessments to be conducted during the pandemic, the clinicians thought the ability of tele-assessments to support regional participants or participants who need to travel long distances was advantageous. “I think particularly for regional families, families that are not close to a centre. It’s so easy. And that’s the feedback that I got from a lot of the families that we did telehealth, it just so happened that they were from regional areas. And at the end of the assessment all of them said this is preferable. Not having to travel. Most of them would usually stay overnight. The child is usually very dysregulated when they turn up to the centre and they’ve been in the car for two, three hours, so that can often be a problem. So this is a real advantage that you get to kind of see, see them when they’re more regulated.” (Clinician #1)  “I think what I really see as a big positive is the ability to reach families that would otherwise not be able to come to the clinic and get an assessment. They could be waiting for months and months and months, might require travel at some cost that not everybody can afford, so the child or client goes without. I think that’s probably the biggest plus I see, is the outreach ability, I think.” (Clinician #2)  Although clients, carers and clinicians shared mostly positive technology experiences, they also described some instances where technology negatively impacted on their assessment experience. All three stakeholder groups expressed concerns that some important aspects of client behaviour may not be as apparent to the clinician in a telehealth assessment. There was a perception from clients and carers that subtle behavioural characteristics that might be important for clinicians to observe may not have been apparent when their view was limited to what could be seen on the screen. “I guess it might be a little bit limiting, you know, as you can only see what’s on the screen  Similarly, clinicians described some challenges with the administration of assessment items via telehealth and not being able to accurately make judgements about behaviours of interest. Clinicians found that verbal and/or conversation skills were the easiest skills to assess, whereas all clinicians found that eye contact was the most difficult behaviour to observe via videoconferencing. “I think the hardest thing was the eye contact because I guess the client is not really engaging in eye contact at all. They’re looking at you on the screen, not in the camera. So that was challenging.” (Clinician #1) “Eye contact is a bit harder to guage, depending on the camera. Because, sometimes the child is actually looking at the screen, but the camera is elsewhere on the computer.” (Clinician #5) “Joint attention was quite difficult. It was something I had to ask the parents about a little bit when it came to that.” (Clinician #4)  The use of inappropriate technology such as using phones rather than computer/laptop/iPad negatively impacted the assessment experience of clients and clinicians. “I’ve had people use their phones rather than a computer. And it’s tough. I’m tiny on the screen…I don’t think it’s really very good if the only access they have is a phone…But it’s not, for me, not being able to see what’s happening. It’s more that the client isn’t really seeing you.. So maybe they’re not referring back to you.” (Clinician #2) “I’ve had one adult face-to-face assessment…for someone who was overseas, and this was the first case where they actually didn’t have the Zoom set up through like a laptop or iPad. They only had their mobile…So the major component of the assessment was the observation, but that was done through a mobile phone which is just really not a great quality observation. That was quite challenging.” (Clinician #4) “I had one parent trying to do it on a phone, that just doesn’t work. The screen is so tiny, the child can’t see. They have to have an iPad or a computer because if they’re a significantly hyperactive child, it doesn’t work because they’re not going to stay in the one spot in front of the phone. I think, first, they need to have appropriate technology, doing it on a phone is not appropriate for [behavioural observations]. The child, especially if they need to view pictures and things on the screen, it’s way too small.” (Clinician #6)  Using appropriate technology in an optimal manner was also very important, for example, even when clients used laptops/computers/iPads, issues with camera angles sometimes prevented clinicians from making accurate observations. “It’s just harder to sometimes see everything that’s going on. You may miss little bits and pieces, the camera is in a fixed location and the child’s moving around. Obviously depending on how far away they are from the screen, the audio might not be as strong. There’s those sort of factors.” (Clinician #4) “I guess the other challenge is more about what you’re able to see through the camera, so if there were ways that we could set up or have things that made it clearer in terms of seeing the room that they were in.” (Clinician #5)  In order to assess clients via telehealth, clinicians needed to adapt their usual assessment practices. The assessments conducted for younger children were perceived to be more difficult because they required more involvement from parents and parental coaching was needed. “Adults were definitely the easiest and then followed by older children with good language…I think it’s the age of the client as well as the tasks being able to be pretty much entirely done over telehealth.” (Clinician #2) “For older children and adults, I actually found it pretty similar to what we would do in face-to-face, so there wasn’t a lot of modifications or variations around that.” (Clinician #5) (Gibbs 2021, Clinicians).  For child assessments, clinicians talked about relying less on their own observations and needing to be more reliant on supplementary sources of information from clients’ parents, teachers, and doctors in order to reach a diagnostic conclusion. “I think the only thing that’s hard diagnostically when you’re looking at the autism criteria is…the non-verbal category. I found myself being more reliant on the parent information and information from other sources to kind of get a read on that. Everything else generally I found diagnostically I could gather.” (Clinician #1)  “I think I spoke a little bit earlier about with the younger children, if they were not particularly easy diagnostically, that over telehealth I felt like I could rely on my own observations a bit less than I would rely on my own observations if I had them face-to-face. I’d say, I think I could see that it could be a bit of a difficulty with some of those cases. I don’t think I would have the same concerns with older children or adults.” (Clinician #2)  “Probably the only thing is in those cases where I feel slightly less confident in my observations, then I have to rely on other information, maybe a little bit more than I would ordinarily.” (Clinician #7)  For child assessments, clinicians identified the importance of the level of preparation of parents/carers prior to the tele-assessment, as assessments tended to flow better when parents/carers were well prepared and organized. “I don’t think this is a typical experience because the parents were extremely prepared and proactive. But for them it was quite easy. She had organized each of the sections into tubs. All I would have to say is, we’re going to move to section now and she would be like, ok, no problem and then we’d get that stuff out. And I had both parents too, so that was really helpful. She was super organized. Yeah. So for me, I found that to be really helpful, but I can imagine that if I didn’t have a client that was as proactive that it would be problematic.” (Clinician #1) “I had a family that was completely unprepared. We managed to get through it, it was absolutely fine. Diagnostically, no problem. It was just, they weren’t very organized. Actually, I’ve done two now I think… It was still just more difficult.” (Clinician #2)  Clinicians reported that the parents’ abilities to be coached and understand instructions impacted the quality of child teleassessments and the degree of difficulty in conducting the assessments. “I think one of the challenges was having to remind parents not to coach the child…. sometimes parents would do things like directing the child more than you would like in an observational assessment.” (Clinician #4) “[some] parents didn’t really listen to your instruction and would go a few steps ahead or would point when you asked them not to point…some parents listen brilliantly. I’d only give short instructions at a time. Others, they’re those parents that have a tendency to take over. You get that in face-to-face as well though. But I guess you can control for more of the clinician-led activities when you’re in face-to-face. Whereas, sometimes the parents went a little bit rogue.” (Clinician #6) .  Based on clinician feedback, it seems that tele-assessments are most suitable for youth and adult clients. “I think it’s great for adults, and the older teenagers who won’t get out of bed or the ones who are more anxious. So, I think it helps them.” (Clinician #3)  Clinicians identified certain client types of clients for whom tele-assessments may not be suitable, including younger children with mild symptoms, children with ADHD and those with moderate or severe challenging behaviours. “I would say some of the kids that were quite hyperactive made it difficult because they wouldn’t stay in front of the camera or they’d go other places and you had to try and move around with them.” (Clinician #5) “I had another child and trying to get him to sit in front of the computer, it ended up taking us an hour and 45 min and it’s usually 45 min. Whereas, in the clinic that’s easier to control. You can have some toys there and give them a break and then use visual schedules.” (Clinician #6) . |
| Juárez et al. (2017) | Psychologists said they were ‘satisfied’ or ‘very satisfied’ with the remote screening technology in 80% of cases  Clinicians would prefer to see the patients in person in 24% of time  Clinicians would prefer to assess in person children who received a diagnosis of developmental delay, those presenting with complex presentations and history, and when technical problems were evident  Some technical difficulties associated with the audio (40%) and video quality (30%) were reported by clinicians, however those did not significantly impede the evaluation process |
| Kellom et al. (2023) | **Assessment** Participants commented on the experience of having a child assessed through telehealth, expressing different opinions. Most parents and providers agreed that the child being in their natural environment improved the physician’s ability to observe their usual behaviors  Providers noted they were able to see behaviors that they might only hear about as part of the history during an in-person visit, where children are often upset or uncomfortable  At the same time, participants (few providers and some caregivers) also questioned the accuracy of an assessment where the provider was not able to physically examine the patient  While all interviewed caregivers did have internet access, providers noted that families without reliable internet or with limited data plans face additional barriers to telehealth.  A few providers described that having to instruct the parent to elicit observations, instead of the provider directly doing so, made assessments more difficult to interpret. One provider reported, “I know how children respond to me when I interact with them. Knowing how they respond to the parent and what sorts of supports the parent is providing sometimes weren’t as clear.”  **Diagnosis** Regarding confidence with diagnoses, participants noted that certain diagnoses, whether assessments are completed in-person or virtually, are challenging to make. There were discrepancies, however, in which patients respondents perceived to be more difficult to assess by telehealth  Some providers reported that it was harder to assess older, more verbal children and those whose features of ASD were less prominent. Overall, though, providers believed that if they were able to observe the child on camera, they could be confident in their assessment  Both stakeholder groups discussed additional elements that are likely to affect parents’ acceptance of diagnoses received over telehealth. Some providers believed that certain ways they adapted to the telehealth environment facilitated parents’ understanding of the assessment process and acceptance of the diagnosis. For example, by explaining to parents what they were looking for, by sharing their screen with diagnostic criteria or as they were instructing families on how to elicit and interpret a prompt, parents were more engaged in the process and could see the results for themselves. Both groups acknowledged that caregivers’ acceptance of and reaction to the diagnosis depended partially on how emotionally prepared they were to hear that diagnosis. .  Providers noted that some parents joined the follow-up visit from work and appeared to struggle more when receiving the diagnosis in a less private space  **Rapport** Some providers believed that establishing rapport was more difficult over telehealth, citing that it was harder to convey empathy and pick up on interpersonal cues virtually than when physically sharing space  Providers and parents alike noted that technical issues were the most significant barrier to developing rapport and engaging in the virtual visit. Members of both stakeholder groups who experienced visits in which a visual was not available (e.g., telephonic visits or video visits in which the camera did not work) expressed concerns about their ability to engage emotionally  **Access** The overwhelming sentiment from participants was that remote visits improve access to care and decrease costs for families. Caregivers and providers recounting family experiences described the benefits of having less time away from work or school; not having to worry about transportation, parking, and their associated costs; not having to find child care for siblings;  enabling multiple caregivers to join from different locations  and mitigation of infection-related concerns during the pandemic.  Participants highlighted that travel-related benefits may have been most impactful for families who are geographically distant from DBP providers and likely experience more travel burdens and families who have anxiety related to travel to a medical office.  **Efficiency of Care** Overall, providers and caregivers perceived an increase or no change in the efficiency of DBP care by telehealth. Survey data from providers demonstrated that the majority (n 5 11, 84.6%) believed their efficiency was better or unchanged compared with in-person care. Most surveyed providers reported that the face-to-face time allotted for both in-person and telehealth new-patient visits was the same (n 5 8, 61.5%) and sufficient (n 5 9, 69.2%). Time allotted for follow-up visits was also equivalent (n 5 9, 69.2%) and sufficient (n 5 11, 84.6%). The amount of time spent outside of direct patient care for follow-up visits was the same or less (n 5 11, 84.6%).  In interviews, providers reported telehealth visits being more efficient, with fewer issues related to clinic flow. Most providers believed time needed for documentation was less than or similar to what was required during in-person care. A minority of providers believed that telehealth visits were less efficient, citing feeling less control over visit flow, more time required helping families navigate technology or eliciting particular behaviors, and more time to complete aftervisit documentation until they became comfortable typing while making observations by video.  Providers and caregivers named the usability of the telehealth platform as a frequent barrier to access. Similarly, providers described having to reschedule or schedule a follow-up session to get needed information when families encountered technical difficulties.  **Family Characteristics and Equity in Telehealth** As previously stated, our sample of caregivers did not personally experience the barriers discussed in the previous section. Therefore, we focus here on the insights of providers who spoke from their experiences serving various families. Providers described that technical issues with the telehealth platform and navigation were challenging for families with PLOE.  Providers described how practices struggled to integrate interpreters into telehealth visits for families with PLOE, negatively affecting access. While systems and telehealth technologies were eventually updated to meet this need, intermediate solutions detracted from the clarity and quality of encounters.  Providers felt more limited in their ability to establish rapport with, accurately observe, and diagnose a child who does not speak English  Providers’ survey responses similarly demonstrated higher levels of confidence diagnosing ASD in children from English-speaking families and less confidence diagnosing ASD by telehealth for children from Spanish-speaking families.  In addition to language needs, providers shared perceptions that certain family characteristics may make telehealth less comfortable or feasible. Providers noted that families experiencing housing insecurity; without a private or quiet space; who have multiple children, older caregivers, or fewer adults at home to help manage the visit made it harder or less likely for a caregiver to participate in telehealth. Providers reflected that many of these demographic issues likely correlate with socioeconomic status and expressed awareness that telehealth is not likely to expand access to care universally.  **Telehealth Postpandemic** In surveys, providers indicated their preference for how to deliver each component of DBP care. Surveyed providers agreed that in-person care would be preferable for testing/developmental assessment and physical examination.  The majority believed that telehealth was preferable for history-taking (84.6%), feedback (53.8%), and follow-up care (84.6%).  Similarly, in interviews, participants suggested telehealth was a beneficial option for follow-up visits when the child might not need to be present and to discuss quick questions without requiring travel  Given the technological challenges many families faced, participants expressed a need for standardized, user-friendly platforms with options for multiple parties (e.g., another caregiver or an interpreter) to join the video call seamlessly.  Both stakeholder groups suggested that families needed to be adequately prepared for the technological aspects of joining and the emotional aspects of conversations about diagnoses  Balancing the value of home environment and not having a physical examination. People expressed views across the spectrum, with most suggesting that having the child in their usual setting improved the physician’s ability to observe usual behaviors and others questioning the accuracy of an assessment where the provider was not able to physically assess the patient and mediated by a device that for some children is distracting  “Another benefit from—especially for that age group, so under 5, especially, developmentally kids under 3, so when we’re assessing them through telehealth, it’s us giving the parent prompts of things to do with the child and then we get to observe. And so it’s very valuable to see how the child is responding to the parent with these prompts, and it also gives the parent insight into, oh, yeah, they really are doing these things or they’re really not doing these things”  Providers noted that certain diagnoses are going to be hard no matter what. “I feel like the reaction varies regardless of whether it’s in person or telehealth just because sometimes [the diagnosis is] unexpected. So I feel like the unexpected is what’s the harder part as opposed to it being that it’s via telehealth.”  *S*ome providers and parents felt like something was lost by not being in the office. “There are still a lot of things that we can do and just see and get from parent rapport that can tell us, okay, well, they’re developmentally high enough, or their developmental level is, at least at this age, which is telling me they should still be able to respond to their name, or point or participate in some back and forth, or do some pretend play. We can still get a lot of that, it’s just not quite the same as actually being able to sit there and do the testing with the kid.”  Diagnosis and buy-in. Some providers believed that telehealth enabled parents to be engaged in the assessment and understand better what the provider was observing. “It’s very valuable to see how the child is responding to the parent with these prompts, and it also gives the parent insight into, oh, yeah, they really are doing these things or they’re really not doing these things.” .  Assessing parents’ expectations and appropriately preparing them for a telehealth visit to discuss diagnosis was highlighted. “I’ve had parents who were on their break at work who started to cry and you’re like how is the parent going to manage this situation when they have to go back to work in 15 minutes and you’ve just given them this devastating news about their child? I’ve had situations where it wasn’t clear that the parent was really focusing on what I was saying; they were clearly distracted by other things that were going on  Establishing rapport with families “I think that the other cool part about [telehealth] that I have found is, that it’s helped me to explain to parents what I’m looking for, and what I am trying to see. And so I have to direct them to do things, right? Like, I have to say, okay, I want you to call your child’s name, and I want to see if they look at you. Just things that I would just do instinctively without saying out loud to the parents during my evaluation. But over Telehealth, I needed them to help me do it. And I think that has actually been really helpful for the parents to see, like, hey, this is actually what she’s looking for, for me to like put it into words as opposed to, hey, all of a sudden I’m diagnosing your kid with autism and maybe the parent feels like it’s out of the blue.”  Remote visits are easy and decrease the cost of care for families. All different types of families really did well with [remote visits]. But over time, what I saw is, that those families, there were more barriers to care. So scheduling multiple siblings, transportation issues, those families of course, were just so much more grateful and able to actually connect with us, versus some families that really did need an evaluation, but would have been lost because of the logistics of it. So yeah, it tends to be families with greater needs, like physically, like I mentioned, transportation or scheduling is really hard. Or limitations, limitations with making the phone call and following up on the phone call and making sure that they’re scheduled and coming to the right building. I definitely saw over time, that I was seeing a greater portion of families who I think would have had a delay to get care, who were able to come in through Telehealth more successfully.”  Certain family characteristics make telehealth particularly beneficial for access, such as their geographic distance from DBP care. “I mean obviously it is an advantage for some families who might have to travel a long distance to get to the hospital, it can make care easier for those families. I mean you didn’t ask me about ways in which it could benefit, but some parents who have trouble with childcare and things like that. There are barriers to in-person care that telehealth can minimize.” (Kellom 2023, Clinicians)  Given some of the behavioral concerns of children presenting for DBP care, there are some unique benefits of telehealth for the patients and their families. “I’m observing the child in their home, and they’re often much more behaviorally, like calm, because they’re in their own setting and I can actually observe them at a higher level of their functioning. So those follow up visits tend to go much better. And so it will not only increase access of care, but then also continuity of care, I think, because so many of my families, they don’t want to come out to a follow up visit, because they know their child has challenges going into new places or unfamiliar settings. So it’s a big burden on families, when the child has autism, often, have sensory overload issues or something like that. So yeah, I do expect it would really help with equity.”  Telehealth seemed to facilitate appropriate triage for how different types of concerns were managed, which likely resulted in more families’ concerns being addressed more efficiently“. One other benefit to it is families will call our triage line or send a [MyChart] message if they’re having some urgent concerns —their child with autism is having worsening behaviors, they’re really struggling with something. And so prior to telehealth we would call the family or send email messages back and forth with them, and none of that is reimbursed by insurance. That’s all just been our kind of administrative reimbursable time. Now what we can do is say, why don’t we set up a video visit, we can talk about this more in depth. It’s a little more efficient to just do it that way instead of all back and forth and then it’s actually reimbursable."  The usability of the telehealth platform was cited by some as a potential barrier to access, particularly for families with PLOE because directions and platforms tended to be available only in English. “I think definitely access to telehealth is more limited for families with limited English proficiency, just because of the amount of bureaucracy. Just getting the app, signing this consent form, signing that consent form. But I don’t think our app has a lot of Spanish translation on it, so I think all of that is—it’s all a barrier for families with limited English proficiency to be able to then access telehealth because of the technology required to access our telehealth."  Familiarity with and access to technology frequently came up as a barrier to access that participants noted affected some families more than others. “I think for families who just aren’t— who are less comfortable with the technology or don’t have the technology or are concerned about the interpreters virtually– then that might affect their care potentially, but we have alternatives. So if a family doesn’t want it, then we have in person.”  Certain family characteristics may make telehealth less comfortable or feasible, including families experiencing housing insecurity or who do not have a private/quiet space. “If there’s a lot of interruption in the household, sometimes that can be disruptive to the visit. If there are a lot of kids, or too many adults, other things going on and it’s a little chaotic, sometimes it’s harder to hear. So, just—it just makes it more challenging to kind of get the information from them.”  Families with preferred language other than English (PLOE) had unique barriers in the telehealth setting related to needing to use interpreters that affected access and the clarity/quality of the encounter. “It makes me three times more nervous when the interpreter talks so much more than I do. And I’ll say something very short, and there will be a very long conversation before they come back. I’m always very worried that some question that I just asked is completely inappropriate or something. And there’s no— sometimes after a visit, I would always talk to the interpreter so that I could understand better if I said something that culturally didn’t land appropriately. Now, I’ll get off. The interpreter gets off actually before you do usually. So I never get a chance to talk to them”  Participants described ways in which scheduling appointments were improved with telehealth options. Providers, not needing to commute, found it easier to add follow-up patients onto their schedules for shorter visits to address specific concerns.  Both stakeholder groups described how the addition of telehealth could be beneficial in meeting family needs.  The vast majority of clinicians (92.3%) were satisfied with the telehealth procedure to assess children  All clinicians reported that they would be willing to use telehealth again in the future  Around half of the clinicians believed that telehealth was more efficient compared to in-person assessment at delivering comprehensive care, while 30% of those believed that the modalities have similar efficiency  The majority of clinicians (92.3%) felt comfortable diagnosing ASD in English-speaking patients  While the majority of clinicians (61.6%) felt comfortable diagnosing ASD in Spanish-speaking patients, 30.8% of them were not comfortable  In most cases (53.8%) clinicians reported that telehealth did not affect the payments received for patient encounters, while some of them (30.8%) were not sure  The vast majority of clinicians (84.6%) would prefer Telehealth over in-person visit for History-taking  All clinicians surveyed would prefer to perform any testing or developmental assessment in person  In terms of providing feedback, slightly more than half of the clinicians mentioned that they would prefer telehealth, whilst the remaining 46.2% would prefer to do that in person  The vast majority of clinicians (84.6%) would prefer Telehealth over in-person visit for follow-up assessment |
| Kryszak et al. (2022) | Most clinicians interviewed agreed that telehealth worked well enough or was even preferred for clinical interviews and feedback.  However, there were mixed responses related to clinician confidence in using telehealth to conduct behavior observations needed to make a final diagnosis. This section outlines the strengths and weaknesses shared regarding virtual behavior observations, as well as the clinical presentations and child/family factors that lead to variance in clinician confidence. Difficulties related to completing other standardized testing by telehealth are also reviewed.  Clinician confidence level—Confidence in accuracy of diagnoses made by telehealth assessment varied significantly from very confident in many cases to not at all confident. Many clinicians felt that, in most cases, their observation of the child’s behavior was sufficient to make an accurate diagnosis. As one clinician noted: “Now, I feel really confident that the data I get is reliable and supportive of an appropriate diagnosis.” A minority of clinicians noted that they did not feel comfortable making any final diagnosis by telehealth or expressed strong reservations about the diagnoses being made: “we had not done telehealth at all. So to take something that we were used to in person and shift it to telehealth…was really uncomfortable for me.”  Strengths and weakness of observation by telehealth— The primary identified strength of completing an observation by telehealth appeared to be the opportunity to see children and families in their home environment. This allowed for a more natural observation, getting to see children play with toys they most typically engaged with around those with whom they most typically interact.  Some clinicians also stated that parent participation in telehealth assessment provided an opportunity for parents to see their child’s behavior from a different perspective, which was beneficial to parent engagement in the evaluation process: “I really liked what that added to the parent’s role in the evaluation. I think having the parent do some of those presses allowed the parent to see their child’s interactions from another perspective at times, could buy in differently because of their role in that and as well as just provided also some opportunities to kind of touch into things that could be later brought up in intervention.”  One clinician mentioned, “I actually think, with some of the little kids, we’re getting a better picture than we would have gotten when they come into clinic.” Clinicians reported that this was particularly true for children who present with significant anxiety or behavior that was inhibited for other reasons in a clinic setting.  The major concern reported was not getting the same quality or reliability of observation when it was completed by telehealth. Clinicians often contrasted the various observation protocols they were currently using with the Autism Diagnostic Observation Schedule (ADOS; Lord et al., 2012), which is considered to be a gold standard tool for assessing ASD symptoms. They often stated that they missed the consistency of opportunities for observation presented by the standardized and controlled administration of the ADOS, contrasting this with the opportunity by video for a more varied and naturalistic observation in the home. There was also concern that social behavior may be just different when interacting by video and we do not yet know how to best interpret this.  Clinical presentations and child/family factors that affected confidence—Clinicians outlined different clinical presentations that affected confidence in diagnosis. In general, symptom severity, age, and developmental impairment impacted the ability to make ASD diagnostic decisions by telehealth. More specifically, younger children and children who were more developmentally impaired were easier to assess, whereas older children with more subtle impairment were more difficult to assess over video conferencing.  There were also child and family characteristics that could make it challenging to conduct a satisfactory virtual behaviour observation. These included children with high activity levels where it was difficult to keep them in the camera view, children who actively refused to interact over video, parent/ child interaction difficulties which affected the child’s cooperation, highly anxious children who were inhibited by being on camera, and children with variable social skills where telehealth made it difficult to notice more nuanced deficits.  While not directly related to making an ASD diagnosis, it was noted that certain safety issues are difficult to assess for or address by telehealth such as suicidal ideation and behavior, abuse concerns, and significant aggression.  Finally, it was noted that clinicians felt less confident making a diagnosis solely through telehealth assessment when families were more resistant to the diagnosis due to this modality being less widely accepted. (Kryszak 2022, Clinicians)  Building and introducing telehealth models of care— To develop telehealth models, many sought out trainings and webinars in both general telehealth practice and on specific telehealth assessment measures, while others consulted with partner agencies. Many clinicians reported feeling high levels of initial doubt and anxiety until they actually tried assessment by telehealth. This experience led most who attempted telehealth assessment to realize that this modality was not so different from their in-clinic practice.  It was also noted that working as part of a team to problem solve difficulties regarding virtual behavior observation and being able to observe peers first improved confidence  Having experience with any sort of parent coaching protocol (e.g., Parent Child Interaction Therapy) in clinic or by telehealth before the pandemic helped a few clinicians feel more confident in directing parents through a telehealth observation.  Comfort with learning new technology was also a factor that some clinicians noted as playing a role in how quickly they adapted.  Other testing by telehealth—Only a few sites attempted to complete standardized, direct assessment with children via telehealth (e.g., cognitive, developmental, speech and language, etc.). Those that did conduct cognitive testing reported being able to collect good information, but that there were limitations, such as not being able to manage behavior effectively, having to rely more on parents for behavior management without interfering with accurate administration, and challenges in maintaining children’s attention over longer testing sessions. Tests available by telehealth were also limited to children age 6 and older and generally with milder cognitive impairments.  Clinicians reported missing having direct testing results of cognitive and developmental level to add additional context to the diagnostic picture.  Spending more time tracking down information from additional sources, such as school reports or outside evaluations, was stated to be helpful in increasing diagnostic confidence, although this information was often more difficult to access during the pandemic as school and other services were also remote.  Establishing Rapport—Establishing rapport was noted to be somewhat different over telehealth, but the majority of clinicians indicated that establishing rapport was far less difficult than they were expecting, especially with parents and caregivers. Building rapport with children was more variable. A few clinicians found establishing rapport over telehealth more challenging, particularly when video conferencing was not possible and only audio conferencing was used.  It was acknowledged that completing a diagnostic assessment by telehealth was not ideal for everyone. Most sites had a plan for bringing patients in clinic for an assessment when symptoms were less clear, or a good observation could not be made by telehealth  Several clinicians, however, stated that few patients needed to come back for an in-clinic appointment, as they were able to confidently make a final diagnosis in the majority of cases. One clinician commented “There was like 5% of kids where I was like, ‘I think we should not [make a telehealth diagnosis].” Multiple clinicians made similar estimates that between 80 and 95% of children who were seen were able to be diagnosed by telehealth assessment. Clinicians also noticed that these patients continued to be difficult to diagnose even at an in-clinic appointment. In other words, “A clear kid is a clear kid, and they're clear in the office and they were clear on video. And the tricky kid is a tricky kid, and they're tricky in the office and they're tricky on video.”  While many clinicians were feeling confident in the assessment activities being completed by telehealth, it is also important to understand the possible impact of patient and family factors on telehealth services. The following section outlines provider perception of families' satisfaction with and willingness to try assessment by telehealth, possible barriers to accessing telehealth services, and possible benefits to telehealth services for families served. Possible benefits and costs of completing services by telehealth to those experiencing health disparities and for non-native English speakers are also outlined.  General family willingness and satisfaction—Clinicians reported that many families were very satisfied with their telehealth assessment experience. Providers reported that families were often initially sceptical about telehealth evaluations but were pleasantly surprised with how they went. Often times some pre-work (e.g., a phone call or letter sent before the evaluation appointment) was needed to help families understand how an evaluation could be completed by telehealth. Even with this pre-work, some families refused to do the evaluation by telehealth preferring to wait for an in-clinic evaluation. There was not necessarily a clear pattern observed as to who refused to do telehealth, although concerns about the accuracy of telehealth evaluation were often reported.  Possible Barriers to telehealth—Clinicians noted that higher socioeconomic status (SES) families with greater access to and understanding of telehealth had the easiest time completing virtual evaluations.  Providers reported that one of the biggest potential barriers to effective telehealth for any given family is having adequate technology. Not having appropriate hardware or sufficiently strong internet connection or difficulty using technology were the biggest barriers to families not being able to participate in telehealth assessments, although providers noted that fewer families had technology issues than expected.  Another potential barrier was identifying a safe and confidential place to complete a telehealth assessment. Without the clear boundaries of an office, some families attempted to do appointments in a space that was inadequate for the assessment or would not provide appropriate confidentiality (in a moving car, at a store, etc.).  Benefits to families of having a telehealth option—Several possible benefits were noted for families including overcoming transportation barriers (e.g., more access to families living in rural areas; easier access for those without a car), saving time and money (e.g., less time missed from work or spent traveling to appointments; no need to hire child care), and flexibility with where families could do the appointment (e.g., their office or car when on break from work). One clinician commented, “It's hard to transport these kids to appointments, take time of work, do different things. And so then being able to access us and these appointments easier has actually been a really good thing for families.”  It was also noted that telehealth allowed families to complete what is often a very stressful experience from the comfort of their own homes.  Using video conferencing allowed for additional family members and other care providers to attend evaluations and feedbacks when they might not otherwise be able to do so (e.g., family members who may not be able to travel or take of work; parents who are co-parenting; therapists, caseworkers, school personnel, etc.).  Factors related to general health disparities—Several clinicians indicated some hesitancy in commenting on how the pandemic and telehealth may have specifically affected health disparities due to lack of data, especially related to who was not seen. Many clinicians reported general concerns about health disparities that did not change whether patients were in clinic or being seen by telehealth.  Telehealth was noted to be likely to decrease health disparities for some families due to overcoming transportation barriers and decreasing other barriers, such as time needed off of work or accessing additional childcare.  The most often cited barrier to accessing telehealth related to disparities was lack of access to technology and/or internet. Other possible barriers noted included more chaos in the house, more children around to distract, and fewer caregivers to help with environmental management and camera work.  Clinicians also reported that telehealth allowed greater insight into families’ home environment that could allow for more individualized treatment recommendations.  Access concerns for non-native-English speaking families—Almost all respondents voiced concerns for nonnative-English speaking families having difficulties accessing services both by telehealth and in person during COVID. A few cited data they had that showed an actual decrease in non-native-English speaking families attending appointments or choosing to wait for in clinic appointments at a higher rate. Most centers appeared to continue to have access to interpreters by telehealth. Many indicated that doing telehealth assessment with an interpreter was difficult, although similar to difficulties seen in person (e.g., increased time for appointments; difficulties translating some concepts).  Coaching a family through a play-based behavior observation by telehealth through an interpreter was noted to be a particularly challenging activity. Respondents from sites with telephone-only interpreters tended to indicate more dissatisfaction than those with access to interpreters with video. A few clinicians noted that it was easier to talk with the family and the interpreter at the same time by telehealth due to how the video platform was set up.  Most clinicians reported that their sites had done little to no work by telehealth before the pandemic. Institutional support was identified as a large factor in how successful a site was in transitioning to telehealth. The significant pivot that most sites made to transition their clinical work to telehealth also had significant effects on efficiency and workflow, rate of no-shows and cancelations, and workplace satisfaction.  Institutional support—Clinicians varied widely on how much support they felt they received from their institutions to transition to telehealth services. A number of factors played a role in whether or not clinicians felt supported including leadership and support for innovating telehealth systems of care, level of pressure to maintain productivity, and institutional infrastructure needed for telehealth. Other important factors included clear communication from leadership, as well as support to help alleviate the pressures that the pandemic put on clinicians in their lives outside of work. Good administrative and technological support was also cited as key to helping families effectively access telehealth platforms and technology and feel comfortable with scheduling assessments by telehealth.  Efficiency—Some clinicians noted that there was a significant decrease in efficiency at the beginning of the pandemic due to having to start many new processes at once. As the pandemic went on, some clinicians noted that telehealth increased overall efficiency by decreasing time taken for certain aspects of the process, particularly the interview, checking in and rooming processes, and how clinicians could schedule their time.  Additionally, it was noted that telehealth and working remotely could help with space issues, such as not having enough offices or exam rooms to see families in clinic.  Telehealth also allowed for greater ease in typing during the clinical appointment, which decreased documentation time outside of the appointment.  Others noted that the telehealth model led to a less efficient workflow, with more time needed for preparing for the evaluation, completing assessment tasks, and documentation after the evaluation. Some models also used personnel less efficiently, as more parts of the evaluation needed to be done back to-back rather than simultaneously or it was deemed important to have multiple people watch the behavior observation when completed remotely.  A few reported having additional administrative responsibilities that made their workflow less efficient, such as having to schedule their own appointments, as administrative staff were occupied assisting families in connecting to the telehealth platforms.  Effects on no shows—Most clinicians noticed a decrease in families not showing up to appointments, often attributed to two factors. First, telehealth was easier to access and clinicians could often call to remind families who forgot in the moment and then immediately start the visit. One clinician stated, “I feel like the rate of no-shows have gone down, because it's really easy to just be like, ‘Oh. I'll just click this button.’ Or, ‘I'll just answer my phone.’” Second, because this process was new, appointments were often being scheduled closer to their actual date and there tended to be more contact with families to help them understand the new processes involved in telehealth. Some clinicians noticed an initial decrease in no show rates, but then a levelling out as the pandemic went on, while others noticed no change in no-show rates.  Changes to workplace satisfaction related to remote and telehealth work—Some observed experiencing an increased level of work-related stress, particularly in the beginning of the pandemic, given the uncertainty, lack of preparedness, and novelty of teleassessment. Increased stress was also related to feeling isolated and having more difficulty connecting with colleagues, having less opportunity for vacation/breaks, omnipresence of video access and increased fatigue related to increased screen time, and an increase in stress at home (e.g., managing own children doing school at home). One clinician commented, “I think it’s also socially isolating for the provider. In the middle of the telehealth time, it was kind of weird going all day without seeing humans in person.” Telehealth services also meant that nearly all clinicians were working from home.  Many expressed increased satisfaction from working at home based on several factors including not having to commute, more flexibility during the work day to balance work and other domestic duties such as household chores and child care, and getting to work in a more comfortable environment.  High satisfaction “But clinically, I've been really pleasantly surprised, and I found it rewarding and enjoyable. And it's been a good challenge. It's kept us all on our toes and had the opportunity now to train other people in virtual assessments, interns and people in supervised practice. And I've enjoyed that. I've enjoyed working with them.” “How satisfied am I with the actual clinical work? I think we're doing a good job, and I think we're doing right by families. And so that leaves me very satisfied.” “I think that the other psychologists, at least in my center, are really satisfied with it. I think overall we really like it, and we really hope that a lot of pieces of it are going to stick around.” “I like them. Honestly, I feel like I've become a bit of a—I've become a bit of a convert and a bit of the champion of the why do we want to do this virtually.”  Mixed satisfaction “I think we are all fairly satisfied, more so than we expected” “It's really hard to say…it's hard to give you just a binary answer like I'm really satisfied versus not. It's really in that range because there are some definite pros and cons.” (Kryszak 2022, Clinicians)  Low satisfaction “We are doing some video assessments, but I have not liked those… There have just been a few where I've done it by video and felt confident in the result” “And I think maybe I started to get resistant to that. I don't think it was a conscious, "I'm not learning this." But it's like, I don't want to get good at this. If this is the new wave of mental health, then I don't want to be part of the new wave of mental health  Level of satisfaction was related to numerous factors. Clinicians who found new challenges rewarding and enjoyable, who felt as though they had support from their colleagues and leadership, and those who had access to strong technological infrastructure tended to be more satisfied with the transition to telehealth. During their interview, one clinician commented, “I think we are doing a good job, and I think we are doing right by families, and so that leaves me very satisfied.”  Clinician confidence Higher confidence “And now, I feel really confident that the data I get is reliable and supportive of an appropriate diagnosis.” "I think, in general, for me, I think what I'm doing is working well, so most of the time, I feel like I can do a good valid assessment and that that works well through telehealth."  Lower confidence “I mean, we're definitely recommending that all these kids need revaluation…Everything is an estimate right now. But there's certain kids where we feel very comfortable with the estimate.” “We had not done telehealth at all. So to take something that we were so used to in-person and just shifted to telehealth with measures like the tele-ASD-peds that was even still under research was really uncomfortable for me.”  Strengths “But I feel like I'm getting a really good sample of the behaviours that I want to get” “So clinically, I feel like I am so much more informed when I'm watching children playing with their own toys in their own home as opposed to come into my office and getting mad because we tried to check their blood pressure, and getting frustrated with the traffic or sensing their parents stressed about getting to the appointment…” “So I think, with some of these kids, we're actually getting like a truer picture of who they are than we would if we saw them.” “And I actually think, with some of the little kids, we're getting a better picture than we would have gotten when they come into clinic because so often, when they come into clinic, it's a situation where parents are like, ‘They're really inhibited. They're not talking as much.’” “I really liked what that added to the parent’s role in the evaluation. I think having the parent do some of those presses allowed the parent to see their child’s interactions from another perspective at times, could buy in differently because of their role in that and as well as just provided also some opportunities to kind of touch into things that could be later brought up in intervention”  Weaknesses “The thing that I'm not comfortable with is the confidence that I have that I'm getting the same quality of information from behavioral observations that I have in the past.” “Again, it gives me something I can score, that I can show to a family. It gives me some activities that I can use to structure a remote visit. I don't feel they are as rich as the ADOS in terms of helping diagnosis … ADOS usually gives me a more secure feeling about the impressions I develop.” “Compared to much of the rest of how we do remotely, I don't think that the remote ADOS is– it didn't compare. The in person ADOS gleans much more reliable information, in my opinion.” “Because they don't have those benchmarks, it was harder to know, well, is this really autism or is it just normal Zoom behavior or Zoom etiquette for a three-year-old.”  Clinical presentations and child/family factors affecting confidence “Yeah, I think everybody's starting to feel like, look, you know, when I see video of kids, and I see them live and face to face, I can see them in the background stimming during a telehealth visit and they're not talking and they're not making eye contact, I kind of feel like, for somebody who's seen kids with autism, I'm not so sure I need an ADOS to tell me that this kid has autism.”  “There are a few kids where it’s impossible to do the evaluation because they can’t sit still.”  “I think what's hard is– eye contact is hard, but you can tell they get it. You can see if they're making eye contact with their parents. You just can't see if it's subtle, where they're maybe looking at your mouth instead of your eyes.  And things like their– you don't always get their sensory stuff because their house environment might not elicit some of their sensory issues, whereas, some of the toys did.”  “The one’s who I struggle with, we struggle with as a team, are the higher functioning children, like they’re five, and their verbal. Because then I want to see their face. I want to be right in front of them to catch the more subtle errors and omissions, and I’m not seeing it on video.”  “We haven't seen a huge increase in physical abuse, but we also aren't seeing these kids in-person so we can't be totally certain…So that's been something we've talked about as a division that we're at least somewhat worried about but don't know what to do about it.”  “I will say I identified potential sexual abuse in one of my patients, and that did not go– that was hard. That was damn near impossible because I couldn't see who was in her room– I couldn't see who was there, but it had to be done.”  “So I guess with teenagers, I worry about suicidality, and I worry that I won't get– I worry that the parent is listening in when I'm talking to them via telehealth.”  “The only time I didn't like it is when you had parents who were skeptical of the process. So if your confident in a diagnosis based on what a parent had reported and what you had seen, and then they're questioning, "Is this a legitimate way to get a developmental diagnosis?" Is this evaluation as robust as it needs to be? And I get that, for a diagnosis that you will have for life.”  “Those were the only visits that I was not overly comfortable in, when you felt like you were getting a lot of pushback from a parent. And the only thing I can say is, "This is just what I'm seeing. I'm happy to evaluate in person, and we can defer any diagnoses or anything until that time," and that's all you can do. So, I think my comfort level was at a good point unless a patient was not.”  Building and introducing telehealth models of care “And I think when this first hit, we were all trying to put feelers out and get a sense of what the autism community as a whole was doing so that we weren't just a solo project over here doing our own thing, and trying to get a feel for what everybody else was doing, what was going to kind of become the standard during COVID times, and trying to have some consistency across other sites as well.”  “I think trying it. Honestly, people were a bit reluctant, like how is this going to work? Am I even going to see what I need to see? And then I think– so trying it and that kind of peer support– we had lots of meetings quite frequently about like, how's it going for you? I've done this. What works? And just sharing ideas.”  “I'm learning through my colleagues because they are very good at it. And it's like, "Oh. I'm going to watch you." And now, I can do it on my own.”  “I think it's all dependent on how comfortable the professional is with, frankly, using a computer and being worried that they're going to break something.” “So I had the ability to draw on the experience where I've asked– I've been coaching parents to do things. And I think, for lots of people, that's the part that's really difficult, is being directive with parents.”  Other testing by telehealth “And then, yeah, the really oppositional kids that don’t want to do anything, it’s easier when we’re in-person to give them reasons why they might want to.”  “And they have been trying to do three hours of remote testing in a block[…]And I've watched, and the kids are like, "Ugh." They hate it. So I've been trying to get them to be able to break it up. […] the kids are doing the best they can, but they haven't even tried the big kids with autism yet.”  “We use subtests from the WISC, and you can get a nonmotor kind of IQ score. And so I think the tricky thing there, again, is getting a child to sit and attend to a computer in their home. You want the parent nearby, but you don't want them giving hints or help or adding to your instructions or changing your instructions. And sometimes, that can be tough to navigate.” “Personally, I think that I would really value doing cognitive assessments again.”  “So I think the drawback is not being able to do some direct assessment to get some standardized scores for kids.”  “I felt more confident with some of them in potentially doing telehealth if we had additional information.”  “It was really hard to get vetting information from another caretaker or therapist or anything like that because mostly everyone was virtual as well.  Establishing rapport “I feel like I can do it. I think it goes okay with families. I think getting the kid to sit there and engage remains harder.” “So I think when it’s a video appointment, it has gone pretty well. It’s not ideal. It’s not the same as having them in person. But I have felt that was pretty manageable.” “Initially I was like, ‘Oh we’re not going to be able to establish rapport and whatever.’ That sort of fear went away quite quickly. I feel like you could truly establish rapport this way. I really do. And I’ve seen it over and over again.”  General family willingness and satisfaction “And so far from the families, they seem to be satisfied with it.” “I think a lot of parents are satisfied with it. […] so many of the families feel like we've gotten an accurate picture of their child. […] they feel that it's been an accurate assessment and a timely assessment.” “I mean, there are the parents who really only want the in-person […] But in general, I think it worked for more than it didn't.”  “And I've had some highly-educated, well-resourced families that will just say, “No, we want to come in person.” That's the experience that they need as part of what they feel is medical care.”  Barriers to telehealth “So just the normal demographics that you'd expect, right? The ones that had more resources and better stable internet connections had an easier time with telemedicine.”  “But I would say that would be the other big barrier is that there were a small, but certainly significant group of people that either couldn’t use the technology or the technology wasn’t available.” “Then you have the technology barriers, where some parents are tech-savvy, and some are not. They find it intimidating and some have refused to use zoom, even though we set it up for them.”  “That whenever you have a parent that may already not be taking the process seriously—so they're trying to drive or they're in the drive-through with their family and I don't have their attention—those parents are the ones where I want to bring in.” “I’ve had a few where, 'oh, well, we’re on our way driving somewhere. Can we do the visit right now?' Not a new diagnostic like that. 'Well, I’ll hold the phone and you can talk to him when he’s sitting in his car seat. 'I don’t think that’s going to work. I’ve had a few like kind of really like, 'oh, my goodness, I can’t believe you thought that was going to be a good idea.'”  Benefits to telehealth “And so I picture these families with four kids and toddlers and screaming kids on buses and just actually getting to the hospital. And what a stressor that is for some families. I love this for them. I love the fact that they don't have to do that.”  “I've been able to see patients as far as a five-hour drive away, […] in some ways—for certain families cut out stress because you don't have to drive to our center in traffic, wait for the hour, then drive home, right, because that disrupts all of the other siblings' schedules and days. It disrupts parents' work schedules.”  “And those who were already working from home, I think really appreciate it because they didn't have to take a lot of time of work to come in.”  “And I imagine there might be value in being in the comfort of your home, in your safest space, going through a difficult process. There might just be a different feel or a different sense of safety or emotional safety. I don't know. I'm guessing that it could also have a positive benefit in that way.” “Reflecting on how a parent might feel after receiving a diagnosis, and then they're like in their home, they can have a cup of tea, they can just relax rather than driving through disgusting city traffic for an hour and a half. It's just a different– it's just a different feel. And I wonder whether that's actually a nice thing to preserve for some families.”  “The other thing that I think that I think it works really well for are split families. I got a chance to meet so many dads that I'd never seen, grandmas, grandpas, teachers, parents showed up. I was like, "This is awesome.” Recently had a mom share the link with the– there was a bunch of stuff going on at school. So she shared the link with the assistant principal who then called me and was like, "Are you sure it's okay?" I'm like, "Oh, heck yeah. Come on." And so fantastic opportunity. Faster for everybody, got everybody on the same page.”  Health disparities Uncertain factors “I think that's the question that we don't have the answer to because we were only able to collect information from those people that participated, but I do suspect that they probably increased disparities or that there were individuals who were not able or chose not to participate in telehealth that would represent that.”  “That's a very hard question to answer. […] What I don't know, is whether there are patients who don't have access. […] I don't know the answer to that because I only know who I'm seeing. I don't know who I'm not seeing.  Same disparities across modalities “I think the same folks that were going to have a really hard time getting into the clinic for transportation or just general life chaos reasons were still having trouble getting in. I don't think that changed. I think that the availability of telehealth for the majority of our patients, though it was very few that had poor access to internet, I think that that increased their access to the service and actually reduced some of the barriers related to coming into a doctor's office and allowed me to establish some trust and rapport before the family had to physically come in and see me […].”  “I feel like it's probably like the same families who have this set of factors that are going to make them probably more likely to be late for your appointment, right. Like they're taking public transit to get there and all of those things, right. And then that same thing– that those same factors just apply to their home environment, like their Internet connection is not the strongest, perhaps, or they've got multiple kids to sort of wrangle in this situation.”  Decreases in disparities “If anything, it's made the evaluation process more accessible to people, because they don't have to try to make it in with medical assistance rideshare or trying to manage getting childcare for their kids while they come in. So I just think it really has improved accessibility for people.”  “And then I think, broadly, it also reduces disparities of no transportation, time out of school, time of work. […] And so I think that that's where we see huge opportunity to reduce disparities through telehealth care.”  Increases in disparities “I think the disparities are there are patients and families that don't have the resource to have the devices that they need and the Wi-Fi and lots of technology pieces that were happening.”  “I think there is a very big aspect of social determinants of health that can come into play in terms of who these assessments work well for and who they don't.”  Greater insight into disparities “And so we learn a lot about the social determinants of health from these interactions. So what does the child's play space look like? How good is or how able is the parent to kind of engage and play with them, or are there other barriers to that? So there's a very rich information.”  “On one hand it's helpful I think for clinicians in creating understanding of, "Wow. This is your world." I get it a little bit more than I could have understood in the clinic or– when you're seeing somebody's home, I think it really helps to provide perspective, or gives you empathy for the fact that this parent is trying so, so hard. And so I think I can provide more empathy in that way.”  Non-native-English speaking families Decreased access to services “The truth is that the families for whom English is not a strength are really being probably further disenfranchised.”  “The non-English -peaking families had a little bit more difficult. And I think they were also the ones that were less comfortable doing virtual visits. Generally speaking, they're the ones who have wanted to come in in person. They've also been the ones that have been more hard hit with COVID.”  “So our predominantly Spanish-speaking […] families are not coming in to the center as much. […] It's because I would imagine they're experiencing a lot more hardship and barriers, or phone number's disconnected or unresponsive. And so I think actually those children, just probably they haven't gotten any services.”  Difficulties related to interpreters “Doing virtual assessments with an interpreter has just proven to be very clunky because you're trying to coach in the moment. And if it has to sort of be relayed through an interpreter, that moment might be gone. And it just gets a lot more challenging.”  “I've always not enjoyed using a phone interpreter. But the times that I do have to use one, not only it's like I can't see the interpreter, I can't see the family. And those are my most dreaded visits over the phone.”  “I am doing many evals over telehealth that are inclusive of a interpreter. And might be some to my style but I feel like, now that that person is an equal sized box on the screen, the interpreter is really in the appointment.”  Institutional support “100%. Whatever we need, whenever we need it, and whatever they could provide, they provided it.” "Because I did not feel that there was a ton of support”  Support/leadership for new initiatives “I think one thing that really helps is that our hospital is very supportive of new initiatives in general.”  “I just wish we had a bit more vision that didn't have to come from the clinicians because we were feeling the pressure and all of the exhaustion that just comes from changing our whole practice”  Productivity “So I have figured this out on my own and was essentially told, "If you don't see patients for these evaluations, then you'll go down to part-time. Why would we pay you full-time if you're not doing the evaluation?"  Technology support “I feel like they did what they could do in this situation based on how prepared or unprepared they were as an institution. So I think the only thing that could have been different is if they were more ahead of the game in terms of telehealth technology to begin with.”  “Some of the barriers really had to do with our own technology related to the healthcare system”  “And then I think the last thing is that each team was only given one camera. […] So just scrambling to help everyone figure out how they could access a computer should they need to work from home”  Clear communication “And good communication. I've really appreciated all the communication that comes out about decisions being made and changes being made, and things like that.”  “Overall leadership did a really nice job of putting out some really clear guidelines […] so there wasn't a guessing game, or it wasn't like, well, this division or this area is pushing harder for you to ramp up than this area.” (Kryszak 2022,)  Support for clinician needs “So giving people like that the flexibility to manage their own time, I think, that was hard to watch that not happen and people be like, “I've never worked harder for you than I am right now. Please, have the respect to know that I can solve this problem.” “So I feel like there was maybe six weeks of tolerance for do your best, do what you can, get caught up on reports. And then I feel like when that shifted about six weeks in, it almost became like now you have to do catch up. […] And it just felt like that sensitivity, understandably, just couldn't be sort of extended as much anymore. So I think that was a tough change.”  Administrative support “What continues to be an ongoing issue is more administrative support for emailing the Zoom links to families. […] we all are required to send Zoom links to all of the patients we see that day.” “And we did a lot of hand-holding. I had phenomenal support. My administrative front desk staff was on it.”  Starting new services “No. I think speed is always an issue. But, hey, you can only develop this process so fast… That was just a reality of– we shut so quickly, like a door shut. What do we do now? And then we all realized we have to go virtual. And then we were all scrambling together and doing our best. But it didn't take very long to get up and running virtually.” “So I think early on, there was definitely some efficiency cost in terms of just getting families up and running with the portal and how it works.”  Increases in efficiency “So in some ways, it sounds like the switch to telehealth actually provides an opportunity for the entire process to be more efficient, potentially.”  “I feel like the interviewing time is shorter We don't have to wait for the family to be roomed, and we don't have to wait for them to go to the bathroom before the visit starts, and we don't have to wait for them to be checked in.”  “And it's also good for space utilization. Because we don't have– I don't know, maybe other people have tons and tons of space. We never have enough clinic space.” “[…] a great boon to my quality of life is that I can type as I talk to people. And so I can generate a fairly decent report like during my interview or at least kind of the bones of it.”  “And also with the online, doing it by telehealth, it allows us to just type everything as we're going to. It feels less intrusive.”  Decreases in efficiency “I think I still go through the same steps. I think in general, the telehealth piece can just take longer because we're waiting to connect … So oftentimes, by the time we get going, we're already like into the appointment timing.”  “I will say that this assessment model is less efficient. […] I would say time as well as documentation takes just a little bit longer because you're writing up your observations probably more […] because you want to make sure that data is there very clear as to "these symptoms are present and these symptoms weren't.”  “I think the cost in the assessment in terms of the resources is a cost in terms of us as a department not really adopting these virtual assessments in terms of– there's usually two of us on an assessment. And that's, again, because the diagnostician have felt that they sort of want that extra set of eyes.”  “I definitely feel like I work more. But again that's not all just because of my assessment, per se. It's also just the added responsibility of doing my own scheduling and calling people. And that piece can be overwhelming.  Decrease in no show rate “So we still occasionally get no-shows and cancellations. For sure, cancellations. No-shows though, we do call right away. And sometimes they're able to jump on the call. So I think that that's improved somewhat.”  “I think for some of the families that would have normally cancelled or just no-showed– I feel like the rate of no-shows have gone down, because it's really easy to just be like, ‘Oh. I'll just click this button.’ Or, ‘I'll just answer my phone.’ In fact, most of the time they have forgotten. And it's like, but they've answered their phone. So they're like, 'Sure. Sure. Sure. Go ahead.’”  “We have a really strong show rate with telehealth, and I think a part of that is, it's a fairly intensive process to work with our clinic to get telehealth signed up and to get the appointment converted… So I think just the timely touchpoints from our support staff really helped the show rate.”  Rates levelled out over time “At the beginning, I think we did have data to demonstrate a reduction in no-shows, but I'd say around August, September, that started to level out, and then we started to see an approaching rate to our previous no-show rate.” “With some of our no-show rate, I feel, ended up stabilizing. But the earlier part, it was definitely a higher rate of showing.”  No change in no show rate “I would say in the second six months, I've had a lot more no-shows and cancellations.” "So I am fairly certain that the rate of no-shows is the same as it was when we were doing the vast majority of our appointments in person.”  Decreases in satisfaction “I think it’s also socially isolating for the provider. In the middle of the telehealth time, it was kind of weird going all day without seeing humans in person.”  “Zoom fatigue is real.” “I mean, my work-life balance is, I guess as challenging as ever because it’s all sort of happening in the same pot right now.”  “One of the things that I feel like is a little more lacking is the teaming that we used to have on the fy. Because we were all in clinic we were all there, we were together, so you would have more opportunity for consultation.”  Increases in satisfaction “[…] in terms of work efficiency, I don’t have to spend that time commuting. I can work on patient paperwork. I can prepare. I have more time to prepare for my visit. So I really like that.”  “I think that being able to toss dinner in the crockpot and then see your next patient has been so amazing for a work-life balance perspective |
| Matthews et al. (2021) | [The client’s] mother reported much more severe symptoms than were observed or indicated in school records, so his presentation was inconsistent with mom’s report and made it difficult to tease out what the actual issues were. He was also unable to sit still for a remote observation due to too many distractions in the home environment. An ADOS was needed to clarify, which indicated the presence of ASD, but on the less impacted side of the spectrum.  This child’s delays in social reciprocity, play skills, and development of relationships were subtle. He showed far more unusual behaviors across the ADOS tasks (hitting himself and his mother during transitions and whenever he could not control an activity). These unusual behaviors did not occur during NODA, and he actually showed fairly good social skills in a variety of NODA situations  Social–emotional reciprocity. This child had some very nice skills and the deficits were not consistently on NODA (i.e., did not link up with mom’s report).  There were many glitches with the remote procedures, and we were unable to administer a KBIT. This client had very subtle symptoms that were not well captured remotely.  Parents differed a lot in their reports, the NODA videos were not great and did not capture social reciprocity or RRBs very well. Pretty subtle case.  Parent provided a confusing history and frequently contradicted herself. The child also had other diagnoses, substance exposure, developmental delays, and was adopted, so the client’s presentation was unclear. The ADOS presses were needed to tease out the client’s presentation.  Client was markedly inattentive during remote observation and refused to participate in several activities. This made it difficult to determine if lack of appropriate social behaviors was due to actual deficits or behavioral concerns. NODA videos provided some evidence, but interactions were mostly client-directed so it was difficult to determine response to conversations and activities of others’ choosing. NODA videos also did not allow for observation of client with same-age peers.  On average, clinicians agreed that the telehealth assessment procedures provided enough information for them to make an accurate diagnostic decision  On average, clinicians would be willing to use telehealth to diagnose this client even if in-person assessments were readily accessible  On average, clinicians somewhat agreed that the clinical information collected from NODA was as useful as an in-person ADOS  On average, clinicians somewhat agreed that the information collected from the remote behavioral observation was as useful as an in-person ADOS  On average, clinicians agreed that the client was attentive during the remote behavioral observation  On average, clinicians agreed that the client’s scores on the Vineland are consistent with his/her general presentation  On average, clinicians agreed that the client’s scores on the KBIT-2 are consistent with his/her general presentation  On average, clinicians strongly agreed that they were able to address the parents’ and/or client’s concerns during the virtual feedback session |
| Spain et al. (2022a) | Forty-four services (85%) had adapted standard diagnostic processes due to the pandemic. Eighteen services (35%) had offered some in-person appointments, and a further eight (15%) were preparing to at the time of the study (prior to the second wave, in England). Most services had started collecting background information “via telephone and video apps.” Clinical interviews with patients and behavioral observation assessments (e.g., the ADOS-2) had initially been postponed, with some services then opting to do these online and others requiring patients to attend in person when feasible. Some participants reported they needed “extra information” beyond what was usual. By the time of the study, most services were offering assessments fully via telehealth, or via a blended approach incorporating telehealth and in-person meetings. Adaptations to in-person appointments included: (1) shortening these; (2) limiting how many people could attend; (3) asking patients and families/carers to complete health screens; (4) wearing PPE (see subtheme 3.2); (5) employing social distancing measures; (6) meeting outside; and (7) using materials that could be sanitized or discarded. (Spain 2022a, Clinicians)  Numerous participants talked about the ADOS-2—a mainstay component of diagnostic assessments—not being validated for use remotely, or with PPE. Services had adopted different solutions to this. Some carried out an ADOS-2 with PPE, nevertheless. Others were using “amended [unvalidated] versions of the ADOS online” to obtain (unscored) “qualitative information,” or they had developed “a new tool to use remotely” comprising a “battery of observational tasks.” Few services had arranged for professionals to be trained up in the BOSA [the Brief Observation of Symptoms of Autism assessment (29); a proxy to the ADOS-2, requiring facilitation by an adult]. Concerns about the lack of validated instruments and consensus about best practice for conducting assessments during pandemic conditions were frequently cited. One participant remarked; “my impression is that everyone is managing this differently and there is little evidence to suggest which assessment method, via video link, is any more valid or efficacious than any other.”  Several participants reported a change in staff allocation, either buddying up with a colleague to see patients when assessments had previously been conducted by sole practitioners, or conversely, having one professional doing the assessment alone. A handful of services reported some tasks—in particular, obtaining the developmental history—were now being conducted by less experienced staff (e.g., assistant psychologists and clinical trainees).  Adaptations to service models appeared to contribute to a lack of parity in service provision, with a knock-on effect on waiting times. One participant reported difficulty “getting interpreters,” meaning people who use English as a second language had to wait longer.  Age also seemed a relevant factor, as highlighted by one participant, “our service has shifted to providing telemedicine-based evaluation services, with a focus on young children. As a result, the wait list is shorter for young children to be seen via telemedicine. The wait list is longer for older children who are more likely to require more in-depth, in-person services.” Conversely, another participant said their service had “mostly kept going with older children and adults.” Different child and adolescent services seemingly followed different guidelines  Patients presenting with increased risk to self or others, and “complex diagnostic cases” also waited longer.  Participants identified challenges to, and advantages of, conducting telehealth assessments. More challenging aspects of telehealth assessments were categorized into: (1) accessibility; (2) IT-related factors; (3) deviation from evidence-based practice; (4) clinical decision-making; (5) assessing autism; (6) patient-related factors; and (7) professional-related factors (see Figure 2). Conversely, advantages were categorized into: (1) pragmatics; (2) potential benefits for patients and their families; and (3) opportunities for innovation (see Figure 2). There was a lack of consensus about whether advantages outweigh the challenges of this approach, with many participants reporting a need to consider these per patient. (Spain 2022a, Clinicians)  Participants rated their degree of confidence in conducting telehealth assessments on a five-point Likert scale. Confidence ranged from “not at all” (N = 3, 6%), through to “a lot,” with 33 (64%) participants feeling at least “quite a bit” confident. Within the qualitative data, opinions about telehealth assessments were polarized. (Spain 2022a, Clinicians)  Many participants said telehealth had “worked really well, and for more ‘straightforward’ diagnostic assessments [has] been very positive.”  Others said this seemed appropriate with “older children/young people where the evidence from parents and school has been strong,”  and that videoconferencing is “good enough where autism is unlikely.”  Telehealth had also allowed patients to “show us things in their homes (collections / interests etc.) that they would otherwise not have been able to” as well as “the interaction between the parent and child in the comfort of their home.” (Spain 2022) [unique insight from TH assessments?)  Difficulties with assessing some symptoms were also reported by participants. Taken together, participants felt remote assessment could render it more challenging to “see the impact of sensory / communication difficulties,” gain “a sense of their [patients’] interaction outside of their home,” observe “every day interaction such as negotiating toilet breaks or whether they want another cup of tea and can explain how they like it,” and assess “eye contact,” “gestures,” and “what they are doing with their lower body/hands at times.”  Others mentioned it is “challenging to evaluate children who are unfamiliar with interacting over a screen . . . difficult to build rapport” and “get a sense of reciprocity.”  Several participants felt “masking” (of autistic traits) may obscure assessment. “Nuances of social interaction” could be less clear, and videoconferencing interaction could cause “conversational asynchrony in the absence of social skills deficits.”  One participant highlighted that this way of working affects “understanding [of] what the child is responding to in their environment and what you cannot see.” (Spain 2022a, Clinicians)  Remote assessment of some sub-groups was, on average, deemed more complex (e.g., young and/or overactive children, adults without informants, people with a learning disability or with complex presentations, or clinical symptoms of paranoia, social anxiety, personality disorder, and/or with limited verbal language).  Participants mentioned that non-autism features could be challenging to assess remotely, such as “differential diagnosis” and “risk.”  Concerns were raised about whether it is possible to see “all the issues” via telehealth, in particular domestic abuse, if someone is substantially underweight, poor hygiene, or self-neglect.  Alongside this, it could be difficult to adequately support patients if they seemed disinhibited, distressed or had started to dissociate. (Spain 2022)  Some participants reported staunchly negative views about telehealth, describing this as “inappropriate,” “non-sensical . . . negligent,” and “unethical.”  One participant said “remote ASD [autism spectrum disorder] assessments have no validity. They do not meet recognized practice standards for diagnostic assessment . . . Diagnostic assessment is an assessment of social interaction and communication.” Reflecting several views, another participant noted “the assessment is much more heavily reliant on the client’s report of their difficulties, [rather] than actual observation of difficulties when in the room with them. This makes it more difficult if the client has limited insight into their difficulties, is poor at understanding what you say, or poor at reporting. Alternatively, a bright client, who is heavily invested in the diagnosis, has researched the diagnosis at length and knows ‘how to answer the questions’ is likely to give a better presentation—but may be more easily able to influence the outcome—whereas seeing them in the room, gut feeling, interaction, their response to interaction gives clues about the likelihood of them having ASD rather than social issues stemming from trauma or attachment.”  Summarizing the juxtaposition participants found themselves in, one said “some clients who present strongly with ASC [autism spectrum condition], for whom a comprehensive background history is available (e.g., ADI-R), a remote assessment via these platforms is very possible.  For others, and perhaps the majority, this is not possible. This is especially the case when [the] presentation is complex, and there are other hypotheses about the root of the clients’ areas of difference (e.g., developmental trauma, acquired brain injury).”  Another considered telehealth “has taken away some clinical observations but provided others at the same time.” Some participants said there was “greater uncertainty about [the] likelihood of getting a clear diagnosis,” and 77% said they had not been able to reach a diagnostic conclusion via telehealth in some instances  Several steps were taken to reach diagnostic conclusions when using telehealth alone was deemed insufficient. These included: (1) offering more telehealth appointments; (2) involving additional professions; (3) obtaining second opinions, (4) using extra assessment measures (e.g., tests of neuropsychological functioning and/or standardized questionnaires); (5) watching home videos for naturalistic observations; (6) speaking to informants about the patient’s behaviors; and (7) if needed, arranging an in-person meeting. Invariably, this meant that patients were placed on an internal waiting list, thus delaying the diagnostic outcome (Spain 2022a, Clinicians)  A handful of services offered patients remote or in person feedback about diagnosis. Some participants felt communicating a diagnostic conclusion via telehealth was much the same as in person.  Conversely, others said telling someone they do not have autism remotely, could feel “tricky,” especially when patients had waited a long time for the assessment. Participants were mindful “we might be missing something because we haven’t seen them face to face.” Some patients and families had complained “if we [health professionals] said it was not ASD then they object as we haven’t met them.”  Conversely, one participant perceived “all outcomes of an assessment can be equally distressing for a client.” Not being in the room together meant it was not possible to have “the same sense of how they [patients] are taking the feedback.” This was compounded by “the connection [rapport] with the individual is not the same . . . [it’s] harder to sit with the uncomfortable feelings/disappointment more,” and it is “more challenging to manage the emotional impact.” Some participants provided patients with a “working diagnosis,” or a “don’t know outcome;” to be revisited when they could meet in person. Alternatively, they might delay giving a diagnosis if rapport was poor or instead, offer formulation-led rather than diagnosis-led feedback  Most participants considered changes to aspects of service design/delivery beneficial. As highlighted by one participant, “the pandemic is not the cause of waiting list difficulties, decades of austerity and underfunding is. A combination of service redesign (balancing NICE guidelines, clinical quality, and need for brevity/throughput), combined with greater financial investment in staffing is needed. This is the issue, not the pandemic!” (Spain 2022a, Clinicians)  Consensus was that different methods of assessment are appropriate for different patients; a combination of in-person and telehealth methods might be pragmatic, if this balanced efficiency and flexibility, with clinical need and risk assessment. Yet some participants had reservations that telehealth might become a panacea, with one describing, “whilst we will become better at it [remote assessment] with practice, I think we need to sit with the discomfort of a lack of quality of remote autism assessments and very strongly resist allowing commissioners to make this become the ’new normal’ forever.” (Spain 2022a, Clinicians)  Development of new policies and guidelines to outline good/best practice for autism assessment during pandemic conditions was emphasized, including “how to assess for each of the diagnostic criteria without being face to face . . . around whether heavier reliance on self-report is adequate and appropriate.” “More robust research” and building “an evidence base” was deemed necessary; focusing on “the best way to assess remotely,” “efficacy” of online assessments and “how remote working might skew assessments of social communication.” Research into “the experience and anxieties of the clinicians doing the assessments . . . [and] the experiences of people receiving diagnosis online” was also mentioned.” (Spain 2022a, Clinicians)  Infrastructural improvements were deemed a priority. This included better administrative support so that professionals did not spend “excessive amounts of clinician time” on nonclinical duties, alongside “reliable . . . better technology,” “better [videoconferencing] platforms with less glitch[es],”, “access to wifi and devices,” and “more tech [technology] support.” It was also suggested schools and GP surgeries could free up a computer for families with poor or no internet connection. More general suggestions included larger clinic spaces and more equipment for behavioral observation assessments, so that meeting in person might be more viable. Having more time to get to know the patient and develop rapport was also key. (Spain 2022a, Clinicians)  Participants advocated the need for “further training” and “more practice,” to enhance IT skills generally and hone capabilities for assessing social communication online. One participant said they would like to “do more assessments to gain a wider insight.” Another highlighted it would be helpful to have “options to watch a gold standard video assessment for a positive diagnosis and no diagnosis, so that we can all be looking for the same sort of service.” Taken together, participants considered more training, along with “peer support” could increase “confidence.” (Spain 2022a, Clinicians)  Most participants welcomed “development” and “validation of adapted test batteries;” specifically, online alternatives to the ADOS-2, IQ tests, and neuropsychological tasks. Building in options to view naturalistic video footage (e.g., home videos), was also considered potentially informative. Some participants thought recording telehealth assessments (with consent), and rating these with colleagues might approximate traditional in person ADOS-2 and ADI-R reliability meetings |
| Spain et al. (2022b) | Using IT Digital poverty was an issue for some participants. One of the participants said the following: ...privileged people can access a lot better and get a much more robust kind of assessment, because it’s not constantly losing connection all the time. So that’s a real concern to me. Professionals working in teams were said to have differing computer literacy levels and access to technology or devices. Some patients and families were described as being accustomed to IT, whereas others could struggle with technology. The lack of familiarity with this could be an independent source of anxiety. One participant remarked they “have one laptop to read, write and call from...the IT is not enough...we need bigger [wider], and more screens,” specifically, one to use for clinical interactions and a second for reading and writing notes. The use of videoconferencing platforms also differs. For example, Zoom videoconference was permitted only in some NHS Health Trusts. One of the digital health services used a custom-built platform.  Some participants noted complexities associated with not being able to blur the screen background: [this] means that people might know more about your personal life than any of us might share The visible contrast between some participants’ and patients’ home environments could be stark: ...there might be things in the background that are a bit distracting for somebody These factors may have influenced engagement and rapport building between patients and professionals, as well as patients’ attention during an assessment.  Flexibility and Efficiency The consensus was that telehealth “gives us flexibility and choices.” Together, participants said that using telehealth could result in (1) fluid appointment times, (2) more accessible appointments, (3) fewer no-shows, (4) options for swiftly filling last-minute cancellations, (5) the possibility for audio and video recording of assessments, (6) less travel and minimal expenditure (eg, on travel or parking), (7) fewer room bookings, (8) environmental benefits or lower carbon footprint, and (9) capacity for recruiting staff living outside of the area. Consequently, many participants felt that flexibility in telehealth could benefit all stakeholders  Logistical Barriers Participants described a range of logistical issues related to patient circumstances that could potentially influence the viability, practical implementation, and success of a telehealth autism assessment. Examples include Environmental factors, such as Location not optimal for an assessment (eg, nowhere suitable to sit at home, dialing into the appointment from work, and walking or riding a bicycle while doing the assessment), Domestic situations not optimal for an assessment (eg, lack of privacy and caring for young children during the assessment), Poor lighting or curtains closed, Poor sound or much background noise, distracted by extraneous cues, finding it hard to sustain attention, and experiencing difficulty in sitting still.  IT-related factors, such as Issues relating to the camera (eg, height and position of the camera, proximity to the patient, and declining to turn the camera on or camera turned off unexpectedly), battery of device running out of charge unexpectedly, Only possible to see what is in front of the camera and not behind or at the periphery.  Engagement-related factors, such as Can feel intrusive to speak to someone while at home, Patient may choose not to join the assessment or wander off part of the way through, displaying behavior that seems inappropriate for the context (eg, patient or family members not fully clothed, disappearing to make a sandwich or go for a walk, and answering the telephone.  Risk-related factors, Domestic abuse, Safeguarding issues  Ethical Considerations Several participants stated that it was unethical for patients to wait longer than necessary. Thus, telehealth was a reasonable option, given the COVID-19 pandemic. Conversely, another participant highlighted that some patients cannot be seen using telehealth (eg, as they do not have IT or their clinical presentation precludes this [see the Theme 4: Clinical Considerations section]), and thus, “there’s a bit of an ethical dilemma there, because obviously they’ve lost their place on our waiting list.” It was also reported that a purely remote assessment may contravene ethical professional standards. As autism is a social communication condition, not meeting a patient in person may mean that naturalistic interactions cannot be adequately assessed.  Limited Standardization Autism assessments lacked standardization, as highlighted by one of the participants, who said that “everybody’s making their best guess at what might work.” Services differed in terms of the (1) number of health professionals involved, (2) depth of information obtained, (3) range of sources from which information was gleaned, (4) types of behavioral observation assessments used, (5) setup of in-person appointments when offered, (6) total number of appointments offered (including feedback), and (7) overall duration of the assessment.  Validity and Reliability Participants’ views differed regarding whether this method of diagnostic assessment was valid and reliable. Reflecting the views of many, as well as a change in usual practice since March 2020, one of the participants said the following: I’ve been really surprised as how useful it is...[before the pandemic], I thought it would be a really bad idea and it wouldn’t be valid, and it would be very limited, not reliable...now I’ve really shifted. However, this was commonly caveated with curiosity and, more specifically, a worry, about “how valid and reliable it is,” especially the behavioral observation components of the assessment (see the Assessment Tools subsection).  Conversely, others said the following: ...feel so strongly about it that it’s not valid...if the full assessment is done remotely, it’s not clinically valid I couldn’t in all conscience assign a diagnosis [about] something as profound as how you interact socially with another human being having never sat in a room with them. Some participants thought telehealth assessments “do work very well, but there are always going to be [patients] when they’re not going to be sufficient.”  The reliability of telehealth assessments could be dependent on the age of the patient, such as being less appropriate for younger children. Others have suggested that this is less reliable for people with “definite speech and language difficulties, with intellectual [learning] disability, learning difficulties such as dyslexia, dyspraxia,” or parents with a learning disability, who may find this a more overwhelming, ambiguous, or confusing meeting. (Spain 2022b, Clinicians)  Assessment Tools Obtaining a developmental history, such as with the Autism Diagnostic Interview–Revised [26], was considered easy via telehealth, and indeed, this commonly occurred before the COVID-19 pandemic.  Formal behavioral observation assessments, such as the Autism Diagnostic Observation Schedule (ADOS)–2 [27], translated less well to web-based forums. Some services decided to complete the ADOS-2 when social distancing measures were no longer in place, resulting in patients being placed on an internal waiting list. Many services demonstrated innovation and developed an ADOS-informed assessment, comprising play-based and conversational tasks. Participants found this beneficial for structuring an appraisal of behaviors suggestive of autism. However, it was noted that these assessments had not been empirically tested, and thus, their psychometric properties (eg, test-retest reliability and interrater reliability) were unknown. On reflection, one of the participants felt that the ADOS-informed assessment they were using “is slightly limiting. We’ve done the best we can.” Another said they had adopted “a really low threshold for review when we weren’t certain...we feel that we may be missing things.” Others described their newly developed assessment as “really successful, and I think it’s been amazing that there’s certain things you can pick up doing it.”  Autism Assessment Opinions on conducting telehealth autism assessments varied. Some participants said that, with practice and experience, this did not differ substantially from assessing someone in person: ...we’ve certainly adjusted to it and for a significant majority of people, doing online assessment has been absolutely fine, and I think the diagnostic conclusion we’ve made has been the same as to whether we’d seen them in a room or not.  Another suggested the following: ...a difficult case is a difficult case, and a straightforward case is a straightforward case...I’m not sure that meeting somebody in person would have made a big difference Some characteristics prototypically associated with autism could be challenging to observe via telehealth, summarized as “you lose a lot of the subtleties...lose out on the interaction.” These included (1) nonverbal behavior (eg, eye contact and quality, flexibility, range, congruence, and integration of facial expressions and gestures); (2) fluidity, responsivity, and reciprocity of social interaction with familiar and unfamiliar others; (3) hypo- and hypersensory sensitivities (eg, to light or noise); (4) repetitive movements and mannerisms, especially those outside the camera shot; and (5) gait and posture.  In addition, it could be difficult to assess coping strategies patients use in their day-to-day lives to manage difficulties or traits: ...you might not see that the curtains are drawn, or you might not see that there’s particular lighting that they need The medium of telehealth could affect judgment about why a trait or behavior was observed. Echoing others’ comments, one of the participants said the following: ...how much of that [social interaction difficulties] is a deficit on their part, and how much of it is just because there might be a slight delay in the internet? Or there might have been a break in the connection. So, it can be complicated to figure out whether their difficulty with reciprocity is because of that, or whether it’s a typical issue. With younger children, there was a specific concern because of the following: ...they’ve not really had much social interaction over the last year [2020], and then you’re trying to discern whether that’s a COVID thing, or whether that’s related to how they prefer things to be anyway. . Seeing patients’ home environments could help with finding out about their preferences and difficulties: I like that the person can show you things in their home. So, if you’re asking somebody about collections, they can then show you that collection, or if they if you’re asking about organisation, you know they can show you things that they’ve organised and so you get that sort of evidence and insight that you wouldn’t get by bringing somebody to a clinic.” It was also easier to “see family dynamics” and “parent child interactions...like mum putting a hand on the child’s shoulder...little things that actually show you a little bit about what their relationship is like.” In contrast, patients could access their “favorite toys” at home; more easily rely on a “scripted, rehearsed kind of story”; and thereby, manage some interactions and ADOS-informed tasks more adeptly.  The remote assessment of domains other than autism could pose challenges. Several participants noted that there is “no possibility of doing a physical examination, not even just blood pressure and pulse, or you know if you thought someone would benefit from blood tests.” Alongside this, many participants described difficulties in assessing the nonspecific elements of social interaction informing diagnostic conclusions: ...do they hold the door open for the informant, then let it go in their face ...who sits next to who ...how do they greet me...how do they sit ...how they cope by coming to a clinic ...if someone’s trousers are stained, or if someone smells, like you’re not getting that information about self-care and things that they might be struggling with and they don’t always have the insight to be able to give you that information verbally.  Some participants said it was more difficult to develop a rapport on the web: [it’s] nice and tangible sitting in a room and there’s some natural toys, and let’s do this task together and let’s work on it together A few participants wondered the following: ...people find it easier to sort of spin you a mistruth, when people want a diagnosis...I’ve got a lady at the moment that I don’t know if she has autism or not, but she’s giving me a lot of conflicting information (Spain 2022b,)  Reaching a Diagnostic Conclusion As for reaching a diagnostic conclusion, one of the participants said the following: I think a lot of it’s to do with the experience. The more you see people with different types of ASD and different presentations, and it takes a long time, but you find patterns in things and in people’s behaviours, so you know you can read [about] it as much as you want or go on as much training courses that you can, but you never quite get it until you’ve been working with individuals for a long period of time.  Others felt that telehealth assessments introduced greater “uncertainty...we spend longer discussing cases,” and the following: I think it [telehealth] makes it much harder as a clinician to be sure of the diagnosis...you can’t rely on your feeling and your responses because you’re just listening to what they’re saying. There was a sense of complexity and difficult diagnostic decisions, and more difficulties for newly qualified health professionals.  Several participants said they would not “confirm a diagnosis with anyone that I have not seen in person.” A few services had adopted an open-door policy, with one participant describing the following: ...assessment is limited by the set up [telehealth]...we generally say we would be happy to review in two years if problems persist. So, if we haven’t given a diagnosis, we’re leaving the door open that we may have missed it Moreover, their service “made recommendations based on the young person’s current needs and situations, so we might give autism-related recommendations even without the diagnosis.”  Communicating the Diagnosis In some services, feedback was provided in person, resulting in a lag between assessment and diagnosis. Some participants felt the following: ...quite callous and not particularly warm and friendly to be doing it over [the internet], like you’re giving someone a life changing diagnosis and you can’t even offer them a cup of tea while you’re doing it or something. You know there’s nothing to kind of soften the blow Another said the following: ...it’s difficult if they are very emotional—you can say warm empathic things, but you can’t hand them the tissue box...you feel a bit inadequate.  Some noted that giving a diagnosis jointly with colleagues seemed easier than giving a diagnosis just as the sole health professional. The patients’ experiences of receiving a diagnosis were important. It was difficult to know “whether it feels better for them to be in their own space and try to process that, or whether it’s better to be in a clinic room.” Some patients were said to “underestimate what the impact of a diagnosis might be like for them.” One participant highlighted the following: ...there’s a bit [of a] difference about you sitting in your bedroom and somebody giving you some news and then hanging up and you’re still kind of sitting in your bedroom, versus coming to a room, somebody telling you something, you’ve been given kind of that time in the room, and then leaving the place where you’ll be given the outcome to travel somewhere different.  Others said receiving a diagnosis could be a relief for patients; however, this was communicated, such as “it explains my past. You know, I’ve got a different narrative now.” Overall, ensuring that patients have “the right emotional support around them” was deemed crucial. Not receiving an autism diagnosis could incur frustration and sadness. Participants reported that relaying this in person or via telehealth could be difficult. One of the participants said their service goes “that extra mile” if a patient does not receive a diagnosis, as “you’ve got to do that in a way that doesn’t [seem] over rejecting...like a huge disappointment.” A few participants dealt with formal complaints whereby parents had said that a diagnosis of autism was not made as the assessment had been conducted via telehealth .  Clinical Complexity Participants said that referrals were increasingly being received for “more and more complex cases.” Examples of complex cases may include patients presenting with limited verbal communication or selective mutism, mental health conditions, enduring personality traits or personality disorders, attachment-based problems, complex trauma, looked-after child status, fetal alcohol syndrome, sensory processing disorders, multimorbidity, or a forensic history.  Some participants felt that the COVID-19 pandemic resulted in a 2-tiered system, with patients with more straightforward presentations being seen via telehealth versus patients with more complexity possibly waiting for longer. Clinical complexity typically meant that the assessment was “more of a challenge”: ...because we need to have more discussion and the MDT process becomes more lengthy, because you have got more to consider Participants found this could make it “really hard to tell whether they’re [patients] autistic or not autistic, and you go away kind of thinking, well after 10 years, I should be able to know whether someone’s autistic or not. It’s very rare that I can’t reach a conclusion [in person], but it seems to be far more complicated [via telehealth].”  In some services, moderate to high risk to self or others, recent suicidality, substance use, high mental health needs, impaired capacity, and known safeguarding concerns precluded the offer of a telehealth assessment. Participants identified a range of risks inherent in clinical work, including to self, to others, and from others. However, the current pandemic context potentially increased the risk for some people, such as “from the fact that you’re doing [the] assessment remotely.” For instance, the following was more crucial: ...know where somebody is when you’re speaking to them [as] they may not be at home...if there were kind of risk issues that came up, it will be important to know where they were.  Another participant said that risky topics could arise when someone “doesn’t want to disclose the ASD assessment to their family or partner or their children.”  Providing feedback for a diagnostic conclusion that patients are not happy with could also feel risky, especially in the absence of good rapport developed in person.  Several participants highlighted that there may be an increased “risk of getting it [the diagnosis] wrong” and incurring “false positives and false negatives.”  Several participants expressed uncertainty about whether the risk can be accurately gauged remotely, with some feeling “it can be quite difficult to hold that risk remotely.” This was deemed especially tricky, as “there isn’t anyone else that’s going to come and pick up and monitor that risk.” Another said the following: ...assessing high risk patients...[such as]...someone who’s very psychotic... creates a bit more anxiety rather than being with the person in the same room and kind of getting a sense of the situation.” Alongside this, it was noted that risk assessment and management is core work for some professional disciplines (eg, psychiatry, clinical psychology, and nursing); however, there may be less emphasis on this in the training of other disciplines: ...there’s extra training to try and bring everybody up to that standard, which is really good, but then sometimes there are still gaps in people’s knowledge and experience It was also apparent that a few services declined referrals for patients deemed to present any risk, again highlighting the potential disparities. Seeing patients in their homes, via the web, could raise unexpected safeguarding concerns. One of the participants said the following regarding the period of a break: ...parents forgot to turn off the camera and volume, and they [the professional] heard inappropriate things where they shouted at their children...it made them feel uncomfortable and they filed a safeguarding concern Another participant identified that talking about safeguarding could potentially increase the risk of further safeguarding issues; for example, when assessing someone in an “abusive...coercive relationship” seen in the company of the abuser. Participants talked about the complexity of dealing with safeguarding issues from their own homes: ...there’s just something about being in a clinic environment where you know you almost kind of have your like safeguarding hat on more. I think because you’re kind of in a role, whereas when you’re at home, sometimes say you know you hear something or you even see something in the background, and I get a moment where I think gosh, this is actually really, you know important...sometimes that’s difficult and not having just that constant kind of liaison with your colleagues is really hard  Commissioning Many services were “not commissioned to provide any postdiagnostic support,” although this was described as follows: ...crucial, because we’ve got lots and lots and lots of children and adults who are being diagnosed with autism. But then, [they ask], what now? Where do I go with this? How can I make this useful? Another participant emphasized the following: ...they [commissioners, managers] sometimes lack the understanding that it is much more than a diagnosis or not. It’s about being able to come away knowing that you feel that you’ve got a pretty good understanding of that child to not only feel comfortable making the diagnostic decision that you made, but also that you’ve been able to do something helpful for families The consensus was that services should be better resourced to provide input after the assessment.  Different Approaches The nature of postdiagnostic interventions differed between services, ranging from no intervention; signposting; resource leaflets; in-depth assessments of functioning; psychoeducation workshops and groups for patients, families, or friends; regular drop-in sessions; and, infrequently, individual sessions. Some services had moved groups to the web, with varying degrees of success. One of the participants described their group now “feels much more like a teaching session...most of the clients don’t want the camera on… so you can feel you could be speaking into the empty [void].” Others considered the move to web-based groups to have “been more successful than I thought it would be”—a valuable asset for patients who may have opted out of or been unable to attend in person. Attending the group on the web also meant that “you don’t have to talk, but you can listen,” reducing potential pressure on patients. (Spain 2022b, Clinicians)  Interventions for Nonautistic Individuals The lack of a postdiagnostic intervention for people who do not receive a diagnosis of autism was mentioned: ...if you don’t have a diagnosis of autism...this is a big issue. Too many autism services just dump them In one of the services, importance was placed on parity of understanding irrespective of diagnosis: ...you still get all of that same process. You still get the formulation. You still get told you will still get a differential diagnosis and opinion and we will still make recommendations for you. So, no matter where you are, autistic or not, you come up with the full assessment and what’s deemed to be your diagnosis, but also what’s deemed to be a formulation, so that if you do have to go into other services, you can take that with you, not have to answer the same questions again. (Spain 2022b, Clinicians)  Team Configuration There was wide variation in workforce configurations. Few participants worked as sole practitioners. Most teams had between 2 and ≥6 professional disciplines represented or available to participate in assessments ad hoc. Echoing many participants’sentiments, one of the participants said the following: I don’t think they [health professionals] need to be from a particular professional background. What’s more important is that they have adequate experience and training and confidence in differential diagnosis across a range of mental conditions and a range of neurodevelopmental conditions and that they know the [care] pathways, whether that’s in the private sector or the NHS, you know, to refer people on for follow-up assessments and follow up treatment (Spain 2022b, Clinicians)  Of the 45 participants, 5 (11% of the sample) worked for a digital health service. Of the remaining participants, most perceived services will continue to use telehealth beyond the COVID-19 pandemic. Whether this would be augmented with at least one in-person appointment depended on factors such as (1) organizational policy, (2) patient choice, (3) clinical complexity, (4) potential risk and safeguarding issues, (5) health professionals ‘preferences, and (6) environmental considerations (eg, whether there is somewhere quiet and confidential that patients and health professionals can use in their own homes or work areas). Reflecting many others’ perspectives, one of the participants noted they are “happy to advocate a hybrid model, as long as the hybrid model is being hybrid to increase capacity without losing quality.” (Spain 2022b, Clinicians)  One of the participants highlighted the following: ...how fortuitous it is that COVID’s come along at exactly the time when we’ve got the technological ability to do this stuff The key to this was the development of new autism assessment tools, taking into account “the cultural differences, and the social cultural context that people are living in.” There was a keenness for “something that does what the ADOS [does], but works in an online environment,” with established validity and reliability. Potential identified innovations included allowing the patient or parents to forward videos of behavior and functioning in everyday situations, using 2 cameras to observe behavior from different angles in the clinic or at home, developing more eye-tracking or neuropsychological tasks for remote use, and having more sophisticated screen-sharing options.  Experiences During the COVID-19 Pandemic The convenience, flexibility, and efficiency of working from home were favored by the participants. However, this was not without its limitations. One of the participants said the following: ...all of us are females in our team and [the] majority of us [have] got children as well, so it’s been a bit of a balance really, having time to home school and time to do the assessments Many participants reported that they “like going to an office and seeing people and being around people” and “prefer sitting in a room with somebody...just to maintain human connection.” Time spent, in person, with colleagues was “absolutely critical...[for] things like humor, team building.” Several participants had experienced a sense of isolation, with one remarking the following: I’ve never met my team. I’ve never met my supervisor. I’ve never met my patients in person...also it felt very isolated with the team and definitely didn’t help with some team dynamics...sometimes it’s nice to knock on somebody’s door and asking the question, or at least meet the people we work with Another said the following: ...it’s difficult working with silence...my mental health is not so good, I think, since I’m always on my own Some general health implications of working at home are highlighted. This could be “more tiring” and “physically intense...I’ve been having eyestrain and more headaches.” Back problems because of “sitting so much” were more common. Another participant said, “the longer [you] spend on a screen, the more burnt out you feel.” Overall, it was suggested that “actually getting up and out of your seat, and not working from a computer all the time, is actually physically more healthy.”  Supervision and Support In keeping with several participants’ viewpoints, one of the participants noted the following: I think we’ve been making things up as we go along and there hasn’t been very much guidance from anywhere about what we [should do] Supervision was deemed “more important now than ever, but it’s more avoided. I think because people are just so tired with it all [the pandemic].” Some participants expressly wanted “safeguarding supervision.” Some pandemic-specific reasons for supervision were described, including the following: ...thinking about the impact of us not having our own routines or home life balance being so blurred, and helping people to find ways to separate work and home when they’re in the same environment...the impact of the pandemic on everybody and how it changed everybody’s life...emotional demands [of the] clinical job...we’re all kind of going through you know extreme stress in our lives Several participants felt that current ways of working raised ethical considerations for discussion in supervision: ...holding [the] tension between what do I clinically feel is the right thing to do...what do families want...what is driving the decision-making process? One of the supervisors reflected they are “a bit more careful when [they’re] supervising remotely and they’ve [the supervisee] assessed remotely,” to ensure the diagnosis reached is accurate. Peer supervision was also described as “really important...[there is] a real power in hearing from other people.” Forums bringing together health professionals working across services were considered useful, with one participant saying the following: ...it [would] be fantastic to you know, see what other people [health professionals] have done and how people have changed things and what they feel, or even if it’s just to confirm that what we’re doing is as good as we can do  Training The following was highlighted: ...none of us were trained to do electronic-based assessments as part of our background core clinical trainings. We’ve been forced into it. Some people have flourished staff wise, others haven’t Few patients had received any telehealth-specific training. Participants identified five telehealth-specific training areas for health professionals: (1) IT skills (eg, general computer literacy, using video conferencing platforms, touch typing, and digital security), (2) clinical skills (eg, knowledge of mental health and differential diagnoses and how to assess them through telehealth, conducting virtual risk assessments and management, and addressing safeguarding concerns remotely), (3) therapeutic skills (eg, deportment on the web, how to enhance virtual engagement, and rapport building), (4) autism-specific skills (eg, how to assess core symptoms and strengths on the web and training in using new [validated] diagnostic tools), and (5) reliability meetings (ie, checking consistency for clinical assessments and standardized tool use). |
| McNally Keehn et al. (2022) | Clinician’s certainty ratings were higher among children diagnosed with ASD as compared with those with developmental delay  In 19% of evaluations, the clinician had to provide technical support to help the child’s caregivers access the telehealth platform at the beginning of the evaluation  Technology barriers were reported by clinicians in 27% of evaluations  Technology barriers were rated as disruptive to the evaluation process in only 15% of the cases  The presence of technology barriers was not associated with the ability to provide a telehealth diagnosis or diagnostic certainty  In 32% of evaluations, clinicians reported barriers due to the family or home setup. These barriers were rated to be disruptive of the evaluation process in 19% of evaluations. The presence of family or home setup barriers was not associated with the ability to provide a telehealth diagnosis or diagnostic certainty  Clinicians reported that the telehealth evaluation modality was adequate for addressing the referral question and caregivers’ concerns in 65% of evaluations. In 53% of evaluations, a preference for seeing the child for a traditional in-person evaluation was endorsed (i.e., had they been given the choice)  92% of clinicians were satisfied with the information obtained from the virtual evaluation  91% of clinicians were satisfied with the overall telehealth evaluation service provided |
| Phelps et al. (2022) | Children who received diagnostic clarity at the virtual visit and were not referred for an in-person follow-up appointment were younger  Children who received diagnostic clarity at the virtual visit and were not referred for an in-person follow-up appointment were more likely to be receiving school services for ASD  Children who received diagnostic clarity at the virtual visit and were not referred for an in-person follow-up appointment reported fewer historical psychiatric diagnoses  Children who received diagnostic clarity at the virtual visit and were not referred for an in-person follow-up appointment scored higher on the CARS-2  Biological sex, race/ethnicity, caregiver-reported adaptive functioning, internalizing symptoms, or externalizing symptoms the presence of interpreter, and having a first degree relative with ASD were not associated with diagnostic clarity  Anxiety and related disorders significantly differentiated groups, such that those with an anxiety disorder were more likely to be referred for in-person evaluation  Mood disorders were found to marginally differentiate group members, such that those with a previously diagnosed mood or related disorder were marginally more likely to be referred for further in-person testing |
| Reisinger et al. (2022) | The vast majority (97%) of clinicians reported being satisfied to some degree with the telehealth assessment  The vast majority (97%) of clinicians reported being satisfied with the information obtained during the telehealth visit  Provider satisfaction was significantly related to whether or not the provider could make a diagnostic determination in the telehealth evaluation (p<0.001). Specifically, providers who were able to provide any diagnosis during the evaluation (M=4.2, SD=0.6) were more satisfied than those who were not (M=3.5, SD=1.0)  Similarly, providers who were able to provide a diagnosis of ASD (M=4.4, SD=0.5) in the telehealth evaluation were more satisfied than those who were unsure of the diagnosis (M=3.6, SD=0.9) or the diagnosis was ruled out (M=4.2, SD=0.6; p<0.001).  Providers who were certain about their diagnosis (M=4.3, SD=0.6) were more satisfied in comparison to those who were uncertain (M=3.4, SD=1.1; p<0.001) (Reisinger 2022).  Technical difficulties (p=0.007) and setup difficulties (p<0.001) were significantly related to provider satisfaction, with providers who did not report any technical or setup difficulties having higher satisfaction ratings  Provider satisfaction was significantly related to child TELE-ASD-PEDS Total scores (p<0.001), with providers rating higher satisfaction for evaluation of children with greater ASD symptom severity |
| Wagner et al. (2021) | All clinicians felt comfortable undergoing a telemedicine assessment for a toddler with concerns for autism  All clinicians felt comfortable making a diagnosis of autism for a toddler following a telemedicine assessment  All clinicians felt comfortable discussing ASD diagnoses and providing recommendations to families during a telemedicine visit  All clinicians felt comfortable walking a caregiver through the TELE-ASD-PEDS during a telemedicine visit  All clinicians found TELE-ASD-PEDS significantly helpful in providing clinical information to guide decision-making  All clinicians felt highly satisfied with the TELE-ASD-PEDS  Providers were also asked for their qualitative feedback on elements of tele-assessment, including feedback on the TELE-ASD-PEDS, challenges encountered during tele-assessment, and perceived benefits of tele-assessment. Regarding the TELE-ASD-PEDS, four providers shared specific feedback on the administration guidelines and rating form. These included suggestions to simplify language and provide more concrete examples within the rating form; as well as challenges related to explaining particular activities (e.g., a “Ready-Set-Go” paradigm) to parents.  Regarding challenges experienced during tele-assessments, all providers reported experiencing technology-related challenges, including dropped calls, inconsistent audio, and challenges with helping caregivers to set up and adjust the camera throughout the appointment.  Some providers (n=5) also reported challenges related to the home environment (e.g., distractions, presence of multiple people with the caregiver and child, availability of play materials).  One provider noted challenges related to obtaining and documenting informed consent for tele-assessment procedures.  Providers also discussed challenges specifically related to the TELE-ASD-PEDS, including reliance on caregivers to share observations of eye contact and language use (n=1), as well as individual differences among caregivers in their understanding of verbal instructions and ways in which they played or interacted with their children (n=4).  Finally, when asked about benefits of tele-assessment, all providers described the value of observing children and families in their home environments. Providers also acknowledge the benefit of continuing to provide services during COVID-19-related restrictions (n=4), as well as benefits to families in terms of limiting travel and transportation barriers (n=6) |
| Wagner et al. (2022) | The majority of clinicians (85%) felt comfortable undergoing a telemedicine assessment for a toddler with concerns about autism  The majority of clinicians (81%) felt comfortable making a diagnosis of autism for a toddler following a telemedicine assessment  The vast majority of clinicians (94%) felt comfortable discussing ASD diagnoses and providing recommendations to families during a telemedicine visit  The majority of clinicians (73%) felt comfortable coaching a caregiver through the TAP during a telemedicine visit  The majority of clinicians (76%) agreed that it would be appropriate for a toddler to receive a diagnosis of ASD over telemedicine, while 19% of them where neutral. Only 6% of clinicians felt this was inappropriate.  All clinicians found TAP helpful in providing clinical information to guide decision-making  Providers also provided qualitative feedback on various aspects of tele-assessment, including perceived benefits and challenges of tele-assessment in general and specific feedback on the TAP. Several themes emerged with regard to the benefits of tele-assessment, many of which were consistent with themes identified in our earlier work (Wagner et al., 2020). For example, the most frequently reported benefit of tele-assessment was the increased access for families who previously faced multiple barriers to attending a traditional, in-person evaluation (n=85).  Many providers also reported that evaluating children in their home environment yielded valuable clinical information  Home observation increased child comfort  and allowed for increased caregiver involvement with the evaluation process  Providers also acknowledged the benefit of continuing to provide services during COVID-19-related restrictions (n=20).  Novel themes not reported in prior work included mention of logistical benefits of tele-assessment, including increased flexibility with scheduling,  increased efficiency,  and the opportunity to have multiple providers attend the visit (n=17).  Many providers (n=87) reported technology-related challenges, including poor wireless connection, dropped calls, and inconsistent audio, all leading to difficulty obtaining adequate clinical observations.  Providers also reported caregiver-related challenges, saying some caregivers had difficulty following directions during assessment activities, keeping the child in the frame, or appeared anxious or unsure of the tele-assessment process (n=59)  Some providers also reported challenges related to the home environment (e.g., distractions, presence of siblings, availability of play materials; n=21). (Wagner 2022,)  New themes emerging from providers’ responses included challenges related to child factors (n=27). Specifically, providers reported that tele-assessment was difficult when assessing children who were older, medically complex, had flexible language, or more subtle symptoms of ASD. Finally, some providers reported that it was challenging to adjust their typical evaluation process (e.g., forgo a physical exam or cognitive assessment, adjust feedback) and find new, evidence-based tools to use (n=33). Providers also identified some challenges specific to the TAP, including the failure of some insurance companies to recognize the test (n=3), insufficient information on scoring (n=5), and caregiver difficulty following the prompts (n=33). A small number of providers expressed that the TAP is too brief for the child to show sufficient symptoms (n=2) and does not probe for or measure a sufficient range of skills and ASD-related symptoms (e.g., imitation, pretend play, restricted and repetitive interests and behaviors, n=3). |
