# Supplementary material for: Families’ and clinicians’ experiences with telehealth assessments for autism: A mixed-methods systematic review
Source: PLOS Digit Health. 2025 Jul 29;4(7):e0000931. doi: 10.1371/journal.pdig.0000931 (PMC12306760; doi:10.1371/journal.pdig.0000931)
Supplement: S1 Table — (DOCX) [file pdig.0000931.s004.docx]

| **Table S1. Critical appraisal of the studies included using the MMAT** | | | | | |
| --- | --- | --- | --- | --- | --- |
| **First** **author** **Year** | **Category** **of** **study** **design** | **Methodological** **quality** **criteria** | **Responses** | | |
|  |  |  | **Yes** | **No** | **Can’t** **tell** |
| Bain 2021 | 4. Quantitative | S1: Are there clear research questions? | Χ |  |  |
|  |  | S2: Do the collected data allow to address the research question? | Χ |  |  |
|  |  | 4.1 Is the sampling strategy relevant to address the research question? | Χ |  |  |
|  |  | 4.2 Is the sample representative of the target population? | Χ |  |  |
|  |  | 4.3 Are the measurements appropriate? | Χ |  |  |
|  |  | 4.4 Is the risk of nonresponse bias low? | Χ |  |  |
|  |  | 4.5 Is the statistical analysis appropriate to answer the research question? | Χ |  |  |
| **Total** **items** **scored** | | | **5** |  |  |
| Bradichansky 2021 | 4. Quantitative | S1: Are there clear research questions? | Χ |  |  |
|  |  | S2: Do the collected data allow to address the research question? | Χ |  |  |
|  |  | 4.1 Is the sampling strategy relevant to address the research question? | Χ |  |  |
|  |  | 4.2 Is the sample representative of the target population? | Χ |  |  |
|  |  | 4.3 Are the measurements appropriate? |  |  | Χ |
|  |  | 4.4 Is the risk of nonresponse bias low? |  |  | Χ |
|  |  | 4.5 Is the statistical analysis appropriate to answer the research question? | Χ |  |  |
| **Total** **items** **scored** | | | **3** |  |  |
| Corona 2021 | 1. Qualitative | S1: Are there clear research questions? | Χ |  |  |
|  |  | S2: Do the collected data allow to address the research question? | Χ |  |  |
|  |  | 1.1. Is the qualitative approach appropriate to answer the research question? |  |  | Χ |
|  |  | 1.2. Are the qualitative data collection methods adequate to address the research question? | Χ |  |  |
|  |  | 1.3. Are the findings adequately derived from the data? | Χ |  |  |
|  |  | 1.4. Is the interpretation of results sufficiently substantiated by data? | Χ |  |  |
|  |  | 1.5. Is there coherence between qualitative data sources, collection, analysis and interpretation? | Χ |  |  |
|  | 4. Quantitative | S1: Are there clear research questions? | Χ |  |  |
|  |  | S2: Do the collected data allow to address the research question? | Χ |  |  |
|  |  | 4.1 Is the sampling strategy relevant to address the research question? | Χ |  |  |
|  |  | 4.2 Is the sample representative of the target population? | Χ |  |  |
|  |  | 4.3 Are the measurements appropriate? | Χ |  |  |
|  |  | 4.4 Is the risk of nonresponse bias low? |  |  | Χ |
|  |  | 4.5 Is the statistical analysis appropriate to answer the research question? | Χ |  |  |
|  | 5. Mixed   methods | S1: Are there clear research questions? | Χ |  |  |
|  |  | S2: Do the collected data allow to address the research question? | Χ |  |  |
|  |  | 5.1. Is there an adequate rationale for using a mixed methods design to address the research question? | Χ |  |  |
|  |  | 5.2. Are the different components of the study effectively integrated to answer the research question? | Χ |  |  |
|  |  | 5.3. Are the outputs of the integration of qualitative and quantitative components adequately interpreted? | Χ |  |  |
|  |  | 5.4. Are divergences and inconsistencies between quantitative and qualitative results adequately addressed? | Χ |  |  |
|  |  | 5.5. Do the different components of the study adhere to the quality criteria of each tradition of the methods involved? |  | Χ |  |
| **Total** **items** **scored** | | | **4** |  |  |
| Corona 2024 | 2. Randomized controlled trials | S1: Are there clear research questions? | Χ |  |  |
|  |  | S2: Do the collected data allow to address the research question? | Χ |  |  |
|  |  | 2.1. Is randomization appropriately performed? |  |  | Χ |
|  |  | 2.2. Are the groups comparable at baseline? | Χ |  |  |
|  |  | 2.3. Are there complete outcome data? | Χ |  |  |
|  |  | 2.4. Are outcome assessors blinded to the intervention provided? | Χ |  |  |
|  |  | 2.5 Did the participants adhere to the assigned intervention? | Χ |  |  |
| **Total** **items** **scored** | | | **4** |  |  |
| Esther 2022 | 1. Qualitative | S1: Are there clear research questions? | Χ |  |  |
|  |  | S2: Do the collected data allow to address the research question? | Χ |  |  |
|  |  | 1.1. Is the qualitative approach appropriate to answer the research question? | Χ |  |  |
|  |  | 1.2. Are the qualitative data collection methods adequate to address the research question? | Χ |  |  |
|  |  | 1.3. Are the findings adequately derived from the data? | Χ |  |  |
|  |  | 1.4. Is the interpretation of results sufficiently substantiated by data? | Χ |  |  |
|  |  | 1.5. Is there coherence between qualitative data sources, collection, analysis and interpretation? | Χ |  |  |
|  | 4. Quantitative | S1: Are there clear research questions? | Χ |  |  |
|  |  | S2: Do the collected data allow to address the research question? | Χ |  |  |
|  |  | 4.1 Is the sampling strategy relevant to address the research question? | Χ |  |  |
|  |  | 4.2 Is the sample representative of the target population? | Χ |  |  |
|  |  | 4.3 Are the measurements appropriate? | Χ |  |  |
|  |  | 4.4 Is the risk of nonresponse bias low? |  | Χ |  |
|  |  | 4.5 Is the statistical analysis appropriate to answer the research question? | Χ |  |  |
|  | 5. Mixed   methods | S1: Are there clear research questions? | Χ |  |  |
|  |  | S2: Do the collected data allow to address the research question? | Χ |  |  |
|  |  | 5.1. Is there an adequate rationale for using a mixed methods design to address the research question? |  | Χ |  |
|  |  | 5.2. Are the different components of the study effectively integrated to answer the research question? | Χ |  |  |
|  |  | 5.3. Are the outputs of the integration of qualitative and quantitative components adequately interpreted? | Χ |  |  |
|  |  | 5.4. Are divergences and inconsistencies between quantitative and qualitative results adequately addressed? |  |  | Χ |
|  |  | 5.5. Do the different components of the study adhere to the quality criteria of each tradition of the methods involved? | Χ |  |  |
| **Total** **items** **scored** | | | **3** |  |  |
| Gibbs 2021 | 1. Qualitative | S1: Are there clear research questions? | Χ |  |  |
|  |  | S2: Do the collected data allow to address the research question? | Χ |  |  |
|  |  | 1.1. Is the qualitative approach appropriate to answer the research question? | Χ |  |  |
|  |  | 1.2. Are the qualitative data collection methods adequate to address the research question? | Χ |  |  |
|  |  | 1.3. Are the findings adequately derived from the data? | Χ |  |  |
|  |  | 1.4. Is the interpretation of results sufficiently substantiated by data? | Χ |  |  |
|  |  | 1.5. Is there coherence between qualitative data sources, collection, analysis and interpretation? | Χ |  |  |
|  | 4. Quantitative | S1: Are there clear research questions? | Χ |  |  |
|  |  | S2: Do the collected data allow to address the research question? | Χ |  |  |
|  |  | 4.1 Is the sampling strategy relevant to address the research question? | Χ |  |  |
|  |  | 4.2 Is the sample representative of the target population? | Χ |  |  |
|  |  | 4.3 Are the measurements appropriate? | Χ |  |  |
|  |  | 4.4 Is the risk of nonresponse bias low? |  | Χ |  |
|  |  | 4.5 Is the statistical analysis appropriate to answer the research question? | Χ |  |  |
|  | 5. Mixed   methods | S1: Are there clear research questions? | Χ |  |  |
|  |  | S2: Do the collected data allow to address the research question? | Χ |  |  |
|  |  | 5.1. Is there an adequate rationale for using a mixed methods design to address the research question? |  | Χ |  |
|  |  | 5.2. Are the different components of the study effectively integrated to answer the research question? | Χ |  |  |
|  |  | 5.3. Are the outputs of the integration of qualitative and quantitative components adequately interpreted? | Χ |  |  |
|  |  | 5.4. Are divergences and inconsistencies between quantitative and qualitative results adequately addressed? |  |  | Χ |
|  |  | 5.5. Do the different components of the study adhere to the quality criteria of each tradition of the methods involved? | Χ |  |  |
| **Total** **items** **scored** | | | **3** |  |  |
| Hodge 2023 | 1. Qualitative | S1: Are there clear research questions? | Χ |  |  |
|  |  | S2: Do the collected data allow to address the research question? | Χ |  |  |
|  |  | 1.1. Is the qualitative approach appropriate to answer the research question? |  | Χ |  |
|  |  | 1.2. Are the qualitative data collection methods adequate to address the research question? | Χ |  |  |
|  |  | 1.3. Are the findings adequately derived from the data? |  |  | Χ |
|  |  | 1.4. Is the interpretation of results sufficiently substantiated by data? |  |  | Χ |
|  |  | 1.5. Is there coherence between qualitative data sources, collection, analysis and interpretation? | Χ |  |  |
|  | 4. Quantitative | S1: Are there clear research questions? | Χ |  |  |
|  |  | S2: Do the collected data allow to address the research question? | Χ |  |  |
|  |  | 4.1 Is the sampling strategy relevant to address the research question? | Χ |  |  |
|  |  | 4.2 Is the sample representative of the target population? | Χ |  |  |
|  |  | 4.3 Are the measurements appropriate? | Χ |  |  |
|  |  | 4.4 Is the risk of nonresponse bias low? |  | Χ |  |
|  |  | 4.5 Is the statistical analysis appropriate to answer the research question? | Χ |  |  |
|  | 5. Mixed methods | S1: Are there clear research questions? | Χ |  |  |
|  |  | S2: Do the collected data allow to address the research question? | Χ |  |  |
|  |  | 5.1. Is there an adequate rationale for using a mixed methods design to address the research question? |  | Χ |  |
|  |  | 5.2. Are the different components of the study effectively integrated to answer the research question? | Χ |  |  |
|  |  | 5.3. Are the outputs of the integration of qualitative and quantitative components adequately interpreted? |  | Χ |  |
|  |  | 5.4. Are divergences and inconsistencies between quantitative and qualitative results adequately addressed? |  | Χ |  |
|  |  | 5.5. Do the different components of the study adhere to the quality criteria of each tradition of the methods involved? |  |  | Χ |
| **Total** **items** **scored** | | | **1** |  |  |
| Jones 2022 | 1. Qualitative | S1: Are there clear research questions? | Χ |  |  |
|  |  | S2: Do the collected data allow to address the research question? | Χ |  |  |
|  |  | 1.1. Is the qualitative approach appropriate to answer the research question? | Χ |  |  |
|  |  | 1.2. Are the qualitative data collection methods adequate to address the research question? | Χ |  |  |
|  |  | 1.3. Are the findings adequately derived from the data? | Χ |  |  |
|  |  | 1.4. Is the interpretation of results sufficiently substantiated by data? |  | Χ |  |
|  |  | 1.5. Is there coherence between qualitative data sources, collection, analysis and interpretation? | Χ |  |  |
|  | 4. Quantitative | S1: Are there clear research questions? | Χ |  |  |
|  |  | S2: Do the collected data allow to address the research question? | Χ |  |  |
|  |  | 4.1 Is the sampling strategy relevant to address the research question? | Χ |  |  |
|  |  | 4.2 Is the sample representative of the target population? | Χ |  |  |
|  |  | 4.3 Are the measurements appropriate? | Χ |  |  |
|  |  | 4.4 Is the risk of nonresponse bias low? | Χ |  |  |
|  |  | 4.5 Is the statistical analysis appropriate to answer the research question? | Χ |  |  |
|  | 5. Mixed   methods | S1: Are there clear research questions? | Χ |  |  |
|  |  | S2: Do the collected data allow to address the research question? | Χ |  |  |
|  |  | 5.1. Is there an adequate rationale for using a mixed methods design to address the research question? | Χ |  |  |
|  |  | 5.2. Are the different components of the study effectively integrated to answer the research question? | Χ |  |  |
|  |  | 5.3. Are the outputs of the integration of qualitative and quantitative components adequately interpreted? | Χ |  |  |
|  |  | 5.4. Are divergences and inconsistencies between quantitative and qualitative results adequately addressed? |  | Χ |  |
|  |  | 5.5. Do the different components of the study adhere to the quality criteria of each tradition of the methods involved? | Χ |  |  |
| **Total** **items** **scored** | | | **4** |  |  |
| Juárez 2018 | 3. Quantitative  non- randomized | S1: Are there clear research questions? | Χ |  |  |
|  |  | S2: Do the collected data allow to address the research question? | Χ |  |  |
|  |  | 3.1. Are the participants representative of the target population? | Χ |  |  |
|  |  | 3.2. Are measurements appropriate regarding both the outcome and intervention (or exposure)? | Χ |  |  |
|  |  | 3.3. Are there complete outcome data? | Χ |  |  |
|  |  | 3.4. Are the confounders accounted for in the design and analysis? |  | Χ |  |
|  |  | 3.5. During the study period, is the intervention administered (or exposure occurred) as intended? | Χ |  |  |
| **Total** **items** **scored** | | | **4** |  |  |
| Kellom 2023 | 1. Qualitative | S1: Are there clear research questions? | Χ |  |  |
|  |  | S2: Do the collected data allow to address the research question? | Χ |  |  |
|  |  | 1.1. Is the qualitative approach appropriate to answer the research question? | Χ |  |  |
|  |  | 1.2. Are the qualitative data collection methods adequate to address the research question? | Χ |  |  |
|  |  | 1.3. Are the findings adequately derived from the data? | Χ |  |  |
|  |  | 1.4. Is the interpretation of results sufficiently substantiated by data? | Χ |  |  |
|  |  | 1.5. Is there coherence between qualitative data sources, collection, analysis and interpretation? | Χ |  |  |
|  | 4. Quantitative | S1: Are there clear research questions? | Χ |  |  |
|  |  | S2: Do the collected data allow to address the research question? | Χ |  |  |
|  |  | 4.1 Is the sampling strategy relevant to address the research question? | Χ |  |  |
|  |  | 4.2 Is the sample representative of the target population? | Χ |  |  |
|  |  | 4.3 Are the measurements appropriate? | Χ |  |  |
|  |  | 4.4 Is the risk of nonresponse bias low? |  |  | Χ |
|  |  | 4.5 Is the statistical analysis appropriate to answer the research question? | Χ |  |  |
|  | 5. Mixed methods | S1: Are there clear research questions? | Χ |  |  |
|  |  | S2: Do the collected data allow to address the research question? | Χ |  |  |
|  |  | 5.1. Is there an adequate rationale for using a mixed methods design to address the research question? | Χ |  |  |
|  |  | 5.2. Are the different components of the study effectively integrated to answer the research question? | Χ |  |  |
|  |  | 5.3. Are the outputs of the integration of qualitative and quantitative components adequately interpreted? | Χ |  |  |
|  |  | 5.4. Are divergences and inconsistencies between quantitative and qualitative results adequately addressed? | Χ |  |  |
|  |  | 5.5. Do the different components of the study adhere to the quality criteria of each tradition of the methods involved? | Χ |  |  |
| **Total** **items** **scored** | | | **4** |  |  |
| Kennelly 2022 | 4. Quantitative | S1: Are there clear research questions? | Χ |  |  |
|  |  | S2: Do the collected data allow to address the research question? | Χ |  |  |
|  |  | 4.1 Is the sampling strategy relevant to address the research question? | Χ |  |  |
|  |  | 4.2 Is the sample representative of the target population? | Χ |  |  |
|  |  | 4.3 Are the measurements appropriate? | Χ |  |  |
|  |  | 4.4 Is the risk of nonresponse bias low? |  | Χ |  |
|  |  | 4.5 Is the statistical analysis appropriate to answer the research question? |  |  | Χ |
| **Total** **items** **scored** | | | **3** |  |  |
| Kryszak 2022 | 1. Qualitative | S1: Are there clear research questions? | Χ |  |  |
|  |  | S2: Do the collected data allow to address the research question? | Χ |  |  |
|  |  | 1.1. Is the qualitative approach appropriate to answer the research question? | Χ |  |  |
|  |  | 1.2. Are the qualitative data collection methods adequate to address the research question? | Χ |  |  |
|  |  | 1.3. Are the findings adequately derived from the data? | Χ |  |  |
|  |  | 1.4. Is the interpretation of results sufficiently substantiated by data? | Χ |  |  |
|  |  | 1.5. Is there coherence between qualitative data sources, collection, analysis and interpretation? | Χ |  |  |
| **Total** **items** **scored** | | | **5** |  |  |
| Matthews 2021 | 1. Qualitative | S1: Are there clear research questions? | Χ |  |  |
|  |  | S2: Do the collected data allow to address the research question? | Χ |  |  |
|  |  | 1.1. Is the qualitative approach appropriate to answer the research question? |  | Χ |  |
|  |  | 1.2. Are the qualitative data collection methods adequate to address the research question? |  | Χ |  |
|  |  | 1.3. Are the findings adequately derived from the data? |  |  | Χ |
|  |  | 1.4. Is the interpretation of results sufficiently substantiated by data? | Χ |  |  |
|  |  | 1.5. Is there coherence between qualitative data sources, collection, analysis and interpretation? |  | Χ |  |
|  | 4. Quantitative | S1: Are there clear research questions? | Χ |  |  |
|  |  | S2: Do the collected data allow to address the research question? | Χ |  |  |
|  |  | 4.1 Is the sampling strategy relevant to address the research question? | Χ |  |  |
|  |  | 4.2 Is the sample representative of the target population? | Χ |  |  |
|  |  | 4.3 Are the measurements appropriate? | Χ |  |  |
|  |  | 4.4 Is the risk of nonresponse bias low? |  | Χ |  |
|  |  | 4.5 Is the statistical analysis appropriate to answer the research question? | Χ |  |  |
|  | 5. Mixed   methods | S1: Are there clear research questions? | Χ |  |  |
|  |  | S2: Do the collected data allow to address the research question? | Χ |  |  |
|  |  | 5.1. Is there an adequate rationale for using a mixed methods design to address the research question? |  | Χ |  |
|  |  | 5.2. Are the different components of the study effectively integrated to answer the research question? | Χ |  |  |
|  |  | 5.3. Are the outputs of the integration of qualitative and quantitative components adequately interpreted? | Χ |  |  |
|  |  | 5.4. Are divergences and inconsistencies between quantitative and qualitative results adequately addressed? | Χ |  |  |
|  |  | 5.5. Do the different components of the study adhere to the quality criteria of each tradition of the methods involved? |  | Χ |  |
| **Total** **items** **scored** | | | **1** |  |  |
| McNally Keehn 2022 | 4. Quantitative | S1: Are there clear research questions? | Χ |  |  |
|  |  | S2: Do the collected data allow to address the research question? | Χ |  |  |
|  |  | 4.1 Is the sampling strategy relevant to address the research question? | Χ |  |  |
|  |  | 4.2 Is the sample representative of the target population? | Χ |  |  |
|  |  | 4.3 Are the measurements appropriate? | X |  |  |
|  |  | 4.4 Is the risk of nonresponse bias low? |  | Χ |  |
|  |  | 4.5 Is the statistical analysis appropriate to answer the research question? | Χ |  |  |
| **Total** **items** **scored** | | | **4** |  |  |
| McNally Keehn 2023 | 4. Quantitative | S1: Are there clear research questions? | Χ |  |  |
|  |  | S2: Do the collected data allow to address the research question? | Χ |  |  |
|  |  | 4.1 Is the sampling strategy relevant to address the research question? | Χ |  |  |
|  |  | 4.2 Is the sample representative of the target population? | Χ |  |  |
|  |  | 4.3 Are the measurements appropriate? | Χ |  |  |
|  |  | 4.4 Is the risk of nonresponse bias low? | Χ |  |  |
|  |  | 4.5 Is the statistical analysis appropriate to answer the research question? | Χ |  |  |
| **Total** **items** **scored** | | | **5** |  |  |
| Phelps 2022 | 4. Quantitative | S1: Are there clear research questions? | Χ |  |  |
|  |  | S2: Do the collected data allow to address the research question? | Χ |  |  |
|  |  | 4.1 Is the sampling strategy relevant to address the research question? | Χ |  |  |
|  |  | 4.2 Is the sample representative of the target population? | Χ |  |  |
|  |  | 4.3 Are the measurements appropriate? | Χ |  |  |
|  |  | 4.4 Is the risk of nonresponse bias low? |  |  | Χ |
|  |  | 4.5 Is the statistical analysis appropriate to answer the research question? | Χ |  |  |
| **Total** **items** **scored** | | | **4** |  |  |
| Reese 2013 | 3. Quantitative  non- randomized | S1: Are there clear research questions? | Χ |  |  |
|  |  | S2: Do the collected data allow to address the research question? | Χ |  |  |
|  |  | 3.1. Are the participants representative of the target population? | Χ |  |  |
|  |  | 3.2. Are measurements appropriate regarding both the outcome and intervention (or exposure)? | Χ |  |  |
|  |  | 3.3. Are there complete outcome data? | Χ |  |  |
|  |  | 3.4. Are the confounders accounted for in the design and analysis? |  | Χ |  |
|  |  | 3.5. During the study period, is the intervention administered (or exposure occurred) as intended? | Χ |  |  |
| **Total** **items** **scored** | | | **4** |  |  |
| Reese 2015 | 4. Quantitative | S1: Are there clear research questions? | Χ |  |  |
|  |  | S2: Do the collected data allow to address the research question? | Χ |  |  |
|  |  | 4.1 Is the sampling strategy relevant to address the research question? | Χ |  |  |
|  |  | 4.2 Is the sample representative of the target population? | Χ |  |  |
|  |  | 4.3 Are the measurements appropriate? | Χ |  |  |
|  |  | 4.4 Is the risk of nonresponse bias low? |  | Χ |  |
|  |  | 4.5 Is the statistical analysis appropriate to answer the research question? |  |  | Χ |
| **Total** **items** **scored** | | | **3** |  |  |
| Reisinger 2022 | 4. Quantitative | S1: Are there clear research questions? | Χ |  |  |
|  |  | S2: Do the collected data allow to address the research question? | Χ |  |  |
|  |  | 4.1 Is the sampling strategy relevant to address the research question? | Χ |  |  |
|  |  | 4.2 Is the sample representative of the target population? | Χ |  |  |
|  |  | 4.3 Are the measurements appropriate? | Χ |  |  |
|  |  | 4.4 Is the risk of nonresponse bias low? |  |  | Χ |
|  |  | 4.5 Is the statistical analysis appropriate to answer the research question? | Χ |  |  |
| **Total** **items** **scored** | | | **4** |  |  |

| Spain 2022a | 1. Qualitative | S1: Are there clear research questions? | Χ |  |  |
| --- | --- | --- | --- | --- | --- |
|  |  | S2: Do the collected data allow to address the research question? | Χ |  |  |
|  |  | 1.1. Is the qualitative approach appropriate to answer the research question? | Χ |  |  |
|  |  | 1.2. Are the qualitative data collection methods adequate to address the research question? | Χ |  |  |
|  |  | 1.3. Are the findings adequately derived from the data? | Χ |  |  |
|  |  | 1.4. Is the interpretation of results sufficiently substantiated by data? | Χ |  |  |
|  |  | 1.5. Is there coherence between qualitative data sources, collection, analysis and interpretation? | Χ |  |  |
|  | 4. Quantitative | S1: Are there clear research questions? | Χ |  |  |
|  |  | S2: Do the collected data allow to address the research question? | Χ |  |  |
|  |  | 4.1 Is the sampling strategy relevant to address the research question? | Χ |  |  |
|  |  | 4.2 Is the sample representative of the target population? | Χ |  |  |
|  |  | 4.3 Are the measurements appropriate? | Χ |  |  |
|  |  | 4.4 Is the risk of nonresponse bias low? |  |  | Χ |
|  |  | 4.5 Is the statistical analysis appropriate to answer the research question? | Χ |  |  |
|  | 5. Mixed   methods | S1: Are there clear research questions? | Χ |  |  |
|  |  | S2: Do the collected data allow to address the research question? | Χ |  |  |
|  |  | 5.1. Is there an adequate rationale for using a mixed methods design to address the research question? |  |  | Χ |
|  |  | 5.2. Are the different components of the study effectively integrated to answer the research question? | Χ |  |  |
|  |  | 5.3. Are the outputs of the integration of qualitative and quantitative components adequately interpreted? | Χ |  |  |
|  |  | 5.4. Are divergences and inconsistencies between quantitative and qualitative results adequately addressed? | Χ |  |  |
|  |  | 5.5. Do the different components of the study adhere to the quality criteria of each tradition of the methods involved? | Χ |  |  |
| **Total** **items** **scored** | | | **4** |  |  |
| Spain 2022b | 1. Qualitative | S1: Are there clear research questions? | Χ |  |  |
|  |  | S2: Do the collected data allow to address the research question? | Χ |  |  |
|  |  | 1.1. Is the qualitative approach appropriate to answer the research question? | Χ |  |  |
|  |  | 1.2. Are the qualitative data collection methods adequate to address the research question? | Χ |  |  |
|  |  | 1.3. Are the findings adequately derived from the data? | Χ |  |  |
|  |  | 1.4. Is the interpretation of results sufficiently substantiated by data? | Χ |  |  |
|  |  | 1.5. Is there coherence between qualitative data sources, collection, analysis and interpretation? | Χ |  |  |
| **Total** **items** **scored** | | | **5** |  |  |
| Stavropoulos 2022 | 1. Qualitative | S1: Are there clear research questions? | Χ |  |  |
|  |  | S2: Do the collected data allow to address the research question? | Χ |  |  |
|  |  | 1.1. Is the qualitative approach appropriate to answer the research question? |  |  | Χ |
|  |  | 1.2. Are the qualitative data collection methods adequate to address the research question? | Χ |  |  |
|  |  | 1.3. Are the findings adequately derived from the data? | Χ |  |  |
|  |  | 1.4. Is the interpretation of results sufficiently substantiated by data? |  | Χ |  |
|  |  | 1.5. Is there coherence between qualitative data sources, collection, analysis and interpretation? | Χ |  |  |
|  | 4. Quantitative | S1: Are there clear research questions? | Χ |  |  |
|  |  | S2: Do the collected data allow to address the research question? | Χ |  |  |
|  |  | 4.1 Is the sampling strategy relevant to address the research question? | Χ |  |  |
|  |  | 4.2 Is the sample representative of the target population? | Χ |  |  |
|  |  | 4.3 Are the measurements appropriate? | Χ |  |  |
|  |  | 4.4 Is the risk of nonresponse bias low? |  |  | Χ |
|  |  | 4.5 Is the statistical analysis appropriate to answer the research question? | Χ |  |  |
|  | 5. Mixed   methods | S1: Are there clear research questions? | Χ |  |  |
|  |  | S2: Do the collected data allow to address the research question? | Χ |  |  |
|  |  | 5.1. Is there an adequate rationale for using a mixed methods design to address the research question? |  | Χ |  |
|  |  | 5.2. Are the different components of the study effectively integrated to answer the research question? | Χ |  |  |
|  |  | 5.3. Are the outputs of the integration of qualitative and quantitative components adequately interpreted? |  | Χ |  |
|  |  | 5.4. Are divergences and inconsistencies between quantitative and qualitative results adequately addressed? | Χ |  |  |
|  |  | 5.5. Do the different components of the study adhere to the quality criteria of each tradition of the methods involved? |  |  | Χ |
|  |  | **Total** **items** **scored** | **2** |  |  |
| Talbott 2020 | 3. Quantitative  non- randomized | S1: Are there clear research questions? | Χ |  |  |
|  |  | S2: Do the collected data allow to address the research question? | Χ |  |  |
|  |  | 3.1. Are the participants representative of the target population? | Χ |  |  |
|  |  | 3.2. Are measurements appropriate regarding both the outcome and intervention (or exposure)? | Χ |  |  |
|  |  | 3.3. Are there complete outcome data? | Χ |  |  |
|  |  | 3.4. Are the confounders accounted for in the design and analysis? |  |  | Χ |
|  |  | 3.5. During the study period, is the intervention administered (or exposure occurred) as intended? | Χ |  |  |
| **Total** **items** **scored** | | | **4** |  |  |
| Talbott 2022a | 3. Quantitative  non- randomized | S1: Are there clear research questions? | Χ |  |  |
|  |  | S2: Do the collected data allow to address the research question? | Χ |  |  |
|  |  | 3.1. Are the participants representative of the target population? | Χ |  |  |
|  |  | 3.2. Are measurements appropriate regarding both the outcome and intervention (or exposure)? | Χ |  |  |
|  |  | 3.3. Are there complete outcome data? | Χ |  |  |
|  |  | 3.4. Are the confounders accounted for in the design and analysis? |  |  | Χ |
|  |  | 3.5. During the study period, is the intervention administered (or exposure occurred) as intended? | Χ |  |  |
| **Total** **items** **scored** | | | **4** |  |  |
| Talbott   2022b | 1. Qualitative | S1: Are there clear research questions? | Χ |  |  |
|  |  | S2: Do the collected data allow to address the research question? | Χ |  |  |
|  |  | 1.1. Is the qualitative approach appropriate to answer the research question? | Χ |  |  |
|  |  | 1.2. Are the qualitative data collection methods adequate to address the research question? | Χ |  |  |
|  |  | 1.3. Are the findings adequately derived from the data? | Χ |  |  |
|  |  | 1.4. Is the interpretation of results sufficiently substantiated by data? | Χ |  |  |
|  |  | 1.5. Is there coherence between qualitative data sources, collection, analysis and interpretation? | Χ |  |  |
|  | 4. Quantitative | S1: Are there clear research questions? | Χ |  |  |
|  |  | S2: Do the collected data allow to address the research question? | Χ |  |  |
|  |  | 4.1 Is the sampling strategy relevant to address the research question? | Χ |  |  |
|  |  | 4.2 Is the sample representative of the target population? | Χ |  |  |
|  |  | 4.3 Are the measurements appropriate? | Χ |  |  |
|  |  | 4.4 Is the risk of nonresponse bias low? | Χ |  |  |
|  |  | 4.5 Is the statistical analysis appropriate to answer the research question? | Χ |  |  |
|  | 5. Mixed   methods | S1: Are there clear research questions? | Χ |  |  |
|  |  | S2: Do the collected data allow to address the research question? | Χ |  |  |
|  |  | 5.1. Is there an adequate rationale for using a mixed methods design to address the research question? | Χ |  |  |
|  |  | 5.2. Are the different components of the study effectively integrated to answer the research question? | Χ |  |  |
|  |  | 5.3. Are the outputs of the integration of qualitative and quantitative components adequately interpreted? | Χ |  |  |
|  |  | 5.4. Are divergences and inconsistencies between quantitative and qualitative results adequately addressed? |  |  | Χ |
|  |  | 5.5. Do the different components of the study adhere to the quality criteria of each tradition of the methods involved? | Χ |  |  |
| **Total** **items** **scored** | | | **4** |  |  |
| Wagner 2021 | 1. Qualitative | S1: Are there clear research questions? | Χ |  |  |
|  |  | S2: Do the collected data allow to address the research question? | Χ |  |  |
|  |  | 1.1. Is the qualitative approach appropriate to answer the research question? | Χ |  |  |
|  |  | 1.2. Are the qualitative data collection methods adequate to address the research question? | Χ |  |  |
|  |  | 1.3. Are the findings adequately derived from the data? |  |  | Χ |
|  |  | 1.4. Is the interpretation of results sufficiently substantiated by data? |  |  | Χ |
|  |  | 1.5. Is there coherence between qualitative data sources, collection, analysis and interpretation? | Χ |  |  |
|  | 3. Quantitative  non- randomized | S1: Are there clear research questions? | Χ |  |  |
|  |  | S2: Do the collected data allow to address the research question? | Χ |  |  |
|  |  | 4.1 Is the sampling strategy relevant to address the research question? | Χ |  |  |
|  |  | 4.2 Is the sample representative of the target population? | Χ |  |  |
|  |  | 4.3 Are the measurements appropriate? | X |  |  |
|  |  | 4.4 Is the risk of nonresponse bias low? |  | Χ |  |
|  |  | 4.5 Is the statistical analysis appropriate to answer the research question? | Χ |  |  |
|  | 5. Mixed   methods | S1: Are there clear research questions? | Χ |  |  |
|  |  | S2: Do the collected data allow to address the research question? | Χ |  |  |
|  |  | 5.1. Is there an adequate rationale for using a mixed methods design to address the research question? |  |  | Χ |
|  |  | 5.2. Are the different components of the study effectively integrated to answer the research question? | Χ |  |  |
|  |  | 5.3. Are the outputs of the integration of qualitative and quantitative components adequately interpreted? | Χ |  |  |
|  |  | 5.4. Are divergences and inconsistencies between quantitative and qualitative results adequately addressed? | Χ |  |  |
|  |  | 5.5. Do the different components of the study adhere to the quality criteria of each tradition of the methods involved? |  | Χ |  |
| **Total** **items** **scored** | | | **3** |  |  |
| Wagner 2022 | 1. Qualitative | S1: Are there clear research questions? | Χ |  |  |
|  |  | S2: Do the collected data allow to address the research question? | Χ |  |  |
|  |  | 1.1. Is the qualitative approach appropriate to answer the research question? | Χ |  |  |
|  |  | 1.2. Are the qualitative data collection methods adequate to address the research question? | Χ |  |  |
|  |  | 1.3. Are the findings adequately derived from the data? |  |  | Χ |
|  |  | 1.4. Is the interpretation of results sufficiently substantiated by data? |  |  | Χ |
|  |  | 1.5. Is there coherence between qualitative data sources, collection, analysis and interpretation? | Χ |  |  |
|  | 4. Quantitative | S1: Are there clear research questions? | Χ |  |  |
|  |  | S2: Do the collected data allow to address the research question? | Χ |  |  |
|  |  | 4.1 Is the sampling strategy relevant to address the research question? | Χ |  |  |
|  |  | 4.2 Is the sample representative of the target population? | Χ |  |  |
|  |  | 4.3 Are the measurements appropriate? | Χ |  |  |
|  |  | 4.4 Is the risk of nonresponse bias low? |  | Χ |  |
|  |  | 4.5 Is the statistical analysis appropriate to answer the research question? | Χ |  |  |
|  | 5. Mixed   methods | S1: Are there clear research questions? | Χ |  |  |
|  |  | S2: Do the collected data allow to address the research question? | Χ |  |  |
|  |  | 5.1. Is there an adequate rationale for using a mixed methods design to address the research question? | Χ |  |  |
|  |  | 5.2. Are the different components of the study effectively integrated to answer the research question? | Χ |  |  |
|  |  | 5.3. Are the outputs of the integration of qualitative and quantitative components adequately interpreted? | Χ |  |  |
|  |  | 5.4. Are divergences and inconsistencies between quantitative and qualitative results adequately addressed? |  |  | Χ |
|  |  | 5.5. Do the different components of the study adhere to the quality criteria of each tradition of the methods involved? | Χ |  |  |
| **Total** **items** **scored** | | | **3** |  |  |

Hong QN, Gonzalez-Reyes A, Pluye P. Improving the usefulness of a tool for appraising the quality of qualitative, quantitative and mixed methods studies, the Mixed Methods Appraisal Tool (MMAT). J Eval Clin Pract. 2018;24(3):459-467. <https://doi.org/10.1111/jep.12884>
