# Supplementary material for: Families’ and clinicians’ experiences with telehealth assessments for autism: A mixed-methods systematic review
Source: PLOS Digit Health. 2025 Jul 29;4(7):e0000931. doi: 10.1371/journal.pdig.0000931 (PMC12306760; doi:10.1371/journal.pdig.0000931)
Supplement: S2 Table — (DOCX) [file pdig.0000931.s005.docx]

| **Table S2.** List of studies subjected to full-text screening | | | |
| --- | --- | --- | --- |
| No | Study reference | Decision | Reason |
| 1 | McDonnell CG, Bradley CC, Kanne SM, Lajonchere C, Warren Z, Carpenter LA. When are we sure? Predictors of clinician certainty in the diagnosis of autism spectrum disorder. Journal of Autism and Developmental Disorders. 2019;49(4):1391–401. | Excluded | Did not explore telehealth applications |
| 2 | Fortney S. Client-Level Barriers to Successful Utilization of Telehealth for Clients with Autism Spectrum Disorder. The Ohio State University; 2021. | Excluded | Examined/ elaborated on an intervention |
| 3 | Dow D, Holbrook A, Toolan C, et al. The Brief Observation of Symptoms of Autism (BOSA): Development of a New Adapted Assessment Measure for Remote Telehealth Administration Through COVID-19 and Beyond. J Autism Dev Disord. 2022;52(12):5383-5394. doi:10.1007/s10803-021-05395-w | Excluded | No relevant data |
| 4 | Seeber RE, Spieler GS, Moon SJ, Kim JW. 1.34 Wearable technology and ASD: A review. J Am Acad Child Adolesc Psychiatry. 2019;58(10):S157–8. | Excluded | Review study |
| 5 | Choi K, Zima BT. 3.27 Parent survey of habilitative teletherapy for children with ASD. J Am Acad Child Adolesc Psychiatry. 2022;61(10):S236. | Excluded | Examined/ elaborated on an intervention |
| 6 | Grodberg D. 5.4 Parent engagement using digital health tools. J Am Acad Child Adolesc Psychiatry. 2019;58(10):S138. | Excluded | Examined/ elaborated on an intervention |
| 7 | Dalmau YP, Dolezal D, Boelter E. 6.2 A hospital model of telehealth behavioral assessment and intervention for individuals with ASD and severe and specialized treatment needs. J Am Acad Child Adolesc Psychiatry. 2018;57(10):S9. | Excluded | Examined/ elaborated on an intervention |
| 8 | Gwynette MF. 7.3 Talk to me: A review of autism spectrum disorder and technology. J Am Acad Child Adolesc Psychiatry. 2020;59(10):S136–7. | Excluded | Conference abstract |
| 9 | Ceranoglu TA. 46.2 Autism spectrum disorders and electronic media use: Empathy with avatars. J Am Acad Child Adolesc Psychiatry. 2016;55(10):S70. | Excluded | Conference abstract |
| 10 | Ganz JB, Pustejovsky JE, Reichle J, Vannest KJ, Foster M, Pierson LM, et al. A case for increased rigor in AAC research: A methodological quality review. Educ Train Autism Dev Disabil. 2023;58(1):3–21. | Excluded | Examined/ elaborated on an intervention |
| 11 | Chericoni N, Balboni G, Costanzo V, Mancini A, Prosperi M, Lasala R, et al. A combined study on the use of the Child Behavior Checklist 1½–5 for identifying autism spectrum disorders at 18 months. J Autism Dev Disord. 2021;51(11):3829–42. | Excluded | Did not explore telehealth applications |
| 12 | Kerub O, Haas EJ, Meiri G, Davidovitch N, Idan M. A comparison between two screening approaches for ASD among toddlers in Israel. J Autism Dev Disord. 2020;50(5):1553–60. | Excluded | Did not explore telehealth applications |
| 13 | Mujeeb Rahman KK, Monica Subashini M. A deep neural network-based model for screening autism spectrum disorder using the Quantitative Checklist for Autism in Toddlers (QCHAT). J Autism Dev Disord. 2022;52(6):2732–46. | Excluded | Did not explore telehealth applications |
| 14 | Kane CL, DeBar RM. A descriptive review of telehealth for individuals with autism spectrum disorder. Behav Modif. 2023;47(2):504–46. | Excluded | Review study |
| 15 | Prieto LA, Meera B, Katz H, Columna L. A feasibility trial for virtual administration of the Test of Gross Motor Development-3 for children with autism spectrum disorders during the COVID-19 pandemic. Adapt Phys Activ Q. 2022;39(4):446–55. | Excluded | Did not examine relevant outcomes |
| 16 | Zervogianni V, Fletcher-Watson S, Herrera G, Goodwin M, Pérez-Fuster P, Brosnan M, et al. A framework of evidence-based practice for digital support, co-developed with and for the autism community. Autism. 2020;24(6):1411–22. | Excluded | Theoretical paper |
| 17 | Ortiz JL. A generic qualitative inquiry of Hispanic families with a child with autism [Internet]. Diss Abstr Int Sect B Sci Eng. 2023;84. Available from: https://search.ebscohost.com/login.aspx?direct=true&db=psyh&AN=2023-68885-256&site=ehost-live | Excluded | Did not explore telehealth applications |
| 18 | Pickard KE, Wainer AL, Bailey KM, Ingersoll BR. A mixed-method evaluation of the feasibility and acceptability of a telehealth-based parent-mediated intervention for children with autism spectrum disorder. Autism. 2016;20(7):845–55. | Excluded | Examined/ elaborated on an intervention |
| 19 | Tran SQ. A multidimensional treatment integrity assessment of parent coaching in a telehealth parent training program for autism spectrum disorder [Internet]. Diss Abstr Int Sect B Sci Eng. 2018;79. Available from: <https://search.ebscohost.com/login.aspx?direct=true&db=psyh&AN=2018-48576-153&site=ehost-live> | Excluded | Examined/ elaborated on an intervention |
| 20 | Gentile M, Messineo L, La Guardia D, Arrigo M, Città G, Ayala A, et al. A parent-mediated telehealth program for children with autism spectrum disorder: Promoting parents’ ability to stimulate the children’s learning, reduce parenting stress, and boost their sense of parenting empowerment. J Autism Dev Disord. 2022;52(12):5285–300. | Excluded | Examined/ elaborated on an intervention |
| 21 | Koirala A, Yu Z, Schiltz H, Van Hecke A, Armstrong B, Zheng Z. A preliminary exploration of virtual reality-based visual and touch sensory processing assessment for adolescents with autism spectrum disorder. IEEE Trans Neural Syst Rehabil Eng. 2021;29:619–28. | Excluded | Explored virtual reality, machine learning or robotic applications |
| 22 | Minissi ME, Chicchi Giglioli IA, Mantovani F, Sirera M, Abad L, Alcañiz M. A qualitative and quantitative virtual reality usability study for the early assessment of ASD children. Annu Rev CyberTher Telemed. 2021;19:47–51. | Excluded | Explored virtual reality, machine learning or robotic applications |
| 23 | Corona LL, Wagner L, Hooper M, Weitlauf A, Foster TE, Hine J, et al. A randomized trial of the accuracy of novel telehealth instruments for the assessment of autism in toddlers. J Autism Dev Disord [Internet]. 2023. Available from: <https://www.springer.com/journal/10803> | Included |  |
| 24 | Larsen A, Schieltz KM, Barrett A, O’Brien MJ. A retrospective analysis of therapists’ coaching behavior when directing parents to conduct behavioral assessments and treatments via telehealth. Behav Modif. 2023;47(1):154–84. | Excluded | Examined/ elaborated on an intervention |
| 25 | Stavropoulos KKM, Bolourian Y, Blacher J. A scoping review of telehealth diagnosis of autism spectrum disorder. PLoS One [Internet]. 2022;17(2). Available from: <https://search.ebscohost.com/login.aspx?direct=true&db=psyh&AN=2022-34369-001&site=ehost-live> | Excluded | Review study |
| 26 | Galligan M, Losh A, Veytsman E, Blacher J. A service desert: Unmet needs of individuals with autism and their families. J Dev Disabil. 2022;27(3):1–14. | Excluded | Did not explore telehealth applications |
| 27 | Freeman NC, Grigoriadis A. A survey of assessment practices among health professionals diagnosing females with autism. Res Dev Disabil. 2023;135:104445. | Excluded | Did not explore telehealth applications |
| 28 | Evans K, van der Meer L, Eggleston MJ, Taylor LJ, Thabrew H, Waddington H, et al. A survey of autistic adults from New Zealand on the autism diagnostic process during adolescence and adulthood. J Autism Dev Disord. 2022;52(2):771–81. | Excluded | Did not explore telehealth applications |
| 29 | Liu X, Zhao W, Qi Q, Luo X. A survey on autism care, diagnosis, and intervention based on mobile apps focusing on usability and software design. Sensors (Basel). 2023;23(14):1–15. | Excluded | Review study |
| 30 | Valentine AZ, Brown BJ, Groom MJ, Young E, Hollis C, Hall CL. A systematic review evaluating the implementation of technologies to assess, monitor and treat neurodevelopmental disorders: A map of the current evidence. Clin Psychol Rev [Internet]. 2020;80. Available from: <https://search.ebscohost.com/login.aspx?direct=true&db=psyh&AN=2020-68772-001&site=ehost-live> | Excluded | Review study |
| 31 | Dahiya AV, McDonnell C, DeLucia E, Scarpa A. A systematic review of remote telehealth assessments for early signs of autism spectrum disorder: Video and mobile applications. Pract Innov (Wash D C). 2020;5(2):150–64. | Excluded | Review study |
| 32 | Dahiya AV, DeLucia E, McDonnell CG, Scarpa A. A systematic review of technological approaches for autism spectrum disorder assessment in children: Implications for the COVID-19 pandemic. Res Dev Disabil. 2021;109:103852. | Excluded | Review study |
| 33 | Meimei L, Zenghui M. A systematic review of telehealth screening, assessment, and diagnosis of autism spectrum disorder. Child Adolesc Psychiatry Ment Health. 2022;16(1):79. | Excluded | Review study |
| 34 | Petruccelli M, Ramella L, Schaefer AJ, Christopher SR, Carter AS, Eisenhower A, et al. A taxonomy of reported harms in pediatric autism spectrum disorder screening: Provider and parent perspectives. J Autism Dev Disord. 2022;52(2):647–73. | Excluded | Did not explore telehealth applications |
| 35 | Kang LRJ, Barlott T, Turpin M, Urbanowicz A. A trial of the AASPIRE healthcare toolkit with Australian adults on the autism spectrum. Aust J Prim Health. 2022;28(4):350–6. | Excluded | Adult sample |
| 36 | Robles M, Namdarian N, Otto J, Wassiljew E, Navab N, Falter-Wagner C, et al. A virtual reality based system for the screening and classification of autism. IEEE Trans Vis Comput Graph. 2022;28(5):2168–78. | Excluded | Explored virtual reality, machine learning or robotic applications |
| 37 | Aranki J, Wright P, Pompa-Craven P, Lotfizadeh AD. Acceptance of telehealth therapy to replace in-person therapy for autism treatment during COVID-19 pandemic: An assessment of patient variables. Telemed J E Health. 2022;28(9):1342–9. | Excluded | Examined/ elaborated on an intervention |
| 38 | Landau-Taylor J, McGivney C, Christiansen A. Access to care after autism diagnosis during the COVID-19 pandemic: A quality improvement project. J Dev Behav Pediatr. 2023;44(2):e73–9. | Excluded | Examined/ elaborated on an intervention |
| 39 | Latifi R, Gunn JKL, Bakiu E, Boci A, Dasho E, Olldashi F, et al. Access to specialized care through telemedicine in limited-resource country: Initial 1,065 teleconsultations in Albania. Telemed J E Health. 2016;22(12):1024–31. | Excluded | Non-ASD sample |
| 40 | Kazak A, Ramirez AP, Scialla MA, Alderfer MA, Sewell-Roberts C, Treadwell-Deering D. Adaptation and pilot implementation of the Psychosocial Assessment Tool for Autism Spectrum Disorders (PAT-ASD). J Autism Dev Disord. 2023;53(11):4308–17. | Excluded | Not assessing for ASD |
| 41 | Lee-Hou T, Jeng-Wen L. Adaptation of diagnosis from autism spectrum disorder to social communication disorder in adolescents with ADHD. J Autism Dev Disord. 2020;50(2):685–7. | Excluded | Case study |
| 42 | Summers M, Herrema R, Dennison E. Adapting behavioural observations for neurodevelopmental assessment: Development and evaluation of online play-based assessments in community CYPS during COVID-19 pandemic. Clin Psychol Forum. 2022;(358):24. | Excluded | Theoretical paper |
| 43 | Eapen V, Hiscock H, Williams K. Adaptive innovations to provide services to children with developmental disabilities during the COVID-19 pandemic. J Paediatr Child Health. 2021;57(1):9–11. | Excluded | Theoretical paper |
| 44 | Massrey C, Wells D, Gibbs K, Choate J, Hall TN, Klinepeter E. Adding tools to our toolkit: How to care for children with autism and challenging behaviors. Neurology. 2023;100(17):e1793–4. | Excluded | Did not explore telehealth applications |
| 45 | Byrne-Smith A. Adolescence, autism and technology: How technology can impact the social lives and wellbeing of adolescents with an autism diagnosis [Internet]. Diss Abstr Int Sect B Sci Eng. 2023;84. Available from: <https://search.ebscohost.com/login.aspx?direct=true&db=psyh&AN=2022-90098-135&site=ehost-live> | Excluded | Examined/ elaborated on an intervention |
| 46 | Shogren KA, Mosconi MW, Raley SK, Dean EE, Edwards B, Wallisch A, et al. Advancing the personalization of assessment and intervention in autistic adolescents and young adults by targeting self-determination and executive processes. Autism Adulthood. 2021;3(4):289–99. | Excluded | Did not explore telehealth applications |
| 47 | Fox E, Gerdts J, Ahlers K, Kotchick B. Age-based tracks: A method to tailor autism diagnostic evaluation in large-scale autism specialty centers. J Clin Med. 2022;11(21):6332. | Excluded | Did not explore telehealth applications |
| 48 | Mertz L. AI, virtual reality, and robots advancing autism diagnosis and therapy. IEEE Pulse. 2021;12(5):6–10. | Excluded | Explored virtual reality, machine learning or robotic applications |
| 49 | Butsch M, Jardon-Aites M, Cartisano T, Jordan B, Flake E, Roy D, et al. An accurate alternative method for diagnoses of autism spectrum disorder using video telehealth parent-led play in the military medical system. Pediatrics. 2021;147(3):e2020011029. | Excluded | Conference abstract |
| 50 | Barton EE, Harris B, Leech N, Stiff L, Choi G, Joel T. An analysis of state autism educational assessment practices and requirements. J Autism Dev Disord. 2016;46(3):737–47. | Excluded | Did not explore telehealth applications |
| 51 | Murray AL. An evaluation of video-based BST of functional behavior assessment for teachers in a South African school for autism [Internet]. Diss Abstr Int Sect B Sci Eng. 2020;81. Available from: <https://search.ebscohost.com/login.aspx?direct=true&db=psyh&AN=2020-28117-083&site=ehost-live> | Excluded | Examined/ elaborated on an intervention |
| 52 | Blake AN. An examination of perceptions, importance, and value of play by professionals serving children with autism spectrum disorder [Internet]. Diss Abstr Int Sect B Sci Eng. 2021;82. Available from: <https://search.ebscohost.com/login.aspx?direct=true&db=psyh&AN=2021-27915-284&site=ehost-live> | Excluded | Did not explore telehealth applications |
| 53 | Green J, Leadbitter K, Ainsworth J, Bucci S. An integrated early care pathway for autism. Lancet Child Adolesc Health. 2022;6(5):335–44. | Excluded | Theoretical paper |
| 54 | Parmanto B, Pulantara IW, Schutte JL, Saptono A, McCue MP. An integrated telehealth system for remote administration of an adult autism assessment. Telemed J E Health. 2013;19(2):88–94. | Excluded | Adult sample |
| 55 | Shawler LA, Clayborne JC, Nasca B, O’Connor JT. An intensive telehealth assessment and treatment model for an adult with developmental disabilities. Res Dev Disabil. 2021;111:103876. | Excluded | Adult sample |
| 56 | Holmes LM, Warreyn P, Uriarte BV, Boonen C, Fletcher-Watson S. An international survey of parental attitudes to technology use by their autistic children at home. J Autism Dev Disord. 2019;49(4):1517–30. | Excluded | Did not explore telehealth applications |
| 57 | Boisvert MK. An investigation of the efficacy of speech and language interventions with students with ASD using telepractice [Internet]. Diss Abstr Int Sect B Sci Eng. 2013;73. Available from: <https://search.ebscohost.com/login.aspx?direct=true&db=psyh&AN=2013-99120-009&site=ehost-live> | Excluded | Examined/ elaborated on an intervention |
| 58 | Ramappa S, Anderson A, Jung J, Chu R, Cummings KK, Patterson G, et al. An observed assessment of sensory responsivity in autism spectrum disorders: Associations with diagnosis, age, and parent report. J Autism Dev Disord. 2023;53(10):3860–72. | Excluded | Did not explore telehealth applications |
| 59 | Hollis C, Falconer CJ, Martin JL, Whittington C, Stockton S, Glazebrook C, et al. Annual research review: Digital health interventions for children and young people with mental health problems - a systematic and meta-review. J Child Psychol Psychiatry. 2017;58(4):474–503. | Excluded | Review study |
| 60 | Ozalp Akin E, Akbas A, Atasoy SC, Kanatli MC, Ince Acici S, Mustafayev R, et al. Applicability of the Guide for Monitoring Child Development as a telehealth-delivered intervention during the pandemic. Front Pediatr. 2022;10:884779. | Excluded | Non-ASD sample |
| 61 | Alcañiz Raya M, Chicchi Giglioli IA, Marín-Morales J, Higuera-Trujillo JL, Olmos E, Minissi ME, et al. Application of supervised machine learning for behavioral biomarkers of autism spectrum disorder based on electrodermal activity and virtual reality. Front Hum Neurosci [Internet]. 2020;14. Available from: <https://search.ebscohost.com/login.aspx?direct=true&db=psyh&AN=2020-27464-001&site=ehost-live> | Excluded | Explored virtual reality, machine learning or robotic applications |
| 62 | Wan G, Kong X, Sun B, Yu S, Tu Y, Park J, et al. Applying eye tracking to identify autism spectrum disorder in children. J Autism Dev Disord. 2019;49(1):209–15. | Excluded | Explored virtual reality, machine learning or robotic applications |
| 63 | Nabil MA, Akram A, Fathalla KM. Applying machine learning on home videos for remote autism diagnosis: Further study and analysis. Health Inform J. 2021;27(1):1460458221991882. | Excluded | Explored virtual reality, machine learning or robotic applications |
| 64 | Gibson KA. Appreciating the world of autism through the lens of video interaction guidance: An exploration of a parent's perceptions, experiences and emerging narratives on autism: Erratum. Disabil Soc. 2014;29(8):vi–vi. | Excluded | Did not examine relevant outcomes |
| 65 | McDonald TAM, Lalani S, Chen I, Cotton CM, MacDonald L, Boursoulian LJ, et al. Appropriateness, acceptability, and feasibility of a neurodiversity-based self-determination program for autistic adults. J Autism Dev Disord. 2023;53(8):2933–53. | Excluded | Examined/ elaborated on an intervention |
| 66 | Sabzevari F, Amelirad O, Moradi Z, Habibi M. Artificial intelligence evaluation of COVID-19 restrictions and speech therapy effects on the autistic children’s behavior. Sci Rep. 2023;13(1):4312. | Excluded | Examined/ elaborated on an intervention |
| 67 | Brett JD, Peden B, Preece DA, Whitehouse A, Becerra R, Maybery MT. Assessing restricted and repetitive behaviours in online-sampled autistic and non-autistic individuals: Factor structure of the Repetitive Behaviours Questionnaire for Adults (RBQ-2A). J Autism Dev Disord [Internet]. 2023. Available from: <https://www.springer.com/journal/10803> | Excluded | Non-ASD sample |
| 68 | Clifford S, Young R, Williamson P. Assessing the early characteristics of autistic disorder using video analysis. J Autism Dev Disord. 2007;37(2):301–13. | Excluded | Did not examine relevant outcomes |
| 69 | Morris AC, Ibrahim Z, Heslin M, Moghraby OS, Stringaris A, Grant IM, et al. Assessing the feasibility of a web-based outcome measurement system in child and adolescent mental health services - myHealthE: A randomised controlled feasibility pilot study. Child Adolesc Ment Health. 2023;28(1):128–47. | Excluded | Did not examine relevant outcomes |
| 70 | Nohelty K, Hahs AD, Rodriguez KA, Rue H, Cameron MJ, Dixon DR. Assessing the social validity of telehealth-based applied behavior analysis services for autism spectrum disorder. Behav Interv. 2023;38(3):590–610. | Excluded | Examined/ elaborated on an intervention |
| 71 | Ha VS, Whittaker A, Rodger S. Assessment and diagnosis of autism spectrum disorder in Hanoi, Vietnam. J Child Fam Stud. 2017;26(5):1334–44. | Excluded | Did not explore telehealth applications |
| 72 | Yaholkoski A, Montgomery JMN, Stoesz B, Piotrowski A. Assessment and diagnostic practices for autism spectrum disorders: A survey of clinicians in Canada. J Dev Disabil. 2021;26(1):1–20. | Excluded | Did not explore telehealth applications |
| 73 | Phillips AQ, Campi E, Talbott MR, Baranek GT. Assessment fidelity of parents implementing a standardized telehealth infant autism screener. OTJR (Thorofare N J). 2023;15394492231164943. | Excluded | Did not examine relevant outcomes |
| 74 | Arnold LE, Aman MG, Martin A, Collier-Crespin A, Vitiello B, Tierney E, et al. Assessment in multisite randomized clinical trials of patients with autistic disorder: The Autism RUPP Network. J Autism Dev Disord. 2000;30(2):99–111. | Excluded | Did not explore telehealth applications |
| 75 | Doyen C, Desailly E, Goupil V, Kaye K. Assessment of a tele-diagnosis experimentation of autism spectrum disorders. Dev Med Child Neurol. 2019;61:24. | Excluded | Conference abstract |
| 76 | Minissi ME, Chicchi Giglioli IA, Mantovani F, Alcañiz Raya M. Assessment of the autism spectrum disorder based on machine learning and social visual attention: A systematic review. J Autism Dev Disord. 2022;52(5):2187–202. | Excluded | Explored virtual reality, machine learning or robotic applications |
| 77 | Brito A, Franco F, Brentani H, Beltrao-Braga PCB. Assessment of vulnerability dimensions considering family history and environmental interplay in autism spectrum disorder. BMC Psychiatry. 2023;23(1):254. | Excluded | Did not explore telehealth applications |
| 78 | Wohofsky L, Scharf P, Lattacher SL, Krainer D. Assistive technology to support people with autism spectrum disorder in their autonomy and safety: A scoping review. Technol Disabil. 2022;34(1):1–11. | Excluded | Did not explore telehealth applications |
| 79 | O’Neill SJ, Smyth S, Smeaton A, O’Connor NE. Assistive technology: Understanding the needs and experiences of individuals with autism spectrum disorder and/or intellectual disability in Ireland and the UK. Assist Technol. 2020;32(5):251–9. | Excluded | Did not explore telehealth applications |
| 80 | Myers K, Roth DE. Asynchronous telehealth technologies to improve the quality of children's mental health care. J Am Acad Child Adolesc Psychiatry. 2016;55(10):S31. | Excluded | Non-ASD sample |
| 81 | Pervin M, Hagmayer Y. Attitudes towards evidence-based practice of professionals working with children and adolescents with autism spectrum disorder in Bangladesh. Adm Policy Ment Health. 2022;49(5):861–80. | Excluded | Did not explore telehealth applications |
| 82 | Dawson G, Campbell K, Hashemi J, Lippmann SJ, Smith V, Carpenter K, et al. Atypical postural control can be detected via computer vision analysis in toddlers with autism spectrum disorder. Sci Rep. 2018;8(1):17008. | Excluded | Explored virtual reality, machine learning or robotic applications |
| 83 | Nadler C, Godwin DL, Dempsey J, Nyp SS. Autism and access to care during the COVID-19 crisis. J Dev Behav Pediatr. 2021;42(1):73–5. | Excluded | Case study |
| 84 | Lewis LF. Autism as a difference or a disorder? Exploring the views of individuals who use peer-led online support groups for autistic partners. Autism. 2023;27(2):321–30. | Excluded | Did not explore telehealth applications |
| 85 | Gibbs V, Cai RY, Aldridge F, Wong M. Autism assessment via telehealth during the COVID-19 pandemic: Experiences and perspectives of autistic adults, parents/carers and clinicians. Res Autism Spectr Disord. 2021;88:101859. | Included |  |
| 86 | Crane L, Batty R, Hanna A, Goddard L, Henry LA, Hill EL. Autism diagnosis in the United Kingdom: Perspectives of autistic adults, parents and professionals. J Autism Dev Disord. 2018;48(11):3761–76. | Excluded | Did not explore telehealth applications |
| 87 | Spain D, Stewart GR, Mason D, Robinson J, Capp SJ, Gillan N, et al. Autism diagnostic assessments with children, adolescents, and adults prior to and during the COVID-19 pandemic: A cross-sectional survey of professionals. Front Psychiatry. 2022;13:789449. | Included |  |
| 88 | Garnett MS, Attwood T, Peterson C, Kelly AB. Autism spectrum conditions among children and adolescents: A new profiling tool. Aust J Psychol. 2013;65(4):206–13. | Excluded | Did not explore telehealth applications |
| 89 | Yuan J, Holtz C, Smith T, Luo J. Autism spectrum disorder detection from semi-structured and unstructured medical data. Eurasip J Bioinform Syst Biol. 2017;2017(1):3. | Excluded | Did not explore telehealth applications |
| 90 | Gautam K. Autism spectrum disorder: The parental experience. J Psychosoc Nurs Ment Health Serv. 2020;58(2):14–9. | Excluded | Did not explore telehealth applications |
| 91 | Hwang SK, Heslop P. Autistic parents' personal experiences of parenting and support: Messages from an online focus group. Br J Soc Work. 2023;53(1):276–95. | Excluded | Did not explore telehealth applications |
| 92 | Lin Y, Gu Y, Xu Y, Hou S, Ding R, Ni S. Autistic spectrum traits detection and early screening: A machine learning based eye movement study. J Child Adolesc Psychiatr Nurs. 2022;35(1):83–92. | Excluded | Explored virtual reality, machine learning or robotic applications |
| 93 | Schweinberger SR, Pohl M, Winkler P. Autistic traits, personality, and evaluations of humanoid robots by young and older adults. Comput Human Behav [Internet]. 2020;106. Available from: <https://search.ebscohost.com/login.aspx?direct=true&db=psyh&AN=2020-11391-001&site=ehost-live> | Excluded | Did not explore telehealth applications |
| 94 | Adams L, Adamo N, Hollocks MJ, Watson J, Brewster A, Valmaggia L, et al. Autistic young people's experiences of remote psychological interventions during COVID-19. Autism. 2023;27(6):1616–27. | Excluded | Intervention study |
| 95 | Khurana A, Verma OP. AviR: Autism rehabilitation with webVR using text classification. In: Enabling Technology for Neurodevelopmental Disorders: From Diagnosis to Rehabilitation [Internet]. New York (NY): Routledge; 2022. p. 184–93. Available from: <https://search.ebscohost.com/login.aspx?direct=true&db=psyh&AN=2023-36284-012&site=ehost-live> | Excluded | Book chapter |
| 96 | Doherty A, Hastings R, Hood K, James-Jenkinson L, Leavey R, Randell E, et al. Barriers and facilitators to primary health care for physical and/or mental health issues experienced by adolescents and adults with intellectual disabilities (only), autism (only), or both: An integrative review. J Intellect Disabil Res. 2019;63(7):873–94. | Excluded | Did not explore telehealth applications |
| 97 | Featherstone C, Sharpe RA, Axford N, Asthana S, Ball S, Husk K. Barriers to healthcare and their relationship to well-being and social support for autistic adults during COVID-19. Prim Health Care Res Dev [Internet]. 2022;23. Available from: <https://search.ebscohost.com/login.aspx?direct=true&db=psyh&AN=2023-30313-001&site=ehost-live> | Excluded | Adult sample |
| 98 | Oberleitner R. Behavior imaging for autism spectrum disorder diagnosis, assessment, and treatment monitoring. J Am Acad Child Adolesc Psychiatry. 2016;55(10):S32–3. | Excluded | Did not examine relevant outcomes |
| 99 | Schieltz KM, O’Brien MJ, Tsami L, Call NA, Lerman DC. Behavioral assessment and treatment via telehealth for children with autism: From local to global clinical applications. Int J Environ Res Public Health. 2022;19(4):2297. | Excluded | Examined/ elaborated on an intervention |
| 100 | Machalicek W, Lequia J, Pinkelman S, Knowles C, Raulston T, Davis T, et al. Behavioral telehealth consultation with families of children with autism spectrum disorder. Behav Interv. 2016;31(3):223–50. | Excluded | Examined/ elaborated on an intervention |
| 101 | Hassrick EM, Holmes LG, Sosnowy C, Walton J, Carley K. Benefits and risks: A systematic review of information and communication technology use by autistic people. Autism Adulthood. 2021;3(1):72–84. | Excluded | Review study |
| 102 | Brosnan M, Holt S, Yuill N, Good J, Parsons S. Beyond autism and technology: Lessons from neurodiverse populations. J Enabling Technol. 2017;11(2):43–8. | Excluded | Did not explore telehealth applications |
| 103 | Goldstein FP, Klaiman C, Williams S. Bridging care gaps: Using tele-health to provide care for people with autism spectrum disorder. Int J Dev Disabil. 2017;63(4):190–4. | Excluded | Review study |
| 104 | Kumazaki H, Muramatsu T, Yoshikawa Y, Yoshimura Y, Ikeda T, Hasegawa C, et al. Brief report: A novel system to evaluate autism spectrum disorders using two humanoid robots. J Autism Dev Disord. 2019;49(4):1709–16. | Excluded | Explored virtual reality, machine learning or robotic applications |
| 105 | Taylor LJ, Eapen V, Maybery M, Midford S, Paynter J, Quarmby L, et al. Brief report: An exploratory study of the diagnostic reliability for autism spectrum disorder. J Autism Dev Disord. 2017;47(5):1551–8. | Excluded | Did not explore telehealth applications |
| 106 | Kumazaki H, Warren Z, Swanson A, Yoshikawa Y, Matsumoto Y, Yoshimura Y, et al. Brief report: Evaluating the utility of varied technological agents to elicit social attention from children with autism spectrum disorders. J Autism Dev Disord. 2019;49(4):1700–8. | Excluded | Explored virtual reality, machine learning or robotic applications |
| 107 | Gerber AH, McCormick CEB, Levine TP, Morrow EM, Anders TF, Sheinkopf SJ. Brief report: Factors influencing healthcare satisfaction in adults with autism spectrum disorder. J Autism Dev Disord. 2017;47(6):1896–904. | Excluded | Did not explore telehealth applications |
| 108 | Casey WL, Kiely LJ, Daniels AM, Toroney J, Vernoia B, Xiao S, et al. Brief report: Impact of COVID-19 on individuals with ASD and their caregivers: A perspective from the SPARK cohort. J Autism Dev Disord. 2021;51(10):3766–73. | Excluded | Did not explore telehealth applications |
| 109 | Dababnah S, Kim I, Wang Y, Reyes C. Brief report: Impact of the COVID-19 pandemic on Asian American families with children with developmental disabilities. J Dev Phys Disabil. 2022;34(3):491–504. | Excluded | Did not explore telehealth applications |
| 110 | Talbott MR, Dufek S, Zwaigenbaum L, Bryson S, Brian J, Smith IM, et al. Brief report: Preliminary feasibility of the TEDI: A novel parent-administered telehealth assessment for autism spectrum disorder symptoms in the first year of life. J Autism Dev Disord. 2020;50(9):3432–9. | Included |  |
| 111 | Koehler JC, Georgescu AL, Weiske J, Spangemacher M, Burghof L, Falkai P, et al. Brief report: Specificity of interpersonal synchrony deficits to autism spectrum disorder and its potential for digitally assisted diagnostics. J Autism Dev Disord. 2022;52(8):3718–26. | Excluded | Adult sample |
| 112 | Rosen V, Blank E, Lampert E, Dominick K, Will M, Erickson C, et al. Brief report: Telehealth satisfaction among caregivers of pediatric and adult psychology and psychiatry patients with intellectual and developmental disability in the wake of COVID-19. J Autism Dev Disord. 2022;52(12):5253–65. | Excluded | No relevant data |
| 113 | Reese RM, Jamison TR, Braun M, Wendland M, Black W, Hadorn M, et al. Brief report: Use of interactive television in identifying autism in young children: Methodology and preliminary data. J Autism Dev Disord. 2015;45(5):1474–82. | Excluded | Did not examine relevant outcomes |
| 114 | Kelleher BL, Halligan T, Witthuhn N, Neo WS, Hamrick L, Abbeduto L. Bringing the laboratory home: PANDABox telehealth-based assessment of neurodevelopmental risk in children. Front Psychol [Internet]. 2020;11. Available from: <https://search.ebscohost.com/login.aspx?direct=true&db=psyh&AN=2020-59836-001&site=ehost-live> | Excluded | Not assessing for ASD |
| 115 | McGinnis K, Gerow S, Gregori E, Davis T. Caregiver-implemented interventions for autistic adolescents and young adults: A systematic literature review. J Dev Phys Disabil. 2023;35(2):167–88. | Excluded | Examined/ elaborated on an intervention |
| 116 | Angell AM, Carreon ED, Akrofi JNS, Franklin MD, Taylor EE, Miller J, et al. Challenges and facilitators to telehealth occupational therapy for autistic children during COVID-19. OTJR Occup Particip Health. 2023;43(3):513–22. | Excluded | Examined/ elaborated on an intervention |
| 117 | Berger NI, Wainer AL, Kuhn J, Bearss K, Attar S, Carter AS, et al. Characterizing available tools for synchronous virtual assessment of toddlers with suspected autism spectrum disorder: A brief report. J Autism Dev Disord. 2022;52(1):423–34. | Excluded | Review study |
| 118 | Koumpouros Y, Kafazis T. Wearables and mobile technologies in autism spectrum disorder interventions: A systematic literature review. Res Autism Spectr Disord. 2019;66:101405. | Excluded | Explored virtual reality, machine learning or robotic applications |
| 119 | Huang Y, Arnold SRC, Foley KR, Trollor JN. Choose your own adventure: Pathways to adulthood autism diagnosis in Australia. J Autism Dev Disord. 2022;52(7):2984–96. | Excluded | Did not explore telehealth applications |
| 120 | Crepeau-Hobson MF, Leech N, Russell C. CLEAR Autism Diagnostic Evaluation (CADE): Evaluation of reliability and validity. J Dev Phys Disabil. 2022;34(5):853–69. | Excluded | Did not explore telehealth applications |
| 121 | Vanegas SB, Xu Y, Magaña S, Heller T. “You had this clean window, but it was glued shut”: Identifying the needs of parents and providers of children and youth with autism spectrum disorders in rural areas through a life course perspective. Journal of Developmental and Physical Disabilities. 2023;35(2):247–71. | Excluded | Did not explore telehealth applications |
| 122 | Singleton G, Warren S, Piersel W. Clinical overview of the need for technologies for around-the-clock monitoring of the health status of severely disabled autistic children. Annu Int Conf IEEE Eng Med Biol Soc. 2014;2014:789–91. | Excluded | Did not explore telehealth applications |
| 123 | Clinical update: Telepsychiatry with children and adolescents. J Am Acad Child Adolesc Psychiatry. 2017;56(10):875–93. | Excluded | Did not examine relevant outcomes |
| 124 | Kryszak EM, Albright CM, Fell LA, Butter EM, Kuhlthau KA. Clinician perspectives on telehealth assessment of autism spectrum disorder during the COVID-19 pandemic. J Autism Dev Disord. 2022;52(12):5083–98. | Included |  |
| 125 | Taylor H, Ingham B, Mason D, Finch T, Wilson C, Scarlett C, et al. Co-design of an NHS primary care health check for autistic adults. Autism. 2023;27(4):1079–91. | Excluded | Did not explore telehealth applications |
| 126 | Lee JD, Yoon CD, Meadan H. Coaching caregivers of young children with autism via telepractice to collect assessment data and implement interventions. Perspect ASHA Spec Interest Groups. 2022;7(2):338–45. | Excluded | Examined/ elaborated on an intervention |
| 127 | Kunze MG. Coaching via telehealth: Caregiver-mediated interventions for young children on the waitlist for an autism diagnosis [Internet]. Vol. 82, Dissertation Abstracts International: Section B: The Sciences and Engineering. ProQuest Information & Learning; 2021. Available from: <https://search.ebscohost.com/login.aspx?direct=true&db=psyh&AN=2020-97492-254&site=ehost-live> | Excluded | Examined/ elaborated on an intervention |
| 128 | Bishop SL, Lord C. Commentary: Best practices and processes for assessment of autism spectrum disorder – the intended role of standardized diagnostic instruments. J Child Psychol Psychiatry. 2023;64(5):834–8. | Excluded | Commentary or editorial |
| 129 | Blackmore CE, Nolan A, Stoencheva V, Greenwood N, Liu-Thwaites N, Maltezos S, et al. Comparison of an online adaptation of the Autism Diagnostic Observation Schedule-2 with its in-person version in an adult autism diagnostic service. BJPsych Open. 2023;9(2):e51. | Excluded | Adult sample |
| 130 | Finkelman MD, Wang T, Lowe SR. Computer-based testing to shorten the Social Communication Questionnaire (SCQ): A proof-of-principle study of the lifetime and current forms. J Psychopathol Behav Assess. 2021;43(2):427–40. | Excluded | Did not explore telehealth applications |
| 131 | Mezher KR, Shahidullah JD, McClain MB. Conclusions and future directions in interprofessional care coordination for pediatric autism spectrum disorder. In: Mezher KR, editor. Interprofessional Care Coordination for Pediatric Autism Spectrum Disorder: Translating Research into Practice [Internet]. Cham: Springer Nature Switzerland AG; 2020. p. 459–62. Available from: <https://search.ebscohost.com/login.aspx?direct=true&db=psyh&AN=2022-06567-027&site=ehost-live> | Excluded | Book chapter |
| 132 | Wacker DP, Lee JF, Dalmau YCP, Kopelman TG, Lindgren SD, Kuhle J, et al. Conducting functional analyses of problem behavior via telehealth. J Appl Behav Anal. 2013;46(1):31–46. | Excluded | Examined/ elaborated on an intervention |
| 133 | Wacker DP, Lee JF, Dalmau YCP, Kopelman TG, Lindgren SD, Kuhle J, et al. Conducting functional communication training via telehealth to reduce the problem behavior of young children with autism. J Dev Phys Disabil. 2013;25(1):35–48. | Excluded | Examined/ elaborated on an intervention |
| 134 | Whittingham LM, Coons-Harding KD. Connecting people with people: Diagnosing persons with fetal alcohol spectrum disorder using telehealth. J Autism Dev Disord. 2021;51(4):1067–80. | Excluded | Non-ASD sample |
| 135 | Shaw SCK, Davis LJ, Doherty M. Considering autistic patients in the era of telemedicine: The need for an adaptable, equitable, and compassionate approach. BJGP Open. 2022;6(1):BJGPO.2021.0167. | Excluded | Theoretical paper |
| 136 | Kaku S, Chandran S, Roopa N, Choudhary A, Ramesh J, Somashekariah S, et al. Coping with autism during lockdown period of the COVID-19 pandemic: A cross-sectional survey. Indian J Psychiatry. 2021;63(6):568–74. | Excluded | Did not explore telehealth applications |
| 137 | Buonaguro EF, Bertelli MO. COVID-19 and intellectual disability/autism spectrum disorder with high and very high support needs: Issues of physical and mental vulnerability. Adv Ment Health Intellect Disabil. 2021;15(1):8–19. | Excluded | Did not explore telehealth applications |
| 138 | Raman B, Brown SL, Edwards EM, Murray MJ. COVID-19 pandemic and impact on patients with autism spectrum disorder. J Autism Dev Disord. 2022;52(1):473–82. | Excluded | Did not explore telehealth applications |
| 139 | Camoni N, Cirio S, Salerno C, Balian A, Bruni G, D’Avola V, et al. COVID-19 pandemic and remote consultations in children: A bibliometric analysis. Int J Environ Res Public Health. 2022;19(16):9787. | Excluded | Non-ASD sample |
| 140 | Keehn RM, Tomlin A, Ciccarelli MR. COVID-19 pandemic highlights access barriers for children with autism spectrum disorder. J Dev Behav Pediatr. 2021;42(7):599–601. | Excluded | Commentary or editorial |
| 141 | McFayden TC, Breaux R, Bertollo JR, Cummings K, Ollendick TH. COVID-19 remote learning experiences of youth with neurodevelopmental disorders in rural Appalachia. Rural Ment Health. 2021;45(2):72–85. | Excluded | Did not explore telehealth applications |
| 142 | Pellicano E, den HJ, Heyworth M, Magiati I, Steward R, et al. COVID-19, social isolation and the mental health of autistic people and their families: A qualitative study. Autism. 2022;26(4):914–27. | Excluded | Did not explore telehealth applications |
| 143 | Dawson G, de Vries PJ, Franz L, Howard J. COVID‐19 and autism research: Perspectives from around the globe. Autism Res. 2020;13(6):854–5. | Excluded | Commentary or editorial |
| 144 | Johnsson G, Kerslake R, Crook S. Delivering allied health services to regional and remote participants on the autism spectrum via video-conferencing technology: Lessons learned. Rural Remote Health. 2019;19(3):5358. | Excluded | No relevant data |
| 145 | Williams RM, Alikhademi K, Gilbert JE. Design of a toolkit for real-time executive function assessment in custom-made virtual experiences and interventions. Int J Hum Comput Stud [Internet]. 2022;158:102747. Available from: <https://search.ebscohost.com/login.aspx?direct=true&db=psyh&AN=2022-12474-001&site=ehost-live> | Excluded | Explored virtual reality, machine learning or robotic applications |
| 146 | Tariq Q, Fleming SL, Schwartz JN, Dunlap K, Corbin C, Washington P, et al. Detecting developmental delay and autism through machine learning models using home videos of Bangladeshi children: Development and validation study. J Med Internet Res. 2019;21(4):e13822. | Excluded | Explored virtual reality, machine learning or robotic applications |
| 147 | Guillon Q, Baduel S, Bejarano-Martín Á, Canal-Bedia R, Magán-Maganto M, Fernández-Álvarez C, et al. Determinants of satisfaction with the detection process of autism in Europe: Results from the ASDEU study. Autism. 2022;26(8):2136–50. | Excluded | Did not explore telehealth applications |
| 148 | Watson L, Jonathan S. Developing a tool-kit for assessment of autism spectrum disorder. Int J Technol Assess Health Care. 2021;37:e12. | Excluded | Conference abstract |
| 149 | Jonathan S, Watson L. Developing a tool-kit for the assessment of autism spectrum disorder for children under 5 years. Arch Dis Child. 2021;106:A276–7. | Excluded | Conference abstract |
| 150 | Bates K, Morgan H, Crosby E, Nurse K, Flynn A, Stern D, et al. Developing digital approaches for adolescents and young adults with autism and learning disabilities: Tools to facilitate access and shared decision-making. Educ Child Psychol. 2021;38(3):124–39. | Excluded | No relevant data |
| 151 | Codd A, Burford B, Petruso G, Davidson N, Vance G. Development and evaluation of a digistory about autistic spectrum disorder: A pilot study. Educ Prim Care. 2018;29(4):232–6. | Excluded | Did not explore telehealth applications |
| 152 | Margolis KG, Buie TM, Turner JB, Silberman AE, Feldman JF, Murray KF, et al. Development of a brief parent-report screen for common gastrointestinal disorders in autism spectrum disorder. J Autism Dev Disord. 2019;49(1):349–62. | Excluded | Did not explore telehealth applications |
| 153 | Rutherford M, Maciver D, Johnston L, Prior S, Forsyth K. Development of a pathway for multidisciplinary neurodevelopmental assessment and diagnosis in children and young people. Children (Basel). 2021;8(11):1033. | Excluded | Did not explore telehealth applications |
| 154 | Herrera G, Alcantud F, Jordan R, Blanquer A, Labajo G, De Pablo C. Development of symbolic play through the use of virtual reality tools in children with autistic spectrum disorders: Two case studies. Autism. 2008;12(2):143–57. | Excluded | Examined/ elaborated on an intervention |
| 155 | Frazier TW, Busch RM, Klaas P, Lachlan K, Jeste S, Kolevzon A, et al. Development of webcam-collected and artificial-intelligence-derived social and cognitive performance measures for neurodevelopmental genetic syndromes. Am J Med Genet C Semin Med Genet. 2023;193(3):e32058. | Excluded | Non-ASD sample |
| 156 | McEwen FS, Stewart CS, Colvert E, Woodhouse E, Curran S, Gillan N, et al. Diagnosing autism spectrum disorder in community settings using the Development and Well-Being Assessment: Validation in a UK population-based twin sample. J Child Psychol Psychiatry. 2016;57(2):161–70. | Excluded | Did not examine relevant outcomes |
| 157 | Carpenter P. Diagnosis and assessment in autism spectrum disorders. Adv Ment Health Intellect Disabil. 2012;6(3):121–9. | Excluded | Did not explore telehealth applications |
| 158 | Coret M, Yohemas M, Goldade R, Gibbard B. Diagnostic and collaboration needs regarding ASD by community paediatricians in Southern Alberta. Paediatr Child Health. 2018;23:e35–41. | Excluded | Did not explore telehealth applications |
| 159 | Gargot T. Diagnostic automated algorithms in neurodevelopmental disorders: Focus on automatic motor assessment. Eur Psychiatry. 2021;64(S1):S18–9. | Excluded | Explored virtual reality, machine learning or robotic applications |
| 160 | Jang J, White SP, Esler AN, Kim SH, Klaiman C, Megerian JT, et al. Diagnostic evaluations of autism spectrum disorder during the COVID-19 pandemic. J Autism Dev Disord. 2022;52(2):962–73. | Excluded | Theoretical paper |
| 161 | Falkmer T, Anderson K, Falkmer M, Horlin C. Diagnostic procedures in autism spectrum disorders: A systematic literature review. Eur Child Adolesc Psychiatry. 2013;22(6):329–40. | Excluded | Did not explore telehealth applications |
| 162 | Carpenter KLH, Hahemi J, Campbell K, Lippmann SJ, Baker JP, Egger HL, et al. Digital behavioral phenotyping detects atypical pattern of facial expression in toddlers with autism. Autism Res. 2021;14(3):488–95. | Excluded | Explored virtual reality, machine learning or robotic applications |
| 163 | Khanlou N, Khan A, Vazquez LM, Zangeneh M. Digital literacy, access to technology and inclusion for young adults with developmental disabilities. J Dev Phys Disabil. 2021;33(1):1–25. | Excluded | Did not explore telehealth applications |
| 164 | Mukherjee D, Bhavnani S, Lockwood Estrin G, Rao V, Dasgupta J, Irfan H, et al. Digital tools for direct assessment of autism risk during early childhood: A systematic review. Autism [Internet]. 2022. Available from: <https://journals.sagepub.com/home/AUT> | Excluded | Review study |
| 165 | Koehler JC, Falter-Wagner CM. Digitally assisted diagnostics of autism spectrum disorder. Front Psychiatry. 2023;14:1066284. | Excluded | Review study |
| 166 | Washington P. Digitally diagnosing multiple developmental delays using crowdsourcing fused with machine learning: A research protocol. medRxiv [Internet]. 2023. Available from: <https://www.medrxiv.org/> | Excluded | Theoretical paper |
| 167 | Uljarević M, Frazier TW, Jo B, Scahill L, Youngstrom EA, Spackman E, et al. Dimensional assessment of restricted and repetitive behaviors: Development and preliminary validation of a new measure. J Am Acad Child Adolesc Psychiatry. 2023;62(5):568–77. | Excluded | Did not explore telehealth applications |
| 168 | Dutheil F, Chambres P, Hufnagel C, Auxiette C, Chausse P, Ghozi R, et al. ‘Do Well B.’: Design of WELL Being monitoring systems. A study protocol for the application in autism. BMJ Open. 2015;5(2):e007716. | Excluded | Did not examine relevant outcomes |
| 169 | Xia C, Zhang D, Li K, Li H, Chen J, Min W, et al. Dynamic viewing pattern analysis: Towards large-scale screening of children with ASD in remote areas. IEEE Trans Biomed Eng. 2023;70(5):1622–33. | Excluded | Conference abstract |
| 170 | Perochon S, Di Martino JM, Carpenter KLH, Compton S, Davis N, Eichner B, et al. Early detection of autism using digital behavioral phenotyping. Nat Med [Internet]. Available from: <https://www.nature.com/nm/> | Excluded | Explored virtual reality, machine learning or robotic applications |
| 171 | Thorsen KL. Early detection of autism: An evaluation of a parent-training intervention [Internet]. Vol. 73, Dissertation Abstracts International: Section B: The Sciences and Engineering. ProQuest Information & Learning; 2012. Available from: <https://search.ebscohost.com/login.aspx?direct=true&db=psyh&AN=2012-99200-032&site=ehost-live> | Excluded | Examined/ elaborated on an intervention |
| 172 | Denis F, Maurier L, Carillo K, Ologeanu-Taddei R, Septans AL, Gepner A, et al. Early detection of neurodevelopmental disorders of toddlers and postnatal depression by mobile health app: Observational cross-sectional study. JMIR Mhealth Uhealth. 2022;10(5):e38181. | Excluded | Examined a mobile application |
| 173 | Bejarano-Martín Á, Canal-Bedia R, Magán-Maganto M, Fernández-Álvarez C, Cilleros-Martín MV, Sánchez-Gómez MC, et al. Early detection, diagnosis and intervention services for young children with autism spectrum disorder in the European Union (ASDEU): Family and professional perspectives. J Autism Dev Disord. 2020;50(9):3380–94. | Excluded | Did not explore telehealth applications |
| 174 | Juárez AP, Weitlauf AS, Nicholson A, Pasternak A, Broderick N, Hine J, et al. Early identification of ASD through telemedicine: Potential value for underserved populations. J Autism Dev Disord. 2018;48(8):2601–10. | Included |  |
| 175 | Shaw KA, Bilder DA, McArthur D, Williams AR, Amoakohene E, Bakian AV, et al. Early identification of autism spectrum disorder among children aged 4 years - Autism and Developmental Disabilities Monitoring Network, 11 Sites, United States, 2020. MMWR Surveill Summ. 2023;72(1):1–15. | Excluded | Did not explore telehealth applications |
| 176 | Stone WL, Ibanez LV, Carpentier P, Posner E, Bravo A, Lindsay F, et al. Early intervention providers’ perspectives about working with families of toddlers with suspected ASD: A qualitative study. J Autism Dev Disord. 2021;51(3):814–26. | Excluded | Did not explore telehealth applications |
| 177 | Mazurek MO, Curran A, Burnette C, Sohl K. ECHO Autism STAT: Accelerating early access to autism diagnosis. J Autism Dev Disord. 2018;48(9):3081–90. | Excluded | Did not explore telehealth applications |
| 178 | Buranova N, Dampf M, Stevenson B, Sohl K. ECHO Autism: Early intervention connecting community professionals to increase access to best practice autism intervention. Clin Pediatr (Phila). 2022;61(8):518–22. | Excluded | Examined/ elaborated on an intervention |
| 179 | Øien RA, Vivanti G, Robins DL. Editorial S.I: Early identification in autism spectrum disorders: The present and future, and advances in early identification. J Autism Dev Disord. 2021;51(3):763–8. | Excluded | Commentary or editorial |
| 180 | Kaur R, Boobna T, Kallingal P. Effect of COVID-19 lockdown on Indian children with autism. Res Dev Disabil. 2022;125:104230. | Excluded | No relevant data |
| 181 | Lopata C, Rodgers JD, Donnelly JP, Thomeer ML, Lodi-Smith J, Gionis ZL, et al. Effect of COVID-19 stay-at-home restrictions on parent-reported symptom severity and adaptive functioning of youth with ASD. J Dev Phys Disabil. 2022;34(3):459–70. | Excluded | Did not explore telehealth applications |
| 182 | Tsami L, Lerman D, Toper‐Korkmaz O. Effectiveness and acceptability of parent training via telehealth among families around the world. J Appl Behav Anal. 2019;52(4):1113–29. | Excluded | Examined/ elaborated on an intervention |
| 183 | Mesa-Gresa P, Gil-Gomez H, Lozano-Quilis JA, Gil-Gomez JA. Effectiveness of virtual reality for children and adolescents with autism spectrum disorder: An evidence-based systematic review. Sensors (Basel). 2018;18(8):2486. | Excluded | Review study |
| 184 | Wendelken ME. Effects of an asynchronous, online training program for parents of children with autism using augmentative and alternative communication [Internet]. Vol. 84, Dissertation Abstracts International: Section B: The Sciences and Engineering. ProQuest Information & Learning; 2023. Available from: <https://search.ebscohost.com/login.aspx?direct=true&db=psyh&AN=2023-85471-201&site=ehost-live> | Excluded | Examined/ elaborated on an intervention |
| 185 | Sheehan R, Dalton-Locke C, Ali A, Vera San Juan N, Totsika V, Hassiotis A. Effects of the COVID-19 pandemic on mental healthcare and services: Results of a UK survey of front-line staff working with people with intellectual disability and/or autism. BJPsych Bull. 2022;46(4):201–7. | Excluded | Did not explore telehealth applications |
| 186 | Mineo BA, Ziegler W, Gill S, Salkin D. Engagement with electronic screen media among students with autism spectrum disorders. J Autism Dev Disord. 2009;39(1):172–87. | Excluded | Explored virtual reality, machine learning or robotic applications |
| 187 | Blumling AA, Brasher S, Stapel-Wax J. Engaging parents of children with autism spectrum disorder to identify rural health disparities and factors related to delayed diagnosis and treatment. Int J Child Health Hum Dev. 2019;12(4):379–89. | Excluded | Did not explore telehealth applications |
| 188 | Kollins SH, Frank E, Torous J, Resnick K, Foxworth P, Kanady J, et al. Ethical issues in the use of digital technology for the assessment and treatment of psychiatric conditions. Neuropsychopharmacology. 2019;44:70–5. | Excluded | Conference abstract |
| 189 | Harris B, Barton EE, Albert C. Evaluating autism diagnostic and screening tools for cultural and linguistic responsiveness. J Autism Dev Disord. 2014;44(6):1275–87. | Excluded | Did not explore telehealth applications |
| 190 | Reese RM, Jamison R, Wendland M, Fleming K, Braun MJ, Schuttler JO, et al. Evaluating interactive videoconferencing for assessing symptoms of autism. Telemed J E Health. 2013;19(9):671–7. | Included |  |
| 191 | Apikos LD. Evaluating parent motivation to complete applied behavioral analysis training delivered as video modeling [Internet]. Vol. 82, Dissertation Abstracts International: Section A: Humanities and Social Sciences. ProQuest Information & Learning; 2021. Available from: <https://search.ebscohost.com/login.aspx?direct=true&db=psyh&AN=2020-86251-114&site=ehost-live> | Excluded | Examined/ elaborated on an intervention |
| 192 | Coster WJ, Kramer JM, Tian F, Dooley M, Liljenquist K, Kao YC, et al. Evaluating the appropriateness of a new computer-administered measure of adaptive function for children and youth with autism spectrum disorders. Autism. 2016;20(1):14–25. | Excluded | Did not examine relevant outcomes |
| 193 | Cihon JH, Ferguson JL, Lee M, Leaf JB, Leaf R, McEachin J. Evaluating the Cool Versus Not Cool procedure via telehealth. Behav Anal Pract. 2022;15(1):260–8. | Excluded | Examined/ elaborated on an intervention |
| 194 | Micheletti M, Brukilacchio BH, Hooper-Boyle H, Basiru T, Brinster MI, Ravenscroft S, et al. Evaluating the efficiency and equity of autism diagnoses via telehealth during COVID-19. J Autism Dev Disord [Internet]. 2023. Available from: <https://www.springer.com/journal/10803> | Excluded | Did not examine relevant outcomes |
| 195 | Crane L, Hearst C, Ashworth M, Davies J. Evaluating the online delivery of an autistic-led programme to support newly diagnosed or identified autistic adults. Autism Dev Lang Impairments. 2023;8:1–9. | Excluded | Examined/ elaborated on an intervention |
| 196 | Horwitz N. Evaluation of clinician and parent/carer views of remote autism assessments during COVID-19 pandemic in a London autism assessment service. Arch Dis Child. 2021;106:A124. | Excluded | Conference abstract |
| 197 | Kalb LG, Kramer JM, Goode TD, Black SJ, Klick S, Caoili A, et al. Evaluation of telemental health services for people with intellectual and developmental disabilities: Protocol for a randomized non-inferiority trial. BMC Health Serv Res. 2023;23(1):795. | Excluded | Examined/ elaborated on an intervention |
| 198 | Ogundele MO, Ayyash HF. Evidence-based multidisciplinary assessment and management of children and adolescents with neurodevelopmental disorders. Arch Dis Child. 2019;104:A268. | Excluded | Did not explore telehealth applications |
| 199 | Hume K, Steinbrenner JR, Odom SL, Morin KL, Nowell SW, Tomaszewski B, et al. Evidence-based practices for children, youth, and young adults with autism: Third generation review. J Autism Dev Disord. 2021;51(11):4013–32. | Excluded | Did not explore telehealth applications |
| 200 | Wainer AL, Arnold ZE, Leonczyk C, Valluripalli S, Soorya L. Examining a stepped-care telehealth program for parents of young children with autism: A proof-of-concept trial. Mol Autism. 2021;12(1):32. | Excluded | Examined/ elaborated on an intervention |
| 201 | Adams L, Adamo N, Hollocks MJ, Valmaggia L, Brewster A, Watson J, et al. Examining clinicians’ concerns delivering telemental health interventions directly to autistic individuals during COVID-19. Res Autism Spectr Disord. 2022;94:101956. | Excluded | Examined/ elaborated on an intervention |
| 202 | Gabellone A, Marzulli L, Matera E, Petruzzelli MG, Margari A, Giannico OV, et al. Expectations and concerns about the use of telemedicine for autism spectrum disorder: A cross-sectional survey of parents and healthcare professionals. J Clin Med. 2022;11(12):3294. | Excluded | Did not examine relevant outcomes |
| 203 | Johnsson G, Lincoln M, Bundy A, Bulkeley K. Experience of an interactive, autism-specific online professional development training and support programme delivered to regional and remote areas. Open Learn. 2023;38(2):165–86. | Excluded | Examined/ elaborated on an intervention |
| 204 | Jacques C, Saulnier G, Ethier A, Soulieres I. Experience of autistic children and their families during the pandemic: From distress to coping strategies. J Autism Dev Disord. 2022;52(8):3626–38. | Excluded | Did not explore telehealth applications |
| 205 | Wallis KE, Kellom K, Christiansen A, Cordero L, Flaherty CM, Hah J, et al. Experiences in telemedicine in developmental-behavioral pediatrics: A DBPNet mixed-methods study of providers and caregivers. Pediatrics [Internet]. 2022;149:1–2. Available from: <https://publications.aap.org/pediatrics/article/149/1MeetingAbstractsFebruary2022/78/185690/> | Excluded | Conference abstract |
| 206 | Rogers CL, Goddard L, Hill EL, Henry LA, Crane L. Experiences of diagnosing autism spectrum disorder: A survey of professionals in the United Kingdom. Autism. 2016;20(7):820–31. | Excluded | Did not explore telehealth applications |
| 207 | Jones L, Goddard L, Hill EL, Henry LA, Crane L. Experiences of receiving a diagnosis of autism spectrum disorder: A survey of adults in the United Kingdom. J Autism Dev Disord. 2014;44(12):3033–44. | Excluded | Adult sample |
| 208 | Brugnaro BH, Vieira FN, Fernandes G, de Camargo OK, Fumincelli L, de Campos AC, et al. Exploration of the feasibility of remote assessment of functioning in children and adolescents with developmental disabilities: Parents’ perspectives and related contextual factors. Int J Environ Res Public Health. 2022;19(22):15101. | Excluded | Did not examine relevant outcomes |
| 209 | Zagzoug MS. Exploring acceptance of using an online platform to teach parents of children with autism methods in applied behavior analysis (ABA) [Internet]. Vol. 77, Dissertation Abstracts International: Section B: The Sciences and Engineering. ProQuest Information & Learning; 2017. Available from: <https://search.ebscohost.com/login.aspx?direct=true&db=psyh&AN=2016-53072-065&site=ehost-live> | Excluded | Examined/ elaborated on an intervention |
| 210 | Grazioli S, Crippa A, Rosi E, Candelieri A, Ceccarelli SB, Mauri M, et al. Exploring telediagnostic procedures in child neuropsychiatry: Addressing ADHD diagnosis and autism symptoms through supervised machine learning. Eur Child Adolesc Psychiatry [Internet]. 2023. Available from: <https://www.springer.com/journal/787> | Excluded | Explored virtual reality, machine learning or robotic applications |
| 211 | Stavropoulos KK, Heyman M, Salinas G, Baker E, Blacher J. Exploring telehealth during COVID for assessing autism spectrum disorder in a diverse sample. Psychol Sch. 2022;59(7):1319–34. | Included |  |
| 212 | Legg H, Tickle A, Gillott A, Wilde S. Exploring the experiences of parents whose child has received a diagnosis of autistic spectrum disorder in adulthood. J Autism Dev Disord. 2023;53(1):205–15. | Excluded | Did not explore telehealth applications |
| 213 | Trémaud M, Aguiar YP, Pavani JB, Gepner B, Tardif C. What do digital tools add to classical tools for sociocommunicative and adaptive skills in children with autism spectrum disorder? Annee Psychol. 2021;121(4):361–92. | Excluded | Examined/ elaborated on an intervention |
| 214 | Štětinová K. Factors contributing to caregiver satisfaction with autism spectrum disorder evaluation feedback [Internet]. Vol. 84, Dissertation Abstracts International: Section B: The Sciences and Engineering. ProQuest Information & Learning; 2023. Available from: <https://search.ebscohost.com/login.aspx?direct=true&db=psyh&AN=2023-01890-015&site=ehost-live> | Excluded | Did not explore telehealth applications |
| 215 | Abdeen F. Factors influencing the adoption of immersive virtual reality for individuals with autism spectrum disorder: Parents’ perceptions [Internet]. Vol. 82, Dissertation Abstracts International: Section A: Humanities and Social Sciences. ProQuest Information & Learning; 2021. Available from: <https://search.ebscohost.com/login.aspx?direct=true&db=psyh&AN=2020-67310-218&site=ehost-live> | Excluded | Explored virtual reality, machine learning or robotic applications |
| 216 | Shorey S, Lau LST, Tan JX, Ng ED, Aishworiya R. Families with children with neurodevelopmental disorders during COVID-19: A scoping review. J Pediatr Psychol. 2021;46(5):514–25. | Excluded | Review study |
| 217 | Sheldrick RC, Frenette E, Vera JD, Mackie TI, Martinez-Pedraza F, Hoch N, et al. What drives detection and diagnosis of autism spectrum disorder? Looking under the hood of a multi-stage screening process in early intervention. Journal of Autism and Developmental Disorders. 2019;49(6):2304–19. | Excluded | Did not explore telehealth applications |
| 218 | Denusik L, Servais M, Glista D, Hatherly K, Moodie S, Cardy JO, et al. Families’ experiences in the virtual Hanen More Than Words program during the COVID-19 pandemic. Am J Speech Lang Pathol. 2023;32(2):701–16. | Excluded | Examined/ elaborated on an intervention |
| 219 | Kizildag D, Eilenberg JS, Blakey A, Cardona N, Feinberg E, Broger-Fingert S, et al. Family experiences with the autism developmental evaluation process: Perspectives of immigrant and US-born mothers. J Child Fam Stud. 2023;32(3):926–37. | Excluded | Did not explore telehealth applications |
| 220 | Martinez M, Thomas KC, Williams CS, Christian R, Crais E, Pretzel R, et al. Family experiences with the diagnosis of autism spectrum disorder: System barriers and facilitators of efficient diagnosis. J Autism Dev Disord. 2018;48(7):2368–78. | Excluded | Did not explore telehealth applications |
| 221 | Mandak K, Light J. Family-centered services for children with ASD and limited speech: The experiences of parents and speech-language pathologists. J Autism Dev Disord. 2018;48(4):1311–24. | Excluded | Did not explore telehealth applications |
| 222 | Matthews NL, Skepnek E, Mammen MA, James JS, Malligo A, Lyon A, et al. Feasibility and acceptability of a telehealth model for autism diagnostic evaluations in children, adolescents, and adults. Autism Res. 2021;14(12):2564–79. | Included |  |
| 223 | Chambers NJ, de Vries PJ, Delehanty AD, Wetherby AM. Feasibility of utilizing Autism Navigator® for primary care in South Africa. Autism Res. 2018;11(11):1511–21. | Excluded | Examined/ elaborated on an intervention |
| 224 | Ur Rehman I, Sobnath D, Nasralla MM, Winnett M, Anwar A, Asif W, et al. Features of mobile apps for people with autism in a post-COVID-19 scenario: Current status and recommendations for apps using AI. Diagnostics (Basel). 2021;11(10):1923. | Excluded | Explored virtual reality, machine learning or robotic applications |
| 225 | Haden S. Flexibility is key: Making autism assessments in COVID times and beyond. Arch Dis Child. 2023;108:A365–6. | Excluded | Conference abstract |
| 226 | Heitzman-Powell LS, Buzhardt J, Rusinko LC, Miller TM. Formative evaluation of an ABA outreach training program for parents of children with autism in remote areas. Focus Autism Other Dev Disabl. 2014;29(1):23–38. | Excluded | Examined/ elaborated on an intervention |
| 227 | Graucher T, Sinai-Gavrilov Y, Mor Y, Netzer S, Cohen EY, Levi L, et al. From clinic room to Zoom: Delivery of an evidence-based, parent-mediated intervention in the community before and during the pandemic. J Autism Dev Disord. 2022;52(12):5222–31. | Excluded | Examined/ elaborated on an intervention |
| 228 | Barbaro J, Winata T, Gilbert M, Nair R, Khan F, Lucien A, et al. General practitioners’ perspectives regarding early developmental surveillance for autism within the Australian primary healthcare setting: A qualitative study. BMC Prim Care. 2023;24(1):1–15. | Excluded | Not assessing for ASD |
| 229 | Gilmore D, Harris L, Hanks C, Coury D, Moffatt-Bruce S, Garvin JH, et al. "Giving the patients less work": A thematic analysis of telehealth use and recommendations to improve usability for autistic adults. Autism. 2023;27(4):1132–41. | Excluded | Adult sample |
| 230 | Looi JCL, Allison S, Kisely SR, Pring W, Reay RE, Bastiampillai T. Greatly increased Victorian outpatient private psychiatric care during the COVID-19 pandemic: New MBS-telehealth-item and face-to-face psychiatrist office-based services from April-September 2020. Australas Psychiatry. 2021;29(4):423–9. | Excluded | Non-ASD sample |
| 231 | Choi KR, Becerra-Culqui TA, Tejada G, Coleman KJ, Bhakta B, Knight EA, et al. Habilitative teletherapy for children with autism spectrum disorder: A survey of parents. J Dev Behav Pediatr. 2022;43(8):454–60. | Excluded | Examined/ elaborated on an intervention |
| 232 | Shirwaikar RD, Sarwari I, Najam M, Shama HM. Has machine learning enhanced the diagnosis of autism spectrum disorder? Crit Rev Biomed Eng. 2023;51(1):1–14. | Excluded | Explored virtual reality, machine learning or robotic applications |
| 233 | Nicolaidis C, Raymaker D, McDonald K, Dern S, Ashkenazy E, Boisclair WC, et al. Healthcare disparities experienced by adults on the autistic spectrum. J Gen Intern Med. 2011;26(Suppl 2):S175. | Excluded | Did not explore telehealth applications |
| 234 | Morris R, Greenblatt A, Saini M. Healthcare providers’ experiences with autism: A scoping review. J Autism Dev Disord. 2019;49(6):2374–88. | Excluded | Did not explore telehealth applications |
| 235 | Ecob C, Olety S, Lancaster R, Harris A. Honouring COVID-19 restrictions: A qualitative study of the virtual ASD diagnostic pathway in a UK NHS CAMHS service. Eur Psychiatry. 2021;64(S1):S232–3. | Excluded | Conference abstract |
| 236 | Brosnan M, Parsons S, Good J, Yuill N. How can participatory design inform the design and development of innovative technologies for autistic communities? J Assist Technol. 2016;10(2):115–23. | Excluded | Did not explore telehealth applications |
| 237 | Bain JM, Dyer CA, Galvin M, Goldman S, Selman J, Silver WG, et al. How providers in child neurology transitioned to telehealth during COVID-19 pandemic. Child Neurol Open [Internet]. 2021;8. Available from: <http://www.sagepub.in/journals/Journal202287> | Included |  |
| 238 | Loman M, Vogt E, Miller L, Landsman R, Duong P, Kasten J, et al. ‘How to’ operate a pediatric neuropsychology practice during the COVID-19 pandemic: Real tips from one practice’s experience. Child Neuropsychol. 2021;27(2):251–79. | Excluded | Theoretical paper |
| 239 | Ciccia AH, Roizen N, Garvey M, Bielefeld R, Short EJ. Identification of neurodevelopmental disabilities in underserved children using telehealth (INvesT): Clinical trial study design. Contemp Clin Trials. 2015;45:226–32. | Excluded | Theoretical paper |
| 240 | Hyman SL, Levy SE, Myers SM. Identification, evaluation, and management of children with autism spectrum disorder. Pediatrics [Internet]. 2020;145(1):e20193447. Available from: <https://search.ebscohost.com/login.aspx?direct=true&db=psyh&AN=2020-00456-001&site=ehost-live> | Excluded | Review study |
| 241 | Paula CS, Cunha GR, Bordini D, Brunoni D, Moya AC, Bosa CA, et al. Identifying autism with a brief and low-cost screening instrument—OERA: Construct validity, invariance testing, and agreement between judges. J Autism Dev Disord. 2018;48(5):1780–91. | Excluded | Did not explore telehealth applications |
| 242 | Zhao Z, Zhu Z, Zhang X, Tang H, Xing J, Hu X, et al. Identifying autism with head movement features by implementing machine learning algorithms. J Autism Dev Disord. 2022;52(7):3038–49. | Excluded | Explored virtual reality, machine learning or robotic applications |
| 243 | Phelps RA, Sample E, Greene RK, Duvall SW. Identifying patient characteristics to understand which children may receive diagnostic clarity in a virtual autism spectrum disorder evaluation. J Autism Dev Disord. 2022;52(12):5126–38. | Included |  |
| 244 | Major S, Campbell K, Espinosa S, Baker JP, Carpenter KL, Sapiro G, et al. Impact of a digital Modified Checklist for Autism in Toddlers–Revised on likelihood and age of autism diagnosis and referral for developmental evaluation. Autism. 2020;24(7):1629–38. | Excluded | Did not examine relevant outcomes |
| 245 | Hathorn C, Alateeqi N, Graham C, O’Hare A. Impact of adherence to best practice guidelines on the diagnostic and assessment services for autism spectrum disorder. J Autism Dev Disord. 2014;44(8):1859–69. | Excluded | Did not explore telehealth applications |
| 246 | Isensee C, Schmid B, Marschik PB, Zhang D, Poustka L. Impact of COVID-19 pandemic on families living with autism: An online survey. Res Dev Disabil. 2022;129:104307. | Excluded | Did not explore telehealth applications |
| 247 | Allison K, Levac D. Impact of the COVID-19 pandemic on school-aged children with disabilities: Changes in therapy service delivery and functioning. Dev Med Child Neurol. 2021;63(Suppl 1):94–5. | Excluded | Examined/ elaborated on an intervention |
| 248 | Merrick H, Driver H, Main C, Kenny RPW, Richmond C, Allard A, et al. Impacts of healthcare service changes implemented due to COVID-19 on children and young people with long-term disability: A mapping review. Dev Med Child Neurol. 2023;65(7):885–99. | Excluded | Review study |
| 249 | Hart LC, Saha H, Lawrence S, Friedman S, Irwin P, Hanks C. Implementation and evolution of a primary care-based program for adolescents and young adults on the autism spectrum. J Autism Dev Disord. 2022;52(7):2924–33. | Excluded | Did not explore telehealth applications |
| 250 | Rutherford M, Forsyth K, McKenzie K, McClure I, Murray A, McCartney D, et al. Implementation of a practice development model to reduce the wait for autism spectrum diagnosis in adults. J Autism Dev Disord. 2018;48(5):1579–95. | Excluded | Did not explore telehealth applications |
| 251 | Valentine AZ, Hall SS, Young E, Brown BJ, Groom MJ, Hollis C, et al. Implementation of telehealth services to assess, monitor, and treat neurodevelopmental disorders: Systematic review. J Med Internet Res. 2021;23(1):e22619. | Excluded | Review study |
| 252 | Drahota A, Meza RD, Bustos TE, Aksheya S, Martinez JI, Brikho B, et al. Implementation-as-usual in community-based organizations providing specialized services to individuals with autism spectrum disorder: A mixed methods study. Adm Policy Ment Health. 2021;48(3):482–98. | Excluded | Did not explore telehealth applications |
| 253 | Nasir R, Czerniewska P, Pearlman S, Nagendran G, Jenkins C, Gurney R, et al. Implementing a telehealth autism diagnostic service in Barnet in response to COVID-19 restrictions. Arch Dis Child. 2021;106(Suppl 1):A278. | Excluded | Conference abstract |
| 254 | Hasan S, Spurrell M. Implementing a value-based complex case management workshop to support intellectual disability and autism care during the pandemic: A quality improvement project. Int J Integr Care. 2022;22(Suppl 1):1–2. | Excluded | Did not explore telehealth applications |
| 255 | Menon D, Singh V, Lipkin P. Improving access to specialty care for underserved children with neurodevelopmental disorders using telemedicine. Ann Neurol. 2016;80(Suppl 22):S387. | Excluded | Conference abstract |
| 256 | Lamy MO, Karlsson CD. Improving collaborative care for individuals with ASD, intellectual disability, and other neurodevelopmental disorders within a psychiatric medical home. J Am Acad Child Adolesc Psychiatry. 2022;61(10):S297. | Excluded | Conference abstract |
| 257 | Choueiri R, Lindenbaum A, Manasa R, Robsky W, Flahive J, Garrison W. Improving early identification and access to diagnosis of autism spectrum disorder in toddlers in a culturally diverse community with the Rapid Interactive Screening Test for Autism in Toddlers. J Autism Dev Disord. 2021;51(11):3937–45. | Excluded | Did not explore telehealth applications |
| 258 | Brinster MI, Brukilacchio BH, Fikki-Urbanovsky A, Shahidullah JD, Ravenscroft S. Improving efficiency and equity in early autism evaluations: The (S)TAAR model. J Autism Dev Disord. 2023;53(1):275–84. | Excluded | Did not explore telehealth applications |
| 259 | Rutherford M, Burns M, Gray D, Bremner L, Clegg S, Russell L, et al. Improving efficiency and quality of the children’s ASD diagnostic pathway: Lessons learned from practice. J Autism Dev Disord. 2018;48(5):1579–95. | Excluded | Did not explore telehealth applications |
| 260 | Verma S, Vandana P, Soda T, Koth KA, Aguayo P, Shaffer RC, et al. Incorporation of telepsychiatry for patients with developmental disorders into routine clinical practice: A survey of specialty clinics adapting to telepsychiatry during the COVID-19 pandemic. J Autism Dev Disord. 2022;52(12):5280–4. | Excluded | Examined/ elaborated on an intervention |
| 261 | Young E, Aiyadurai R, Jegathesan T, Brown C, Bechard N, Minhas RS, et al. Increasing access to developmental services for children with autism spectrum disorder: The Pediatric Developmental Passport Pilot randomized trial. J Autism Dev Disord. 2019;49(12):4867–76. | Excluded | Did not explore telehealth applications |
| 262 | Bennett A, Ray M, Zucker E, Chuo J. Increasing diagnostic services for autism spectrum disorder in the Native American community: A pilot collaborative telecare model. Pediatrics. 2021;147(3):970–1. | Excluded | Conference abstract |
| 263 | Shahidullah JD, Brinster M, Patel P, Cannady M, Krishnan A, Talebi H, et al. Increasing resources for autism evaluation and support for under-resourced schools through a state-wide school telehealth initiative. Psychol Sch. 2022;59(7):1295–307. | Excluded | No relevant data |
| 264 | Desideri L, Pérez-Fuster P, Herrera G. Information and communication technologies to support early screening of autism spectrum disorder: A systematic review. Child. 2021;8(2):93. | Excluded | Review study |
| 265 | Parsons S, Yuill N, Brosnan M, Good J. Innovative technologies for autism: Critical reflections on digital bubbles. J Assist Technol. 2015;9(2):116–23. | Excluded | Did not explore telehealth applications |
| 266 | De Luca R, Leonardi S, Portaro S, Le Cause M, De Domenico C, Colucci PV, et al. Innovative use of virtual reality in autism spectrum disorder: A case study. Appl Neuropsychol Child. 2021;10(1):90–100. | Excluded | Case study |
| 267 | Schrader E, Delehanty AD, Casler A, Petrie E, Rivera A, Harrison K, et al. Integrating a new online autism screening tool in primary care to lower the age of referral. Clin Pediatr (Phila). 2020;59(3):305–9. | Excluded | Did not explore telehealth applications |
| 268 | Colombo P, Buo N, Ceccarelli SB, Molteni M. Integrating a new online platform in primary care for early detection, referral, and intervention in autism spectrum disorder: The first Italian pivotal clinical study. Brain Sci. 2022;12(2):256. | Excluded | No relevant data |
| 269 | Jameson R, Lorence D, Lee J. Integrating computerized primitives and annotated video patterns: A proposed model for autism diagnosis and research. J Med Syst. 2012;36(3):2037–45. | Excluded | Theoretical paper |
| 270 | Egger HL, Lebwohl R. Interdisciplinary children’s digital health: Moving from ideas to population-level impact. J Am Acad Child Adolesc Psychiatry. 2018;57(10):S33. | Excluded | Theoretical paper |
| 271 | Pearl PL, Sable C, Evans S, Knight J, Cunningham P, Lotrecchiano GR, et al. International telemedicine consultations for neurodevelopmental disabilities. Telemed J E Health. 2014;20(6):559–62. | Excluded | Examined/ elaborated on an intervention |
| 272 | Yeh MJ, Shirley L, Balzer DT, Boe BA, El-Said H, Foerster S, et al. Interpreting quality improvement when introducing new technology: A collaborative experience in ASD device closures. Pediatr Cardiol. 2022;43(3):596–604. | Excluded | Non-ASD sample |
| 273 | Smith CJ, Rozga A, Matthews N, Oberleitner R, Nazneen N, Abowd G. Investigating the accuracy of a novel telehealth diagnostic approach for autism spectrum disorder. Psychol Assess. 2017;29(3):245–52. | Excluded | Did not examine relevant outcomes |
| 274 | Johnsson G, Kerslake R, Crook S, Cribb C. Investigation of training and support needs in rural and remote disability and mainstream service providers: Implications for an online training model. Aust Health Rev. 2017;41(6):693–7. | Excluded | Did not explore telehealth applications |
| 275 | Anonymous. Involving parents in the remote diagnosis of autism during the COVID-19 pandemic: A case study. J Psychopathol. 2022;28(1):27–9. | Excluded | Did not examine relevant outcomes |
| 276 | Reis D, Fricke O, Schulte AG, Schmidt P. Is examining children and adolescents with autism spectrum disorders a challenge? Measurement of Stress Appraisal (SAM) in German dentists with key expertise in paediatric dentistry. PLoS One. 2022;17(8):e0271406. | Excluded | Did not explore telehealth applications |
| 277 | Harris L, Gilmore D, Hanks C, Coury D, Moffatt-Bruce S, Garvin JH, et al. ‘It was surprisingly equivalent to the appointment I had in person’: Advantages and disadvantages of synchronous telehealth for delivering primary care for autistic adults. Autism. 2022;26(6):1573–80. | Excluded | Adult sample |
| 278 | St Amant HG, Schrager SM, Peña-Ricardo C, Williams ME, Vanderbilt DL. Language barriers impact access to services for children with autism spectrum disorders. J Autism Dev Disord. 2018;48(2):333–40. | Excluded | Did not explore telehealth applications |
| 279 | Parmanto B, Saptono A, Pramana G, Pulantara W, Schein RM, Schmeler MR, et al. VISYTER: Versatile and integrated system for telerehabilitation. Telemed J E Health. 2010;16(9):939–44. | Excluded | Examined/ elaborated on an intervention |
| 280 | Talbott MR, Dufek S, Young G, Rogers SJ. Leveraging telehealth to evaluate infants with prodromal autism spectrum disorder characteristics using the telehealth evaluation of development for infants. Autism. 2022;26(5):1242–54. | Included |  |
| 281 | Kingsdorf S, Pančocha K. Looking at Europe’s recent behavioral telehealth practices for children and families impacted by neurodevelopmental disabilities. Int J Dev Disabil. 2023;69(2):147–62. | Excluded | No relevant data |
| 282 | Raya MA, Marín-Morales J, Minissi ME, García GT, Abad L, Giglioli IAC. Machine learning and virtual reality on body movements’ behaviors to classify children with autism spectrum disorder. J Clin Med. 2020;9(5):1260. | Excluded | Explored virtual reality, machine learning or robotic applications |
| 283 | Koehler JC, Dong MS, Nelson AM, Fischer S, Spath J, Plank IS, et al. Machine learning classification of autism spectrum disorder based on reciprocity in naturalistic social interactions. medRxiv [Internet]. 2022; Available from: <https://www.medrxiv.org/> | Excluded | Explored virtual reality, machine learning or robotic applications |
| 284 | Dimitriou D, Esposito G. Management and support of individuals with developmental disabilities during the COVID-19 pandemic. Res Dev Disabil. 2022;125:104228. | Excluded | Commentary or editorial |
| 285 | Ahmed N, Raheem E, Rahman N, Khan MZR, Mosabbir AA, Hossain MS. Managing autism spectrum disorder in developing countries by utilizing existing resources: A perspective from Bangladesh. Autism. 2019;23(3):801–3. | Excluded | Did not explore telehealth applications |
| 286 | Huijnen CAGJ, Lexis MAS, de Witte LP. Matching robot KASPAR to autism spectrum disorder (ASD) therapy and educational goals. Int J Soc Robot. 2016;8(4):445–55. | Excluded | Explored virtual reality, machine learning or robotic applications |
| 287 | Stainbrook JA, Weitlauf AS, Juárez AP, Taylor JL, Hine J, Broderick N, et al. Measuring the service system impact of a novel telediagnostic service program for young children with autism spectrum disorder. Autism. 2019;23(4):1051–6. | Excluded | Did not examine relevant outcomes |
| 288 | Dreiling NG, Cook ML, Lamarche E, Klinger LG. Mental health Project ECHO autism: Increasing access to community mental health services for autistic individuals. Autism. 2022;26(2):434–45. | Excluded | Examined/ elaborated on an intervention |
| 289 | Pi HJ, Kallapiran K, Munivenkatappa S, Kandasamy P, Kirubakaran R, Russell P, et al. Meta-analysis of RCTs of technology-assisted parent-mediated interventions for children with ASD. J Autism Dev Disord. 2022;52(8):3325–43. | Excluded | Examined/ elaborated on an intervention |
| 290 | David N, Duckert S, Gewohn P, König H, Rahlff P, Erik F, et al. Mixed-methods investigation of barriers and needs in mental healthcare of adults with autism and recommendations for future care (BarrierfreeASD): Study protocol. BMJ Open. 2022;12(8):e061773. | Excluded | Adult sample |
| 291 | Sanders BW, Bedrick S, Broder-Fingert S, Brown SA, Dolata JK, Fombonne E, et al. Mobile and online consumer tools to screen for autism do not promote equity. Autism. 2023;27(3):714–22. | Excluded | Did not examine relevant outcomes |
| 292 | Bonnot O, Adrien V, Venelle V, Bonneau D, Gollier-Briant F, Mouchabac S. Mobile app for parental empowerment for caregivers of children with autism spectrum disorders: Prospective open trial. JMIR Ment Health [Internet]. 2021;8(9). Available from: <https://mental.jmir.org/2021/9/e27803/> | Excluded | Examined/ elaborated on an intervention |
| 293 | Tariq Q, Daniels J, Schwartz JN, Washington P, Kalantarian H, Wall DP. Mobile detection of autism through machine learning on home video: A development and prospective validation study. PLoS Med. 2018;15(11):e1002705. | Excluded | Explored virtual reality, machine learning or robotic applications |
| 294 | Conti E, Chericoni N, Costanzo V, Lasala R, Mancini A, Prosperi M, et al. Moving toward telehealth surveillance services for toddlers at risk for autism during the COVID-19 pandemic. Front Psychiatry. 2020;11:565999. | Excluded | Theoretical paper |
| 295 | Baghdadli A, Audras-Torrent L, Rattaz C, Gonnier V, Ferrando L, Michelon C, et al. Multistage screening process for neurodevelopmental disorders in siblings of children with autism: The FRATSA protocol study. BMJ Open. 2023;13(1):e066520. | Excluded | No relevant data |
| 296 | Williams NM, Hurt-Thaut C, Thaut MH. Novel screening tool and considerations for music therapists serving autistic individuals via telehealth: Qualitative results from a survey of clinicians’ experiences. J Music Ther. 2022;59(4):368–93. | Excluded | Examined/ elaborated on an intervention |
| 297 | MacKenzie KT, Mazefsky CA, Eack SM. Obtaining a first diagnosis of autism spectrum disorder: Descriptions of the diagnostic process and correlates of parent satisfaction from a national sample. J Autism Dev Disord. 2023;53(10):3799–812. | Excluded | Did not explore telehealth applications |
| 298 | Eggleston MJF, Thabrew H, Frampton CMA, Eggleston KHF, Hennig SC. Obtaining an autism spectrum disorder diagnosis and supports: New Zealand parents’ experiences. Res Autism Spectr Disord. 2019;62:18–25. | Excluded | Did not explore telehealth applications |
| 299 | Eigsti IM, Thomas RP, Stabile M, Mohan A, Dieckhaus MFS, Crutcher J, et al. Online administration of the ADOS for research with adolescents and adults in response to the pandemic. Autism Res. 2022;15(10):1909–16. | Excluded | Adult sample |
| 300 | Oberleitner R, Elison-Bowers P, Reischl U, Ball J. Optimizing the personal health record with special video capture for the treatment of autism. J Dev Phys Disabil. 2007;19(5):513–8. | Excluded | Examined/ elaborated on an intervention |
| 301 | Tsami L, Nguyen JT, Alphonso N, Lerman D, Matteucci M, Chen N. Outcomes and acceptability of telehealth-based coaching for caregivers in Asian countries. Behav Modif. 2023;47(2):297–323. | Excluded | Examined/ elaborated on an intervention |
| 302 | Bejarano-Martín Á, Canal-Bedia R, Magán-Maganto M, Fernández-Álvarez C, Martín-Cilleros MV, Sánchez-Gómez MC, et al. Early detection, diagnosis, and intervention services for children with autism spectrum disorder across Europe according to the gross domestic product. Eur Neuropsychopharmacol. 2021;53:S249–50. | Excluded | Did not explore telehealth applications |
| 303 | Hall CM. Parent consultation and transitional care for military families of children with autism: A teleconsultation implementation project. J Educ Psychol Consult. 2022;32(2):185–203. | Excluded | Examined/ elaborated on an intervention |
| 304 | Gauert S, Rittenhouse-Cea H, Rittenhouse-Shaw K. Parent implementation of DTT following telehealth instruction. J Autism Dev Disord. 2023;53(10):3980–6. | Excluded | Examined/ elaborated on an intervention |
| 305 | Garnett R, Davidson B, Eadie P. Parent perceptions of a group telepractice communication intervention for autism. Autism Dev Lang Impair [Internet]. 2022;7. Available from: <https://search.ebscohost.com/login.aspx?direct=true&db=psyh&AN=2022-22059-001&site=ehost-live> | Excluded | Examined/ elaborated on an intervention |
| 306 | Corona LL, Weitlauf AS, Hine J, Berman A, Miceli A, Nicholson A, et al. Parent perceptions of caregiver-mediated telemedicine tools for assessing autism risk in toddlers. J Autism Dev Disord. 2021;51(2):476–86. | Included |  |
| 307 | Salomone E, Maurizio Arduino G. Parental attitudes to a telehealth parent coaching intervention for autism spectrum disorder. J Telemed Telecare. 2017;23(3):416–20. | Excluded | Examined/ elaborated on an intervention |
| 308 | Eapen V, Winata T, Gilbert M, Nair R, Khan F, Lucien A, et al. Parental experience of an early developmental surveillance programme for autism within Australian general practice: A qualitative study. BMJ Open. 2022;12(11):e064375. | Excluded | Did not examine relevant outcomes |
| 309 | Snijder MIJ, Langerak IPC, Kaijadoe SPT, Buruma ME, Verschuur R, Dietz C, et al. Parental experiences with early identification and initial care for their child with autism: Tailored improvement strategies. J Autism Dev Disord. 2022;52(8):3473–85. | Excluded | Did not explore telehealth applications |
| 310 | Dasal Tenzin Jashar, Fein D, Berry LN, Burke JD, Miller LE, Barton ML, et al. Parental perceptions of a comprehensive diagnostic evaluation for toddlers at risk for autism spectrum disorder. J Autism Dev Disord. 2019;49(5):1763–77. | Excluded | Did not explore telehealth applications |
| 311 | Meral BF. Parental views of families of children with autism spectrum disorder and developmental disorders during the COVID-19 pandemic. J Autism Dev Disord. 2022;52(4):1712–24. | Excluded | Did not explore telehealth applications |
| 312 | Bent CA, Barbaro J, Dissanayake C. Parents’ experiences of the service pathway to an autism diagnosis for their child: What predicts an early diagnosis in Australia? Res Dev Disabil. 2020;103:103689. | Excluded | Did not explore telehealth applications |
| 313 | Miller S, Juricic M, Coates J, O’Donnell M, Mulpuri K. Parents’ perspective of receiving a diagnosis for their child. Dev Med Child Neurol. 2021;63:27–8. | Excluded | Did not explore telehealth applications |
| 314 | Molteni P, Maggiolini S. Parents’ perspectives towards the diagnosis of autism: An Italian case study research. J Child Fam Stud. 2015;24(4):1088. | Excluded | Did not explore telehealth applications |
| 315 | Jacobs D, Steyaert J, Dierickx K, Hens K. Parents’ views and experiences of the autism spectrum disorder diagnosis of their young child: A longitudinal interview study. Eur Child Adolesc Psychiatry. 2020;29(8):1143–54. | Excluded | Did not explore telehealth applications |
| 316 | Maun R, Fabri M, Trevorrow P. Participatory methods to engage autistic people in the design of digital technology: A systematic literature review. J Autism Dev Disord [Internet]. 2023; Available from: <https://www.springer.com/journal/10803> | Excluded | Review study |
| 317 | Kennelly AM, McIntyre B, Wood AC, Monteiro S, Voigt RG. Patient satisfaction in neurodevelopmental pediatrics: In-person vs telemedicine. J Child Neurol. 2022;37(3):181–5. | Included |  |
| 318 | Legault O, Neufeld R. Patterns of referrals for evaluation of autism spectrum disorder in the pediatric population of Saskatchewan as a predictor of diagnostic outcome. Paediatr Child Health. 2022;27:e22. | Excluded | Did not explore telehealth applications |
| 319 | Crais ER, McComish CS, Humphreys BP, Watson LR, Baranek GT, Reznick JS, et al. Pediatric healthcare professionals’ views on autism spectrum disorder screening at 12–18 months. J Autism Dev Disord. 2014;44(9):2311. | Excluded | Did not explore telehealth applications |
| 320 | Hornby B, Tolen L, Jennejahn W, Johnson D, Joseph C, Laux J, et al. Pediatric therapists’ telehealth experiences. Dev Med Child Neurol. 2022;64:116. | Excluded | Examined/ elaborated on an intervention |
| 321 | Magro K. Perception of resources for parents of children with autism who are in online communities [Internet]. Vol. 84, Dissertation Abstracts International Section A: Humanities and Social Sciences. ProQuest Information & Learning; 2023. Available from: <https://search.ebscohost.com/login.aspx?direct=true&db=psyh&AN=2023-68882-170&site=ehost-live> | Excluded | Did not explore telehealth applications |
| 322 | Manning J, Billian J, Matson J, Allen C, Neelkamal S. Perceptions of families of individuals with autism spectrum disorder during the COVID-19 crisis. J Autism Dev Disord. 2021;51(8):2920–8. | Excluded | Did not explore telehealth applications |
| 323 | Swan MZ. Perceptions of parent-implemented autism intervention online [Internet]. Vol. 84, Dissertation Abstracts International: Section B: The Sciences and Engineering. ProQuest Information & Learning; 2023. Available from: <https://search.ebscohost.com/login.aspx?direct=true&db=psyh&AN=2023-76638-105&site=ehost-live> | Excluded | Examined/ elaborated on an intervention |
| 324 | Gholipour K, Ghiasi A, Shahrokhi H, Dadashi Z, Javanmard S, Tabatabaei SH, et al. Perceptions of the professionals and parents of children with autism spectrum disorders about autism services: A qualitative study. J Autism Dev Disord. 2023;53(1):96–109. | Excluded | Did not explore telehealth applications |
| 325 | Narzisi A. Phase 2 and later of COVID-19 lockdown: Is it possible to perform remote diagnosis and intervention for autism spectrum disorder? An online-mediated approach. J Clin Med. 2020;9(6):1–12. | Excluded | Theoretical paper |
| 326 | Millar L, McConnachie A, Minnis H, Wilson P, Thompson L, Anzulewicz A, et al. Phase 3 diagnostic evaluation of a smart tablet serious game to identify autism in 760 children aged 3–5 years in Sweden and the United Kingdom. BMJ Open. 2019;9(7):e026226. | Excluded | Explored virtual reality, machine learning or robotic applications |
| 327 | Williams ME, Harley EK, Quebles I, Poulsen MK. Policy and practice barriers to early identification of autism spectrum disorder in the California Early Intervention System. J Autism Dev Disord. 2021;51(10):3423–31. | Excluded | Did not explore telehealth applications |
| 328 | Cumin J, Pelaez S, Mottron L. Positive and differential diagnosis of autism in verbal women of typical intelligence: A Delphi study. Autism. 2022;26(5):1153–64. | Excluded | Did not explore telehealth applications |
| 329 | Pistoljevic N, Eldin D, Ghaziuddin M. Practice patterns and potential barriers to early diagnosis of autism in Bosnia and Herzegovina: A preliminary study. J Autism Dev Disord. 2021;51(12):4447–55. | Excluded | Did not explore telehealth applications |
| 330 | Banaschewski T, Bruni O, Fuentes J, Hill CM, Hvolby A, Posserud MB, et al. Practice tools for screening and monitoring insomnia in children and adolescents with autism spectrum disorder. J Autism Dev Disord. 2022;52(8):3758–68. | Excluded | Did not examine relevant outcomes |
| 331 | Johnsson G, Bulkeley K. Practitioner and service user perspectives on the rapid shift to teletherapy for individuals on the autism spectrum as a result of COVID-19. Int J Environ Res Public Health. 2021;18(22):11800. | Excluded | Examined/ elaborated on an intervention |
| 332 | Franz L, Howard J, Viljoen M, Sikich L, Chandrasekhar T, Kollins SH, et al. Pragmatic adaptations of telehealth-delivered caregiver coaching for children with autism in the context of COVID-19: Perspectives from the United States and South Africa. Autism. 2022;26(1):270–5. | Excluded | Examined/ elaborated on an intervention |
| 333 | Anderberg E, South M. Predicting parent reactions at diagnostic disclosure sessions for autism. J Autism Dev Disord. 2021;51(10):3533–46. | Excluded | Did not explore telehealth applications |
| 334 | Kaba D, Hasanlı J, Efe A, Yavuz-Çolak M, Akın-Sarı B. Predictors of burnout and distress in parents of children with autism spectrum disorder during COVID-19 home confinement. Child Health Care. 2023;52(4):409–29. | Excluded | Did not explore telehealth applications |
| 335 | Brei NG, Schwarz GN, Klein-Tasman BP. Predictors of parenting stress in children referred for an autism spectrum disorder diagnostic evaluation. J Dev Phys Disabil. 2015;27(5):617–35. | Excluded | Did not explore telehealth applications |
| 336 | Ferguson EF, Jimenez-Muñoz M, Feerst H, Vernon TW. Predictors of satisfaction with autism treatment services during COVID-19. J Autism Dev Disord. 2022;52(8):3686–97. | Excluded | Examined/ elaborated on an intervention |
| 337 | Reese RM, Braun MJ, Hoffmeier S, Stickle L, Rinner L, Smith C, et al. Preliminary evidence for the integrated systems using telemedicine. Telemed J E Health. 2015;21(7):581–7. | Included |  |
| 338 | Vismara LA, McCormick C, Young GS, Nadhan A, Monlux K. Preliminary findings of a telehealth approach to parent training in autism. J Autism Dev Disord. 2013;43(12):2953–69. | Excluded | Examined/ elaborated on an intervention |
| 339 | Kryszak EM, Albright CM, Stephenson KG, Nevill RE, Hedley D, Burns CO, et al. Preliminary validation and feasibility of the Autism Detection in Early Childhood-Virtual (ADEC-V) for autism telehealth evaluations in a hospital setting. J Autism Dev Disord. 2022;52(12):5139–49. | Excluded | Did not examine relevant outcomes |
| 340 | Neely L, Rispoli M, Gerow S, Hong ER. Preparing interventionists via telepractice in incidental teaching for children with autism. J Behav Educ. 2016;25(4):393–416. | Excluded | Did not explore telehealth applications |
| 341 | Hatfield M, Falkmer M, Falkmer T, Ciccarelli M. Process evaluation of the BOOST-ATM transition planning program for adolescents on the autism spectrum: A strengths-based approach. J Autism Dev Disord. 2018;48(2):377–88. | Excluded | Examined/ elaborated on an intervention |
| 342 | Kellom KS, Flaherty CM, Cacia J, Christiansen A, Cordero L, Hah J, et al. Provider and caregiver perspectives on telehealth assessments for autism spectrum disorder in young children: A multimethod DBPNet study exploring equity. J Dev Behav Pediatr. 2023;44(6):e397–411. | Included |  |
| 343 | Reisinger DL, Hines E, Raches C, Tang Q, James C, Keehn RM. Provider and caregiver satisfaction with telehealth evaluation of autism spectrum disorder in young children during the COVID-19 pandemic. J Autism Dev Disord. 2022;52(12):5099–113. | Included |  |
| 344 | Morales YM, Sanders B, Vazquez LR, Fuchu P, Fernandez J, Dolata J, et al. Provider perspective on benefits and challenges of mHealth autism screeners in underserved families. Pediatrics [Internet]. 2022;149. Available from: <https://publications.aap.org/pediatrics/article/149/1MeetingAbstractsFebruary2022/88/185761> | Excluded | Conference abstract |
| 345 | Fell LA, Albright CM, Kryszak EM, Butter E, Kuhlthau KA. Provider perspectives on telehealth services for children with autism spectrum disorder during the Coronavirus Disease 2019 pandemic. Acad Pediatr. 2023;23(6):1196–203. | Excluded | Examined/ elaborated on an intervention |
| 346 | Scattoni ML, Micai M, Ciaramella A, Salvitti T, Fulceri F, Fatta LM, et al. Real-world experiences in autistic adult diagnostic services and post-diagnostic support and alignment with services guidelines: Results from the ASDEU study. J Autism Dev Disord. 2021;51(11):4129–46. | Excluded | Adult sample |
| 347 | Christopoulou M, Drosos K, Petinou K. Recent advances of telepractice for autism spectrum disorders in speech and language pathology. Neuropsychiatr Dis Treat. 2022;18:2379–89. | Excluded | Theoretical paper |
| 348 | Onishchenko D, Huang Y, van Horne J, Smith PJ, Msall ME, Chattopadhyay I. Reduced false positives in autism screening via digital biomarkers inferred from deep comorbidity patterns. Sci Adv. 2021;7(41):eabf0354. | Excluded | Did not explore telehealth applications |
| 349 | Rollins PR, De Froy AM. Reexamining pathways early autism intervention in children before and after the third birthday: A randomized control trial. J Autism Dev Disord. 2023;53(3):1189–201. | Excluded | Did not explore telehealth applications |
| 350 | Bernie C, Williams K, O’Connor B, Rogers S, May T. Referral, assessment and use of screening measures related to autism spectrum disorder at a tertiary hospital setting. J Autism Dev Disord. 2021;51(8):2673–85. | Excluded | Did not explore telehealth applications |
| 351 | Coffman M, Di Martino JM, Aiello R, Carpenter KLH, Chang Z, Compton S, et al. Relationship between quantitative digital behavioral features and clinical profiles in young autistic children. Autism Res. 2023;16(7):1360–74. | Excluded | Examined a mobile application |
| 352 | Chen Y, Wang J, Guo Y, Zhu Z, Bai X, Li X. Reliability and validity of a novel caregiver-assessed skills system based on the ALSO conception in children with autism spectrum disorders. Transl Pediatr. 2021;10(2):366–77. | Excluded | Did not explore telehealth applications |
| 353 | Mohan HS, Tiwari S, Guddattu V. Reliability of oral language and emergent literacy tele-assessments in autistic children: A preliminary study. Technol Disabil. 2023;35(3):195–206. | Excluded | Did not examine relevant outcomes |
| 354 | Adamou M, Jones SL, Fullen T, Galab N, Abbott K, Yasmeen S. Remote assessment in adults with autism or ADHD: A service user satisfaction survey. PLoS One. 2021;16(3):e0249237. | Excluded | Adult sample |
| 355 | Keller MA, Tharpe AM, Bodfish J. Remote microphone system use in preschool children with autism spectrum disorder and language disorder in the classroom: A pilot efficacy study. Am J Speech Lang Pathol. 2021;30(1):266–78. | Excluded | Examined/ elaborated on an intervention |
| 356 | Patane S, Cigala V, Patti A, Mandalari P, Lo Giudice M, Basile K, et al. Reorganization of ‘the intensive and early diagnosis and treatment service’ for children with ASD: The Messina NPIA UOC ASP5 experience. Dev Med Child Neurol. 2021;63:48. | Excluded | Conference abstract |
| 357 | Lenart A, Pasternak J. Resources, problems and challenges of autism spectrum disorder diagnosis and support system in Poland. J Autism Dev Disord. 2023;53(4):1629–41. | Excluded | Did not explore telehealth applications |
| 358 | O’Brolchain F, Gordijn B. Responsibility-enhancing assistive technologies and people with autism. Camb Q Healthc Ethics. 2020;29(4):607–16. | Excluded | Did not explore telehealth applications |
| 359 | Zwaigenbaum L, Bishop S, Stone WL, Ibanez L, Halladay A, Goldman S, et al. Rethinking autism spectrum disorder assessment for children during COVID‐19 and beyond. Autism Res. 2021;14(11):2251–9. | Excluded | Commentary or editorial |
| 360 | Ben-Sasson A, Robins DL, Yom-Tov E. Risk assessment for parents who suspect their child has autism spectrum disorder: Machine learning approach. J Med Internet Res. 2018;20(4):e134. | Excluded | Explored virtual reality, machine learning or robotic applications |
| 361 | Saleh MA, Hanapiah FA, Hashim H. Robot applications for autism: A comprehensive review. Disabil Rehabil Assist Technol. 2021;16(6):580–602. | Excluded | Explored virtual reality, machine learning or robotic applications |
| 362 | Antezana L, Scarpa A, Valdespino A, Albright J, Richey JA. Rural trends in diagnosis and services for autism spectrum disorder. Front Psychol [Internet]. 2017;8. Available from: <https://search.ebscohost.com/login.aspx?direct=true&db=psyh&AN=2017-18143-001&site=ehost-live> | Excluded | Did not explore telehealth applications |
| 363 | Moody EJ, Reyes N, Ledbetter C, Wiggins L, Diguiseppi C, Alexander A, et al. Screening for autism with the SRS and SCQ: Variations across demographic, developmental and behavioral factors in preschool children. J Autism Dev Disord. 2017;47(11):3550. | Excluded | Did not explore telehealth applications |
| 364 | Kanne SM, Carpenter LA, Warren Z. Screening in toddlers and preschoolers at risk for autism spectrum disorder: Evaluating a novel mobile-health screening tool. Autism Res. 2018;11(7):1038–49. | Excluded | No relevant data |
| 365 | Gonzalez-Herrero B, Morgante F, Pagonabarraga J, Stanton B, Edwards M. Screening of autistic traits in people with functional neurological disorders. Mov Disord. 2022;37:S285. | Excluded | Did not explore telehealth applications |
| 366 | Sanders B, Fombonne E, Zuckerman K, Bedrick S, Broder-Fingert S, Dolata J, et al. Searches for web tools to screen for autism produce few results. Pediatrics. 2021;147(3):21. | Excluded | Conference abstract |
| 367 | Washington P, Leblanc E, Dunlap K, Penev Y, Varma M, Jung J-Y, et al. Selection of trustworthy crowd workers for telemedical diagnosis of pediatric autism spectrum disorder. Pac Symp Biocomput. 2021;26:14–25. | Excluded | Explored virtual reality, machine learning or robotic applications |
| 368 | Ingersoll B, Shannon K, Berger N, Pickard K, Holtz B. Self-directed telehealth parent-mediated intervention for children with autism spectrum disorder: Examination of the potential reach and utilization in community settings. J Med Internet Res. 2017;19(7):410–20. | Excluded | Examined/ elaborated on an intervention |
| 369 | Mello MP, Urbano RC, Goldman SE, Hodapp RM. Services for children with autism spectrum disorder: Comparing rural and non-rural communities. Educ Train Autism Dev Disabil. 2016;51(4):355. | Excluded | Did not explore telehealth applications |
| 370 | Su W-C, Srinivasan S, Cleffi C, Bhat A. Short report on research trends during the COVID-19 pandemic and use of telehealth interventions and remote brain research in children with autism spectrum disorder. Autism. 2021;25(6):1816–22. | Excluded | Review study |
| 371 | Talbott MR, Lang E, Avila F, Dufek S, Young G. Short report: Experiences of caregivers participating in a telehealth evaluation of development for infants (TEDI). J Autism Dev Disord. 2022;52(12):5266–73. | Included |  |
| 372 | Castro-Kemp S. Silver linings of the COVID-19 pandemic… for some! Comparing experiences and sociodemographic characteristics of autistic and non-autistic children with SEND in England. J Autism Dev Disord. 2023;53(10):3968–79. | Excluded | Did not explore telehealth applications |
| 373 | Al Mamun KA, Bardhan S, Ullah MA, Anagnostou E, Brian J, Akhter S, et al. Smart autism: A mobile, interactive and integrated framework for screening and confirmation of autism. Annu Int Conf IEEE Eng Med Biol Soc. 2016;2016:5989–92. | Excluded | Explored virtual reality, machine learning or robotic applications |
| 374 | Sievers SB, Trembath D, Westerveld MF. Speech-language pathologists’ knowledge and consideration of factors that may predict, moderate, and mediate AAC outcomes. J Autism Dev Disord. 2020;50(1):238–49. | Excluded | Did not explore telehealth applications |
| 375 | Stone WL, Ibanez L, Newschaffer C, Rohloff E, Abdullah M, Burkom D, et al. Streamlining the diagnosis of autism spectrum disorder for the National Children’s Study. Birth Defects Res A Clin Mol Teratol. 2014;100(5):367. | Excluded | Did not explore telehealth applications |
| 376 | Davis IN. Supporting early childhood educators’ completion of the Autism Navigator About Autism in Toddlers course [Internet]. Vol. 81, Dissertation Abstracts International Section A: Humanities and Social Sciences. ProQuest Information & Learning; 2020. Available from: <https://search.ebscohost.com/login.aspx?direct=true&db=psyh&AN=2020-28115-075&site=ehost-live> | Excluded | Examined/ elaborated on an intervention |
| 377 | Goh TJ, Lim T, Foo M, Ong SKA, Aishworiya R, Nair T, et al. Supporting individuals with autism spectrum disorder in medical settings during COVID-19. Asian J Psychiatr. 2020;54. | Excluded | Theoretical paper |
| 378 | Wahlstrom D. Technology and computerized assessments: Current state and future directions. In: APA Handbook of Forensic Neuropsychology [Internet]. Washington, DC: American Psychological Association; 2017. p. 463–76. Available from: <https://search.ebscohost.com/login.aspx?direct=true&db=psyh&AN=2017-29067-021&site=ehost-live> | Excluded | Book chapter |
| 379 | Soysa AI, Al Mahmud A. Technology for children with autism spectrum disorder: What do Sri Lankan parents and practitioners want? Interact Comput. 2019;31(3):282–302. | Excluded | Did not examine relevant outcomes |
| 380 | Jordan KA. Technology, autism, and occupational therapy. In: Technology and the Treatment of Children with Autism Spectrum Disorder [Internet]. Cham: Springer International Publishing; 2016. p. 37–47. Available from: <https://search.ebscohost.com/login.aspx?direct=true&db=psyh&AN=2015-42152-004&site=ehost-live> | Excluded | Book chapter |
| 381 | McNally Keehn R, Enneking B, Ryan T, James C, Tang Q, Blewitt A, et al. Tele-assessment of young children referred for autism spectrum disorder evaluation during COVID-19: Associations among clinical characteristics and diagnostic outcome. Autism. 2023;27(5):1362–76. | Included |  |
| 382 | Law J, Dornstauder M, Charlton J, Greaux M. Tele-practice for children and young people with communication disabilities: Employing the COM-B model to review the intervention literature and inform guidance for practitioners. Int J Lang Commun Disord. 2021;56(2):415–34. | Excluded | Theoretical paper |
| 383 | Bertelli MO, Zorzi S, Buonaguro EF, Bianco A, Armellini M, Scattoni ML. Teleassistance and telerehabilitation: COVID-19, other epidemic situations and future prospects. In: Textbook of Psychiatry for Intellectual Disability and Autism Spectrum Disorder [Internet]. Cham: Springer Nature Switzerland AG; 2022. p. 1031–50. Available from: <https://search.ebscohost.com/login.aspx?direct=true&db=psyh&AN=2023-11548-043&site=ehost-live> | Excluded | Book chapter |
| 384 | Myers K, Chronis-Tuscano AM. Telebehavioral health interventions: Diverse populations and settings. J Am Acad Child Adolesc Psychiatry. 2018;57(10):S8–9. | Excluded | Examined/ elaborated on an intervention |
| 385 | Pedernera Bradichansky PP, Selvatici L, Napoli S, Lejarraga C, Mato A, Urinovsky MG, et al. Teleconsultation during a pandemic: Experience of developmental pediatricians at Hospital de Pediatria ‘Prof. Dr. Juan P. Garrahan’. Arch Argent Pediatr. 2021;119(6):419–23. | Included |  |
| 386 | Ellison KS, Guidry J, Picou P, Adenuga P, Davis TE III. Telehealth and autism prior to and in the age of COVID-19: A systematic and critical review of the last decade. Clin Child Fam Psychol Rev. 2021;24(3):599–630. | Excluded | Review study |
| 387 | Sutherland R, Trembath D, Roberts J. Telehealth and autism: A systematic search and review of the literature. Int J Speech Lang Pathol. 2018;20(3):324–36. | Excluded | Review study |
| 388 | Sutherland R, Trembath D, Hodge MA, Rose V, Roberts J. Telehealth and autism: Are telehealth language assessments reliable and feasible for children with autism? Int J Lang Commun Disord. 2019;54(2):281–91. | Excluded | Not assessing for ASD |
| 389 | Lindgren S, Wacker D, Suess A, Schieltz K, Pelzel K, Kopelman T, et al. Telehealth and autism: Treating challenging behavior at lower cost. Pediatrics. 2016;137:S167–75. | Excluded | Examined/ elaborated on an intervention |
| 390 | Solomon D, Soares N. Telehealth approaches to care coordination in autism spectrum disorder. In: Interprofessional Care Coordination for Pediatric Autism Spectrum Disorder: Translating Research Into Practice [Internet]. Cham: Springer Nature Switzerland AG; 2020. p. 289–306. Available from: <https://search.ebscohost.com/login.aspx?direct=true&db=psyh&AN=2022-06567-019&site=ehost-live> | Excluded | Book chapter |
| 391 | Spain D, Stewart GR, Mason D, Milner V, Fairhurst B, Robinson J, et al. Telehealth autism diagnostic assessments with children, young people, and adults: Qualitative interview study with England-wide multidisciplinary health professionals. JMIR Ment Health. 2022;9(7):1–18. | Included |  |
| 392 | Butsch M, Aites M, Cartisano T, Roy D, Jordan B, Flake E, et al. Telehealth diagnoses of autism spectrum disorder in toddlers. J Investig Med. 2020;68(1):A162. | Excluded | Conference abstract |
| 393 | Ludwig NN, Child AE, Jashar DT, Mostow AJ, Wodka EL. Telehealth diagnosis of autism spectrum disorder through clinical cases. Clin Neuropsychol. 2022;36(5):960–80. | Excluded | Theoretical paper |
| 394 | McNally Keehn R, Enneking B, James C, Tang Q, Rouse M, Hines E, et al. Telehealth evaluation of pediatric neurodevelopmental disabilities during the COVID-19 pandemic: Clinician and caregiver perspectives. J Dev Behav Pediatr. 2022;43(5):262–72. | Included |  |
| 395 | Gopalkrishnan IK, Venkatesan S. Telehealth for children with autistic spectrum disorder: Indian need versus challenges. In: Enabling Technology for Neurodevelopmental Disorders: From Diagnosis to Rehabilitation [Internet]. New York, NY: Routledge; 2022. p. 58–78. Available from: <https://search.ebscohost.com/login.aspx?direct=true&db=psyh&AN=2023-36284-005&site=ehost-live> | Excluded | No relevant data |
| 396 | Cameron MJ, Moore T, Bogran C, Leidt A. Telehealth for family guidance: Acceptance and commitment therapy, parent-focused preference assessment, and activity-based instruction for the support of children with autism spectrum disorder and their families. Behav Anal Pract. 2021;14(4):1128–34. | Excluded | Examined/ elaborated on an intervention |
| 397 | Leyser M, Schieltz K, Strathearn L, Cooper-Brown L, McBrien D, O’Brien M. Telehealth in the field of developmental-behavioral pediatrics: Advantages, challenges, and future perspectives. J Dev Behav Pediatr. 2021;42(3):240–4. | Excluded | Theoretical paper |
| 398 | Sivaraman M, Virues-Ortega J, Roeyers H. Telehealth mask-wearing training for children with autism during the COVID-19 pandemic. J Appl Behav Anal. 2021;54(1):70–86. | Excluded | Did not examine relevant outcomes |
| 399 | Nelson EL, Zhang E, Bellinger S, Cain S, Davis A, Lassen S, et al. Telehealth ROCKS at home: Pandemic transition of rural school-based to home-based telebehavioral health services. Rural Ment Health. 2023;47(2):114–22. | Excluded | Theoretical paper |
| 400 | Doyen C, Goupil V, Desailly E, Oreve MJ, Kaye K. Telemedicine and autism spectrum disorder in children and adolescents: Theoretical and practical guide. Ann Med Psychol. 2019;177(7):702–9. | Excluded | Theoretical paper |
| 401 | Langkamp DL, McManus MD, Blakemore SD. Telemedicine for children with developmental disabilities: A more effective clinical process than office-based care. Telemed J E Health. 2015;21(2):110–4. | Excluded | Did not examine relevant outcomes |
| 402 | Stuckey R, Domingues-Montanari S. Telemedicine is helping the parents of children with neurodevelopmental disorders living in remote and deprived areas. Paedia. 2017;37(3):155–7. | Excluded | Commentary or editorial |
| 403 | Goldstein F, Myers K. Telemental health: A new collaboration for pediatricians and child psychiatrists. Pediatr Ann. 2014;43(2):79–84. | Excluded | Theoretical paper |
| 404 | Silva AP da, Caceres-Assenco AM. Telemonitoring of children with risk indicators for autism spectrum disorder: Preliminary findings. CoDAS. 2023;35(5):e20210308. | Excluded | No relevant data |
| 405 | Garnett R, Davidson B, Eadie P, Clarke K, Aggarwal D. Telepractice delivery of an autism communication intervention to parent groups: A feasibility study. J Clin Pract Speech Lang Pathol. 2019;21(2):70–7. | Excluded | Examined/ elaborated on an intervention |
| 406 | Boisvert M, Lang R, Andrianopoulos M, Boscardin ML. Telepractice in the assessment and treatment of individuals with autism spectrum disorders: A systematic review. Dev Neurorehabil. 2010;13(6):423–32. | Excluded | Review study |
| 407 | Clarke CS. Telepsychiatry in Asperger’s syndrome. Ir J Psychol Med. 2018;35(4):325–8. | Excluded | Case study |
| 408 | Lee G. The application of telehealth procedures to provide behavioral assessment and treatment to families with young children with autism spectrum disorder in Korea [Internet]. Vol. 79, Dissertation Abstracts International: Section B: The Sciences and Engineering. ProQuest Information & Learning; 2018. Available from: <https://search.ebscohost.com/login.aspx?direct=true&db=psyh&AN=2018-40527-003&site=ehost-live> | Excluded | Examined/ elaborated on an intervention |
| 409 | Olcay S, Karabulut E, Saral D. Zoom in screen with simultaneous prompting to teach young children with autism. Int J Dev Disabil. 2023;70(8):1478-1489. Published 2023 Mar 22. | Excluded | Examined/ elaborated on an intervention |
| 410 | Chen V, Gellasch P, Glascoe F. The Delaware Early Childhood Screening Initiative: Raising the bar. Pediatrics. 2020;146(1):42. | Excluded | Did not explore telehealth applications |
| 411 | Nicolaidis C, Raymaker D, McDonald K, Kapp S, Weiner M, Ashkenazy E, et al. The development and evaluation of an online healthcare toolkit for autistic adults and their primary care providers. J Gen Intern Med. 2016;31(10):1180–9. | Excluded | Adult sample |
| 412 | Lin CS, Chang SH, Liou WY, Tsai YS. The development of a multimedia online language assessment tool for young children with autism. Res Dev Disabil. 2013;34(10):3553–65. | Excluded | Did not examine relevant outcomes |
| 413 | Kumm AJ, Viljoen M, de Vries PJ. The digital divide in technologies for autism: Feasibility considerations for low- and middle-income countries. J Autism Dev Disord. 2022;52(5):2300–13. | Excluded | Theoretical paper |
| 414 | Antill KL. The effects of coaching provided via video-conferencing to caregivers of children with ASD [Internet]. Vol. 84, Dissertation Abstracts International Section A: Humanities and Social Sciences. ProQuest Information & Learning; 2023. Available from: <https://search.ebscohost.com/login.aspx?direct=true&db=psyh&AN=2023-46640-084&site=ehost-live> | Excluded | Examined/ elaborated on an intervention |
| 415 | Allen AA, Shane HC. The evaluation of children with an autism spectrum disorder: Adaptations to accommodate a telepractice model of clinical care. Perspect Telepract. 2014;4(2):42–51. | Excluded | Did not examine relevant outcomes |
| 416 | Karrim SB, Flack PS, Naidoo U, Beagle S, Pontin A. The experiences of speech-language therapists providing telerehabilitation services to children with autism spectrum disorder. S Afr J Commun Disord. 2022;69(2):1–12. | Excluded | Examined/ elaborated on an intervention |
| 417 | Brunt S, Sadikova E, Pappagianopoulos J, Mazurek MO. The impact of COVID-19 on receipt of health services among children with and without autism. Autism. 2023;27(3):714–29. | Excluded | Theoretical paper |
| 418 | Gillespie-Smith K, McConachie D, Ballantyne C, Auyeung B, Goodall K. The impact of COVID-19 restrictions on psychological distress in family caregivers of children with neurodevelopmental disability in the UK. J Autism Dev Disord. 2023;53(4):1573–87. | Excluded | Did not explore telehealth applications |
| 419 | Coutelle R, Boedec M, Vermeulen K, Kummeling J, Koolen DA, Kleefstra T, et al. The impact of lockdown on young people with genetic neurodevelopmental disabilities: A study with the international participatory database GenIDA. BMC Psychiatry. 2022;22. | Excluded | Did not explore telehealth applications |
| 420 | Madayi A, Yeleswarapu SP, Wong CM. The impact of Singapore’s COVID-19 circuit breaker measures on children with developmental delays and their families. Arch Dis Child. 2021;106:A211–2. | Excluded | Did not explore telehealth applications |
| 421 | Sharma A, Marwaha H. The impact of technology on children with autism spectrum disorder. In: Enabling Technology for Neurodevelopmental Disorders: From Diagnosis to Rehabilitation [Internet]. New York, NY: Routledge; 2022. p. 46–57. Available from: <https://search.ebscohost.com/login.aspx?direct=true&db=psyh&AN=2023-36284-004&site=ehost-live> | Excluded | Book chapter |
| 422 | Mete Yesil A, Sencan B, Omercioglu E, Ozmert EN. The impact of the COVID-19 pandemic on children with special needs: A descriptive study. Clin Pediatr. 2022;61(2):141–9. | Excluded | Did not explore telehealth applications |
| 423 | Lipkin M, Crepeau-Hobson F. The impact of the COVID-19 school closures on families with children with disabilities: A qualitative analysis. Psychol Sch. 2023;60(5):1544–59. | Excluded | Did not explore telehealth applications |
| 424 | Ali D, O’Brien S, Hull L, Kenny L, Mandy W. "The key to this is not so much the technology. It’s the individual who is using the technology": Perspectives on telehealth delivery for autistic adults during the COVID-19 pandemic. Autism. 2023;27(2):552–64. | Excluded | Adult sample |
| 425 | Newbutt N, Schmidt MM, Riva G, Schmidt C. The possibility and importance of immersive technologies during COVID-19 for autistic people. Journal of Enabling Technologies. 2020;14(3):187–99. | Excluded | Explored virtual reality, machine learning or robotic applications |
| 426 | Hedley D, Brewer N, Nevill R, Uljarevic M, Butter E, Mulick JA. The relationship between clinicians’ confidence and accuracy, and the influence of child characteristics, in the screening of autism spectrum disorder. Journal of Autism and Developmental Disorders. 2016;46(7):2340. | Excluded | Did not explore telehealth applications |
| 427 | Pandina G. The role of digital medicine in autism spectrum disorder. Eur Neuropsychopharmacol. 2021;48:42–4. | Excluded | Theoretical paper |
| 428 | Martin RFK, Leppink-Shands P, Tlachac M, Dubois M, Conelea C, Jacob S, et al. The use of immersive environments for the early detection and treatment of neuropsychiatric disorders. Front Digit Health. 2021;2:576076. | Excluded | Review study |
| 429 | Barnhardt EW, Steingass K, Levine A, Jurbank M, Piercefield J, Nyp SS. The value of telehealth and a team-based approach in improving developmental and behavioral care during the COVID-19 pandemic. J Dev Behav Pediatr. 2021;42(7):602–4. | Excluded | Case study |
| 430 | Avlund SH, Thomsen PH, Schendel D, Jørgensen M, Clausen L. Time trends in diagnostics and clinical features of young children referred on suspicion of autism: A population-based clinical cohort study, 2000–2010. Journal of Autism and Developmental Disorders. 2021;51(2):444–58. | Excluded | Did not explore telehealth applications |
| 431 | Jones LS, Russell A, Collis E, Brosnan M. To what extent can digitally-mediated team communication in children’s physical health and mental health services bring about improved outcomes? A systematic review. Child Psychiatry Hum Dev. 2022;53(5):1018–35. | Excluded | Review study |
| 432 | Sánchez-García AB, Galindo-Villardón P, Nieto-Librero AB, Martín-Rodero H, Robins DL. Toddler screening for autism spectrum disorder: A meta-analysis of diagnostic accuracy. Journal of Autism and Developmental Disorders. 2019;49(5):1837–52. | Excluded | Review study |
| 433 | Sibley MH, Ortiz M, Gaias LM, Reyes R, Joshi M, Alexander D, et al. Top problems of adolescents and young adults with ADHD during the COVID-19 pandemic. J Psychiatr Res. 2021;136:190–7. | Excluded | Did not explore telehealth applications |
| 434 | Corona LL, Wagner L, Wade J, Weitlauf AS, Hine J, Nicholson A, et al. Toward novel tools for autism identification: Fusing computational and clinical expertise. Journal of Autism and Developmental Disorders. 2021;51(11):4003–12. | Excluded | Did not examine relevant outcomes |
| 435 | Tomlinson SRL, Gore N, McGill P. Training individuals to implement applied behavior analytic procedures via telehealth: A systematic review of the literature. J Behav Educ. 2018;27(2):172–222. | Excluded | Examined/ elaborated on an intervention |
| 436 | Salinas CM, Bordes Edgar V, Berrios Siervo G, Bender HA. Transforming pediatric neuropsychology through video-based teleneuropsychology: An innovative private practice model pre-COVID-19. Arch Clin Neuropsychol. 2020;35(8):1189–95. | Excluded | Did not examine relevant outcomes |
| 437 | Garcia J, Brazendale K, Lee E. Transition of an in-person culinary program to remote delivery during the COVID-19 pandemic for adolescents with autism spectrum disorder. J Allied Health. 2021;50(1):86–6. | Excluded | Conference abstract |
| 438 | Awasthi S, Aravamudhan S, Jagdish A, Joshi B, Mukherjee P, Kalkivaya R, et al. Transitioning ABA services from in-clinic to telehealth: Case study of an Indian organization’s response to COVID-19 lockdown. Behav Anal Pract. 2021;14(4):893–912. | Excluded | Examined/ elaborated on an intervention |
| 439 | Wagner L, Weitlauf AS, Hine J, Corona LL, Berman AF, Nicholson A, et al. Transitioning to telemedicine during COVID-19: Impact on perceptions and use of telemedicine procedures for the diagnosis of autism in toddlers. J Autism Dev Disord. 2022;52(5):2247–57. | Included |  |
| 440 | Joudar SS, Albahri AS, Hamid RA. Triage and priority-based healthcare diagnosis using artificial intelligence for autism spectrum disorder and gene contribution: A systematic review. Comput Biol Med. 2022;146:105553. | Excluded | Review study |
| 441 | D’Aprano AL, Carapetis JR, Andrews R. Trial of a developmental screening tool in remote Australian Aboriginal communities: A cautionary tale. J Paediatr Child Health. 2011;47(1):12–7. | Excluded | Non-ASD sample |
| 442 | Parr J, Wigham S, Farr W, Reddy V, Male I. UK childhood autism diagnostic services survey 2020: Evidence for challenges and innovations. Arch Dis Child. 2021;106:A238. | Excluded | Conference abstract |
| 443 | Jones MK, Zellner MA, Hobson AN, Levin A, Roberts MY. Understanding caregiver satisfaction with a telediagnostic assessment of autism spectrum disorder. Am J Speech Lang Pathol. 2022;31(2):982–90. | Included |  |
| 444 | Schutte JL, McCue MP, Parmanto B, McGonigle J, Handen B, Lewis A, et al. Usability and reliability of a remotely administered adult autism assessment, the autism diagnostic observation schedule (ADOS) module 4. Telemed J E Health. 2015;21(3):176–84. | Excluded | Adult sample |
| 445 | Schutte JL. Usability and reliability of Autism Diagnostic Observation Schedule (ADOS) module 4 remote administration [Internet]. Vol. 82, Dissertation Abstracts International: Section B: The Sciences and Engineering. ProQuest Information & Learning; 2021. Available from: <https://search.ebscohost.com/login.aspx?direct=true&db=psyh&AN=2021-61327-280&site=ehost-live> | Excluded | Adult sample |
| 446 | Campbell K, Carpenter KLH, Espinosa S, Hashemi J, Qiu Q, Tepper M, et al. Use of a digital Modified Checklist for Autism in Toddlers–Revised with follow-up to improve quality of screening for autism. J Pediatr. 2017;183:133–9. | Excluded | Examined a mobile application |
| 447 | Ford K, Wang M, Koegel LK, Koegel RL, Fedders A. Use of a videoconferencing intervention and systematic hierarchy to teach daily living skills to young adults with autism spectrum disorder. J Positive Behav Interventions. 2021;23(2):81–92. | Excluded | Adult sample |
| 448 | Goldberg WA, Thorsen KL, Osann K, Spence MA. Use of home videotapes to confirm parental reports of regression in autism. Journal of Autism and Developmental Disorders. 2008;38(6):1136. | Excluded | Did not examine relevant outcomes |
| 449 | Alfuraydan M, Croxall J, Hurt L, Kerr M, Brophy S. Use of telehealth for facilitating the diagnostic assessment of Autism Spectrum Disorder (ASD): A scoping review. PLoS One. 2020;15(7):e0236415. | Excluded | Review study |
| 450 | Wallis KE, Mulé C, Mittal S, Cerda N, Shaffer R, Scott A, et al. Use of telehealth in fellowship-affiliated developmental behavioral pediatric practices during the COVID-19 pandemic. J Dev Behav Pediatr. 2021;42(4):314–21. | Excluded | Did not examine relevant outcomes |
| 451 | Esposito S, Rosafio C, Antodaro F, Argentiero A, Bassi M, Becherucci P, et al. Use of telemedicine healthcare systems in children and adolescents with chronic disease or in transition stages of life: Consensus document of Italian pediatric societies. J Pers Med. 2023;13(2):235. | Excluded | Non-ASD sample |
| 452 | Rynkiewicz A, Vasa R, Lucka I, Mazur A. Use of the Brief Observation of Symptoms of Autism (BOSA) as a new clinical approach during the COVID-19 pandemic. Pediatr Pol. 2020;95(4):241–3. | Excluded | No relevant data |
| 453 | Wagner L, Corona LL, Weitlauf AS, Marsh KL, Berman AF, Broderick NA, et al. Use of the TELE-ASD-PEDS for autism evaluations in response to COVID-19: Preliminary outcomes and clinician acceptability. J Autism Dev Disord. 2021;51(9):3063–72. | Included |  |
| 454 | Wood-Downie H, Ward V, Ivil K, Kovshoff H, Parsons S. Using digital stories for assessments and transition planning for autistic pre-school children. Educational and Child Psychology. 2021;38(3):62–74. | Excluded | Explored virtual reality, machine learning or robotic applications |
| 455 | Samadi SA, Bakhshalizadeh-Moradi S, Khandani F, Foladgar M, Poursaid-Mohammad M, McConkey R. Using hybrid telepractice for supporting parents of children with ASD during the COVID-19 lockdown: A feasibility study in Iran. Brain Sci. 2020;10(11):1–14. | Excluded | Did not explore telehealth applications |
| 456 | Gal E, Weiss PL (Tamar), Zancanaro M. Using innovative technologies as therapeutic and educational tools for children with autism spectrum disorder. In: Virtual Reality for Psychological and Neurocognitive Interventions. Cham: Springer Nature Switzerland AG; 2019. p. 227–46. | Excluded | Book chapter |
| 457 | Keith JM, Bennetto L. Using innovative technologies to assess self-regulation and social motivation in young children with and without autism spectrum disorder. Psychother Psychosom. 2022;91:29. | Excluded | Explored virtual reality, machine learning or robotic applications |
| 458 | Dubey I, Bishain R, Dasgupta J, Bhavnani S, Belmonte MK, Gliga T, et al. Using mobile health technology to assess childhood autism in low-resource community settings in India: An innovation to address the detection gap. Autism [Internet]. 2023. Available from: <https://journals.sagepub.com/home/AUT> | Excluded | Examined a mobile application |
| 459 | Wiggins LD, Reynolds A, Rice CE, Moody EJ, Bernal P, Blaskey L, et al. Using standardized diagnostic instruments to classify children with autism in the Study to Explore Early Development. Journal of Autism and Developmental Disorders. 2015;45(5):1271. | Excluded | Did not explore telehealth applications |
| 460 | Nicksic-Springer TK. Using technology to deliver home-based applied behavior analysis to children in foster care with autism [Internet]. Vol. 78, Dissertation Abstracts International Section A: Humanities and Social Sciences. ProQuest Information & Learning; 2017. Available from: <https://search.ebscohost.com/login.aspx?direct=true&db=psyh&AN=2017-16343-134&site=ehost-live> | Excluded | Examined/ elaborated on an intervention |
| 461 | Pickard KE, Ingersoll BR. Using the Double ABCX Model to integrate services for families of children with ASD. Journal of Child and Family Studies. 2017;26(3):810. | Excluded | Theoretical paper |
| 462 | Brodhead MT, Rispoli MJ. Using videos to assess preference for novel stimuli in children with autism. Dev Neurorehabil. 2017;20(8):560–4. | Excluded | Did not examine relevant outcomes |
| 463 | Holtman SJ, Winans KS, Hoch JD. Utility of diagnostic classification for children 0-5 to assess features of autism: Comparing in-person and COVID-19 telehealth evaluations. J Autism Dev Disord. 2022;52(12):5114–25. | Excluded | Did not examine relevant outcomes |
| 464 | Eslami Jahromi M, Ayatollahi H. Utilization of telehealth to manage the COVID-19 pandemic in low- and middle-income countries: A scoping review. J Am Med Informatics Assoc. 2023;30(4):738–51. | Excluded | Review study |
| 465 | Arias AA, Rea MM, Adler EJ, Haendel AD, Van Hecke AV. Utilizing the Child Behavior Checklist (CBCL) as an autism spectrum disorder preliminary screener and outcome measure for the PEERS® intervention for autistic adolescents. Journal of Autism and Developmental Disorders. 2022;52(5):2061–74. | Excluded | Did not explore telehealth applications |
| 466 | Farr W, Male I. Validation of a new digital tool, the Pirates Autism Assessment App, as an adjunct to assessment of primary school children referred with possible autism. Arch Dis Child. 2021;106:A56–7. | Excluded | Did not examine relevant outcomes |
| 467 | Attar SM, Bradstreet LE, Ramsey RK, Kelly K, Robins DL. Validation of the Electronic Modified Checklist for Autism in Toddlers, Revised with Follow-Up: A nonrandomized controlled trial. J Pediatr. 2023. | Excluded | No relevant data |
| 468 | Washington P, Kalantarian H, Tariq Q, Schwartz J, Dunlap K, Chrisman B, et al. Validity of online screening for autism: Crowdsourcing study comparing paid and unpaid diagnostic tasks. J Med Internet Res. 2019;21(5):e13668. | Excluded | Explored virtual reality, machine learning or robotic applications |
| 469 | Sutantio JD, Pusponegoro HD, Sekartini R. Validity of telemedicine for diagnosing autism spectrum disorder: Protocol-guided video recording evaluation. Telemed J E Health. 2021;27(4):427–31. | Excluded | Did not examine relevant outcomes |
| 470 | Cardon TA, Guimond A, Smith-Treadwell AM. Video modeling and children with autism spectrum disorder: A survey of caregiver perspectives. Educ Treat Children. 2015;38(3):403–20. | Excluded | Examined/ elaborated on an intervention |
| 471 | Thompson TL. Video modeling for children and adolescents with autism spectrum disorder: A meta-analysis [Internet]. Vol. 75, Dissertation Abstracts International Section A: Humanities and Social Sciences. ProQuest Information & Learning; 2014. Available from: <https://search.ebscohost.com/login.aspx?direct=true&db=psyh&AN=2014-99230-214&site=ehost-live> | Excluded | Did not explore telehealth applications |
| 472 | Russo-Ponsaran N, McKown C, Johnson J, Russo J, Crossman J, Reife I. Virtual environment for social information processing: Assessment of children with and without autism spectrum disorders. Autism Res. 2018;11(2):305–17. | Excluded | Explored virtual reality, machine learning or robotic applications |
| 473 | Good J, Parsons S, Yuill N, Brosnan M. Virtual reality and robots for autism: Moving beyond the screen. Journal of Assistive Technologies. 2016;10(4):211. | Excluded | Explored virtual reality, machine learning or robotic applications |
| 474 | Stasolla F. Virtual reality and wearable technologies to support adaptive responding of children and adolescents with neurodevelopmental disorders: A critical comment and new perspectives. Front Psychol [Internet]. 2021;12. Available from: <https://search.ebscohost.com/login.aspx?direct=true&db=psyh&AN=2021-70027-001&site=ehost-live> | Excluded | Explored virtual reality, machine learning or robotic applications |
| 475 | Ke F, Lee S. Virtual reality based collaborative design by children with high-functioning autism: Design-based flexibility, identity, and norm construction. Interactive Learning Environments. 2016;24(7):1511–33. | Excluded | Explored virtual reality, machine learning or robotic applications |
| 476 | Gleason LJ. Virtual reality technologies and autism spectrum disorder: Directors of special services’ perceptions [Internet]. Vol. 78, Dissertation Abstracts International Section A: Humanities and Social Sciences. ProQuest Information & Learning; 2018. Available from: <https://search.ebscohost.com/login.aspx?direct=true&db=psyh&AN=2017-33536-289&site=ehost-live> | Excluded | Explored virtual reality, machine learning or robotic applications |
| 477 | Prelack M, Fridinger S, Gonzalez AK, Kaufman MC, Xian J, Galer PD, et al. Visits of concern in child neurology telemedicine. Dev Med Child Neurol. 2022;64(11):1351–8. | Excluded | Did not examine relevant outcomes |
